# Exploring the Antimicrobial Action of Quaternary Amines against *Acinetobacter baumannii*

**DOI:** 10.1128/mBio.02394-17

**Published:** 2018-02-06

**Authors:** Gregory A. Knauf, Ashley L. Cunningham, Misha I. Kazi, Ian M. Riddington, Alexander A. Crofts, Vincent Cattoir, M. Stephen Trent, Bryan W. Davies

**Affiliations:** a Department of Molecular Biosciences, University of Texas at Austin, Austin, Texas, USA; b Center for Systems and Synthetic Biology, John Ring LaMontagne Center for Infectious Diseases, Institute for Cellular and Molecular Biology, University of Texas at Austin, Austin, Texas, USA; c Department of Chemistry, University of Texas at Austin, Austin, Texas, USA; d Department of Infectious Diseases, University of Georgia, College of Veterinary Medicine, Athens, Georgia, USA; e University of Rennes 1, Inserm Unit U1230, Rennes, France; f Department of Clinical Microbiology, University Hospital of Rennes, Rennes, France; g National Reference Center for Antimicrobial Resistance (lab 'Enterococci'), Rennes, France; Harvard Medical School

**Keywords:** *Acinetobacter baumannii*, BZK, antimicrobial, benzalkonium chloride, biocide, clostridium, proteostasis, quaternary amine

## Abstract

Quaternary amine compounds (QAC) are potent antimicrobials used to prevent the spread of pathogenic bacteria. While they are known for their membrane-damaging properties, QAC action has been suggested to extend beyond the surface to intracellular targets. Here we characterize the range of action of the QAC biocide benzalkonium chloride (BZK) against the bacterial pathogen *Acinetobacter baumannii*. At high concentrations, BZK acts through membrane disruption, but at low concentrations we show that wide-spread protein aggregation is associated with BZK-induced cell death. Resistance to BZK is found to develop through ribosomal protein mutations that protect *A. baumannii* against BZK-induced protein aggregation. The multifunctional impact of BZK led us to discover that alternative QAC structures, with low human toxicity, retain potent action against multidrug-resistant *A. baumannii*, *Staphylococcus aureus*, and *Clostridium difficile* and present opportunities for their development as antibiotics.

## IMPORTANCE

Quaternary amine compounds (QACs) are widely used to prevent the spread of bacterial pathogens, but our understanding of their mode of action is incomplete. Here we describe disruption of bacterial proteostasis as an unrecognized action of QAC antimicrobial action and uncover the potential of diverse QAC structures to act as multitarget antibiotics.

## INTRODUCTION

Chemical biocides are commonly used in clinical disinfection to prevent the spread of opportunistic ESKAPE pathogens (*Enterococcus faecium*, *Staphylococcus aureus*, *Klebsiella pneumoniae*, *Acinetobacter baumannii*, *Pseudomonas aeruginosa*, and *Enterobacter* spp.) and *Clostridium difficile* (1–4). Quaternary amine compounds (QACs) are among the most commonly used biocides and are considered nonspecific membrane-active agents (5). The model for their action proposes that their positively charged head group absorbs to acidic components of the bacterial cell envelope and that the long alkyl chains solubilize the membrane, leading to cell death (6, 7). However, several studies indicate that QACs also have intracellular effects that contribute to their antimicrobial action, with the critical lethal action being dependent on the exposure concentration (8–17). The concentrations of QACs recommended for routine use far exceed that required to eliminate vegetative bacteria under laboratory conditions. But practical conditions, including nonvegetative bacterial growth, the presence of biofilm and nonbiofilm organic matter, high ion concentrations, and pH, can all impact QAC action, allowing bacteria to persist and spread (18–21). Thus, understanding how concentration affects the mechanism of QAC action is critical to understanding and anticipating potential impacts on surviving bacteria.

Here we characterize the action of the commonly used QAC biocide benzalkonium chloride (BZK) against the ESKAPE pathogen *A. baumannii*. While at high concentrations BZK acts primarily through membrane damage, we show that at low concentrations disruption of cellular protein homeostasis (proteostasis) is associated with *A. baumannii* cell death. With this expanded mechanistic understanding, we demonstrate that alternative QAC structures, with low toxicity, still retain their antimicrobial action, opening new scaffolds for design of antibiotics and treatment of multidrug-resistant bacteria.

## RESULTS

### Cell envelope responses protect *A. baumannii* against BZK.

To explore the global effects of BZK exposure on *A. baumannii*, we used transposon insertion mutagenesis with deep sequencing (Tn-seq) and transcriptome sequencing (RNA-seq) to identify genes impacting *A. baummanii* fitness and/or transcriptional responsiveness to sublethal BZK exposure. We used *A. baumannii* strain 17978 for our studies since it has a well-characterized genome (22), is easy to genetically manipulate (23), and showed the median BZK MIC among the nine clinical and laboratory-passaged strains that we tested (see Table S1 in the supplemental material). The sensitivity of our *A. baumannii* strains to BZK was the same using either Mueller-Hinton (MH) broth or lysogeny broth (LB). We used LB for all of the following experiments to maintain a constant growth medium throughout our studies.

10.1128/mBio.02394-17.6 TABLE S1  *A. baumannii* benzalkonium chloride (BZK) MICs determined by agar and liquid microdilution methods. Download TABLE S1, PDF file, 0.02 MB. Copyright © 2018 Knauf et al. 2018 Knauf et al. This content is distributed under the terms of the Creative Commons Attribution 4.0 International license.

**FIG 1:**
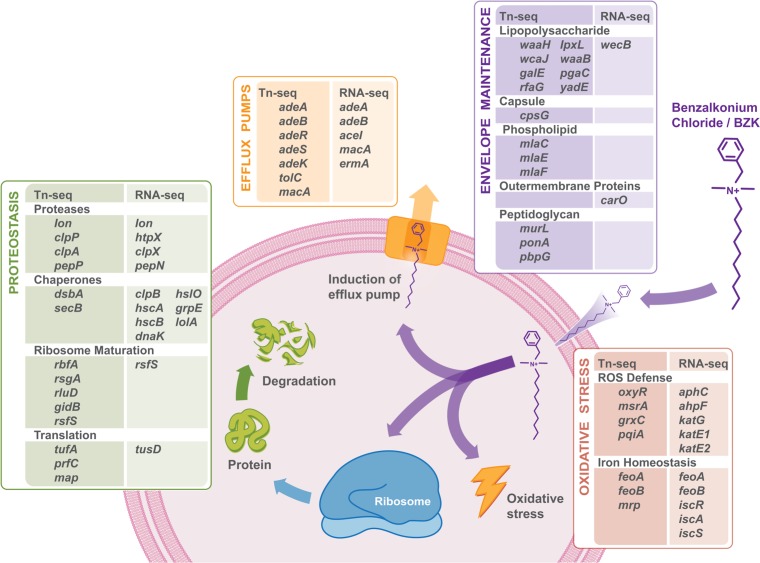
The structure of the C12 form of BZK is shown. Genes identified as promoting *A. baumannii* fitness in BZK (Tn-seq) and upregulated by BZK exposure (RNA-seq) encode functions related to cell envelope maintenance, drug efflux pumps, proteostasis, and oxidative stress defense. Selected genes for each functional group identified in our analysis are shown. ROS, reactive oxygen species.

For our Tn-seq analysis, we generated and grew an *A. baumannii* 17978 mutant library (~90,000 transposons) in a sublethal BZK dose (5 μg/ml) to saturation. We anticipated that *A. baumannii* transposon mutants deficient in processes required for BZK resistance would be less fit and would be outcompeted by the remaining population (Fig. 1) (Table S2). In parallel, we used RNA-seq to identify genes induced by the same BZK concentration. We focused on genes that increased in expression with the hypothesis that *A. baumannii* would differentially upregulate cellular functions important for BZK resistance (Fig. 1) (Table S3). We used a cutoff of a ≥2-fold (false-discovery-rate [FDR]-corrected *P* value of <0.01) decrease in fitness (Tn-seq) or increase in expression (RNA-seq) to broadly capture genes and cellular processes influencing the impact of BZK on *A. baumannii*. This identified 227 genes that promoted *A. baumannii* BZK fitness and 335 genes induced by BZK exposure (Fig. 1) (Table S2 and S3). We analyzed our data sets for gene ontology functional enrichment as previously described (24), but did not identify overrepresented metabolic or cellular processes associated with annotated categories. However, manual inspection identified several genes encoding functions related to four broad categories: cell envelope maintenance, drug efflux, proteostasis, and oxidative stress defense (Fig. 1) (Table S2 and S3).

10.1128/mBio.02394-17.7 TABLE S2  Results from Tn-seq analysis performed to identify genes that impact *A. baumannii* fitness in the presence of BZK. Genes showing a ±2-fold decrease or a greater effect (*P* < 0.01) are indicated. Color-coded genes are also shown in Fig. 1. Download TABLE S2, PDF file, 0.5 MB. Copyright © 2018 Knauf et al. 2018 Knauf et al. This content is distributed under the terms of the Creative Commons Attribution 4.0 International license.

10.1128/mBio.02394-17.8 TABLE S3  Results from RNA-seq analysis performed to identify genes that change expression in *A. baumannii* in the presence of BZK. Genes showing an effect greater than ±2-fold (*P* < 0.01) are indicated. Color-coded genes are also shown in Fig. 1. Download TABLE S3, PDF file, 0.7 MB. Copyright © 2018 Knauf et al. 2018 Knauf et al. This content is distributed under the terms of the Creative Commons Attribution 4.0 International license.

Cell envelope maintenance functions were prominent in our Tn-seq analysis and included genes encoding outer membrane proteins and enzymes functioning in lipo-oligosaccharide (LOS) synthesis, phospholipid retrograde maintenance, and peptidoglycan synthesis. This supports the idea of the importance of cell envelope integrity in BZK action. We hypothesized that as a critical target of BZK action, *A. baumannii* may induce expression of genes to bolster or alter cell envelope functions. Interestingly we identified increased expression in only two genes associated with cell envelope functions (*carO*, *wecB*) (Fig. 1) (Table S3). This was intriguing since other cationic membrane-active antimicrobials, such as polymyxins (e.g., polymyxin B, colistin), that bind to and affect the cell surface induce expression of several genes that alter the cell envelope (25). Strain R2, a polymyxin-resistant *A. baumannii* mutant that has an altered LOS charge, was previously isolated (26). We replicated the polymyxin resistance of the R2 strain (see Fig. S1A in the supplemental material), but found that the R2 and parental strains were identical in their sensitivities to BZK (Fig. S1B). This agrees with an earlier study that showed that a *Salmonella enterica* serovar Typhimurium strain with altered surface charge showed no change in BZK sensitivity (27). These results support the idea of the importance of cell envelope integrity for BZK resistance, but indicate that BZK likely interacts with *A. baumannii* membranes differently from cationic-membrane-active agents such as polymyxins.

10.1128/mBio.02394-17.1 FIG S1  Surface charge alterations affect *A. baumannii* polymyxin resistance but not BZK resistance. (A) Colistin sensitivity of wild-type *A. baumannii* and R2 mutant. The colistin resistance of the indicated strains was determined using an *E* test colistin strip. The black arrows indicate the MIC. All experiments were repeated at least three times. A representative image of each is shown. (B) The levels of plating efficiency of *A. baumannii* wild-type (WT) strain and R2 mutant strain CFUs on increasing BZK concentrations were identical. Download FIG S1, PDF file, 1.2 MB. Copyright © 2018 Knauf et al. 2018 Knauf et al. This content is distributed under the terms of the Creative Commons Attribution 4.0 International license.

Drug efflux systems allow bacteria to expel toxic compounds from the cytoplasm, inner membrane, and periplasm (28). We identified *adeA* and *adeB*, which encode subunits of a multidrug efflux system, as strongly induced by BZK and important for *A. baumannii* fitness (Fig. 1). Insertions in *adeA* and *adeB* or in *adeR and adeS*, encoding the two-component regulatory system, decreased *A. baumannii* fitness during BZK exposure (Table S2). BZK induced *adeAB* expression as measured by RNA-seq (Table S3). An *A. baumannii* 17978 Δ*adeB* strain showed a 5-log decrease in colony formation on agar containing BZK compared to the wild-type strain, supporting the idea of the importance of this efflux pump in *A. baumannii* defense against BZK (Fig. S2). AdeA and AdeB commonly function with a third component, AdeC, to form the AdeABC tripartite drug efflux system. *A. baumannii* 17978 does not encode AdeC, but does encode a homolog, AdeK, which was identified as affecting *A. baumannii* fitness in our Tn-seq analysis and which may function with AdeA and AdeB. Our results support data from previous studies linking AdeABC to BZK and broader biocide susceptibility in *A. baumannii* (29–31) and suggest an action for BZK past the outer membrane.

10.1128/mBio.02394-17.2 FIG S2  Plating efficiency of *A. baumannii* wild-type (parental) strain and Δ*adeB* mutants on increasing concentrations of BZK agar. At 12 μg/ml, the wild-type strain showed higher plating efficiency; *P* < 0.05 (unpaired two-tailed Student’s *t* test). At 12 μg/ml, the wild-type strain + vector showed higher plating efficiency than the mutant strain + vector, but not the complemented strain; *P* < 0.05 (one-way ANOVA, Tukey posttest). Download FIG S2, PDF file, 0.3 MB. Copyright © 2018 Knauf et al. 2018 Knauf et al. This content is distributed under the terms of the Creative Commons Attribution 4.0 International license.

### Intracellular responses protect *A. baumannii* against BZK.

**FIG 2:**
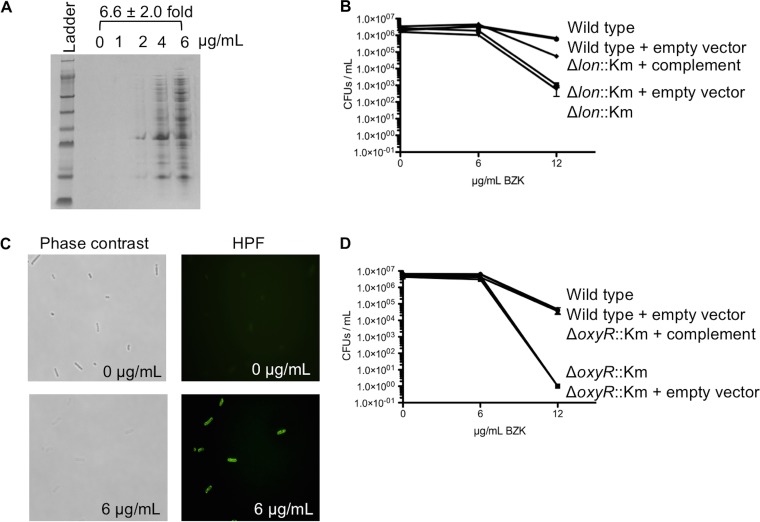
BZK impacts proteostasis and oxidative stress response. (A) Protein aggregates from *A. baumannii* wild type (WT) treated with increasing levels of BZK. The amount of aggregate loaded from each sample was normalized by cell number. The experiment was repeated at least three times, with a representative result shown. Treatment with 6 µg/ml BZK resulted in a 6.6-fold ± 2.0-fold increase in aggregates compared to no treatment; *P* < 0.05 (unpaired two-tailed Student’s *t* test). (B) Plating efficiency of *A. baumannii* wild-type strain and Δ*lon* mutant on increasing concentrations of BZK agar. At 12 μg/ml, the wild-type strain showed higher plating efficiency than the mutant; *P* < 0.05 (unpaired two-tailed Student’s *t* test). At 12 μg/ml, the combination of the wild-type strain with vector (Wild type + empty vector) showed higher plating efficiency than the mutant + vector and the complemented strain; *P* < 0.05 (one-way analysis of variance [ANOVA], Tukey posttest). (C) Phase-contrast and HPF fluorescence microscopy images of wild-type *A. baumannii* with and without 6 µg/ml sub-MIC BZK treatment. (D) Plating efficiency of *A. baumannii* wild-type strain and Δ*oxyR* mutant on increasing concentrations of BZK agar. At 12 μg/ml, the wild-type strain showed higher plating efficiency than the mutant; *P* < 0.05 (unpaired two-tailed Student’s *t* test). At 12 μg/ml, the wild-type strain +vector showed higher plating efficiency than the mutant + vector, but not than the complemented strain; *P* < 0.05 (one-way ANOVA, Tukey posttest).

Since BZK action is attributed to cell envelope disruption, we were intrigued that transposon insertions disrupting intracellular proteostasis and oxidative defense affected BZK fitness as strongly as many envelope maintenance functions in our Tn-seq analysis (Fig. 1) (Table S2). Insertions in genes encoding products associated with ribosome biogenesis (RsgA, RluD, RimL), translation control (PrfC, Map), and damaged/aggregated protein turnover (Lon, ClpP) decreased *A. baumannii* BZK fitness (Table S2). Correlatively, BZK induced expression of several genes encoding products associated with damaged/aggregated protein turnover and protein folding functions (Lon, HtpX, ClpB, HscB) (Table S3). Similar proteostasis gene expression responses have been observed in bacteria following treatment with ribosomal antibiotics and have been linked to protein damage, with formation of deleterious protein aggregates (32–35). We assayed the impact of sublethal BZK on the proteome of *A. baumannii* and observed a dose-dependent increase in total protein aggregate levels (Fig. 2A), indicating that BZK was disrupting proteostasis. We assayed the ability of the membrane-disrupting cationic antibiotic colistin to induce protein aggregates. Treatment of *A. baumannii* with an equivalent sublethal dose of colistin did not induce protein aggregates (Fig. S3A), indicating that the membrane disruption alone was likely not inducing the observed aggregate formation. Many proteins share overlapping functions in the prevention and the turnover of protein aggregates, diminishing the effect of loss of any one proteostasis factor (36, 37). Nevertheless, we did find that deletion of Lon protease alone was sufficient to decrease the ability of *A. baumannii* to form colonies on agar containing BZK, further linking effects on proteostasis with BZK action (Fig. 2B). Correlatively, more protein aggregates accumulated in the Δ*lon* strain than in the wild type following BZK exposure (Fig. S3B and C).

10.1128/mBio.02394-17.3 FIG S3  Protein aggregates are not induced by colistin and are more abundant in Δ*lon* mutants than in wild-type cells. (A) Protein aggregates from *A. baumannii* treated with and without sublethal colistin. The amount of aggregate loaded was normalized by cell number, and the experiments were performed in duplicate, with a representative image presented here. (B) Representative image of protein aggregates from the *A. baumannii* wild-type (WT) strain and the Δ*lon* mutant following 4 µg/ml BZK treatment. A 4-μg/ml volume of BZK was used due to the increased sensitivity of the Δ*lon* mutant. The amount of aggregate loaded was normalized by cell number. (C) Quantitation of the aggregates from quadruplicate biological replicates. The statistical significance of the increase in aggregates between the Δ*lon* mutant and the WT treated with 4 µg/ml BZK is noted; *P* < 0.05 (one-way ANOVA, Tukey posttest). Download FIG S3, PDF file, 0.8 MB. Copyright © 2018 Knauf et al. 2018 Knauf et al. This content is distributed under the terms of the Creative Commons Attribution 4.0 International license.

Tn-seq indicated that the master oxidative stress regulator OxyR contributed to BZK resistance (Fig. 1) (Table S2), and expression levels of OxyR-regulated genes, including all genes encoding catalases (*kat*) and alkyl hydroperoxidases (*ahp*), were induced following BZK exposure (Fig. 1) (Table S3). We visualized BZK-induced oxidative stress using the redox-sensitive fluorescent dye hydroxyphenyl fluorescein (HPF), which accumulates in the cell and fluoresces upon oxidation. Exposure of *A. baumannii* to sublethal BZK levels, and treatment with HPF, resulted in highly fluorescent cells (Fig. 2C), supporting the idea of an induction of oxidative stress by BZK. Furthermore, deletion of *oxyR* decreased the ability of *A. baumannii* to form colonies on BZK agar, supporting the idea of a role for oxidative stress in BZK-induced lethality (Fig. 2D).

### Ribosomal protein mutations promote BZK resistance.

**FIG 3:**
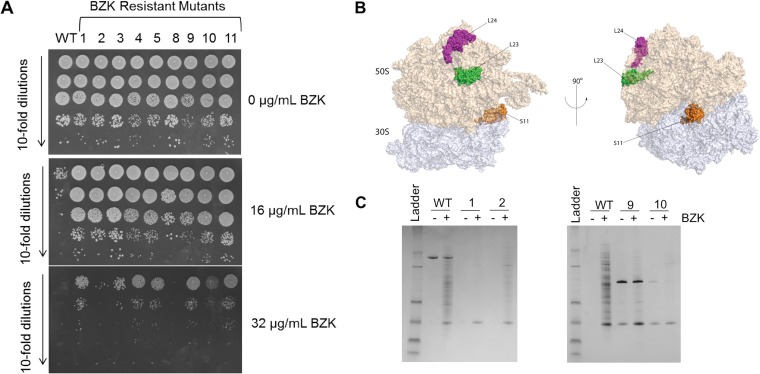
BZK-resistant mutant phenotypes and ribosome locations. (A) Plating efficiency of *A. baumannii* 17978 (WT) and BZK-resistant mutants on 0, 16, and 32 μg/ml BZK plates. (B) Ribosomes showing locations of proteins with mutations affecting BZK sensitivity. (C) Protein aggregates from *A. baumannii* 17978 (WT) and BZK-resistant mutants 1, 2, 9, and 10 with (+) or without (-) 6 μg/ml sub-MIC BZK treatment. The amount of aggregate loaded from each sample was normalized by cell number.

Our results indicate that BZK can act through disruption of intracellular processes, but the potential target(s) remained unclear. Target mutation is a common mechanism by which drug resistance is acquired. To leverage this unbiased approach, we performed a single-passage selection for spontaneous *A. baumannii* mutants that could grow on agar containing twice the concentration of BZK that the parental strain can tolerate. After a single passage, we isolated 11 *A. baumannii* mutants whose agar MIC had increased from 16 μg/ml to 32 μg/ml. This corresponds to a 100× to 1,000× increase in CFU survival when spotted on BZK-containing medium (Fig. 3A). We sequenced the genomes of these 11 BZK-resistant mutants and compared them to the resequenced parental genome to identify potential mutations. We identified unique mutations in 9 of the 11 BZK resistant strains, each confirmed by Sanger sequencing. Remarkably, 8 of 9 isolates carried mutations in ribosomal proteins or the untranslated region (UTR) between two ribosomal protein operons (Table 1). Among the 9 mutants, 7 had a single mutation. Mutants 3, 10, and 11 each had a unique mutation in the 50S protein L24. The mutation in isolate 1 was found in the UTR between two ribosomal protein operons. The mutations in isolates 2 and 9 were found in L23 and S11, respectively. Mutants 4 and 5 appeared to be siblings as they shared two identical mutations in S11 and SecY. Ribosomal proteins L23 and L24 are located together near the exit tunnel and interact with SecY during protein secretion (38), while S11 is a component of the ribosomal P site and interacts with S7 to influence translation fidelity (39) (Fig. 3B). Mutant 8 carried a single mutation in a gene coding for a hypothetical protein of unknown function.

**TABLE 1:** Mutation locations of BZK-resistant *A. baumannii* mutants

Strain	BZK MIC (µg/ml)^a^	Protein/feature- encoding mutation	Amino acid change
Parental	16		
Mutant 1	32	Ribosome protein operon UTR	None
Mutant 2	32	L23	I42F
Mutant 3	32	L24	Frameshift
Mutant 10	32	L24	G15V
Mutant 11	32	L24	I4N
Mutant 9	32	S11	D111Y
Mutant 4^b^	32	S11	A62D
Mutant 5^b^	32	S11	A62D
Mutant 4^b^	32	SecY	L306I
Mutant 5^b^	32	SecY	L306I
Mutant 8	32	Hypothetical (A1S_1648)	S137F

a Data represent MIC values determined by the agar microdilution method.

b The identical two mutations were identified in these isolates.

### BZK-resistant mutants protect against protein aggregation.

Our omic results, combined with our observation of BZK-induced protein aggregation and of BZK resistance resulting from ribosomal protein mutations, suggested that part of the antimicrobial action of BZK may occur through disruption of proteostasis. However, ribosome mutations can have pleiotropic effects, including affecting bacterial growth rate, which can influence drug action. We tested the BZK-resistant mutants and found they all had growth rates similar to that of the parental strain, suggesting that their BZK resistance was not due to growth effects (Fig. S4). We also considered that BZK-resistant mutants might indirectly strengthen cell membranes against BZK. We assayed the membrane permeability of these mutants by measuring propidium iodide (PI) uptake. However, we found no significant difference in the levels of PI uptake between the parental and mutant strains following BZK treatment (Fig. S5A).

10.1128/mBio.02394-17.4 FIG S4  Exponential growth of the wild-type strain and BZK-resistant *A. baumannii* mutants in LB medium measured by CFU. All strains were measured in biological triplicate. Data are represented as means ± standard errors of the means (SEM). Download FIG S4, PDF file, 0.5 MB. Copyright © 2018 Knauf et al. 2018 Knauf et al. This content is distributed under the terms of the Creative Commons Attribution 4.0 International license.

10.1128/mBio.02394-17.5 FIG S5  (A) Flow cytometry analysis of membrane damage caused by BZK against the *A. baumannii* parental strain and BZK-resistant mutants measured by cell uptake of propidium iodide. Increased fluorescent intensity indicates increased propidium iodide uptake and membrane damage. Equal numbers of cells were counted under all conditions. (B) Effect of BZK and erythromycin (Erm) on *in vitro* translation of DHFR. Only the results of Erm treatment show a difference in the levels of DHFR production; *, *P* < 0.05 (one-way ANOVA with Dunnett’s multiple-comparison test using the untreated control). Download FIG S5, PDF file, 0.5 MB. Copyright © 2018 Knauf et al. 2018 Knauf et al. This content is distributed under the terms of the Creative Commons Attribution 4.0 International license.

A study in *Mycobacterium smegmatis* indicated that ribosomal protein mutations could promote resistance to several classes of antibiotics (40). We determined the activity of antibiotics targeting different cellular pathways against the BZK-resistant mutants (Table 2). The levels of sensitivity to the membrane-damaging antibiotic polymyxin B did not differ between the parental and mutant strains (Table 2), further indicating they do not have altered cell membranes. Mutants with mutations affecting L23 and L24 and the UTR upstream of the L24 operon showed increased resistance to erythromycin, but not to other ribosomal antibiotics. Different subsets of BZK-resistant mutants conferred resistance to rifampin, ciprofloxacin, and carbenicillin, while other subsets conferred increased sensitivity to telithromycin and gentamicin (Table 2).

**TABLE 2:** Antibiotic sensitivity of BZK-resistant *A. baumannii* mutants

Strain	Mutation site(s)	MIC (µg/ml)^a^									
		BZK	Pxb	Erm	Tem	Clm	Gem	Tet	CIP	Rif	Cbn
Parental		16	1	8	8	64	8	1	0.25	2	32
Mutant 1	UTR	32	1	16	8	64	8	1	1	4	32
Mutant 2	L23	32	1	16	8	64	8	1	0.5	4	32
Mutant 3	L24	32	1	16	8	64	8	1	0.5	4	32
Mutant 4^b^	S11, SecY	32	1	8	8	64	4	1	0.5	4	64
Mutant 5^b^	S11, SecY	32	1	8	8	64	4	1	0.5	4	64
Mutant 8	A1S_1648	32	1	8	8	64	8	1	0.25	2	32
Mutant 9	S11	32	1	8	4	64	8	1	0.5	2	32
Mutant 10	L24	32	1	8	8	64	4	1	1	4	64
Mutant 11	L24	32	1	16	8	64	8	1	1	4	32

a Data represent MIC values determined by the agar microdilution method. BZK, benzalkonium chloride; Cbn, carbenicillin; Cip, ciprofloxacin; Clm, clindamycin; Erm, erythromycin; Gem, gentamicin; Pxb, polymyxin B; Rif, rifampin; Tem, telithromycin; Tet, tetracycline.

b The identical two mutations were identified in these isolates.

While it is unclear how ribosomal mutations influence the activity of these various antibiotics, we hypothesized that the ribosomal protein mutations may confer resistance to BZK by stabilizing the *A. baumannii* proteome. We treated mutants 1, 2, 9, and 10, representing distinct ribosome protein mutations, with BZK and analyzed their protein aggregate profiles. All four BZK-resistant mutants showed reduced protein aggregate formation compared to the parental strain (Fig. 3C). The correlation of reduced protein aggregation and BZK resistance further suggests that BZK may act in part through disruption of intracellular proteostasis.

We hypothesize that BZK may directly inhibit protein translation. We measured the effect of BZK on the translation of a known protein (dihydrofolate reductase [DHFR]) with an *in vitro Escherichia coli* transcription-translation system. Addition of erythromycin to the *in vitro* reaction effectively inhibited translation of DHFR; however, BZK did not have an effect on *in vitro* DHFR translational activity (Fig. S5B).

The known effects of BZK on membrane integrity and the resistance mutations seen in secretion-related proteins such as SecY, L23, and L24 (Table 1) led us to question whether BZK might affect some classes of proteins more than others. We analyzed the protein content of aggregates in *A. baumannii* with and without sub-MIC BZK treatment by mass spectroscopy (Table S4). While the treated sample produced significantly more total aggregates, the two samples shared the majority of identified proteins, 1,388 proteins, in common. Only three proteins were unique to the untreated sample and seven to the treated sample. We also did not identify any major enrichment of specific cellular classes of proteins using Gene Ontology (GO) terms (Table S4). This result suggests that the effects of BZK on proteostasis are not specific to any one class of protein.

10.1128/mBio.02394-17.9 TABLE S4  Results from mass spectrometry analysis of BZK-treated and untreated aggregates in biological duplicate. All proteins detected are shown with each samples' relative abundance (normalized emPAI value), values of fold change between the treated and untreated duplicate samples, their *P* values determined by *t* test, and associated GO term categories. "INF" denotes an infinite increase; "X" denotes whether a protein is associated with each column's GO term. Download TABLE S4, PDF file, 1.4 MB. Copyright © 2018 Knauf et al. 2018 Knauf et al. This content is distributed under the terms of the Creative Commons Attribution 4.0 International license.

### Therapeutic QACs have antimicrobial action.

QAC biocides were developed for their membrane action and favor long alkyl tails, which also promote cytotoxicity and exclude their use as antibiotics (13). However, our results implicating additional processes in BZK action led us to ask if QACs with less-toxic structures could still retain antimicrobial activity. We identified three candidates (Table 3); bretylium tosylate and clofilium tosylate are antiarrhythmic agents, while otilonium bromide (OB) is a muscarinic receptor inhibitor used to treat irritable bowel syndrome (IBS) (41–43). We tested each QAC against *A. baumannii* 17978 and multidrug-resistant (MDR) *A. baumannii* AYE. Since QACs are frequently more effective against Gram-positive bacteria (6), we also tested *Staphylococcus aureus* and *Clostridium difficile*. Due to the instability of otilonium bromide (44), we measured the activity of these compounds by a minimal bactericidal concentration (MBC) assay (45) (Table 3). Bretylium tosylate showed no effect against any strain. Clofilium tosylate showed activity against *A. baumannii* strains and a more potent effect against *S. aureus* strains. Remarkably, otilonium bromide exhibited potent activity against all bacteria and was slightly more effective against *C. difficile* strain 630 than the antibiotic vancomycin (Table 3), which is often used for the treatment of severe *C. difficile* infections (46).

**TABLE 3:**
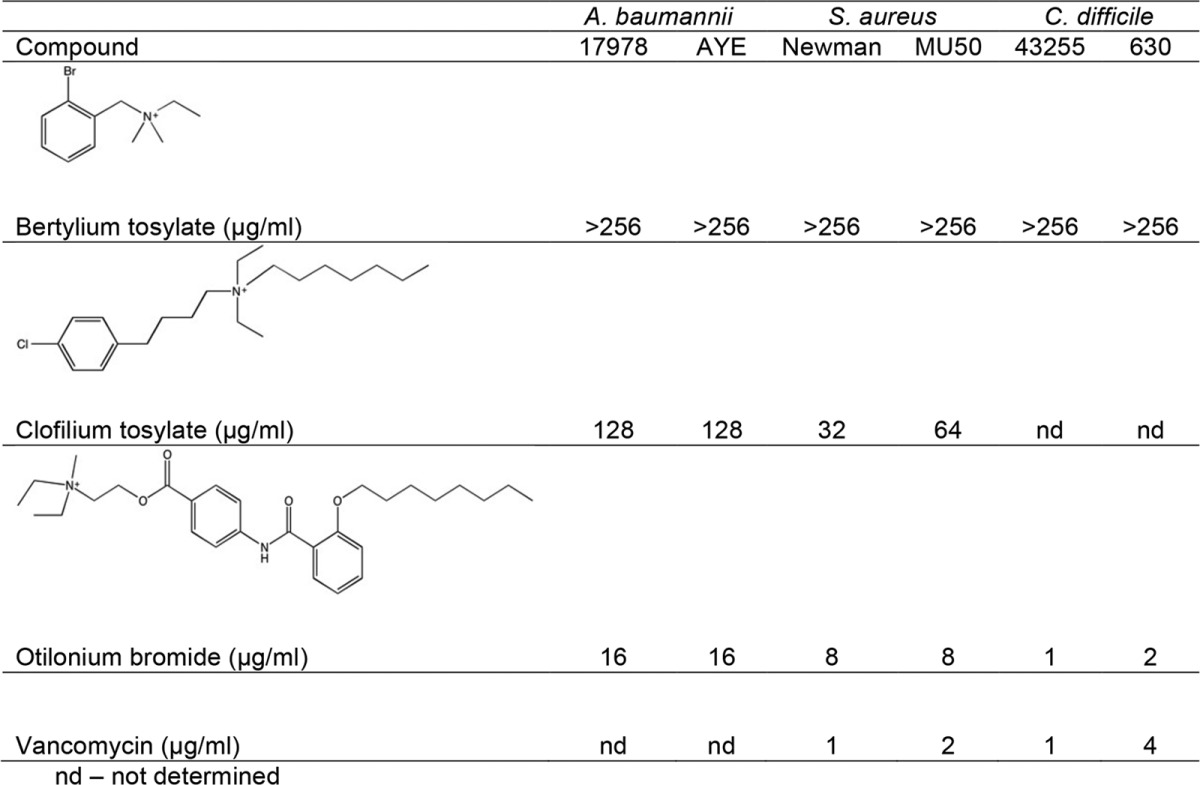
Minimal bactericidal concentration assay of clinically used and explored QACs and vancomycin

**FIG 4:**
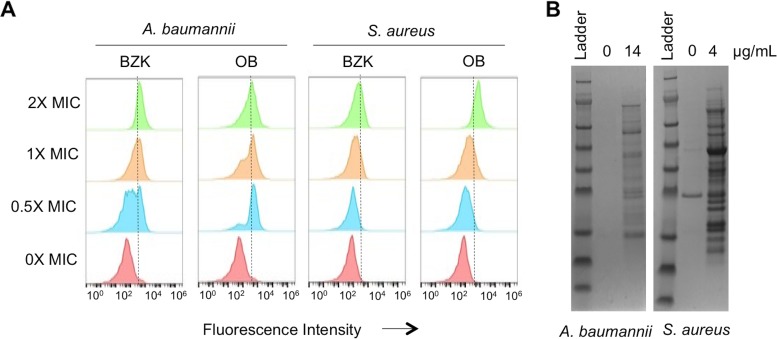
BZK and OB have similar cellular effects. (A) Membrane damage caused by BZK and OB at 0× to 2× MIC in *A. baumannii* and *S. aureus* measured by cell uptake of propidium iodide. Increased fluorescent intensity indicates increased propidium iodide uptake and membrane damage. Equal numbers of cells were counted under all conditions. (B) Subinhibitory OB treatment induces aggregate formation in *S. aureus* and *A. baumannii*. The amount of aggregate loaded from each sample was normalized by cell number as described in the Fig. 2 legend.

Otilonium bromide is structurally distinct from BZK. To determine if otilonium bromide acted similarly to BZK, we assayed its impact on membrane permeability and protein aggregation. We assayed membrane permeability by measuring uptake of PI following a sublethal treatment with otilonium bromide. Otilonium bromide increased the population fluorescence in *A. baumannii* and *S. aureus* in a dose-dependent manner, indicating that it was capable of inducing membrane permeability (Fig. 4A). Otilonium bromide also induced accumulation of protein aggregates in both *A. baumannii* and *S. aureus* following a sublethal exposure (Fig. 4B). Our results indicate that both membrane actions and proteostasis actions are conserved in antimicrobial QACs and that nontoxic QAC scaffolds represent potent leads for antibiotic development.

## DISCUSSION

QACs have been staple biocides since the 1930s (47). While QAC disruption of cell membranes is well established, its effects at low concentrations have remained unclear. Our results suggest that disruption of proteostasis is an important pathway of BZK antimicrobial action at low concentrations. Quaternary amines are excellent phase transfer catalysts, being soluble in both aqueous and organic solvents (48). In this context, the alkyl chain of BZK could mediate transition of the quaternary amine through hydrophobic cell membrane barriers to affect cytosolic factors controlling proteome homeostasis. The positively charged quaternary amine may interact with many negatively charged intracellular molecules such as RNA and DNA. During this transition, efflux pumps can be activated, which eliminates BZK from the cell. Our studies specifically implicate the AdeABC efflux system in *A. baumannii* BZK resistance. Expression of the AdeABC efflux pump has been linked to broad-spectrum antibiotic resistance in *Acinetobacter* spp. (30, 31). BZK-induced or BZK-selected upregulation of *adeABC* could support antibiotic cross-resistance. Our results demonstrate that BZK can select for ribosomal mutations that confer cross-resistance to different classes of antibiotics. This impact of ribosomal protein mutation was also observed for *M. smegmatis* (40). The potential for QAC-antibiotic cross-resistance has been reported in several studies (7); however, a consensus on its importance to public health has not been reached (49). Our discovery of BZK as a driver for ribosomal mutations that promote broad antibiotic resistance may help provide a focus on the public health impact of QAC-ribosomal antibiotic cross-resistance.

Our omic results, combined with our observation of BZK-induced protein aggregation and BZK resistance mediated by ribosomal protein mutations, suggest that part of the BZK antimicrobial action occurs through disruption of cellular proteostasis. How BZK elicits this effect is unclear. Our results indicated that BZK does not directly inhibit protein translation *in vitro*. While it is still possible that BZK directly acts on the ribosome *in vivo*, it may also influence ribosome association with other important proteostasis components. The single amino acid substitution L23 and L24 mutations may promote chaperone interactions to protect nascent polypeptides at the exit tunnel to prevent their aggregation. The chaperone Trigger Factor docks on L23 to interact with nascent peptides and protect them during maturation (50). Cryo-electron microscopy showed that L23 and L24 directly interact with SecY and nascent polypeptides (38). The mutations in L23, L24, and SecY conferring BZK resistance may alter the interactions of this complex to better chaperone proteins for export. While we did not identify a bias in proteins found in aggregate with and without BZK treatment, it is possible that the presence of an increased amount of specific proteins has a strong impact on BZK tolerance.

We also identified mutations in the small 30S subunit affecting S11. A previous study also reported a mutation in the S1 30S protein in an *E. coli* strain with increased BZK resistance (15). These mutations may have long-range effects or could implicate more than one ribosomal target in BZK action. Mutant 1 has a solitary mutation in a ribosomal protein operon untranslated region. This mutation potentially could interfere with transcription termination of the upstream operon. The result would be increased transcription from readthrough, potentially causing upregulation of the downstream ribosomal proteins, including L24. L24 is an initiator of 50S subunit assembly (51) which, if overexpressed, could potentially have important effects on 50S subunit assembly. The possibility of a similar type of effect on assembly cannot be excluded for mutants 3, 10, and 11, which have mutations in L24. Protection against protein aggregation also provides a rationale for the associated impact of oxidative stress on BZK survival. Damage to protein during synthesis has been proposed to disrupt membrane integrity and trigger increased production of reactive oxygen species (32). Damaged and unfolded proteins are in turn more susceptible to oxidative damage that can further amplify the proteome damage (33). However, the dramatic effect of otilonium bromide on *C. difficile*, which is grown anaerobically, indicates that the effects of oxidative stress are likely not as critical as the impact on protein quality control. Future studies to clarify BZK proteostasis effects and how ribosomal protein mutations facilitate BZK resistance will provide sharper insight into the full spectrum of BZK action.

Regardless of the details of the mechanism, our observation of the impact of BZK beyond the cell envelope led us to explore QACs with structures not typically associated with antimicrobial action. Our results highlight the possibility of using QACs as scaffolds for antibiotic development. Otilonium bromide shows potent activity against multidrug-resistant *A. baumannii*, *S. aureus*, and *C. difficile* and is safe for oral human use (42). It shows low toxicity through several routes of delivery (52, 53). At therapeutic doses, the concentration of otilonium bromide in the intestine and colonic smooth muscle reaches approximately 5 to 6 μg/ml, which is 2× to 3× higher than the MBC that we determined for *C. difficile*. The C8 alkyl tail is shorter than those typically associated with QAC activity (6), but is similar in length to the C9 tail of daptomycin. Otilonium bromide is an antimuscarinic used to treat IBS that acts by inhibiting muscarinic receptor-coupled calcium signaling (42). Advances in microbiome research have revealed important connections between our gut flora and IBS (54). Thus, in addition to its antimuscarinic activity, otilonium bromide may also alleviate IBS symptoms by eliminating antagonistic microbes. Our identification of an intracellular action for BZK shows similarities to the findings described for the biocide Tricolsan. Like BZK, triclosan disrupts cell membranes, but it was also shown to specifically inhibit the FabI enzyme (55). This finding has spurred pursuit of FabI inhibitors as antibiotics (56).

## MATERIALS AND METHODS

### Bacterial strains and plasmids used.

All bacterial strains and plasmids used, along with their sources, are described in Table S5 in the supplemental material.

10.1128/mBio.02394-17.10 TABLE S5  Bacterial strains and plasmids. Download TABLE S5, PDF file, 0.02 MB. Copyright © 2018 Knauf et al. 2018 Knauf et al. This content is distributed under the terms of the Creative Commons Attribution 4.0 International license.

### Growth conditions and genetic manipulations.

Bacteria were cultured in lysogeny broth (LB) broth at 37°C unless otherwise stated. *Clostridium difficile* was grown with brain heart infusion medium with yeast extract (BHIS) and reinforced clostridial medium (RCM). Carbenicillin (75 µg/ml), kanamycin (25 µg/ml), gentamicin (10 µg/ml), chloramphenicol (10 µg/ml), and tetracycline (10 µg/ml) were used for selection. Benzalkonium chloride was from Sigma and was a mixture of the C12 and C14 alkyl chain lengths. Clofilium tosylate and otilonium bromide (OB) were purchased from Sigma. Gene deletion and complementation were conducted as previously described (23).

### MICs, minimal bactericidal concentrations, and plating efficiency assay.

Agar dilution method MICs were determined using standard procedures in LB, BHIS, or RCM (57). All media containing quaternary amines or antibiotics were prepared on the day of use. Minimal bactericidal concentrations (MBC) to determine 99.9% viability reduction were conducted using standard procedures (45). For CFU assays, overnight cultures were diluted to approximately 10^6^ to 10^7^ CFU/ml. Each culture was serially diluted and plated onto agar plates with or without increasing concentrations of BZK. CFU counting was performed after overnight growth. All assays were performed in at least biological triplicate.

### Tn-seq and RNA-seq.

For Tn-seq analysis, an *A. baumannii* mutant transposon library (~90,000 transposons) for sequencing was constructed as previously described (58) using gentamicin and chloramphenicol to select for transconjugants. Library aliquots were diluted to ~10^7^ CFU/ml in 5 ml of LB medium with or without 5 μg/ml BZK and grown to saturation at 25°C with shaking. Bacteria were pelleted and processed for transposon sequencing as previously described (58). For RNA-seq, fresh *A. baumannii* colonies were collected from LB plates and diluted to ~10^8^ CFU/ml in 5 ml of LB growth medium. Cultures were grown and treated with or without 5 μg/ml BZK for 45 min at 25°C with shaking. The cells were pelleted and processed for RNA sequencing as previously described (59). Both RNA-seq and Tn-seq data were aligned to the *A. baumannii* 17978 genome and analyzed using CLC Genomics Workbench software and reads per kilobase per million (RPKM) values. The statistical test performed was a Baggerley’s test of the proportions of counts in each group of samples to generate a *P* value associated with the weighted proportions of fold change between the experiment and control groups for each gene. We used an arbitrary cutoff of 2-fold weighted proportion change with an FDR-corrected *P* of <0.01. Nonessential genes were identified for Tn-seq analysis based on previous results (60).

### BZK-resistant mutant isolation.

Two milliliters of a saturated *A. baumannii* culture (~1^10^/ml) was pelleted and suspended in 200 µl of LB. The 200-µl suspension was spread on LB agar containing 32 µg/ml BZK. The plates were incubated overnight at 37°C. After incubation, single colonies were picked and streaked on 16 µg/ml benzalkonium chloride LB plates. These plates were then incubated overnight and analyzed to confirm increased resistance to BZK. Samples that successfully grew on 16 µg/ml BZK after streaking had their MICs for BZK and antibiotics determined by the agar dilution method.

### Genomic variant analysis.

Single-nucleotide polymorphisms were identified as described previously (24). Briefly, extracted genomic DNA was processed for Illumina HiSeq sequencing using an NEB Next Ultra DNA Library Prep Kit. Genome sequences were aligned and single-nucleotide polymorphisms detected using CLC Genomic Workbench software. Variants were called with over 50-fold coverage at a frequency of 70%. False positives were manually checked for and removed from the results. All mutations were validated by Sanger sequencing.

### Fluorescence microscopy.

Exponentially growing *A. baumannii* cultures were normalized to an optical density at 600 nm (OD_600_) of ~0.4 and treated with 0 or 6 µg/ml BZK for 30 min with shaking at 37°C. One milliliter of each sample was pelleted and resuspended in 1× phosphate-buffered saline (PBS) with 5 µM 3′-(p-hydroxyphenyl)fluorescein (Thermo Fisher H36004). The suspensions were incubated in the dark for 15 min and then resuspended in 1 ml of fresh PBS. Ten microliters of the cell suspension was then allocated onto a slide and imaged.

### Protein aggregate isolation.

Cellular protein aggregate isolation and analysis were performed as previously described (37). Briefly, 50-ml LB cultures of exponentially growing bacteria were treated with the indicated concentration of BZK or otilonium bromide. Following isolation, aggregates were normalized and separated by SDS-PAGE and Coomassie stained for visualization. Aggregates were quantified using the area density feature in VisionWorks LS software (UVP, Inc.) on an image of the aggregate gel.

### Aggregate proteomics.

Protein identification was provided by the Proteomics Facility at the University of Texas at Austin following previously published procedures (61). Scaffold (version Scaffold_4.8.2; Proteome Software Inc., Portland, OR) was used to validate tandem mass spectrometry (MS/MS)-based peptide and protein identifications. Tandem and Sequest were set up to search acinetobacter_07-15.fasta (unknown version; 3,798 entries), assuming the presence of the digestion enzyme trypsin. Peptide identifications were accepted if they could be established at greater than 89.0% probability to achieve an FDR of less than 1.0%. Peptide probabilities evaluated using Sequest software were assigned by the Scaffold Local FDR algorithm. Peptide probabilities evaluated using X! Tandem software were assigned by the Peptide Prophet algorithm (62) with Scaffold delta-mass correction. Protein identifications were accepted if they could be established to achieve an FDR of less than 5.0% at greater than 99.0% probability and contained at least 2 identified peptides. Protein probabilities were assigned by the Protein Prophet algorithm (63). Proteins that contained similar peptides and that could not be differentiated based on MS/MS analysis alone were grouped to satisfy the principles of parsimony. Proteins were annotated with Gene Ontology (GO) terms from gene_association.goa_uniprot (downloaded 14 January 2015) (64). Relative abundances were quantified by the use of normalized exponentially modified protein abundance index (emPAI) values, with a minimal value setting of 0.

### Flow cytometry.

Bacterial cell membrane damage and pore formation induced by BZK and OB were examined by detection of propidium iodide (PI) influx (66). The bacteria were cultured at 37°C to mid-log phase and then diluted to an OD_600_ of 0.1 in PBS. BZK and OB were added to a 500-µl bacterial suspension at concentrations of 0× to 2× MIC and incubated for 30 min. Bacteria were collected and resuspended in buffer. PI solution was added to reach a final concentration of 2 µg/ml. The fluorescence signal in treated cells was determined by flow cytometry (BD Accuri) and further analyzed with FlowJo (Treestar, USA).

### *In vitro* translation assay.

*In vitro* translation was performed with an NEB PURExpress *in vitro* protein synthesis kit per the protocol described by the manufacturer. S35-methionine was used to measure production of the model protein DHFR.

### Data availability.

Tn-seq and RNA-seq data have been deposited with the NCBI’s Gene Expression Omnibus under GenBank accession number GSE96913. Whole-genome sequencing data were deposited with NCBI’s Sequence Read Archive under accession numbers SRR5343897 to SRR5343908.
