# Supplementary material for: Exploring the Antimicrobial Action of Quaternary Amines against Acinetobacter baumannii
Source: mBio. 2018 Feb 6;9(1):e02394-17. doi: 10.1128/mBio.02394-17 (PMC5801471; doi:10.1128/mBio.02394-17)
Supplement: TABLE S1 [file mbo001183722st1.pdf]

Table S1. *A. baumannii* benzalkonium chloride (BZK) minimal inhibitory concentrations (MICs) determined by agar and liquid microdilution methods.

Strain	Isolation Date	Source	Antibiotic Resistance	MIC agar dilution (μg/mL)[d]	MIC liquid microdilution (μg/mL)[d]
Clinical 1	2010	Blood Culture	Susceptible	16	8
Clinical 2	2012	Broncho-alveolar lavage	Susceptible	16	8
Clinical 3	2012	Blood Culture	Susceptible	16	8
Clinical 4	2013	Blood Culture	MDR[a]	16	8
Clinical 5	2013	Skin	Susceptible	16	8
AYE	2003	Blood Culture	MDR[a]	16	8
19606	1948	Urine	Gm[b]	≥64	16
17978	1951	Fatal Meningitis	Cm[c]	16	8
5075	2008	Osteomyelitis	MDR	16	8

[a] multi-drug resistant
[b] resistance to gentamycin
[c] resistance to chloramphenicol
[d] MIC represents the value determined from at least 3 biological replicates.
