# Supplementary material for: Exploring the Antimicrobial Action of Quaternary Amines against Acinetobacter baumannii
Source: mBio. 2018 Feb 6;9(1):e02394-17. doi: 10.1128/mBio.02394-17 (PMC5801471; doi:10.1128/mBio.02394-17)
Supplement: TABLE S2 [file mbo001183722st2.pdf]

Table S2. Results from Tn-seq analysis to identify genes that impact *A. baumannii* fitness in the presence of BZK. Genes showing -/+2-fold decrease or greater effect (p <0.01) are shown. Color coded genes are also shown in Figure 1.

Feature ID	Baggerley's test: BZK vs control normalized values - Weighted proportions fold change	Baggerley's test: BZK vs control normalized values - FDR p-value correction	Annotation	Categorization
A1S_0430	-23.13295159	1.00403E-21	rfaG -LPS glucosyltransferase - putative glycosyltransferase	**Cell Envelope**
A1S_0431	-22.33872483	9.06856E-13	lpxL - lipid A biosynthesis lauroyl acyltransferase; K025	
A1S_2902	-21.27164945	4.9467E-84	hypothetical protein - likely part of A1S_2901	
A1S_0065	-21.27134222	7.2849E-131	putative UDP-glucose 4-epimerase;galE	
A1S_2901	-18.59609531	5.4057E-27	putative polysaccharide deacetylase - predicted polysaccharide deacetylase lipoprotein - yadE	
A1S_3102	-17.56781406	2.21815E-12	toluene tolerance efflux transporter; mlaE	
A1S_0061	-15.09757154	4.95537E-36	putative UDP-galactose phosphate transferase - wcaJ	
A1S_3297	-13.14163085	2.20104E-08	putative outer membrane protein	
A1S_2899	-11.90170449	1.9744E-32	putative glycosyltransferase - waaB galactosyltransferase	
A1S_0060	-11.46627658	2.52139E-11	hypothetical protein - UDP-glucuronate:LPS(HepIII) glycosyltransferase- waaH	
A1S_0380	-10.53162104	4.38746E-52	glutamate racemase; murI - L-glutamate to D-glutamate for peptidoglycan	
A1S_0237	-10.36559791	0.000419878	D-alanyl-D-alanine endopeptidase penicillin-binding 7 - pbpG	
A1S_3100	-9.886894892	6.35676E-09	putative toluene tolerance protein (Ttg2D); mlaC	
A1S_2900	-8.031023112	7.15361E-15	putative lipopolysaccharide core biosynthesis - pgaC	
A1S_3103	-7.632592727	2.02319E-11	toluene tolerance efflux transporter; mlaF	
A1S_0066	-5.535821918	6.63941E-12	hypothetical protein; K01840 phosphomannomutase cpsG	
A1S_3196	-3.507631336	2.90098E-56	putative penicillin binding protein (PonA)	
A1S_3099	-2.811465922	2.39115E-08	putative toluene-tolerance protein (Ttg2E)	
A1S_3197	-2.184150028	0.009194616	putative penicillin binding protein (PonA)	
A1S_1751	-26.49387615	1.22297E-15	AdeA membrane fusion protein	**Efflux Pumps**
A1S_1753	-9.355528867	2.71487E-12	two-component system - AdeR	
A1S_1750	-8.624604681	3.56006E-14	AdeB; K03296 hydrophobic/amphiphilic exporter-1	
A1S_1754	-5.851169325	1.03485E-08	two-component system - AdeS	
A1S_0255	-3.16399952	1.04555E-53	RND efflux transporter; K12340 outer membrane channel - tolC	
A1S_2737	-2.649812775	0.000550895	AdeK	
A1S_0537	-2.61624967	1.17932E-08	RND efflux transporter - macA	
A1S_0535	-2.290114984	2.46257E-05	RND efflux transporter outer membrane factor	
A1S_0037	-14.74760372	4.03215E-08	alkali-inducible disulfide interchange protein; dsbA	**Proteostasis**
A1S_0528	-13.76542375	2.19981E-06	preprotein translocase subunit SecB	
A1S_0841	-13.56250704	7.6633E-77	ribosomal large subunit pseudouridine synthase D; rluD	
A1S_0476	-11.5332038	8.7781E-06	ATP-dependent Clp protease proteolytic subunit; clpP	
A1S_0554	-10.17961372	3.64464E-05	prfC; peptide chain release factor 3; K02837 pepti	
A1S_0570	-8.982101002	1.08706E-24	hypothetical protein; rsfS	
A1S_2324	-7.126360779	1.6036E-07	methionine aminopeptidase; map	
A1S_1031	-4.456670263	9.32222E-22	DNA-binding ATP-dependent protease La - lon	
A1S_1039	-3.755270329	8.98296E-07	aminopeptidase P; K01262 Xaa-Pro aminopeptidase - pepP	
A1S_0531	-3.050406438	0.003423366	putative GTPase; K06949 ribosome biogenesis GTPase- rsgA	
A1S_1030	-2.880387964	4.53683E-09	DNA-binding ATP-dependent protease La - lon	
A1S_0338	-2.877975086	0.000122324	ribosome-binding factor A; rbfA	
A1S_1910	-2.757161911	1.00466E-09	ATP-binding protease component; ClpA	
A1S_0279	-2.684165033	0.00015031	elongation factor Tu (EC:3.6.5.3); tufA	
A1S_1550	-2.552188716	1.14437E-12	gidB; 16S rRNA methyltransferase GidB;	
A1S_0992	-19.19509236	1.52505E-24	LysR family transcriptional regulator; oxyR	**Oxidative Stress Response**
A1S_0529	-13.45777192	4.49532E-07	glutaredoxin; K03676 glutaredoxin 3- grxC	
A1S_0780	-6.469156028	6.81816E-64	putative ATP-binding protein; mrp	
A1S_0243	-6.342530134	1.00789E-26	putative ferrous iron transport protein B; feoB	
A1S_0242	-6.031329994	0.000668827	putative ferrous iron transport protein A; feoA	
A1S_2206	-2.971258276	7.60709E-21	paraquat-inducible protein A; pqiA	
A1S_0455	-2.550915126	8.81406E-05	peptide methionine sulfoxide reductase; msrA	
A1S_1192	-19.51261996	5.99313E-18	aspartate carbamoyltransferase non-catalytic chain	**All other genes**
A1S_0144	-18.22285366	1.21074E-26	high affinity Zn transport protein; K09817 zinc tr	

Table S2. Results from Tn-seq analysis to identify genes that impact *A. baumannii* fitness in the presence of BZK. Genes showing -/+2-fold decrease or greater effect (p <0.01) are shown. Color coded genes are also shown in Figure 1.

Feature ID	Baggerley's test: BZK vs control normalized values - Weighted proportions fold change	Baggerley's test: BZK vs control normalized values - FDR p-value correction	Annotation	Categorization
A1S_0145	-14.8156525	0.000138724	Zn transport system transcriptional repressor; K09	
A1S_0749	-13.58315085	2.37073E-43	BfmS; K07639 two-component system	
A1S_2898	-13.14882681	2.25206E-42	hypothetical protein - sugar hydrolase in Acinetobacter	
A1S_0249	-11.65378001	7.13192E-29	cyclic 3'5'-adenosine monophosphate phosphodiester	
A1S_0268	-10.52575352	2.53275E-12	putative DNA binding protein; K03746 DNA-binding p	
A1S_0143	-10.5149909	6.06006E-10	high affinity Zn transport protein; K09816 zinc tr	
A1S_3134	-10.39976548	5.42499E-09	glutamate dehydrogenase (NAD(P)+) oxidoreductase p	
A1S_2261	-10.11374635	0.000990346	putative cold shock protein - cspA - stress protein transcription antiterminator	
A1S_0308	-9.282123992	2.23795E-23	beta-hydroxylase; K12979 beta-hydroxylase [EC:1.14	
A1S_0999	-9.265624283	1.19679E-10	putative signal peptide	
A1S_0784	-8.55367724	7.71453E-14	dcd; deoxycytidine triphosphate deaminase (EC:3.5.	
A1S_0064	-8.25288933	2.91158E-59	pgi; glucose-6-phosphate isomerase	
A1S_3034	-8.013319291	2.76264E-21	hypothetical protein	
A1S_2543	-7.46510279	7.94351E-16	hypothetical protein	
A1S_0238	-7.306188996	5.81389E-13	threonine synthase; K01733 threonine synthase [EC:	
A1S_0239	-6.956492496	4.26664E-11	homoserine dehydrogenase (EC:1.1.1.3); K00003 homo	
A1S_0323	-6.658636674	9.68777E-35	hypothetical protein; K09913 hypothetical protein	
A1S_0250	-6.121590348	7.72182E-39	adenosine diphosphate sugar pyrophosphatase; K0151	
A1S_0588	-6.029653751	2.01775E-22	hypothetical protein; rapZ -controlling the processing and stability of the small RNA regulator GlmZ	
A1S_0025	-5.792284289	0.000421558	putative transcriptional repressor; RutR - pyrimidine metabolism regulator	
A1S_1624	-5.438683466	0.009206067	hypothetical protein	
A1S_0610	-5.372832212	8.8216E-22	pyrroline-5-carboxylate reductase; K00286 pyrrolin	
A1S_3026	-5.260129975	4.4109E-91	hypothetical protein	
A1S_0316	-5.204752234	2.06662E-45	putative transcriptional regulator	
A1S_2989	-5.136005798	1.17074E-25	putative phospholipase D protein	
A1S_0903	-5.113783481	1.41518E-21	hypothetical protein	
A1S_2129	-4.977677677	1.82892E-09	hypothetical protein	
A1S_3152	-4.837716063	2.33253E-39	D-3-phosphoglycerate dehydrogenase; K00058 D-3-pho	
A1S_3105	-4.733166228	1.43228E-32	inositol-1-monophosphatase; K01092 myo-inositol-1(	
A1S_2048	-4.710226885	4.92111E-19	hypothetical protein	
A1S_3402	-4.657472823	0.000859833	arginase/agmatinase/formimionoglutamate hydrolase;	
A1S_0504	-4.472137985	6.41263E-07	hypothetical protein	
A1S_2118	-4.461674176	1.43853E-05	hypothetical protein	
A1S_0244	-4.458405491	3.40129E-06	hypothetical protein	
A1S_0424	-4.359526796	1.26891E-14	putative L-asparaginase I (AnsA); K01424 L-asparag	
A1S_0953	-4.294201073	4.22516E-09	hypothetical protein	
A1S_1191	-4.261787531	1.10413E-07	aspartate carbamoyltransferase non-catalytic chain	
A1S_2427	-4.192045551	2.34804E-08	putative transporter; K08218 MFS transporter	
A1S_1912	-4.152350875	2.70835E-10	hypothetical protein	
A1S_2686	-4.150028398	3.1589E-11	carbamoyl phosphate synthase small subunit (EC:6.3	
A1S_1034	-4.145947768	1.17932E-08	putative ligase; K01934 5-formyltetrahydrofolate c	
A1S_2839	-4.018765854	3.03884E-10	hypothetical protein; K14415 tRNA-splicing ligase	
A1S_0767	-3.950896176	8.57949E-20	Excalibur	
A1S_1862	-3.862755392	2.65466E-07	hypothetical protein	
A1S_0701	-3.842529908	4.36382E-13	hypothetical protein	
A1S_0873	-3.793946299	0.000153574	arginyl-tRNA-protein transferase (EC:2.3.2.8); K00	
A1S_2891	-3.789056814	1.16136E-43	phospholipase D endonuclease domain-containing pro	
A1S_1959	-3.771290696	4.98745E-11	acetyltransferase	
A1S_3156	-3.692667636	3.94365E-25	hypothetical protein; K07090	
A1S_1401	-3.68207231	2.55665E-25	putative transcriptional regulator	
A1S_3227	-3.681088949	1.09389E-07	putative RNA binding protein; K06959 uncharacteriz	

Table S2. Results from Tn-seq analysis to identify genes that impact *A. baumannii* fitness in the presence of BZK. Genes showing -/+2-fold decrease or greater effect (p <0.01) are shown. Color coded genes are also shown in Figure 1.

Feature ID	Baggerley's test: BZK vs control normalized values - Weighted proportions fold change	Baggerley's test: BZK vs control normalized values - FDR p-value correction	Annotation	Categorization
A1S_3050	-3.675160028	8.41236E-28	hypothetical protein	
A1S_3002	-3.569996605	8.33305E-16	stringent starvation protein A; K03599 RNA polymer	
A1S_2642	-3.468311717	0.000114321	regulatory protein TetR	
A1S_3228	-3.468293226	3.07586E-31	putative RNA binding protein	
A1S_2203	-3.461524895	3.25545E-16	hypothetical protein; K09857 hypothetical protein	
A1S_1160	-3.452638965	3.70263E-05	hypothetical protein	
A1S_0468	-3.341297693	4.93319E-10	hypothetical protein; K02428 dITP/XTP pyrophosphat	
A1S_0736	-3.327570374	3.48002E-09	hypothetical protein	
A1S_3450	-3.317699131	2.74554E-95	uracil transport protein	
A1S_0505	-3.289299752	2.87626E-27	hypothetical protein	
A1S_0339	-3.283947662	5.75064E-09	putative monovalent cation/H+ antiporter subunit G	
A1S_0564	-3.274625705	2.46008E-55	hypothetical protein	
A1S_0445	-3.235843271	1.01519E-06	hypothetical protein	
A1S_0256	-3.210539053	0.0038264	high affinity phosphate uptake transcriptional rep	
A1S_2974	-3.178559199	1.41302E-09	hypoxanthine phosphoribosyltransferase; K00760 hyp	
A1S_2846	-3.13423438	3.66122E-13	CysI-like sulfite reductase protein; K00381 sulfit	
A1S_3157	-3.07188014	8.81657E-62	hypothetical protein; K07090	
A1S_2697	-3.066653932	2.21088E-14	multifunctional protein; K02302 uroporphyrin-III C	
A1S_0169	-3.029270922	0.000820888	hypothetical protein	
A1S_2445	-3.007170952	2.99207E-07	phosphate transporter ATP-binding protein; K02036	
A1S_3045	-2.955918012	4.45225E-07	exoribonuclease R; K12573 ribonuclease R [EC:3.1.-	
A1S_0562	-2.933994701	4.6204E-06	tRNA-dihydrouridine synthase A; K05539 tRNA-dihydr	
A1S_0768	-2.878722939	0.000123804	LysR family transcriptional regulator	
A1S_3268	-2.847749103	0.000513229	hypothetical protein	
A1S_2841	-2.833909205	1.20939E-09	putative type 4 fimbrial biogenesis protein FimT;	
A1S_0399	-2.825789069	6.48762E-11	LysR family transcriptional regulator	
A1S_1338	-2.818957285	1.27436E-06	hypothetical protein; K02611 ring-1	
A1S_1038	-2.816710708	1.56037E-28	hypothetical protein; K09895 hypothetical protein	
A1S_1003	-2.804870433	3.87107E-43	hypothetical protein	
A1S_1114	-2.734470425	0.002334692	ferulate or vanillate catabolism transcriptional r	
A1S_2861	-2.733796482	8.51049E-16	putative signal peptide	
A1S_1145	-2.728111678	5.43472E-12	putative Cro protein	
A1S_1525	-2.71079193	0.002327133	hypothetical protein	
A1S_1000	-2.700332805	0.001310645	sulfate adenylyltransferase subunit 2 (EC:2.7.7.4)	
A1S_1461	-2.697683377	5.36074E-17	hypothetical protein	
A1S_2073	-2.68906871	3.58899E-17	ABC transporter ATPase	
A1S_0667	-2.688113434	0.001189191	hypothetical protein	
A1S_3372	-2.675095178	5.63053E-10	putative short-chain dehydrogenase	
A1S_2276	-2.671047648	0.000168528	bifunctional cyclohexadienyl dehydrogenase/3-phosp	
A1S_0489	-2.639788967	5.08747E-14	proA; gamma-glutamyl phosphate reductase (EC:1.2.1	
A1S_0590	-2.637169724	1.45849E-09	hypothetical protein; K09889 ribosome-associated protein	
A1S_1388	-2.629000715	0.001853951	hypothetical protein	
A1S_3245	-2.619027987	5.85781E-15	imidazole glycerol phosphate synthase cyclase subu	
A1S_2818	-2.615501509	3.96097E-22	nodulation protein precursor; K03296 hydrophobic/a	
A1S_3472	-2.602307396	5.60322E-17	DNA replication protein	
A1S_0713	-2.593317095	4.27533E-11	quinolinate synthetase; K03517 quinolinate synthas	
A1S_3250	-2.59327045	7.92465E-11	hypothetical protein	
A1S_0972	-2.582460964	5.58156E-05	phosphate transporter; K03306 inorganic phosphate	
A1S_1245	-2.55564546	1.11997E-06	putative acyltransferase	
A1S_1410	-2.527635268	8.28225E-25	LysR family transcriptional regulator	
A1S_0253	-2.516002342	1.1461E-09	transcriptional regulator	

Table S2. Results from Tn-seq analysis to identify genes that impact *A. baumannii* fitness in the presence of BZK. Genes showing -/+2-fold decrease or greater effect (p <0.01) are shown. Color coded genes are also shown in Figure 1.

Feature ID	Baggerley's test: BZK vs control normalized values - Weighted proportions fold change	Baggerley's test: BZK vs control normalized values - FDR p-value correction	Annotation	Categorization
A1S_0881	-2.507548956	0.000515591	cell division inhibitor; K03610 septum site-determ	
A1S_0735	-2.495829484	9.01504E-13	LysR family transcriptional regulator	
A1S_0209	-2.490099758	9.55903E-62	transposase; K07497 putative transposase	
A1S_2046	-2.478854522	5.14657E-06	putative ferredoxin-dependent glutamate synthase	
A1S_1819	-2.473148616	5.66224E-06	Short-chain dehydrogenase/reductase SDR	
A1S_1129	-2.460881967	2.93666E-12	repressor protein	
A1S_0089	-2.451758234	8.85449E-15	dual specificity pseudouridine synthase; K06177 tR	
A1S_2074	-2.447782699	3.79856E-08	hypothetical protein	
A1S_2546	-2.443816452	3.0309E-09	secreted trypsin-like serine protease	
A1S_2082	-2.415015889	0.009498303	putative transcriptional regulator	
A1S_0548	-2.40252055	0.005236914	TetR family transcriptional regulator	
A1S_3038	-2.396194965	0.001150186	hypothetical protein	
A1S_0599	-2.381407805	4.03813E-07	hypothetical protein	
A1S_1811	-2.374174569	3.32179E-05	hypothetical protein	
A1S_0413	-2.365773998	3.91239E-61	phosphoenolpyruvate-protein phosphotransferase; K0	
A1S_3461	-2.359639416	0	DNA replication protein	
A1S_0130	-2.350956076	0.000436962	guaA; GMP synthase (EC:6.3.5.2); K01951 GMP syntha	
A1S_0787	-2.316082686	9.27785E-06	putative signal peptide	
A1S_2588	-2.309552935	4.03813E-07	ruvB; Holliday junction DNA helicase RuvB (EC:3.1.	
A1S_0618	-2.29631714	1.81716E-13	hypothetical protein	
A1S_2709	-2.292105122	0.009451717	hypothetical protein; K06938	
A1S_0490	-2.291466353	0.00060809	putative hydrolase	
A1S_0533	-2.284703096	5.65986E-06	hypothetical protein	
A1S_3337	-2.284433988	0.000172671	glutathione synthetase (EC:6.3.2.3); K01920 glutat	
A1S_0076	-2.280909717	1.32984E-05	aconitate hydratase (EC:4.2.1.3); K01681 aconitate	
A1S_2778	-2.279191157	4.98745E-11	Maf-like protein; K06287 septum formation protein	
A1S_0007	-2.278164839	2.50458E-08	putative transport protein; K06158 ATP-binding cas	
A1S_2895	-2.25469562	0.002327133	hypothetical protein	
A1S_3315	-2.25081798	3.46191E-39	ArsR family transcriptional regulator; K03892 ArsR	
A1S_0388	-2.249420892	6.51007E-05	hypothetical protein	
A1S_0134	-2.229012227	1.83685E-19	pirin-related protein; K06911	
A1S_2760	-2.22112483	1.421E-08	geranyltranstransferase; K00795 farnesyl diphospha	
A1S_0379	-2.219261542	5.9235E-11	hypothetical protein; K09986 hypothetical protein	
A1S_3347	-2.217876974	5.56343E-27	thiol:disulfide interchange protein precursor	
A1S_3148	-2.208547103	0.007358088	NADPH specific quinone oxidoreductase; K03923 modu	
A1S_2919	-2.204894661	0.001720892	hypothetical protein; K08994 putative membrane pro	
A1S_2761	-2.195170333	1.94987E-06	hypothetical protein; K09788 hypothetical protein	
A1S_0738	-2.190233252	5.62156E-08	putative flavoprotein oxidoreductase; K09024 flavi	
A1S_0913	-2.189011825	3.09363E-14	hypothetical protein	
A1S_0936	-2.187214535	3.17487E-07	hypothetical protein	
A1S_0027	-2.180852013	1.99577E-14	alkanesulfonate transport protein; K15554 sulfonat	
A1S_0893	-2.176253911	0.001340249	hypothetical protein; K09801 hypothetical protein	
A1S_1602	-2.172544601	2.47785E-09	hypothetical protein; K00657 diamine N-acetyltrans	
A1S_1684	-2.170375266	4.062E-05	hypothetical protein; K09747 hypothetical protein	
A1S_1703	-2.144449755	0.000533192	dihydrolipoamide dehydrogenase	
A1S_0559	-2.135902515	6.85575E-10	putative NAD(P)-binding enzyme	
A1S_2462	-2.131275586	0.007636862	ATP-dependent helicase HepA; K03580 ATP-dependent	
A1S_0397	-2.129368628	3.35919E-09	putative oxidase; putative coproporphyrinogen III	
A1S_0571	-2.123616877	0.008785342	hydroxypyruvate isomerase; K01816 hydroxypyruvate	
A1S_2260	-2.116798344	6.46002E-08	ATP-dependent RNA helicase RhlB; K03732 ATP-depend	
A1S_0552	-2.111205717	1.66642E-06	hypothetical protein	

Table S2. Results from Tn-seq analysis to identify genes that impact *A. baumannii* fitness in the presence of BZK. Genes showing -/+2-fold decrease or greater effect (p <0.01) are shown. Color coded genes are also shown in Figure 1.

Feature ID	Baggerley's test: BZK vs control normalized values - Weighted proportions fold change	Baggerley's test: BZK vs control normalized values - FDR p-value correction	Annotation	Categorization
A1S_0778	-2.108338671	9.06289E-05	metG; methionyl-tRNA synthetase (EC:6.1.1.10); K01	
A1S_0727	-2.100307406	4.36382E-13	putative substrate-binding protein; K02020 molybda	
A1S_1297	-2.092157806	6.41028E-10	hypothetical protein; K11897 type VI secretion sys	
A1S_3476	-2.08865826	3.86037E-39	secretory lipase	
A1S_0911	-2.0811646	6.33898E-11	hypothetical protein	
A1S_0737	-2.079216764	1.30274E-11	5-methyltetrahydropteroyltriglutamate--homocystein	
A1S_2447	-2.068239074	6.03732E-05	EsvD; K02037 phosphate transport system permease p	
A1S_0433	-2.066836029	6.08278E-05	transport protein Uup; K15738 ATP-binding cassette	
A1S_2624	-2.064965414	7.70031E-16	putative CPS-53 prophage bactoprenol glucosyl tran	
A1S_1197	-2.061886667	6.29925E-12	putative extracellular nuclease; K07004	
A1S_0594	-2.056979017	1.81139E-22	putative glutathione S-transferase; K00799 glutath	
A1S_2865	-2.051854468	3.32122E-13	protein tyrosine/serine phosphatase	
A1S_1274	-2.0479685	2.46257E-05	alcohol dehydrogenase GroES-like protein	
A1S_0123	-2.04769944	1.88842E-10	hypothetical protein	
A1S_0942	-2.044621128	0.00284097	nucleoside/purine/pyrimidine transport protein Pnu	
A1S_3166	-2.039368807	0.000983004	pilin like competence factor; K02655 type IV pilus	
A1S_2859	-2.037265158	2.72834E-27	putative hemolysin III (HLY-III); K11068 hemolysin	
A1S_0800	-2.03551504	8.3902E-11	bacterioferritin; K03594 bacterioferritin	
A1S_0032	-2.024810738	0.002835769	putative signal peptide	
A1S_2487	-2.021626889	6.12255E-06	hypothetical protein	
A1S_2395	-2.012119283	9.23537E-08	hypothetical protein; K06911	
A1S_3158	-2.002415722	2.39016E-12	truB; tRNA pseudouridine synthase B; K03177 tRNA p	
A1S_0204	-2.001979426	4.02476E-23	class II aldolase/adducin domain protein; K01628 L	
A1S_0566	2.000159251	0.00072606	pyridine nucleotide transhydrogenase (proton pump)	
A1S_2247	2.001842996	0.000411589	putative signal peptide	
A1S_2275	2.002646673	8.45234E-11	hypothetical protein	
A1S_1449	2.00451727	4.67617E-11	transcriptional regulatory protein	
A1S_1935	2.007353797	2.00083E-05	hypothetical protein	
A1S_1718	2.008111858	0.009034517	putative oxidoreductase	
A1S_1507	2.008319248	0.004186364	fimbrial protein	
A1S_0776	2.01440873	0.001382267	TetR family transcriptional regulator	
A1S_1857	2.024826351	0.000820888	vanillate O-demethylase oxidoreductase	
A1S_1183	2.028745265	0.004946229	hypothetical protein; K07397 putative redox protei	
A1S_0114	2.031426586	3.95377E-07	Acyl carrier protein	
A1S_0437	2.032982273	0.000945379	hypothetical protein	
A1S_3221	2.037543212	0.000125157	putative ABC transporter ATP-binding protein	
A1S_1606	2.041203918	6.26591E-09	hypothetical protein	
A1S_1356	2.042520941	0.001159333	4-hydroxybenzoate 3-monooxygenase (EC:1.14.13.2);	
A1S_0810	2.043243033	0.000108125	putative ribosomal large subunit pseudouridine syn	
A1S_2452	2.04610617	0.000236019	NAD-dependent aldehyde dehydrogenases	
A1S_1484	2.049214958	2.10847E-07	D-methionine transport protein; K02073 D-methionin	
A1S_1757	2.051075376	6.66109E-10	Alpha/beta hydrolase; K06889	
A1S_3048	2.052315115	0.002627075	hypothetical protein; K07070	
A1S_3207	2.056872371	0	sulfate transport protein; K02048 sulfate transpor	
A1S_0674	2.059993691	0	putative transposase	
A1S_2621	2.061482497	0	hypothetical protein; K09686 antibiotic transport	
A1S_0006	2.062456986	7.86603E-08	putative DedA family protein	
A1S_1499	2.064746194	8.55177E-05	hypothetical protein	
A1S_3173	2.08343893	1.66777E-09	hypothetical protein; K07567 TdcF protein	
A1S_1626	2.086010455	0.002503124	putative adenylate or guanylate cyclase	
A1S_1308	2.092215164	1.38774E-11	hypothetical protein; K11902 type VI secretion sys	

Table S2. Results from Tn-seq analysis to identify genes that impact *A. baumannii* fitness in the presence of BZK. Genes showing -/+2-fold decrease or greater effect (p <0.01) are shown. Color coded genes are also shown in Figure 1.

Feature ID	Baggerley's test: BZK vs control normalized values - Weighted proportions fold change	Baggerley's test: BZK vs control normalized values - FDR p-value correction	Annotation	Categorization
A1S_1015	2.096997475	0.002568509	ureE; urease accessory protein UreE; K03187 urease	
A1S_1394	2.11251048	2.37925E-05	putative two-component response regulator protein	
A1S_2594	2.125891712	1.28348E-10	tolB; translocation protein TolB; K03641 TolB prot	
A1S_0991	2.134747387	0.000952543	hypothetical protein	
A1S_2119	2.137671663	0	putative acetyltransferase	
A1S_3028	2.143982791	3.76764E-09	putative tRNA-i(6)A37 modification enzyme	
A1S_3159	2.147868419	0	lipase chaperone	
A1S_3290	2.152487492	3.68828E-05	EsvF2; K03707 thiaminase (transcriptional activato	
A1S_0941	2.155450722	2.50098E-06	enoyl-CoA hydratase (EC:4.2.1.17)	
A1S_3328	2.156454842	3.75691E-14	aceE; pyruvate dehydrogenase subunit E1; K00163 py	
A1S_2107	2.161798322	0	glutamine amidotransferase (EC:6.3.5.2); K01951 GM	
A1S_1043	2.162157914	0.000443308	putative transcriptional regulator	
A1S_1512	2.164035309	5.30463E-09	putative ferredoxin	
A1S_2133	2.172198004	0.001500889	hypothetical protein	
A1S_0522	2.180715064	0	3-oxoacyl-(acyl carrier protein) synthase I (EC:2.	
A1S_1650	2.189367446	2.7695E-05	hypothetical protein	
A1S_0444	2.19936363	5.65986E-06	hypothetical protein	
A1S_1442	2.204795885	3.20418E-05	taurine ABC transporter periplasmic taurine-bindin	
A1S_1533	2.208824408	4.93319E-10	AraC family transcriptional regulator	
A1S_1561	2.209477012	8.81406E-05	putative transcriptional regulator	
A1S_2685	2.212085828	0.008538567	hypothetical protein	
A1S_0391	2.212417493	9.4417E-08	rpmE2; 50S ribosomal protein L31 type B; K02909 la	
A1S_0611	2.212701718	0.000837818	putative integral membrane resistance protein; K02	
A1S_3208	2.214510827	3.0923E-12	putative peptide signal	
A1S_2607	2.221587856	0.001871626	putative hydrolase	
A1S_1620	2.222244422	9.28158E-05	hypothetical protein	
A1S_0804	2.223330504	0.008768645	trehalose-6-phosphate phophatase; K01087 trehalose	
A1S_1812	2.229281438	2.98348E-13	hypothetical protein	
A1S_3317	2.23166959	1.1994E-14	putative outer membrane protein	
A1S_2763	2.23600496	0	aromatic amino acid APC transporter	
A1S_2733	2.248496634	0	hypothetical protein	
A1S_1992	2.250982266	0.003423366	DMT family permease	
A1S_2281	2.25291432	5.89471E-11	hypothetical protein	
A1S_0327	2.268612486	4.22387E-15	type 4 prepilin-like proteins leader peptide proce	
A1S_3174	2.272285722	4.65753E-10	putative regulatory or redox component complexing	
A1S_2309	2.283027649	3.54804E-05	putative DNA exonuclease X; K10857 exodeoxyribonuc	
A1S_2657	2.291940658	7.77176E-09	putative transglycosylase	
A1S_0703	2.294454487	2.80821E-05	putative esterase; K01070 S-formylglutathione hydr	
A1S_2560	2.29731645	0.000192432	hypothetical protein; K13408 membrane fusion prote	
A1S_0370	2.298786971	0.001121197	general secretion pathway protein G; K02456 genera	
A1S_1081	2.315244675	9.78631E-05	putative transcriptional regulator	
A1S_0170	2.315935469	1.95806E-13	putative outer membrane copper receptor (OprC); K0	
A1S_0806	2.321521625	0.001357423	adenosylmethionine-8-amino-7-oxononanoate aminotra	
A1S_2505	2.323682583	0	hypothetical protein	
A1S_1375	2.324309766	3.08156E-05	putative propionyl-CoA carboxylase (Beta subunit);	
A1S_0140	2.332767718	0	malate dehydrogenase; K00027 malate dehydrogenase	
A1S_0520	2.333483381	3.73912E-05	putative oxidoreductase protein; putative dehydrog	
A1S_0937	2.341405184	0	peptidyl-prolyl cis-trans isomerase; K01802 peptid	
A1S_1868	2.341605192	0.005866775	porin for benzoate transport (BenP)	
A1S_2159	2.342453711	3.09453E-07	hypothetical protein	
A1S_2407	2.343223388	0.007663197	hypothetical protein	

Table S2. Results from Tn-seq analysis to identify genes that impact *A. baumannii* fitness in the presence of BZK. Genes showing -/+2-fold decrease or greater effect (p <0.01) are shown. Color coded genes are also shown in Figure 1.

Feature ID	Baggerley's test: BZK vs control normalized values - Weighted proportions fold change	Baggerley's test: BZK vs control normalized values - FDR p-value correction	Annotation	Categorization
A1S_0499	2.345452361	1.14723E-05	putative Fe-S-cluster redox enzyme; K06941 23S rRN	
A1S_0779	2.346724836	1.6534E-13	hypothetical protein	
A1S_1849	2.354293866	0.005596459	beta-ketoadipyl CoA thiolase; K00680 [EC:2.3.1.-]	
A1S_0438	2.366583375	2.54409E-06	hypothetical protein	
A1S_1876	2.369093815	1.05823E-07	putative metallo-beta lactamase	
A1S_1208	2.378617672	0.000252446	3-oxoacyl-(acyl carrier protein) synthase III; K00	
A1S_1807	2.383911556	9.78631E-05	regulatory protein GntR HTH; K03710 GntR family tr	
A1S_1530	2.393816944	0.000219636	SSS family major sodium/proline symporter; K11928	
A1S_1474	2.406441887	0.000285223	uridylyltransferase; K00990 [protein-PII] uridylyl	
A1S_3113	2.415870648	3.0799E-05	hypothetical protein	
A1S_1419	2.418014181	0.000355959	anti-sigm factor ChrR	
A1S_2632	2.435053237	0	diaminopimelate decarboxylase; K01586 diaminopimel	
A1S_0514	2.452247589	0	hypothetical protein	
A1S_0512	2.465199661	0	hypothetical protein	
A1S_2228	2.46594258	0.000361924	hypothetical protein	
A1S_2039	2.478712085	0.000227209	hypothetical protein	
A1S_1425	2.507912242	0.001126247	malonate decarboxylase gamma subunit; K13933 malon	
A1S_2570	2.516711825	5.52745E-05	putative siderophore biosynthesis protein; putativ	
A1S_1776	2.518167511	1.57395E-14	transcriptional regulatory protein	
A1S_0270	2.524077498	4.31934E-05	putative general secretion pathway protein; K02452	
A1S_2320	2.537059751	4.07211E-09	AraC family transcriptional regulator	
A1S_0414	2.548130111	0	dinucleoside polyphosphate hydrolase; K08311 putat	
A1S_1672	2.550853402	2.68259E-07	hypothetical protein; K09686 antibiotic transport	
A1S_1413	2.557721193	0.000282883	regulatory protein	
A1S_2308	2.570438132	1.1994E-14	hypothetical protein	
A1S_2152	2.578783995	0.00054073	AraC family transcriptional regulator	
A1S_1722	2.596714146	0.000555095	putative ATP-binding component of ABC transporter;	
A1S_1817	2.600700289	1.90773E-07	Acyl-CoA dehydrogenase	
A1S_2618	2.612793374	3.3064E-05	RND efflux transporter	
A1S_1416	2.618640602	4.85956E-14	negative transcriptional regulator; K07734 transcr	
A1S_2806	2.631961308	6.17993E-10	hypothetical protein	
A1S_2444	2.663684328	0.001257466	putative periplasmic protease; K04774 serine prote	
A1S_0515	2.675044751	0	histidine ammonia-lyase protein; K01745 histidine	
A1S_2964	2.700022206	1.50828E-09	phosphoribosylaminoimidazole carboxylase mutase su	
A1S_2435	2.706601396	0	D-ala-D-ala-carboxypeptidase; penicillin-binding p	
A1S_2187	2.714757265	1.15815E-12	hypothetical protein; K00602 phosphoribosylaminoim	
A1S_1917	2.71637376	2.38829E-09	putative potassium uptake protein; K03499 trk syst	
A1S_2071	2.747651072	0.007705489	hypothetical protein	
A1S_0844	2.747751316	3.16292E-11	smpB; SsrA-binding protein; K03664 SsrA-binding pr	
A1S_2866	2.777879368	4.29168E-07	hypothetical protein	
A1S_2768	2.781894199	0	undecaprenyl pyrophosphate phosphatase (EC:3.6.1.2	
A1S_0976	2.783378529	0.000490372	hypothetical protein	
A1S_1310	2.787240899	0.007725678	hypothetical protein; K11892 type VI secretion sys	
A1S_1635	2.803033522	0	hypothetical protein	
A1S_1113	2.813295728	0.00020171	putative transcriptional regulator	
A1S_1919	2.819903537	0	putative phospholipase A1 precursor (PldA); K01058	
A1S_1943	2.820098492	0.004017934	hypothetical protein	
A1S_2511	2.841224325	1.30729E-05	phenylacetic acid degradation-related protein	
A1S_2267	2.845716823	0	hypothetical protein; K09861 hypothetical protein	
A1S_2643	2.856192511	0.008266605	short chain dehydrogenase/reductase family oxidore	
A1S_2151	2.857305465	0.001956839	AraC family transcriptional regulator	

Table S2. Results from Tn-seq analysis to identify genes that impact *A. baumannii* fitness in the presence of BZK. Genes showing -/+2-fold decrease or greater effect (p <0.01) are shown. Color coded genes are also shown in Figure 1.

Feature ID	Baggerley's test: BZK vs control normalized values - Weighted proportions fold change	Baggerley's test: BZK vs control normalized values - FDR p-value correction	Annotation	Categorization
A1S_0269	2.859215494	2.46857E-13	putative general secretion pathway protein; K02463	
A1S_1855	2.875345059	0.007139219	putative transcriptional regulator	
A1S_3358	2.877172281	1.1994E-14	ureidoglycolate hydrolase (EC:3.5.3.19); K01483 ur	
A1S_1022	2.920444471	0	hypothetical protein	
A1S_2622	2.944580332	0	hypothetical protein; K09686 antibiotic transport	
A1S_0479	2.948517859	5.14004E-05	putative signal peptide	
A1S_0619	2.969280866	0.001578501	putative carbon-nitrogen hydrolase	
A1S_1676	2.972445668	0.003353924	hypothetical protein	
A1S_3329	2.98027266	6.78506E-08	EsvJ	
A1S_0808	2.988504523	0.001582578	putative biotin biosynthesis protein (BioC); K0216	
A1S_1105	2.990326281	0	hypothetical protein	
A1S_1834	2.995180302	0.000612503	hypothetical protein	
A1S_3033	2.999722399	1.42713E-11	hypothetical protein	
A1S_1469	3.010420299	2.68378E-14	peptide methionine sulfoxide reductase; K07305 pep	
A1S_1443	3.039833978	0	taurine ATP-binding transport system component; K1	
A1S_1403	3.04580648	0	putative cysteine desulfurase 1 (Csd); K11717 cyst	
A1S_1193	3.047528324	8.92186E-06	OmpA/MotB	
A1S_1072	3.086434594	1.10413E-07	hypothetical protein	
A1S_2442	3.108263657	0.000791148	hypothetical protein; K08984 putative membrane pro	
A1S_2211	3.116967987	4.72383E-06	ADP-ribose pyrophosphatase	
A1S_2423	3.20297656	0.009492725	rpmE; 50S ribosomal protein L31; K02909 large subu	
A1S_1320	3.211734033	0.000128741	transcriptional regulator SoxR; K13639 MerR family	
A1S_0525	3.214529224	0	3-oxoacyl-(acyl carrier protein) synthase II (EC:2	
A1S_1420	3.216874453	0.006436669	regulatory protein LysR:LysR	
A1S_2342	3.222661401	0.00539951	hypothetical protein	
A1S_1432	3.236418039	4.22387E-15	hypothetical protein	
A1S_3176	3.276908842	0	hypothetical protein	
A1S_2416	3.285266851	0	hypothetical protein	
A1S_0607	3.29760025	1.62035E-07	exopolyphosphatase; K01524 exopolyphosphatase / gu	
A1S_1735	3.309884992	0.000100387	hypothetical protein	
A1S_3348	3.350972656	5.43915E-09	putative signal peptide	
A1S_0670	3.352253916	0	protein tyrosine phosphatase	
A1S_1348	3.381146846	0	carbonic anhydrase; K02617 phenylacetic acid degra	
A1S_2378	3.388489198	0	putative ABC transporter	
A1S_2809	3.422404172	0.000740605	bacteriolytic lipoprotein entericidin B	
A1S_1491	3.432278953	0.000236019	glutamate/aspartate transport protein; K10003 glut	
A1S_1698	3.436213159	0.005655956	lipoyl synthase	
A1S_2692	3.455130066	9.64522E-05	putative universal stress protein A (UspA)	
A1S_1025	3.476708253	0	putative iron-sulfur protein; K03616 electron tran	
A1S_1612	3.487539439	0	ABC-type dipeptide/oligopeptide/nickel transport s	
A1S_1490	3.524962121	0.002085696	glutamate/aspartate transport protein; K10001 glut	
A1S_1603	3.547298923	1.11706E-05	biopolymer transport protein ExbD/TolR; K03559 bio	
A1S_1686	3.596370295	7.31865E-09	ribonuclease D; K03684 ribonuclease D [EC:3.1.13.5	
A1S_3448	3.615734669	0.005454633	putative transcriptional regulator	
A1S_2881	3.655737082	0	putative fatty acid desaturase; K00507 stearoyl-Co	
A1S_1389	3.655759436	1.6471E-05	DNA polymerase V component; K03503 DNA polymerase	
A1S_1737	3.66228127	0	3-hydroxybutyrate dehydrogenase (EC:1.1.1.30); K00	
A1S_2254	3.664064358	0.007947516	hypothetical protein; K02462 general secretion pat	
A1S_3029	3.684863012	0	putative tRNA-i(6)A37 modification enzyme; K06168	
A1S_2997	3.703059365	0	apaH; diadenosine tetraphosphatase (EC:3.6.1.41);	
A1S_2341	3.720655151	3.44502E-05	HtrA-like serine protease	

Table S2. Results from Tn-seq analysis to identify genes that impact *A. baumannii* fitness in the presence of BZK. Genes showing -/+2-fold decrease or greater effect (p <0.01) are shown. Color coded genes are also shown in Figure 1.

Feature ID	Baggerley's test: BZK vs control normalized values - Weighted proportions fold change	Baggerley's test: BZK vs control normalized values - FDR p-value correction	Annotation	Categorization
A1S_0523	3.722588119	0	putative 3-hydroxylacyl-(acyl carrier protein) deh	
A1S_2083	3.72634259	0.006170043	AsnC family transcriptional regulator; K03719 Lrp/	
A1S_1319	3.761510361	0.001462511	hypothetical protein; K09765 hypothetical protein	
A1S_1605	3.800361191	7.83733E-05	biopolymer transport proteins; K03561 biopolymer t	
A1S_0507	3.828001642	0	hypothetical protein	
A1S_1188	3.847175571	0	putative N-6 adenine-specific DNA methylase; K1229	
A1S_0217	3.871927677	0	hypothetical protein; K09806 hypothetical protein	
A1S_1020	3.882419172	0	penicillin-binding protein 2; K05515 penicillin-bi	
A1S_2744	3.917358153	0.000209746	SAM-dependent methyltransferase	
A1S_1341	3.917441176	1.3977E-08	enoyl-CoA hydratase/carnithine racemase	
A1S_1248	3.926924077	0.006102088	hypothetical protein	
A1S_0519	3.978854424	0	hypothetical protein	
A1S_2047	3.992773997	2.11636E-05	putative lysophospholipase	
A1S_2086	4.008765596	0.009492725	putative short chain dehydrogenase	
A1S_0556	4.011618777	0	hypothetical protein	
A1S_3037	4.021364067	0	putative ribonuclease (Rbn); K07058 membrane prote	
A1S_2188	4.050419085	0.001228245	hypothetical protein	
A1S_1906	4.061748532	0.000169116	hypothetical protein	
A1S_2122	4.07548822	1.59839E-08	transcriptional regulator; K03576 LysR family tran	
A1S_1619	4.099287983	0.000457142	ribonuclease activity regulator protein RraA; K025	
A1S_1549	4.111702894	0.000475866	putative nucleotidyl transferase	
A1S_0297	4.153738874	0	hypothetical protein	
A1S_1322	4.164563163	2.13395E-05	dihydrodipicolinate synthetase; K01714 dihydrodipi	
A1S_0659	4.164678725	0	hypothetical protein	
A1S_1797	4.194549113	1.55879E-06	aldehyde dehydrogenase	
A1S_0884	4.262578608	0	putative outer membrane protein	
A1S_0524	4.299054576	5.41856E-13	hypothetical protein; K00059 3-oxoacyl-[acyl-carri	
A1S_0574	4.306415133	6.43763E-06	GacS-like sensor kinase protein; K07678 two-compon	
A1S_2099	4.34701444	0	hypothetical protein	
A1S_1281	4.371600633	0	TPR domain-containing protein	
A1S_1165	4.527978901	4.83781E-08	putative phage tail tape meausure protein	
A1S_1383	4.53302184	4.7411E-06	surface antigen	
A1S_2540	4.53797753	0	putative organic radical activating enzyme; K10026	
A1S_1651	4.540778495	0.005171101	hypothetical protein	
A1S_1554	4.548187689	0.001145879	putative biopolymer transport protein ExbD/TolR; K	
A1S_1659	4.703376077	2.42208E-05	cobalbumin biosynthesis enzyme; K02231 adenosylcob	
A1S_0509	4.845853681	0	putative acyl carrier protein; K02078 acyl carrier	
A1S_0518	4.968243608	1.17602E-11	hypothetical protein	
A1S_0521	5.029948594	4.86776E-05	hypothetical protein	
A1S_2016	5.073685843	0	Phage-related lysozyme	
A1S_0902	5.089128629	0.005466996	lactoylglutathione lyase-related protein	
A1S_1867	5.116546264	0.004400125	major facilitator transporter	
A1S_0887	5.173642926	0	phosphomannomutase; K15778 phosphomannomutase / ph	
A1S_1353	5.217028681	1.41255E-12	LysR family transcriptional regulator	
A1S_1518	5.253574039	8.23229E-06	putative suppressor of F exclusion of phage T7; K0	
A1S_0742	5.25545159	0	iron-regulated protein	
A1S_3276	5.276343323	3.86202E-08	hypothetical protein	
A1S_1509	5.341099054	0.000770562	pili assembly chaperone	
A1S_1562	5.486180599	7.33124E-09	putative general secretion pathway protein G precu	
A1S_2377	5.61398961	0.004017934	putative ABC transporter	
A1S_2246	5.683353037	0.001631167	hypothetical protein; K09117 hypothetical protein	

Table S2. Results from Tn-seq analysis to identify genes that impact *A. baumannii* fitness in the presence of BZK. Genes showing -/+2-fold decrease or greater effect (p <0.01) are shown. Color coded genes are also shown in Figure 1.

Feature ID	Baggerley's test: BZK vs control normalized values - Weighted proportions fold change	Baggerley's test: BZK vs control normalized values - FDR p-value correction	Annotation	Categorization
A1S_1379	5.787048066	2.84645E-10	putative SAM-dependent methyltransferase	
A1S_1312	5.915257929	4.22387E-15	hypothetical protein	
A1S_0236	5.993400872	0	response regulator - Ec UvrY - DNA repair carbon metabolism	
A1S_1889	6.119327409	0.000774042	3-oxoadipate enol-lactonase I; K01055 3-oxoadipate	
A1S_1374	6.142159537	9.47627E-06	3-methylglutaconyl-CoA hydratase	
A1S_2303	6.148282894	0	LysR family transcriptional regulator	
A1S_0511	6.158677036	0.000191765	hypothetical protein	
A1S_2360	6.16990982	7.44016E-05	trpC; indole-3-glycerol-phosphate synthase; K01609	
A1S_0510	6.378320121	8.26104E-15	acyl carrier protein; K02078 acyl carrier protein	
A1S_1979	7.228950882	0.000786883	putative transcriptional regulator	
A1S_1044	7.306797311	0.004506413	Co/Zn/Cd efflux system	
A1S_1983	7.408116714	0	putative signal peptide	
A1S_2375	7.442830746	4.24637E-05	putative ABC transporter	
A1S_0513	8.027756614	0	putative glycosyl transferase	
A1S_1818	8.563758058	8.84645E-10	MaoC-like dehydratase	
A1S_0258	8.978569708	0.003566982	argininosuccinate lyase	
A1S_1337	12.37341951	3.75691E-14	paaB; phenylacetate-CoA oxygenase subunit PaaB; K0	
A1S_1963	12.90082342	6.81367E-07	regulatory protein; K03565 regulatory protein	
A1S_1360	15.23433193	3.15138E-12	ABC transporter	
A1S_2153	16.54079501	0	methyl transferase; K02493 release factor glutamin	
A1S_0835	21.00143492	0.006596464	outer-membrane lipoprotein precursor; lolB outer membrane	
A1S_2599	301.7104545	8.27796E-05	hypothetical protein	
