# Supplementary material for: Exploring the Antimicrobial Action of Quaternary Amines against Acinetobacter baumannii
Source: mBio. 2018 Feb 6;9(1):e02394-17. doi: 10.1128/mBio.02394-17 (PMC5801471; doi:10.1128/mBio.02394-17)
Supplement: TABLE S3 [file mbo001183722st3.pdf]

Table S3. Table S3. Results from RNA-seq analysis to identify genes that change expression in *A. baumannii* in the presence of BZK. Genes showing greater than a -/+ 2-fold effect (p <0.01) are shown. Color coded genes are also shown in Figure 1.

Feature ID	Baggerley's test: BZK vs LB normalized values - Weighted proportions fold change	Baggerley's test: BZK vs LB normalized values - FDR p-value correction	Annotation	Categorization
A1S_2538	2.902340356	0	outer membrane protein - carO	**Cell Envelope**
A1S_2489	2.415473258	5.31082E-06	UDP-N-acetyl glucosamine-2-epimerase; wecB	
A1S_2063	209.9552605	0	hypothetical protein- aceI	**Efflux Pumps**
A1S_1752	81.05035901	0	AdeA membrane fusion protein	
A1S_1751	44.03790036	2.01123E-13	AdeA membrane fusion protein	
A1S_1750	19.37102562	3.26787E-10	AdeB; K03296 hydrophobic/amphiphilic exporter	
A1S_2618	4.288555766	0	RND efflux transporter	
A1S_3447	2.699162322	4.36106E-08	RND efflux transporter - macA	
A1S_1773	2.54117605	0	RND family drug transporter; ermA	
A1S_1186	16.23295399	0	ATP-dependent protease Hsp 100; clpB	**Proteostasis**
A1S_1988	15.35549498	0	putative intracellular sulfur oxidation protein - tusD	
A1S_1030	5.912190316	0	DNA-binding ATP-dependent protease La; - lon	
A1S_1031	4.426033436	0	DNA-binding ATP-dependent protease La- lon	
A1S_2444	4.007452968	0	putative periplasmic protease;	
A1S_1629	3.48042488	0	co-chaperone protein (Hsc20); hscB	
A1S_2658	3.278993143	0	heat shock protein HtpX	
A1S_2729	2.96635706	0	outer-membrane lipoproteins carrier protein; lolA	
A1S_2092	2.947156739	0	pepN; aminopeptidase N; K01256 aminopeptidase N	
A1S_0477	2.832001916	0	clpX; ATP-dependent protease ATP-binding	
A1S_2960	2.792991165	0	dnaK; molecular chaperone DnaK;	
A1S_2959	2.712809331	0	Hsp 24 nucleotide exchange factor; grpE	
A1S_0366	2.65026846	0	heat shock protein Hsp33; hslO	
A1S_0570	2.333203681	0	hypothetical protein; rsfS	
A1S_1628	2.260336362	0	hscA; chaperone protein HscA	
A1S_1201	83.44801582	6.71651E-10	alkyl hydroperoxide reductase subunit F - ahpF	**Oxidative Stress Response**
A1S_1458	59.35285413	2.21987E-08	alkyl hydroperoxide reductase subunit F- ahpF	
A1S_1200	34.22271764	0	alkyl hydroperoxide reductase subunit F; ahpF	
A1S_1460	22.39909418	1.65984E-13	alkyl hydroperoxide reductase subunit F - ahpF	
A1S_1634	7.585021565	0	iscRSUA operon repressor; iscR	
A1S_0412	6.728965126	6.04141E-15	catalase; katG	
A1S_1630	6.398600414	0	hypothetical protein; K13628 iron-sulfur cluster - iscA	
A1S_1633	5.62644747	0	cysteine desulfurase; K04487 cysteine desulfurase - iscS	
A1S_1386	5.019176849	5.4217E-14	catalase; K03781 catalase katE	
A1S_3382	4.948118867	1.31024E-06	catalase; K03781 catalase katE2	
A1S_0242	4.098768094	2.22615E-10	putative ferrous iron transport protein A; feoA	
A1S_1632	3.61230792	0	cysteine desulfurase - iscS	
A1S_0243	2.861683675	0	putative ferrous iron transport protein B; feoB	
A1S_1205	2.405076207	0.001990771	alkyl hydroperoxide reductase C22 subunit; ahpC	
A1S_1823	27.91649076	0	TetR family transcriptional regulator: rutD pyrimidine metabolism	**All other genes**
A1S_3113	25.7446868	0	hypothetical protein: pspC TF or toxin of pspC_B pair	
A1S_2969	18.98082685	0	hypothetical protein; Alx - predicted membrane-bound redox modulator	
A1S_2504	18.52362055	0	excinuclease ABC subunit B; K03702 excinuclease AB	
A1S_3294	18.49966519	0	TetR/AcrR family transcriptional regulator	
A1S_0408	18.11366418	0	putative glutathione S-transferase; K00799 glutath	
A1S_2705	17.13683733	0	hypothetical protein; K09158 hypothetical protein	

Feature ID	Baggerley's test: BZK vs LB normalized values - Weighted proportions fold change	Baggerley's test: BZK vs LB normalized values - FDR p-value correction	Annotation	Categorization
A1S_0445	14.76385939	0	hypothetical protein	
A1S_1383	13.87348098	0	surface antigen	
A1S_3293	13.83093649	0	putative NADPH:quinone reductase and related Zn-de	
A1S_1987	13.75567059	0	putative UDP-galactose 4-epimerase (GalE-like); K0	
A1S_1824	13.68526419	0	hypothetical protein	
A1S_1044	13.44894606	3.07033E-15	Co/Zn/Cd efflux system	
A1S_1453	13.43213408	0	arsenite inducible repressor; K03892 ArsR family t	
A1S_3114	13.04141883	0	hypothetical protein; K07168 CBS domain-containing	
A1S_1354	12.61457931	0	(Acyl-carrier protein) phosphodiesterase; K01118 F	
A1S_2195	11.72176221	0	hypothetical protein	
A1S_0564	10.9855186	0	hypothetical protein	
A1S_1265	10.52144872	0	putative transcriptional regulator	
A1S_1950	9.983766351	0	putative universal stress protein	
A1S_0472	9.900884173	0	2-isopropylmalate synthase (EC:2.3.3.13); K01649 2	
A1S_1746	9.064870888	0	putative transcriptional regulator	
A1S_2158	8.993575671	0	putative monooxygenase	
A1S_1932	8.632289698	0	hypothetical protein	
A1S_2230	8.208277713	0	hypothetical protein	
A1S_3372	8.145939494	6.65499E-14	putative short-chain dehydrogenase	
A1S_1377	7.99103531	0	AcrR family transcriptional regulator	
A1S_1631	7.864097289	0	iron-binding protein; K04488 nitrogen fixation pro	
A1S_1986	7.793688943	0	fumC; fumarate hydratase (EC:4.2.1.2); K01679 fuma	
A1S_2820	7.688629159	0	hypothetical protein; K09946 hypothetical protein	
A1S_0094	7.507984548	0	lrp regulon transcriptional regulator (AsnC family	
A1S_0441	7.492589976	0	hypothetical protein	
A1S_3104	7.387594879	0	putative ATP-dependent RNA helicase	
A1S_1832	7.384020967	0	oxidoreductase FMN-binding	
A1S_2689	7.352439531	0	TetR family transcriptional regulator	
A1S_2503	7.175714788	0	putative outer membrane lipoprotein; K03098 outer	
A1S_1002	7.083624134	0	hypothetical protein; K09965 hypothetical protein	
A1S_3285	7.027808896	0	hypothetical protein	
A1S_1729	6.964327853	0	putative acetyl-CoA acetyltransferase; K00626 acet	
A1S_2412	6.827861759	0	hypothetical protein; K02616 phenylacetic acid deg	
A1S_3139	6.61037302	0	putative signal peptide	
A1S_3472	6.521522097	0	DNA replication protein	
A1S_2635	6.274688559	2.66897E-12	hypothetical protein	
A1S_2093	5.941346188	0	hypothetical protein	
A1S_1503	5.924184289	0	transmembrane pair	
A1S_0623	5.845114509	2.90904E-14	DNA mismatch repair enzyme	
A1S_1355	5.510061338	0	p-hydroxybenzoate hydroxylase transcriptional acti	
A1S_2690	5.507968863	0	hypothetical protein	
A1S_1171	5.475511359	1.09245E-10	hypothetical protein	
A1S_3286	5.377894129	0	putative inner membrane protein	
A1S_2183	5.314297643	0	putative signal peptide	
A1S_1273	5.308245441	0	hypothetical protein	
A1S_0603	5.308179708	0	ihfA; integration host factor subunit alpha; K0476	
A1S_2573	5.301886263	0.003258733	23-dihydroxybenzoate-AMP ligase	
A1S_2289	5.177855408	0	putative signal peptide	

Feature ID	Baggerley's test: BZK vs LB normalized values - Weighted proportions fold change	Baggerley's test: BZK vs LB normalized values - FDR p-value correction	Annotation	Categorization
A1S_2681	5.174117409	0	cell division protein; K03798 cell division protea	
A1S_2057	5.14264865	0	major facilitator superfamily methyl viologen resi	
A1S_1322	5.085597827	0	dihydrodipicolinate synthetase; K01714 dihydrodipi	
A1S_1172	5.007825886	0	putative transposase	
A1S_3468	4.948271214	0	putative lipoprotein	
A1S_0946	4.823788421	0.007482583	hypothetical protein	
A1S_2491	4.705279148	0	putative signal peptide	
A1S_1645	4.701041989	0	hypothetical protein	
A1S_0310	4.591649413	0	uvrC; excinuclease ABC subunit C; K03703 excinucle	
A1S_1614	4.591370161	0	hypothetical protein	
A1S_3469	4.576902268	1.56735E-15	diaminopimelate decarboxylase	
A1S_3174	4.554985143	0	putative regulatory or redox component complexing	
A1S_0563	4.499207968	0	MFS family transporter	
A1S_2067	4.47723035	7.50627E-15	transcriptional regulatory protein	
A1S_3260	4.389724683	0	hypothetical protein	
A1S_1515	4.346619901	0	hypothetical protein	
A1S_3259	4.315265386	0	putative transcriptional regulator YdzF	
A1S_0617	4.280405166	0	hypothetical protein	
A1S_1045	4.17312954	0	Co/Zn/Cd efflux system	
A1S_2696	4.119884322	0	hypothetical protein	
A1S_1410	4.023015839	0	LysR family transcriptional regulator	
A1S_0738	4.001853374	0	putative flavoprotein oxidoreductase; K09024 flavi	
A1S_1062	3.987639067	4.24892E-11	putative FMN oxidoreductase	
A1S_1647	3.958304342	1.56735E-15	putative siderophore biosynthesis protein	
A1S_0926	3.906712604	2.77439E-14	choline dehydrogenase	
A1S_2699	3.904461283	3.64501E-09	putative transcriptional regulator	
A1S_1409	3.856946656	1.25611E-05	putative short-chain dehydrogenase; K00059 3-oxoac	
A1S_3054	3.852474953	0	monooxygenase	
A1S_1422	3.837188931	0.000364447	triphosphoribosyl-dephospho-CoA synthase (EC:2.7.8	
A1S_3007	3.801443836	0	acyl-CoA dehydrogenase; K11731 citronellyl-CoA deh	
A1S_0011	3.76449792	0	hypothetical protein; K15724 iron-sulfur cluster i	
A1S_1454	3.760497447	5.30704E-13	transmembrane arsenate pump protein; K03325 arseni	
A1S_2473	3.716503428	0	LysR family transcriptional regulator	
A1S_0546	3.708711187	0	hypothetical protein	
A1S_2642	3.667874371	0	regulatory protein TetR	
A1S_2382	3.638595202	4.52421E-05	BasD	
A1S_2303	3.633154293	0	LysR family transcriptional regulator	
A1S_3284	3.630101374	0	putative transcriptional regulator	
A1S_1730	3.627773861	4.54023E-15	short-chain fatty acid transporter; K02106 short-c	
A1S_0927	3.61799566	0	betaine aldehyde dehydrogenase (EC:1.2.1.8); K0013	
A1S_0682	3.602201823	0	RNA polymerase factor sigma-54 (EC:2.7.7.6); K0309	
A1S_1324	3.555769314	0	hypothetical protein	
A1S_0776	3.535713745	0	TetR family transcriptional regulator	
A1S_2500	3.532626775	0	hypothetical protein	
A1S_2058	3.530334142	0	putative transcriptional regulator	
A1S_0088	3.506655155	4.03201E-12	hypothetical protein	
A1S_0132	3.5043309	0	putative transcriptional regulator	
A1S_0652	3.497144341	4.50054E-09	putative ferrous iron transport protein A; K04758	

Feature ID	Baggerley's test: BZK vs LB normalized values - Weighted proportions fold change	Baggerley's test: BZK vs LB normalized values - FDR p-value correction	Annotation	Categorization
A1S_1384	3.479536905	0.003119402	CinA-like protein; K03743	
A1S_2599	3.461984208	0	hypothetical protein	
A1S_2682	3.454112143	5.68327E-08	cell division protein; K02427 23S rRNA (uridine255	
A1S_1566	3.385061064	0	putative 6-pyruvoyl-tetrahydropterin synthase; K01	
A1S_2301	3.377607258	2.03279E-05	amino acid ABC transporter permease; K10016 histid	
A1S_3116	3.370676689	0	hypothetical protein	
A1S_0924	3.34087498	0	choline dehydrogenase; K00108 choline dehydrogenas	
A1S_1378	3.336922282	4.8656E-08	putative long chain fatty-acid CoA ligase; K00666	
A1S_2703	3.310893426	0	hypothetical protein	
A1S_1916	3.307321196	0	threonine dehydratase; K01754 threonine dehydratas	
A1S_1456	3.307098721	3.07033E-15	putative chromate transport protein; K07240 chroma	
A1S_2600	3.263150038	0	hypothetical protein	
A1S_3467	3.251955165	0	hypothetical protein	
A1S_2850	3.247027869	0	putative acyl-CoA transferase/carnitine dehydratas	
A1S_1452	3.229996513	6.55518E-08	arsenate reductase; K00537 arsenate reductase [EC:	
A1S_0676	3.218038905	0	putative transposase	
A1S_0925	3.209130475	0	choline dehydrogenase	
A1S_1180	3.167175274	0	putative Zn-dependent protease with chaperone func	
A1S_0478	3.157475019	0	putative signal peptide	
A1S_1345	3.138838498	0	hypothetical protein; K01912 phenylacetate-CoA lig	
A1S_3010	3.138711696	6.24963E-10	hypothetical protein	
A1S_2062	3.083145465	0	acetyl-CoA acetyltransferase (EC:2.3.1.9); K00626	
A1S_0648	3.071480459	0	hypothetical protein	
A1S_1437	3.042551195	4.27429E-09	putative acyl-CoA dehydrogenase	
A1S_0813	3.038049113	0	hypothetical protein; K07566 tRNA threonylcarbamoy	
A1S_1644	3.03105947	0	hypothetical protein	
A1S_2446	3.022158388	0	high-affinity phosphate transport protein; K02038	
A1S_0012	3.01962152	0	hydrolase	
A1S_3292	3.017556361	4.68292E-13	hypothetical protein; K08987 putative membrane pro	
A1S_0244	3.017300708	0	hypothetical protein	
A1S_0806	2.941510238	0	adenosylmethionine-8-amino-7-oxononanoate aminotra	
A1S_1425	2.925731046	0.004194835	malonate decarboxylase gamma subunit; K13933 malon	
A1S_2447	2.921490474	0	EsvD; K02037 phosphate transport system permease p	
A1S_1827	2.903955128	4.50479E-09	hypothetical protein	
A1S_3053	2.882837991	2.55349E-09	acyl coenzyme A dehydrogenase; K00255 long-chain-a	
A1S_1518	2.881617262	0	putative suppressor of F exclusion of phage T7; K0	
A1S_1831	2.878715054	0.002072038	hypothetical protein	
A1S_2252	2.857615568	0	putative colicin V producing membrane protein; K03	
A1S_2706	2.85041924	0	sigma D (sigma 70) factor of RNA polymerase major	
A1S_1774	2.840716203	2.17473E-05	putative hydrolase; K01463 [EC:3.5.1.-]	
A1S_0917	2.82866557	8.0093E-05	transcriptional regulator	
A1S_2159	2.826849292	4.54023E-15	hypothetical protein	
A1S_2259	2.809184096	0	putative signal peptide	
A1S_0979	2.808313408	0	putative membrane-bound protein in GNT I transport	
A1S_3392	2.796648575	0	phosphatidylglycerophosphatase A; K01095 phosphati	
A1S_1046	2.79553061	0	lysine exporter protein LysE/YggA; K06895 L-lysine	
A1S_1333	2.78824935	4.90208E-09	putative amino acid transporter	
A1S_0183	2.775882137	0	hypothetical protein	

Feature ID	Baggerley's test: BZK vs LB normalized values - Weighted proportions fold change	Baggerley's test: BZK vs LB normalized values - FDR p-value correction	Annotation	Categorization
A1S_2624	2.774202227	0.001308567	putative CPS-53 prophage bactoprenol glucosyl tran	
A1S_0093	2.772692195	3.31378E-08	hypothetical protein	
A1S_3381	2.730801201	0.006680101	AnkB protein; K06867	
A1S_0663	2.729975934	1.10693E-07	putative DNA helicase; K07505 hypothetical protein	
A1S_1387	2.72545311	2.24183E-05	oxidoreductase	
A1S_1450	2.714006129	2.95845E-05	hypothetical protein	
A1S_1216	2.70748564	5.98262E-06	LysR regulator	
A1S_3428	2.694014917	1.67738E-06	putative glucose dehydrogenase precursor	
A1S_0670	2.693314723	1.91321E-14	protein tyrosine phosphatase	
A1S_1426	2.686574073	0.000171759	phosphoribosyl-dephospho-CoA transferase; K13934 p	
A1S_0771	2.67884142	1.06105E-11	hypothetical protein	
A1S_2083	2.672074529	3.62197E-08	AsnC family transcriptional regulator; K03719 Lrp/	
A1S_0667	2.667891289	0.002208659	hypothetical protein	
A1S_0661	2.625676582	4.90208E-09	phage integrase family protein	
A1S_0317	2.623444049	0	putative fusaric acid resistance protein	
A1S_2598	2.622424738	0	RNA polymerase factor sigma-70 (EC:2.7.7.6); K0308	
A1S_0785	2.616222307	0	hypothetical protein	
A1S_0320	2.599062795	0	hypothetical protein; K07735 putative transcriptio	
A1S_1642	2.598503457	9.00524E-08	putative acyl-CoA dehydrogenase; K00249 acyl-CoA d	
A1S_1920	2.59456333	0	putative metalloprotease; K06972	
A1S_3462	2.589663587	0	hypothetical protein	
A1S_2548	2.587901952	0	putative enoyl-CoA hydratase/isomerase	
A1S_1524	2.586415491	0	hypothetical protein	
A1S_0666	2.575529436	0.00022578	TrbL/VirB6 plasmid conjugal transfer protein; K032	
A1S_2387	2.567275695	9.17527E-08	BauE; K02013 iron complex transport system ATP-bin	
A1S_1696	2.563847365	0	hypothetical protein	
A1S_0977	2.562985185	1.78659E-06	arylsulfatase; K01130 arylsulfatase [EC:3.1.6.1]	
A1S_1654	2.561347414	9.70335E-09	putative demethylmenaquinone methyltransferase	
A1S_3399	2.560408045	2.95915E-09	VirP protein; K01091 phosphoglycolate phosphatase	
A1S_1457	2.55886412	0.000285708	putative chromate transport protein; K07240 chroma	
A1S_0210	2.541456729	0	transposase; K07497 putative transposase	
A1S_0423	2.539725811	0	truA; tRNA pseudouridine synthase A (EC:5.4.99.12)	
A1S_1225	2.514593284	0	peptidase S24 S26A and S26B	
A1S_1217	2.51441541	3.18318E-10	heavy metal translocating P-type ATPase; K01533 Cu	
A1S_1687	2.491708173	0	transcriptional regulator	
A1S_2558	2.487380028	0.000126118	putative transposase	
A1S_0558	2.481241574	0	aconitate hydratase 1; K01681 aconitate hydratase	
A1S_1344	2.468221858	5.82749E-14	thiolase; K00680 [EC:2.3.1.-]	
A1S_0356	2.467876717	3.76016E-07	exonuclease V beta chain	
A1S_0665	2.46161689	2.80335E-11	conjugal transfer protein TrbJ	
A1S_1291	2.460461023	0	hypothetical protein	
A1S_2299	2.457329318	1.25161E-06	ABC transporter; K10017 histidine transport system	
A1S_2958	2.455734764	0	putative transcriptional regulator	
A1S_2616	2.451066169	0	hypothetical protein	
A1S_2755	2.450949991	2.06751E-08	putative acyltransferase	
A1S_1007	2.449257541	2.34549E-14	putative transcriptional regulator	
A1S_2641	2.446485481	5.144E-07	glycerate kinase; K00865 glycerate kinase [EC:2.7.	
A1S_3115	2.443767768	2.51469E-06	hypothetical protein	

Feature ID	Baggerley's test: BZK vs LB normalized values - Weighted proportions fold change	Baggerley's test: BZK vs LB normalized values - FDR p-value correction	Annotation	Categorization
A1S_0161	2.423584805	0	MFS family transporter	
A1S_2971	2.409640467	1.71542E-06	putative vanillate O-demethylase oxygenase subunit	
A1S_0009	2.399338232	0	putative RND type efflux pump	
A1S_2999	2.391994205	0	4-hydroxythreonine-4-phosphate dehydrogenase; K000	
A1S_3471	2.386312134	5.50194E-11	hypothetical protein	
A1S_2302	2.381946736	3.1614E-08	lysine/arginine/ornithine ABC transporter periplas	
A1S_1931	2.380734766	0	hypothetical protein	
A1S_2077	2.380722897	0.001237494	putative outer membrane porin receptor for Fe(III)	
A1S_0980	2.370469137	2.42017E-11	ferric enterobactin receptor precursor; K16089 out	
A1S_0971	2.357499182	5.80358E-07	metH; B12-dependent methionine synthase (EC:2.1.1.	
A1S_1124	2.355536348	9.23218E-10	AraC family transcriptional regulator	
A1S_0741	2.349723412	0.003122536	hypothetical protein	
A1S_0871	2.337247322	2.36016E-11	putative metal-dependent hydrolase; K07044	
A1S_0945	2.330311104	0.00049909	putative ferredoxin; K05710 dioxygenase ferredoxin	
A1S_2290	2.328979626	6.01284E-05	putative secretion pathway ATPase; K02454 general	
A1S_2525	2.323977377	0	putative serine protease; K01362 [EC:3.4.21.-]	
A1S_3246	2.321044065	0.000663905	hypothetical protein	
A1S_2463	2.318571413	0	putative ribosomal large subunit pseudouridine syn	
A1S_2005	2.317998135	0.000281698	nitrite reductase; K00362 nitrite reductase (NAD(P	
A1S_2863	2.317573542	0	putative antioxidant protein	
A1S_0843	2.31670517	0	putative flavodoxin or tryptophan repressor bindin	
A1S_1942	2.295317279	0	hypothetical protein	
A1S_0837	2.294983922	0	hemA; glutamyl-tRNA reductase; K02492 glutamyl-tRN	
A1S_2601	2.287209408	0	putative outer membrane protein A	
A1S_2073	2.28130789	9.32345E-08	ABC transporter ATPase	
A1S_1356	2.278266178	3.12603E-07	4-hydroxybenzoate 3-monooxygenase (EC:1.14.13.2);	
A1S_2557	2.271634611	0.00048859	hypothetical protein	
A1S_1781	2.26960285	0.007533801	putative ribose-phosphate pyrophosphokinase; K0094	
A1S_2179	2.251886809	6.67778E-10	hypothetical protein	
A1S_3339	2.247305336	0.000109564	putative ferric siderophore receptor protein; K020	
A1S_2988	2.23816382	1.77653E-14	hypothetical protein; K07098	
A1S_2101	2.231813447	2.23453E-08	putative transcriptional regulator	
A1S_3443	2.222586687	0	chaperone protein DnaJ; K03686 molecular chaperone	
A1S_3051	2.220564477	1.63172E-14	hypothetical protein	
A1S_0775	2.218478755	7.04054E-05	MFS family transporter	
A1S_2499	2.218334031	9.59901E-08	hypothetical protein	
A1S_1867	2.217153217	0.004623607	major facilitator transporter	
A1S_0769	2.202978032	0	ferredoxin--NADP+ reductase; K00528 ferredoxin--NA	
A1S_2386	2.202112704	3.99336E-07	putative ferric acinetobactin binding protein; K02	
A1S_2550	2.198929068	6.24366E-08	Tn7 transposase A	
A1S_2020	2.195706468	0	hypothetical protein; K07339 hypothetical protein	
A1S_0739	2.192974146	0.005956222	putative transcriptional regulator	
A1S_1421	2.191653112	0.002097886	malonate decarboxylase alpha subunit; K13929 malon	
A1S_0677	2.191310168	0.000566499	transposase	
A1S_0573	2.190802964	1.64028E-05	enoyl-CoA hydratase (EC:4.2.1.17)	
A1S_3474	2.185981945	6.45445E-12	hypothetical protein; K00076 7-alpha-hydroxysteroi	
A1S_0203	2.177136213	4.15347E-14	hypothetical protein; K01714 dihydrodipicolinate s	
A1S_2291	2.17304445	8.00127E-06	hypothetical protein	

Feature ID	Baggerley's test: BZK vs LB normalized values - Weighted proportions fold change	Baggerley's test: BZK vs LB normalized values - FDR p-value correction	Annotation	Categorization
A1S_1680	2.16860012	5.25367E-10	hypothetical protein	
A1S_3253	2.167999173	0	putative signal peptide	
A1S_2318	2.167983074	1.96729E-08	hypothetical protein	
A1S_2886	2.167762104	0	acyl-CoA dehydrogenase; K00257 [EC:1.3.99.-]	
A1S_1944	2.16502683	1.79115E-05	Alpha/beta hydrolase	
A1S_1958	2.162991185	0.007529017	putative transcriptional regulator	
A1S_0162	2.159393603	0	TetR/AcrR family transcriptional regulator	
A1S_3295	2.156313139	0	excinuclease ABC subunit A; K03701 excinuclease AB	
A1S_1049	2.153925095	2.4944E-05	hypothetical protein; K07460 putative endonuclease	
A1S_1993	2.1499028	1.29416E-08	regulatory protein GntR HTH	
A1S_2570	2.147460176	2.54435E-07	putative siderophore biosynthesis protein; putativ	
A1S_2467	2.146619701	0	hypothetical protein	
A1S_2196	2.142327341	0	membrane-associated dicarboxylate transport protei	
A1S_0628	2.140872814	0	putative transposase	
A1S_0234	2.140491073	0.000156963	type 4 fimbriae expression regulatory protein; K02	
A1S_2411	2.137354871	0.000255603	short chain dehydrogenase/reductase family oxidore	
A1S_0664	2.134904607	0.008937933	replication C family protein	
A1S_0172	2.134571724	4.2047E-06	hypothetical protein	
A1S_3326	2.13454092	0	hypothetical protein	
A1S_0737	2.134493971	0	5-methyltetrahydropteroyltriglutamate--homocystein	
A1S_1308	2.116250446	1.72413E-05	hypothetical protein; K11902 type VI secretion sys	
A1S_0721	2.115163278	6.16789E-11	glutaryl-CoA dehydrogenase; K00252 glutaryl-CoA de	
A1S_3016	2.11417465	2.64559E-09	hypothetical protein	
A1S_2251	2.112263919	3.07033E-15	amidophosphoribosyltransferase (EC:2.4.2.14); K007	
A1S_3043	2.095651193	0	hypothetical protein	
A1S_3315	2.077015139	0	ArsR family transcriptional regulator; K03892 ArsR	
A1S_0750	2.067923993	0	hypothetical protein	
A1S_0180	2.065880136	0.00042762	hypothetical protein	
A1S_0559	2.060679378	4.69368E-09	putative NAD(P)-binding enzyme	
A1S_0224	2.05969875	0	hypothetical protein	
A1S_2253	2.055465037	0	dihydroorotate dehydrogenase 2 (EC:1.3.3.1); K0022	
A1S_1678	2.054288817	0	putative histidine triad family protein; K02503 Hi	
A1S_0803	2.053491004	5.91638E-08	trehalose-6-phosphate synthase; K00697 trehalose 6	
A1S_2975	2.052750578	3.68993E-13	hypothetical protein	
A1S_2754	2.049742897	1.52434E-06	MFS family transporter	
A1S_1667	2.048379343	3.07033E-15	putative ferric hydroxamate siderophore receptor;	
A1S_2970	2.047174171	1.90807E-07	hypothetical protein	
A1S_0653	2.039619376	1.08032E-09	putative ferrous iron transport protein B; K04759	
A1S_0378	2.03455291	5.26915E-12	EsvG; K00311 electron-transferring-flavoprotein de	
A1S_0838	2.03380247	0	DNA primase; K02316 DNA primase [EC:2.7.7.-]	
A1S_1072	2.031005348	5.4859E-10	hypothetical protein	
A1S_0807	2.027573918	1.43853E-07	8-amino-7-oxononanoate synthase (EC:2.3.1.47); K00	
A1S_3461	2.027467789	7.19181E-11	DNA replication protein	
A1S_1096	2.026472744	0.001757	hypothetical protein	
A1S_1651	2.017837651	1.61676E-08	hypothetical protein	
A1S_2157	2.016442426	0	putative signal peptide	
A1S_3095	2.016028542	1.94912E-11	ATP-dependent DNA helicase RecG; K03655 ATP-depend	
A1S_2891	2.00486492	0	phospholipase D endonuclease domain-containing pro	

Feature ID	Baggerley's test: BZK vs LB normalized values - Weighted proportions fold change	Baggerley's test: BZK vs LB normalized values - FDR p-value correction	Annotation	Categorization
A1S_1679	2.004748932	0	putative signal peptide	
A1S_0786	2.004375801	4.54023E-15	putative signal peptide	
A1S_0800	-2.000951248	2.90328E-16	bacterioferritin; K03594 bacterioferritin	
A1S_1462	-2.001718562	7.07016E-55	hypothetical protein	
A1S_2432	-2.006752511	5.55333E-05	lipoprotein precursor; K06194 lipoprotein NlpD	
A1S_2128	-2.007866267	3.75253E-22	aconitate hydratase 2	
A1S_1686	-2.007962799	1.43853E-07	ribonuclease D; K03684 ribonuclease D [EC:3.1.13.5	
A1S_2663	-2.008425334	1.04677E-06	diacylglycerol kinase; K00901 diacylglycerol kinas	
A1S_3217	-2.018476822	2.29288E-05	RND divalent metal cation efflux transporter; K157	
A1S_1445	-2.018578938	0.000784179	tauD; taurine dioxygenase (EC:1.14.11.17); K03119	
A1S_0141	-2.020715231	2.32686E-44	putative dyp-type peroxidase; K07223 putative iron	
A1S_0756	-2.024811445	1.17869E-45	NADH dehydrogenase I chain F; K00335 NADH-quinone	
A1S_2508	-2.026709487	1.49983E-52	aspartate aminotransferase A; K00812 aspartate ami	
A1S_2712	-2.033877227	4.29773E-08	succinate dehydrogenase hydrophobic subunit; K0024	
A1S_2246	-2.034267929	2.31611E-40	hypothetical protein; K09117 hypothetical protein	
A1S_1685	-2.036331642	3.31745E-27	recR; recombination protein RecR; K06187 recombina	
A1S_2131	-2.041312765	2.29921E-06	hypothetical protein	
A1S_1967	-2.042719349	1.41592E-23	lpxD; UDP-3-O-[3-hydroxymyristoyl] glucosamine N-a	
A1S_0429	-2.042898152	2.0191E-31	DAACS family glutamate:aspartate symporter; K11102	
A1S_0514	-2.043410183	6.65388E-07	hypothetical protein	
A1S_2126	-2.045076537	3.76917E-09	aconitate hydratase 2; K01682 aconitate hydratase	
A1S_0086	-2.045552824	0.000999501	hypothetical protein	
A1S_0984	-2.046294762	1.68724E-13	putative carbonic anhydrase; K01673 carbonic anhyd	
A1S_2628	-2.047444956	1.12406E-21	electron transfer flavoprotein beta-subunit; K0352	
A1S_2984	-2.049421983	3.44135E-07	rpmH; 50S ribosomal protein L34; K02914 large subu	
A1S_0747	-2.052576052	2.08916E-98	ribonucleotide-diphosphate reductase subunit alpha	
A1S_1080	-2.055473928	3.05653E-20	putative lipoprotein	
A1S_1579	-2.060095907	1.04419E-34	putative ATPase; K06916	
A1S_2127	-2.060639051	1.57182E-41	aconitate hydratase 2; K01682 aconitate hydratase	
A1S_0071	-2.06096251	4.80136E-31	aromatic amino acid aminotransferase (EC:2.6.1.57)	
A1S_2916	-2.061987548	8.6291E-13	IISP family preprotein translocase membrane subuni	
A1S_0126	-2.06217835	5.07783E-08	hypothetical protein	
A1S_1974	-2.068532824	2.01821E-24	ribosome releasing factor; K02838 ribosome recycli	
A1S_2900	-2.069215638	9.65868E-08	putative lipopolysaccharide core biosynthesis glyc	
A1S_3342	-2.070936214	3.01615E-10	putative arsenate reductase	
A1S_1610	-2.073930939	0.000334987	Zn-dependent oligopeptidase; K01417 [EC:3.4.24.-]	
A1S_0818	-2.074287159	4.317E-122	hypothetical protein; K00059 3-oxoacyl-[acyl-carri	
A1S_2914	-2.076306223	6.74875E-20	secD; preprotein translocase subunit SecD; K03072	
A1S_1527	-2.076504669	1.95877E-08	tRNA (uracil-5-)-methyltransferase (EC:2.1.1.35);	
A1S_1965	-2.085592771	7.21234E-94	lpxA - lipidA - UDP-N-acetylglucosamine acyltransferase (EC:2.3.1.	
A1S_3332	-2.085829205	6.80214E-40	cell division protein; K03590 cell division protei	
A1S_3237	-2.087344372	0.000925012	exonuclease putative	
A1S_0996	-2.090200113	0.006753406	hypothetical protein	
A1S_0475	-2.090809252	1.25951E-42	tig; trigger factor; K03545 trigger factor	
A1S_0936	-2.092149157	4.76397E-08	hypothetical protein	
A1S_2924	-2.092339471	1.34901E-47	putative rhodanese-related sulfurtransferase	
A1S_1562	-2.092655689	0.008792986	putative general secretion pathway protein G precu	
A1S_2903	-2.092969221	0.002147164	hypothetical protein; K07276 hypothetical protein	

Feature ID	Baggerley's test: BZK vs LB normalized values - Weighted proportions fold change	Baggerley's test: BZK vs LB normalized values - FDR p-value correction	Annotation	Categorization
A1S_2671	-2.09590875	2.32503E-07	MFS permease; K03761 MFS transporter	
A1S_3041	-2.095934393	8.72368E-09	hypothetical protein	
A1S_1947	-2.097110567	1.27068E-09	phosphotransferase system fructose-specific IIBC c	
A1S_2879	-2.098369657	3.11412E-06	putative glycerophosphodiester phosphodiesterase;	
A1S_0451	-2.102100365	4.64073E-17	formyltetrahydrofolate deformylase; K01433 formylt	
A1S_3425	-2.117412176	4.33335E-56	phosphoribosylaminoimidazole-succinocarboxamide sy	
A1S_0764	-2.119653359	9.16414E-36	NADH dehydrogenase I chain N; K00343 NADH-quinone	
A1S_1973	-2.121176033	1.97428E-18	undecaprenyl pyrophosphate synthetase; K00806 unde	
A1S_0308	-2.123500703	2.77466E-15	beta-hydroxylase; K12979 beta-hydroxylase [EC:1.14	
A1S_1945	-2.123599245	8.44669E-08	phosphotransferase system fructose-specific EI/HPr	
A1S_0758	-2.123890789	6.31059E-63	NADH dehydrogenase subunit H (EC:1.6.5.3); K00337	
A1S_0055	-2.126602814	1.03299E-60	WecE protein; K13017 UDP-3-keto-D-GlcNAcA aminotra	
A1S_1538	-2.127174996	9.45631E-20	serine/threonine transporter SstT; K07862 serine/t	
A1S_0227	-2.13104862	5.80915E-32	leucyl aminopeptidase; K01255 leucyl aminopeptidas	
A1S_2870	-2.133451493	4.75761E-53	trpA; tryptophan synthase subunit alpha (EC:4.2.1.	
A1S_2424	-2.134517954	0.00150842	putative Na+-dependent transporter	
A1S_2207	-2.135056773	0.001317454	membrane protein putative	
A1S_2661	-2.142768305	0.002133016	glycerophosphoryl diester phosphodiesterase; K0112	
A1S_1113	-2.146279802	0.002932652	putative transcriptional regulator	
A1S_3248	-2.148703548	4.10796E-15	glycerol uptake facilitator; K06188 aquaporin Z	
A1S_3449	-2.149748674	1.03658E-11	phosphoenolpyruvate carboxylase (EC:4.1.1.31); K01	
A1S_2427	-2.155936819	1.08148E-09	putative transporter; K08218 MFS transporter	
A1S_3365	-2.157140773	4.80112E-13	disulfide bond formation protein; K03611 disulfide	
A1S_2951	-2.161338532	2.90539E-34	putative sulfide dehydrogenase	
A1S_2320	-2.1644705	1.28339E-05	AraC family transcriptional regulator	
A1S_3250	-2.166036315	6.48814E-09	hypothetical protein	
A1S_1532	-2.167392331	1.02628E-15	glycine cleavage complex protein H; K02437 glycine	
A1S_3202	-2.16754318	2.62788E-11	UDP-N-acetylmuramoyl-tripeptide--D-alanyl-D-alanin	
A1S_0049	-2.168450846	3.47042E-33	protein tyrosine kinase; K08253 non-specific prote	
A1S_0746	-2.170641624	1.7663E-104	ribonucleotide-diphosphate reductase subunit beta	
A1S_0630	-2.171907811	1.99216E-24	hypothetical protein	
A1S_0322	-2.173075849	6.96056E-05	hypothetical protein	
A1S_2789	-2.17565432	3.38454E-05	putative metallopeptidase; K07386 putative endopep	
A1S_0257	-2.178169677	1.25718E-06	hypothetical protein	
A1S_3331	-2.179344914	1.2492E-158	cell division protein FtsZ; K03531 cell division p	
A1S_2782	-2.181253665	3.48372E-22	aspartyl/glutamyl-tRNA(Asn/Gln) amidotransferase s	
A1S_0201	-2.183709222	4.93825E-74	putative outer membrane protein	
A1S_1296	-2.185146546	3.51214E-34	hypothetical protein; K11903 type VI secretion sys	
A1S_1761	-2.186139736	0.001693056	acetyltransferase; K00680 [EC:2.3.1.-]	
A1S_2225	-2.193830919	0.004759489	hypothetical protein	
A1S_1499	-2.194793162	7.59366E-09	hypothetical protein	
A1S_2901	-2.195504478	2.01278E-10	putative polysaccharide deacetylase	
A1S_0497	-2.20077818	4.52663E-08	hypothetical protein	
A1S_1565	-2.20364136	4.70983E-06	general secretion pathway protein K; K02460 genera	
A1S_2968	-2.206016559	5.88907E-06	hypothetical protein	
A1S_2757	-2.207469932	3.69734E-14	hypothetical protein; K07340 hypothetical protein	
A1S_1536	-2.210826362	9.3238E-09	putative nucleoprotein/polynucleotide-associated e	
A1S_1975	-2.211444065	2.60775E-46	pyrH; uridylate kinase; K09903 uridylate kinase [E	

Feature ID	Baggerley's test: BZK vs LB normalized values - Weighted proportions fold change	Baggerley's test: BZK vs LB normalized values - FDR p-value correction	Annotation	Categorization
A1S_0759	-2.212641686	1.1739E-100	NADH dehydrogenase subunit I (EC:1.6.5.3); K00338	
A1S_3389	-2.215272425	2.42026E-47	ribH; 6-7-dimethyl-8-ribityllumazine synthase; K00	
A1S_3451	-2.220761515	8.50511E-06	uracil transport protein	
A1S_3199	-2.22281093	3.44135E-07	hypothetical protein	
A1S_1941	-2.230056185	2.5855E-18	branched chain amino acid transporter; K03311 bran	
A1S_2206	-2.23074303	0.00160322	paraquat-inducible protein A; K03808 paraquat-indu	
A1S_0289	-2.239360339	1.9775E-07	hypothetical protein	
A1S_1646	-2.239723447	0.000192584	hypothetical protein	
A1S_2479	-2.240574641	3.4448E-19	putative D-ala-D-ala-carboxypeptidase penicillin-b	
A1S_2169	-2.240931955	2.34606E-06	cytochrome o ubiquinol oxidase subunit IV; K02300	
A1S_0886	-2.242981828	1.24451E-14	deoxyuridine 5'-triphosphate nucleotidohydrolase;	
A1S_0148	-2.243219249	6.6428E-242	F0F1 ATP synthase subunit A (EC:3.6.3.14); K02108	
A1S_0590	-2.244280597	1.67741E-06	hypothetical protein; K09889 ribosome-associated p	
A1S_0484	-2.250337814	1.9606E-09	hypothetical protein; K01625 2-dehydro-3-deoxyphos	
A1S_1683	-2.250450009	1.94141E-17	O-succinylhomoserine sulfhydrylase; K10764 O-succi	
A1S_0524	-2.251062311	2.36796E-08	hypothetical protein; K00059 3-oxoacyl-[acyl-carri	
A1S_0431	-2.253847353	1.43619E-08	lpxL - lipid A biosynthesis lauroyl acyltransferase; K025	
A1S_0150	-2.254633617	4.6785E-298	membrane-bound ATP synthase F0 sector	
A1S_0522	-2.256876319	0.000138951	3-oxoacyl-(acyl carrier protein) synthase I (EC:2.	
A1S_0304	-2.258312461	0.000190278	hypothetical protein	
A1S_2821	-2.268192639	3.9869E-14	putative alkylphosphonate uptake protein (PhnA) in	
A1S_0882	-2.26854573	1.75856E-05	hypothetical protein	
A1S_2930	-2.270986416	7.74301E-22	putative ferrous iron transport protein B	
A1S_0047	-2.272335481	2.2672E-22	FKBP-type 22KD peptidyl-prolyl cis-trans isomerase	
A1S_0534	-2.275727967	1.67631E-23	NADH-dependent enoyl-ACP reductase; K00208 enoyl-[	
A1S_2738	-2.284125283	8.93429E-08	hypothetical protein	
A1S_2213	-2.295278689	0.001077569	CsuE	
A1S_2168	-2.302386048	3.42056E-34	cytochrome o ubiquinol oxidase subunit III; K02299	
A1S_2165	-2.303701778	9.60187E-20	hypothetical protein	
A1S_1619	-2.304099504	3.31883E-15	ribonuclease activity regulator protein RraA; K025	
A1S_1293	-2.306811162	1.28379E-06	hypothetical protein	
A1S_2708	-2.306972	4.72228E-58	hypothetical protein; K07146 UPF0176 protein	
A1S_0159	-2.309024061	2.79574E-33	glutathione peroxidase; K00432 glutathione peroxid	
A1S_0763	-2.309370423	3.95824E-94	NADH dehydrogenase subunit M (EC:1.6.5.3); K00342	
A1S_1318	-2.31460088	2.73429E-08	GCN5-related N-acetyltransferase	
A1S_2911	-2.315501387	3.65229E-05	hypothetical protein; K03744 LemA protein	
A1S_3289	-2.315753807	3.08807E-09	hypothetical protein; K09799 hypothetical protein	
A1S_0259	-2.316039628	1.05534E-47	argininosuccinate lyase; K01755 argininosuccinate	
A1S_0390	-2.317426248	5.88816E-17	putative type III effector	
A1S_1496	-2.322337902	6.97461E-10	hypothetical protein	
A1S_0533	-2.326934577	9.81841E-68	hypothetical protein	
A1S_1443	-2.335177979	0.000171099	taurine ATP-binding transport system component; K1	
A1S_1552	-2.343006087	4.40976E-17	chromosome partitioning protein; K03497 chromosome	
A1S_2987	-2.345144256	3.78897E-31	putative lipoprotein precursor	
A1S_1676	-2.345147068	0.00074442	hypothetical protein	
A1S_0391	-2.349200948	5.14949E-12	rpmE2; 50S ribosomal protein L31 type B; K02909 la	
A1S_3037	-2.352586901	1.68294E-08	putative ribonuclease (Rbn); K07058 membrane prote	
A1S_2109	-2.35404958	6.13245E-06	lpxH - lipidA-peptidyl-prolyl cis-trans isomerase precursor; K03	

Feature ID	Baggerley's test: BZK vs LB normalized values - Weighted proportions fold change	Baggerley's test: BZK vs LB normalized values - FDR p-value correction	Annotation	Categorization
A1S_3241	-2.363299355	9.08939E-06	putative polyketide synthesis monooxygenase; K0714	
A1S_2163	-2.365712681	6.74027E-11	hypothetical protein; K09773 hypothetical protein	
A1S_2129	-2.366009579	2.81117E-13	hypothetical protein	
A1S_0515	-2.369325241	2.61023E-12	histidine ammonia-lyase protein; K01745 histidine	
A1S_1564	-2.372343425	2.87264E-05	general secretion pathway protein J precursor; K02	
A1S_2753	-2.378581545	1.687E-41	putative protein (DcaP-like)	
A1S_0151	-2.381888822	3.0669E-85	F0F1 ATP synthase subunit B; K02109 F-type H+-tran	
A1S_2441	-2.382935179	2.8538E-12	adenylosuccinate lyase (EC:4.3.2.2); K01756 adenyl	
A1S_2261	-2.383821719	2.76194E-05	putative cold shock protein; K03704 cold shock pro	
A1S_3444	-2.387089159	1.79647E-10	hypothetical protein	
A1S_2896	-2.388908559	2.65678E-09	hypothetical protein	
A1S_1665	-2.392416845	3.75545E-12	hypothetical protein; K07090	
A1S_0869	-2.401105988	7.59582E-53	elongation factor Tu (EC:3.6.5.3); K02358 elongati	
A1S_2929	-2.411338702	7.01596E-07	putative cation efflux system protein	
A1S_2203	-2.412474281	7.89482E-09	hypothetical protein; K09857 hypothetical protein	
A1S_0483	-2.414947224	3.56017E-18	phosphogluconate dehydratase (EC:4.2.1.12); K01690	
A1S_2985	-2.418300994	8.07124E-12	hypothetical protein	
A1S_2450	-2.420257757	7.7414E-15	putative pyruvate decarboxylase; K04103 indolepyru	
A1S_1509	-2.420285756	1.58992E-06	pili assembly chaperone	
A1S_0323	-2.427571031	1.72117E-35	hypothetical protein; K09913 hypothetical protein	
A1S_1061	-2.428167163	0.000193573	putative oligopeptide transport protein	
A1S_2426	-2.429450565	8.61334E-33	lactoylglutathione lyase; K01759 lactoylglutathion	
A1S_1639	-2.429642107	5.33657E-56	peptidyl-prolyl cis-trans isomerase precursor	
A1S_2627	-2.436591627	1.05514E-06	electron transfer flavoprotein alpha-subunit; K035	
A1S_1250	-2.446774859	7.58642E-12	GCN5-related N-acetyltransferase	
A1S_3228	-2.448816388	1.98018E-13	putative RNA binding protein	
A1S_2460	-2.449877059	3.09699E-28	TetR family transcriptional regulator	
A1S_0877	-2.45309106	5.37849E-08	threonine efflux system; K05835 threonine efflux p	
A1S_1442	-2.453278511	3.83139E-06	taurine ABC transporter periplasmic taurine-bindin	
A1S_3419	-2.456662602	0.007511126	hypothetical protein	
A1S_1330	-2.460020255	0.002643211	hypothetical protein	
A1S_2859	-2.466846315	1.87777E-18	putative hemolysin III (HLY-III); K11068 hemolysin	
A1S_2849	-2.470281179	2.37367E-16	putative glucose-sensitive porin (OprB-like); K07	
A1S_0762	-2.47071103	3.94491E-45	NADH dehydrogenase subunit L; K00341 NADH-quinone	
A1S_2297	-2.476011584	1.56517E-14	putative 4Fe-4S ferredoxin	
A1S_2316	-2.484226608	5.24145E-16	membrane-bound lytic murein transglycosylase B; K0	
A1S_0248	-2.494578917	1.55507E-90	DnaK suppressor protein; K06204 DnaK suppressor pr	
A1S_0883	-2.495987781	1.78368E-09	putative acyltransferase	
A1S_2201	-2.50193914	1.20609E-06	hypothetical protein	
A1S_3410	-2.514371526	1.8926E-06	putative acyltransferase	
A1S_0625	-2.525190765	8.89252E-11	TetR family transcriptional regulator	
A1S_3281	-2.526829098	1.3889E-11	4-aminobutyrate aminotransferase PLP-dependent; K0	
A1S_2655	-2.545787499	1.88724E-10	hypothetical protein	
A1S_2452	-2.559705186	8.49248E-54	NAD-dependent aldehyde dehydrogenases	
A1S_0152	-2.563076153	0	F0F1 ATP synthase subunit delta; K02113 F-type H+-	
A1S_2523	-2.563386374	6.25403E-05	ribosomal-protein-alanine acetyltransferase; K0067	
A1S_0525	-2.564835457	3.5023E-06	3-oxoacyl-(acyl carrier protein) synthase II (EC:2	
A1S_3434	-2.576469388	5.7528E-07	hypothetical protein	

Feature ID	Baggerley's test: BZK vs LB normalized values - Weighted proportions fold change	Baggerley's test: BZK vs LB normalized values - FDR p-value correction	Annotation	Categorization
A1S_3390	-2.578299458	2.13275E-06	nusB; transcription antitermination protein NusB;	
A1S_2428	-2.600102875	2.07095E-31	putative ATP-dependent protease	
A1S_1937	-2.602732415	2.09925E-22	putative glutaredoxin-related protein; K07390 mono	
A1S_0054	-2.602805185	2.90484E-74	WbbJ protein; K13018 UDP-D-GlcNAc3NA acetyltransfe	
A1S_1398	-2.604056902	0.00051194	GlnQ protein; K02028 polar amino acid transport sy	
A1S_0997	-2.613106774	4.84152E-10	hypothetical protein	
A1S_0521	-2.614048503	6.34811E-11	hypothetical protein	
A1S_1605	-2.617177214	0.001762879	biopolymer transport proteins; K03561 biopolymer t	
A1S_0059	-2.626883214	1.40384E-13	putative glycosyltransferase	
A1S_3450	-2.628419394	1.25962E-17	uracil transport protein	
A1S_2342	-2.644868435	4.90208E-09	hypothetical protein	
A1S_0218	-2.649076276	1.13056E-11	nitrogen assimilation regulatory protein P-II 2; K	
A1S_0819	-2.669925031	2.45022E-14	acyl carrier protein (ACP); K02078 acyl carrier pr	
A1S_0279	-2.68647377	1.40113E-59	elongation factor Tu (EC:3.6.5.3); K02358 elongati	
A1S_2107	-2.694288945	2.53674E-08	glutamine amidotransferase (EC:6.3.5.2); K01951 GM	
A1S_1279	-2.702169358	0.007229717	hypothetical protein; K11746 glutathione-regulated	
A1S_2014	-2.705572023	0.000111243	hypothetical protein	
A1S_0153	-2.731275408	2.95989E-57	F0F1 ATP synthase subunit alpha (EC:3.6.3.14); K02	
A1S_3208	-2.737503401	4.68292E-13	putative peptide signal	
A1S_0486	-2.739430308	1.87456E-06	thermoresistant gluconokinase; K00851 gluconokinas	
A1S_3136	-2.74748267	2.34054E-37	hypothetical protein	
A1S_2793	-2.757851045	1.02023E-14	putative amino-acid transport protein; K03310 alan	
A1S_2785	-2.767518803	1.27134E-22	putative protease	
A1S_0518	-2.776863001	1.03394E-15	hypothetical protein	
A1S_0258	-2.786104741	1.04945E-06	argininosuccinate lyase	
A1S_1828	-2.798884552	0.00025143	MerR family transcriptional regulator	
A1S_2533	-2.811098786	3.43433E-10	putative esterase; K07002	
A1S_1195	-2.816849556	6.88972E-10	putative glutathione S-transferase; K00799 glutath	
A1S_3363	-2.827076339	0.000136513	membrane metalloendopeptidases proteins	
A1S_1139	-2.848402176	2.27861E-11	putative signal peptide	
A1S_1227	-2.85040717	0.001070064	amino acid transporter LysE	
A1S_1898	-2.852838292	4.01036E-69	eno; phosphopyruvate hydratase; K01689 enolase [EC	
A1S_2167	-2.855511949	1.85897E-50	cytochrome o ubiquinol oxidase subunit I; K02298 c	
A1S_2931	-2.888573869	9.14771E-19	hypothetical protein	
A1S_0154	-2.890821242	2.9145E-192	F0F1 ATP synthase subunit gamma; K02115 F-type H+-	
A1S_1636	-2.899404542	4.90208E-09	putative poly(hydroxyalcanoate) granule associated	
A1S_0519	-2.903881889	3.36904E-17	hypothetical protein	
A1S_0509	-2.904247709	1.14859E-24	putative acyl carrier protein; K02078 acyl carrier	
A1S_0155	-2.91839784	3.0504E-44	F0F1 ATP synthase subunit beta (EC:3.6.3.14); K021	
A1S_1638	-2.931769813	2.38592E-60	peptidyl-prolyl cis-trans isomerase precursor; K03	
A1S_0485	-2.935112389	4.16876E-31	GntP family high-affinity gluconate permease; K032	
A1S_2532	-2.938169293	0.002229236	sulfate transport protein	
A1S_0520	-2.958192449	1.31086E-12	putative oxidoreductase protein; putative dehydrog	
A1S_0517	-2.973353696	0.003944411	hypothetical protein	
A1S_1337	-2.980035038	5.09948E-19	paaB; phenylacetate-CoA oxygenase subunit PaaB; K0	
A1S_1684	-2.994085938	7.23969E-65	hypothetical protein; K09747 hypothetical protein	
A1S_0582	-3.0129495	0.001685862	putative DNA uptake protein; K02237 competence pro	
A1S_2322	-3.038059847	5.7267E-46	tsf; elongation factor Ts; K02357 elongation facto	

Feature ID	Baggerley's test: BZK vs LB normalized values - Weighted proportions fold change	Baggerley's test: BZK vs LB normalized values - FDR p-value correction	Annotation	Categorization
A1S_0761	-3.039860774	4.44848E-11	NADH dehydrogenase I chain K; K00340 NADH-quinone	
A1S_0631	-3.046713184	1.60792E-08	hypothetical protein	
A1S_1530	-3.051438487	2.30552E-79	SSS family major sodium/proline symporter; K11928	
A1S_2719	-3.051648462	3.14526E-26	succinyl-CoA synthetase alpha chain; K01902 succin	
A1S_0157	-3.121038691	4.33094E-56	hypothetical protein	
A1S_2296	-3.125877758	7.48313E-36	putative protease; K08303 putative protease [EC:3.	
A1S_0629	-3.131844479	1.89064E-15	hypothetical protein	
A1S_2042	-3.144355166	9.17402E-07	TetR family transcriptional regulator	
A1S_2210	-3.144428396	0.001014573	hypothetical protein	
A1S_2280	-3.15405193	3.23946E-40	C4-dicarboxylate transporter DctA; K11103 aerobic	
A1S_2202	-3.176320784	6.92457E-07	aspartate racemase; K01779 aspartate racemase [EC:	
A1S_0516	-3.176352544	0.000741244	hypothetical protein; K07107 acyl-CoA thioester hy	
A1S_0498	-3.180301891	1.411E-195	nucleoside diphosphate kinase; K00940 nucleoside-d	
A1S_2894	-3.195584046	3.3265E-100	aspS; aspartyl-tRNA synthetase (EC:6.1.1.12); K018	
A1S_3231	-3.235153825	3.6159E-165	putative acetyl-CoA hydrolase/transferase; K01076	
A1S_0548	-3.239502423	9.91104E-12	TetR family transcriptional regulator	
A1S_2758	-3.282613297	1.25548E-31	putative membrane protease subunit	
A1S_3301	-3.294940697	8.74357E-17	hypothetical protein	
A1S_1402	-3.299787182	0.000610907	putative amino acid efflux transmembrane protein	
A1S_2429	-3.346742359	6.9805E-12	putative ATP-dependent protease	
A1S_2710	-3.37631184	6.88932E-86	hypothetical protein; K01647 citrate synthase [EC:	
A1S_2166	-3.384248299	3.63979E-57	cytochrome o ubiquinol oxidase subunit II; K02297	
A1S_0510	-3.394103912	2.30143E-14	acyl carrier protein; K02078 acyl carrier protein	
A1S_0156	-3.402897101	8.62669E-76	atpC; F0F1 ATP synthase subunit epsilon; K02114 F-	
A1S_0640	-3.409603773	9.14969E-40	hypothetical protein	
A1S_1399	-3.412668183	1.17229E-05	ArtI protein; K02030 polar amino acid transport sy	
A1S_2435	-3.415506141	3.1508E-107	D-ala-D-ala-carboxypeptidase; penicillin-binding p	
A1S_0545	-3.440003676	5.7367E-151	acetohydroxy acid isomeroreductase; K00053 ketol-a	
A1S_1602	-3.445289034	0.000921981	hypothetical protein; K00657 diamine N-acetyltrans	
A1S_3350	-3.481163118	3.54295E-70	hypothetical protein	
A1S_2814	-3.507991631	1.1429E-12	twitching motility protein; K02658 twitching motil	
A1S_0370	-3.513483499	3.92262E-54	general secretion pathway protein G; K02456 genera	
A1S_0647	-3.515174704	1.81542E-22	IcmO protein; K12217 intracellular multiplication	
A1S_1968	-3.539510454	1.04401E-49	putative outer membrane protein (OmpH); K06142 out	
A1S_1726	-3.546483828	5.99704E-50	aspA; aspartate ammonia-lyase (EC:4.3.1.1); K01744	
A1S_2430	-3.554893932	6.32757E-36	putative ATP-dependent protease	
A1S_1926	-3.558634188	7.2739E-40	hypothetical protein	
A1S_1727	-3.571915984	2.11581E-09	LysR family transcriptional regulator	
A1S_1622	-3.574071496	4.57519E-05	hypothetical protein	
A1S_2662	-3.649039564	5.49033E-61	putative hydrolase	
A1S_3236	-3.702427925	2.02987E-57	hypothetical protein	
A1S_0820	-3.706540844	8.0569E-49	putative peptidoglycan-binding LysM	
A1S_1811	-3.864943925	4.59681E-07	hypothetical protein	
A1S_0200	-3.900672081	0	inorganic pyrophosphatase; K01507 inorganic pyroph	
A1S_0171	-3.984542009	6.92872E-24	hypothetical protein	
A1S_0523	-3.996719005	2.76751E-09	putative 3-hydroxylacyl-(acyl carrier protein) deh	
A1S_3377	-4.046349765	2.88119E-21	hypothetical protein	
A1S_3108	-4.072818935	3.56144E-78	coproporphyrinogen III oxidase (EC:1.3.3.3); K0022	

Feature ID	Baggerley's test: BZK vs LB normalized values - Weighted proportions fold change	Baggerley's test: BZK vs LB normalized values - FDR p-value correction	Annotation	Categorization
A1S_2718	-4.131258608	0	succinyl-CoA synthetase beta chain; K01903 succiny	
A1S_2419	-4.132634697	1.0924E-300	elongation factor P; K02356 elongation factor P	
A1S_0641	-4.216443238	1.54488E-18	hypothetical protein; K12218 intracellular multipl	
A1S_2214	-4.274246347	1.22629E-07	CsuD; K07347 outer membrane usher protein	
A1S_2531	-4.345879702	4.40715E-32	sulfate transport protein; K02048 sulfate transpor	
A1S_3355	-4.499039654	2.45779E-57	hypothetical protein	
A1S_2907	-4.50269762	0.004032604	hypothetical protein	
A1S_0643	-4.52229658	1.2106E-18	hypothetical protein; K12203 defect in organelle t	
A1S_3273	-4.57073967	7.61081E-25	putative peptide signal	
A1S_2461	-4.701274105	3.43275E-14	hypothetical protein	
A1S_1336	-4.899578773	8.1937E-127	paaA; phenylacetate-CoA oxygenase subunit PaaA; K0	
A1S_2215	-5.027066762	4.9001E-12	CsuC; K07346 fimbrial chaperone protein	
A1S_0302	-5.11676885	6.1111E-21	hypothetical protein	
A1S_0650	-5.17930782	1.78024E-87	conjugal transfer protein	
A1S_1281	-5.26926903	6.7957E-08	TPR domain-containing protein	
A1S_0646	-5.393327152	2.86641E-65	IcmB protein; K12206 intracellular multiplication	
A1S_1319	-5.598852458	4.2076E-100	hypothetical protein; K09765 hypothetical protein	
A1S_0057	-5.660497386	9.19562E-56	capsular polysaccharide synthesis enzyme	
A1S_0642	-5.670865716	1.4675E-64	hypothetical protein	
A1S_1199	-5.684976577	8.90521E-37	putative glutathionine S-transferase; K00799 gluta	
A1S_1335	-5.94334995	3.1954E-43	bifunctional aldehyde dehydrogenase/enoyl-CoA hydr	
A1S_1879	-6.029983749	7.7165E-182	hypothetical protein	
A1S_0058	-6.31134198	7.44514E-29	glycosyltransferase	
A1S_2926	-6.347396676	8.44594E-29	hypothetical protein	
A1S_1510	-6.371061959	6.06559E-82	fimbrial protein; K07345 major type 1 subunit fimb	
A1S_0526	-6.393239002	7.4179E-114	hypothetical protein	
A1S_2925	-7.125862901	7.83202E-56	hypothetical protein; K09767 hypothetical protein	
A1S_0644	-7.360779472	3.9322E-119	hypothetical protein; K12204 defect in organelle t	
A1S_1925	-7.521347555	1.55466E-30	cytochrome d terminal oxidase polypeptide subunit	
A1S_1637	-7.667999325	5.627E-246	DNA-binding protein HU-beta; K03530 DNA-binding pr	
A1S_1924	-8.728132166	6.4344E-141	cytochrome d terminal oxidase polypeptide subunit	
A1S_0645	-10.64224013	8.0908E-36	hypothetical protein; K12213 intracellular multipl	
A1S_2216	-11.1854056	5.88026E-05	CsuB	
A1S_2218	-11.79045854	1.4702E-56	CsuA/B	
A1S_2217	-12.33869284	2.02311E-06	CsuA	
A1S_0292	-25.05182385	2.48453E-34	putative outer membrane protein W; K07275 outer me	
A1S_0891	-45.67391906	1.06487E-13	hypothetical protein; K07216 hemerythrin	
