# Supplementary material for: Exploring the Antimicrobial Action of Quaternary Amines against Acinetobacter baumannii
Source: mBio. 2018 Feb 6;9(1):e02394-17. doi: 10.1128/mBio.02394-17 (PMC5801471; doi:10.1128/mBio.02394-17)
Supplement: FIG S1 [file mbo001183722sf1.pdf]

Figure S1.

A.

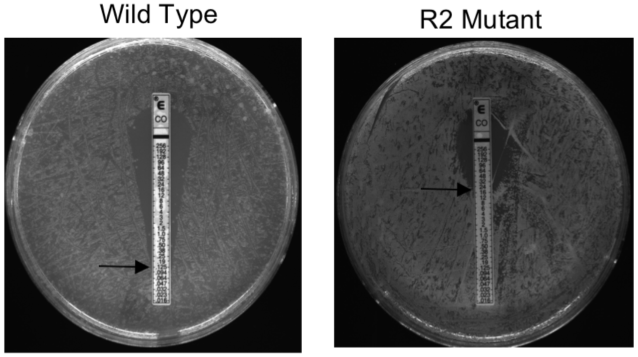

B.

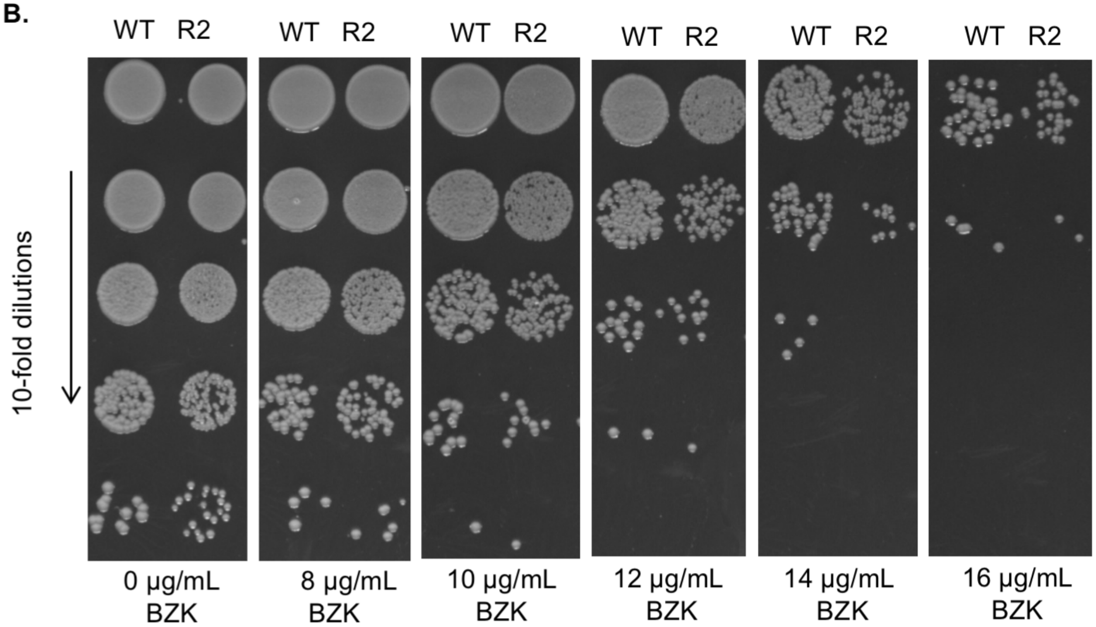

Figure S1. Surface charge alterations affect *A. baumannii* colistin resistance but not BZK resistance. A) Colistin sensitivity of wild type *A. baumannii* and R2 mutant. Colistin resistance of indicated strains using E-test colistin strip. The black arrows indicate the MIC. All experiments were repeated at least three times. A representative image of each is shown. B) Plating efficiency of *A. baumannii* wild type (WT) and R2 mutant strain colony forming units on increasing BZK concentrations were identical.
