# Supplementary material for: Exploring the Antimicrobial Action of Quaternary Amines against Acinetobacter baumannii
Source: mBio. 2018 Feb 6;9(1):e02394-17. doi: 10.1128/mBio.02394-17 (PMC5801471; doi:10.1128/mBio.02394-17)
Supplement: FIG S2 [file mbo001183722sf2.pdf]

# Figure S2.

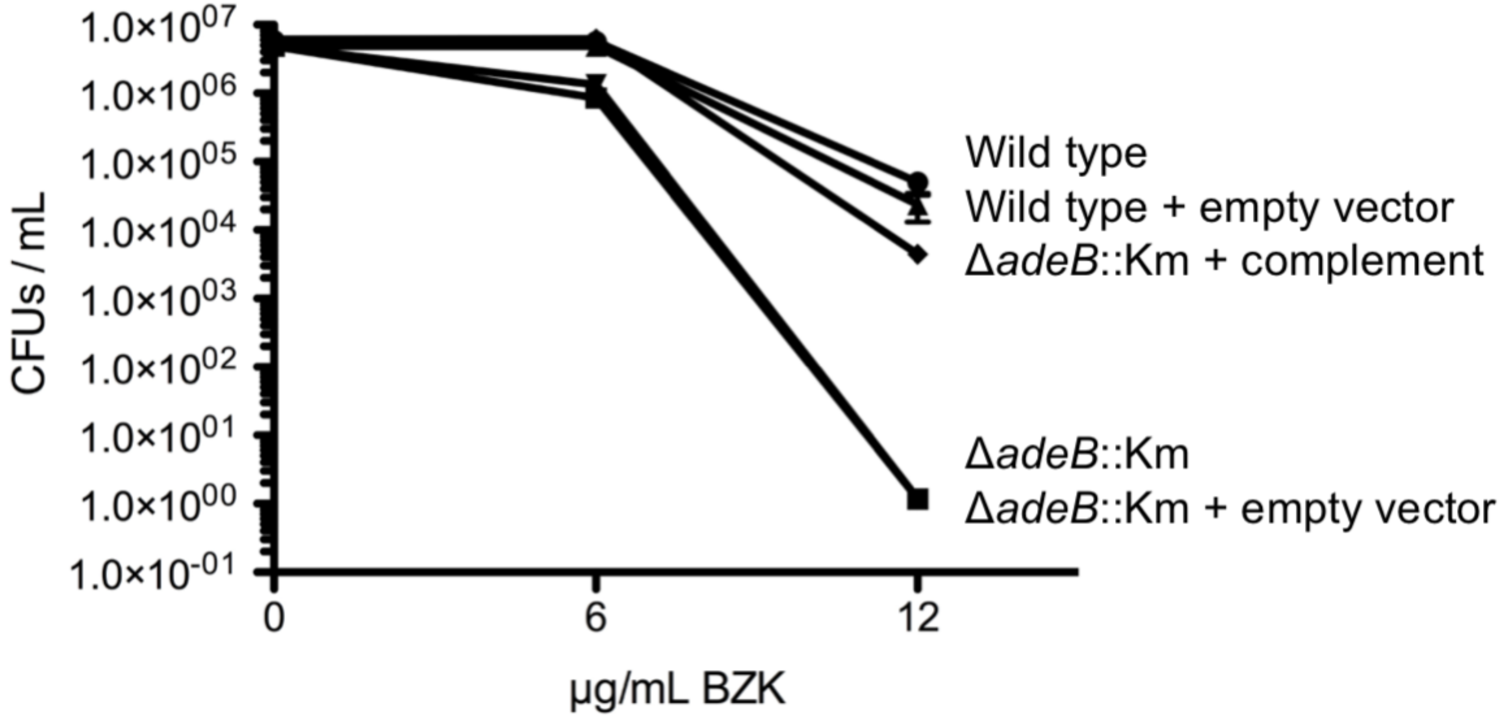


**Figure S2**. Plating efficiency of *A. baumannii* wild type (parental) and Δ*adeB* mutants on increasing concentrations of BZK agar. At 12μg/mL the wild type strain showed a higher plating efficiency; $p < 0.05$ unpaired two-tailed Student's t test. At 12 μg/mL the wild type+vector strain showed a higher plating efficiency than mutant+vector, but not the complemented strain; $p < 0.05$ One-way ANOVA, Tukey post test.
