# Supplementary material for: Exploring the Antimicrobial Action of Quaternary Amines against Acinetobacter baumannii
Source: mBio. 2018 Feb 6;9(1):e02394-17. doi: 10.1128/mBio.02394-17 (PMC5801471; doi:10.1128/mBio.02394-17)
Supplement: FIG S3 [file mbo001183722sf3.pdf]

Figure S3.

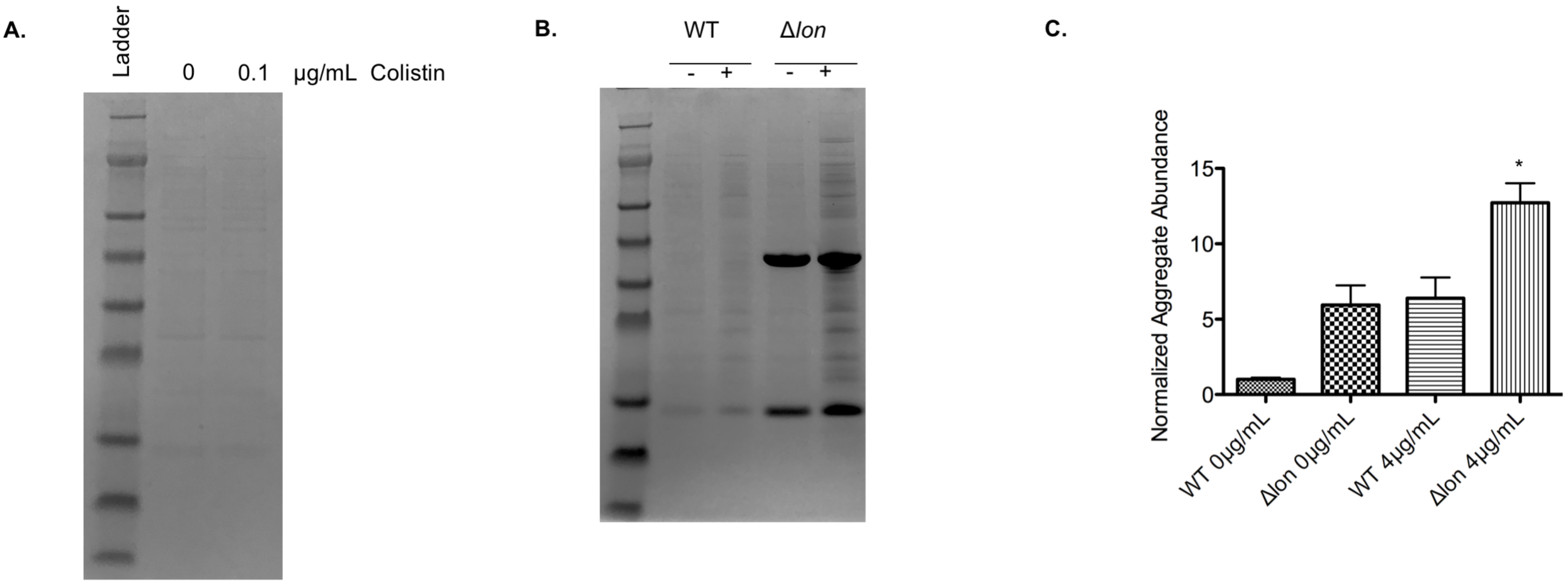

Figure S3. Protein aggregates are not induced by colistin and are more abundant in Δ*lon* mutants than wild type cells. A) Protein aggregates from *A. baumannii* treated with and without sub-lethal colistin. The amount of aggregate loaded was normalized by cell number and was performed in duplicate with a representative image presented here. B) Representative image of protein aggregates from *A. baumannii* wild type (WT) and Δ*lon* mutant following 4µg/ml BZK treatment. 4µg/ml BZK was used due to the increased sensitivity of the Δ*lon* mutant. The amount of aggregate loaded was normalized by cell number. C) Quantitation of the aggregates from quadruplicate biological replicates. The statistical significance of the increase in aggregates between the Δ*lon* mutant and WT when treated with 4µg/ml BZK is noted; $p < 0.05$ One-way ANOVA, Tukey post test.
