# Supplementary material for: Exploring the Antimicrobial Action of Quaternary Amines against Acinetobacter baumannii
Source: mBio. 2018 Feb 6;9(1):e02394-17. doi: 10.1128/mBio.02394-17 (PMC5801471; doi:10.1128/mBio.02394-17)
Supplement: FIG S4 [file mbo001183722sf4.pdf]

**Figure S4**.

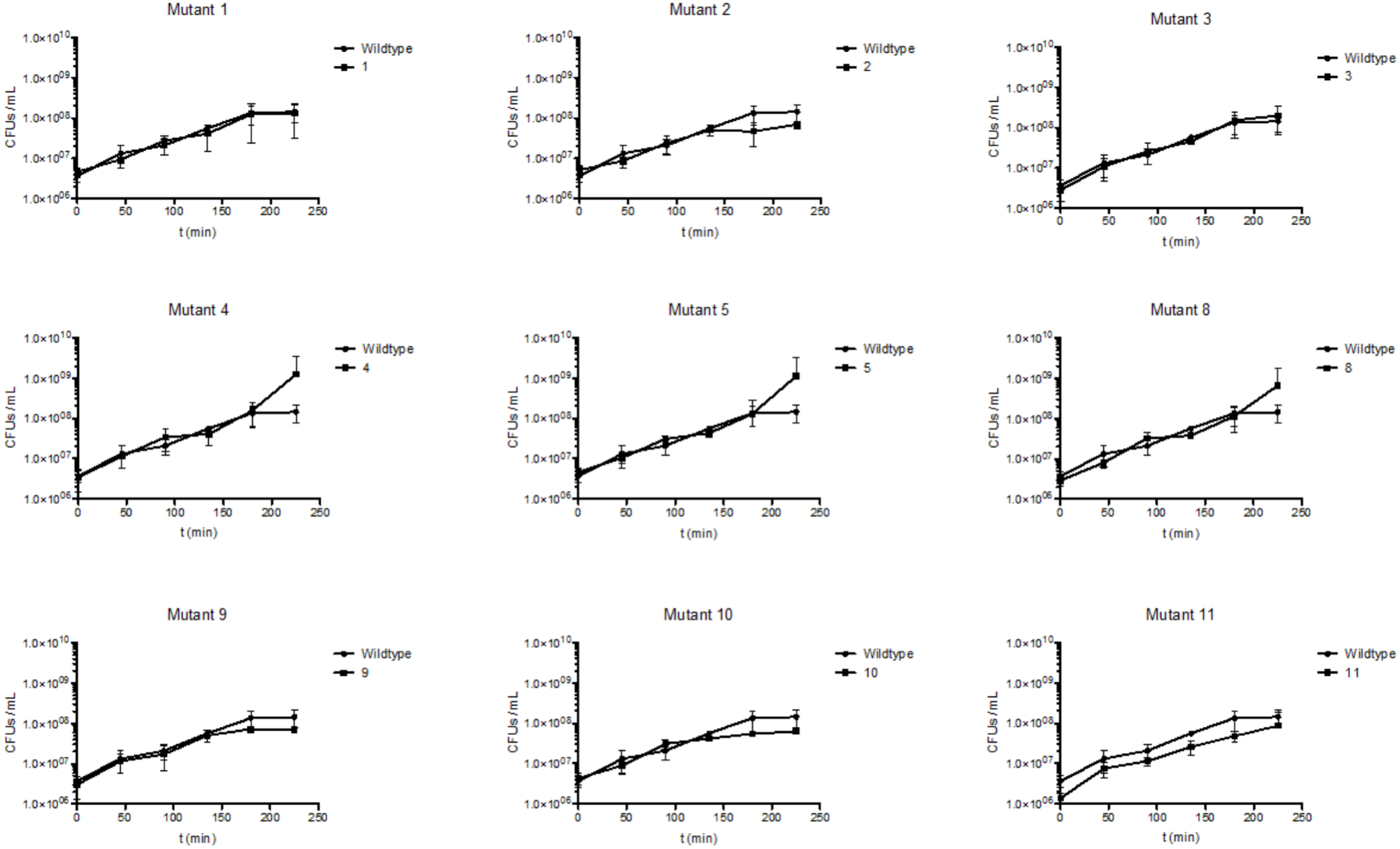


**Figure S4.** Exponential growth of wild type and BZK resistant *A. baumannii* mutants in LB medium measured by CFU. All strains were measured in biological triplicate. Data are represented as mean ± SEM.
