# Supplementary material for: Exploring the Antimicrobial Action of Quaternary Amines against Acinetobacter baumannii
Source: mBio. 2018 Feb 6;9(1):e02394-17. doi: 10.1128/mBio.02394-17 (PMC5801471; doi:10.1128/mBio.02394-17)
Supplement: FIG S5 [file mbo001183722sf5.pdf]

Figure S5.

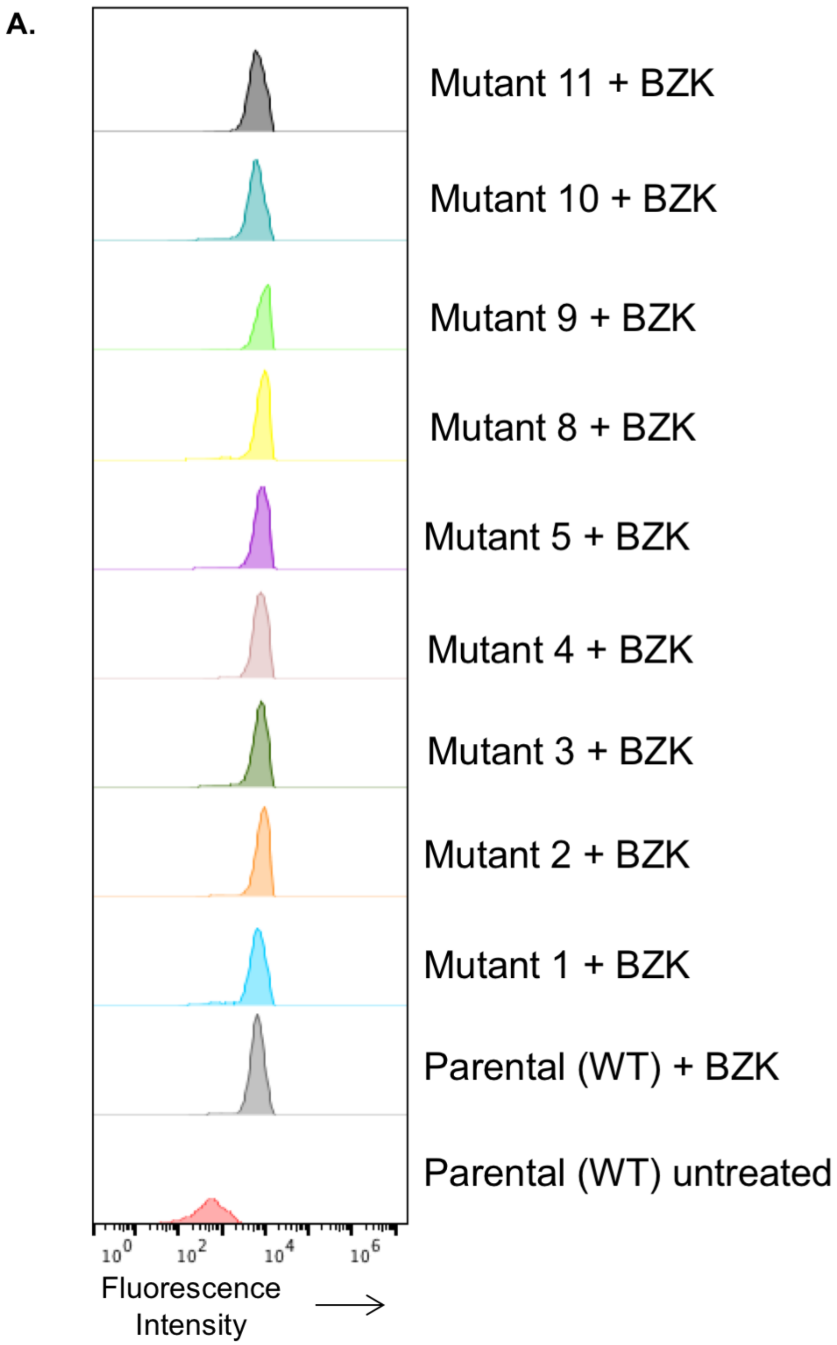

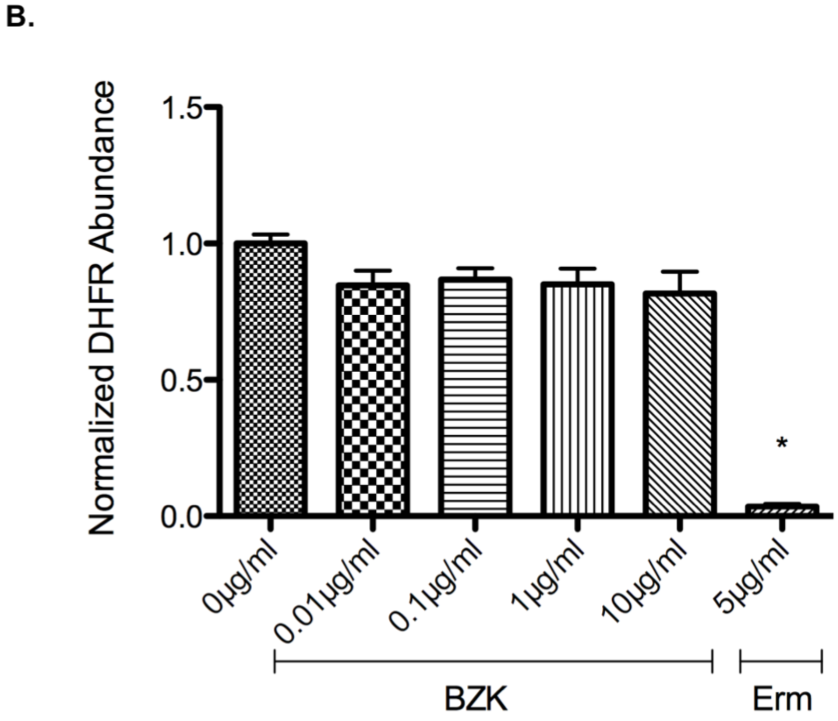

Figure S5. A) Flow cytometry measuring membrane damage caused by BZK against *A. baumannii* parental and BZK resistant mutants measured by cell uptake of propidium iodide. Increased fluorescent intensity indicates increased propidium iodide uptake and membrane damage. An equal number of cells were counted in each condition. B) Effect of BZK and erythromycin (Erm) on *in vitro* translation of DHFR. Only Erm treatment shows a difference in DHFR production * $p < 0.05$ one-way ANOVA with Dunnetts's Multiple Comparison Test to the untreated control.
