# Supplementary material for: Exploring the Antimicrobial Action of Quaternary Amines against Acinetobacter baumannii
Source: mBio. 2018 Feb 6;9(1):e02394-17. doi: 10.1128/mBio.02394-17 (PMC5801471; doi:10.1128/mBio.02394-17)
Supplement: TABLE S4 [file mbo001183722st4.pdf]

Table S4. Results from mass spectrometry analysis of BZK treated and untreated aggregates in biological duplicate. All proteins detected are shown with each samples relative abundance (normalized emPAI value), fold change between the treated and untreated duplicate samples, their p-value determined by T-test, and associated GO term categories. INF denotes infinite increase. X denotes whether a protein is associated with each columns GO term.

																														Normalized emPAI Values			
Identified Proteins (1398)	Accession Number	T-Test (p-value): (p < 0.05)	Fold Change Untreated vs. Treated	Biological Regulation	Cellular Process	Developmental Process	Establishment of Localization	Localization	Metabolic Process	Multi-organisim Process	Reproduction	Reproductive Process	Response to Stimulus	Cytoplasm	Extracellular Region	Intracellular Organelle	Membrane	Organelle Part	Plasma Membrane	Ribosome	Antioxidant Activity	Binding	Catalytic Activity	Electron Carrier Activity	Enzyme Regulator Activity	Molecular Function	Molecular Transducer Activity	Structural Molecule Activity	Transporter Activity	Untreated 1	Untreated 2	Treated 1	Treated 2
Ribonucleoside diphosphate reductase alpha subunit	A3M2P3_ACIBT	0.25	0.9		X				X													X	X			X				8.1355	9.1445	8.0104	7.2808
Elongation factor G	EFG_ACIBT	0.0029	0.1		X				X					X								X	X			X				21.582	20.394	1.5259	3.1421
DNA-directed RNA polymerase subunit beta	RPOB_ACIBT	0.083	1.4		X				X													X	X			X				2.1225	1.8349	3.002	2.5889
DNA-directed RNA polymerase subunit beta'	RPOC_ACIBT	0.092	1.2		X				X													X	X			X				1.6973	1.8336	2.1283	1.9926
Cell division protein FtsZ	A3M9X6_ACIBT	0.031	3		X						X	X		X								X	X			X				56.193	49.154	178.83	140.92
Trigger factor	TIG_ACIBT	0.85	1.1		X		X	X	X					X									X			X				59.279	27.252	60.096	34.969
30S ribosomal protein S1	A3M505_ACIBT	0.81	0.9		X				X					X		X				X		X				X		X		15.752	19.769	20.359	12.838
Phosphoenolpyruvate carboxykinase [GTP]	PCKG_ACIBT	0.98	1						X					X								X	X			X				5.77	11.009	7.448	9.5063
Outer membrane protein Omp38	OMP38_ACIBT	0.33	1.1		X		X	X									X									X			X	19.573	16.724	19.311	22.204
Uncharacterized protein	A3M3J3_ACIBT	0.17	1.3						X								X						X			X				2.212	1.7177	2.5475	2.4504
Isocitrate dehydrogenase	A3M7K1_ACIBT	0.0019	0.02		X				X													X	X			X				14.782	16.115	0.29533	0.23959
Putative transport protein	A3M9L8_ACIBT	0.11	2.5						X													X	X			X				4.2457	5.8479	10.091	15.298
Protein translocase subunit SecA	SECA_ACIBT	0.0032	0.3				X	X						X			X		X			X				X				4.2916	4.6649	1.1733	1.129
Chaperone protein ClpB	A3M3X2_ACIBT	0.0087	0.2						X				X	X								X	X			X				3.9504	4.521	0.8929	0.55995
Phosphoribosylformylglycinamidine synthase	A3M7V8_ACIBT	0.0039	0.1		X				X					X								X	X			X				3.1304	3.4894	0.45929	0.45684
Urocanase	A3MA52_ACIBT	0.73	1.1						X														X			X				14.246	9.645	17.624	9.8412
ATP-dependent zinc metalloprotease FtsH	A3M850_ACIBT	0.02	0.6		X				X								X		X			X	X			X				6.4827	5.9735	3.8087	3.2213
ATP synthase subunit beta	ATPB_ACIBT	0.33	1.5		X		X	X	X								X		X			X	X			X			X	7.9053	12.219	18.609	11.798
Alanine--tRNA ligase	SYA_ACIBT	0.01	0.1		X				X					X								X	X			X				4.0318	4.8242	0.54038	0.50493
Uncharacterized protein	A3M0X6_ACIBT	0.28	1.8																											1.6034	1.5637	3.7577	2.0097
2-oxoglutarate decarboxylase component of the 2-oxoglutarate dehydrogenase complex (E1)	A3M884_ACIBT	0.00092	0.2		X				X													X	X			X				3.8486	4.0042	0.62945	0.74979
Glycerol-3-phosphate acyltransferase	A3M994_ACIBT	0.019	1.8		X				X								X		X				X			X				1.4246	1.6812	2.6717	2.8963
DNA gyrase subunit B	A3M0Q7_ACIBT	0.49	1.2		X				X					X		X						X	X			X				1.8682	1.6062	2.5746	1.6784
Chromosome partition protein Smc	A3M2U1_ACIBT	0.011	11		X				X					X		X						X				X				0.15495	0.16121	1.9737	1.6301
Aconitate hydratase 2	A3M6K9_ACIBT	0.006	0.4						X													X	X			X				115.24	105.08	44.632	43.119
RNA polymerase sigma factor RpoD	A3M875_ACIBT	0.14	1.5	X	X				X					X								X				X				2.6565	1.7575	3.3743	3.2282
Protein tyrosine kinase	A3M0V0_ACIBT	0.7	1		X				X								X						X			X				2.4373	2.3782	2.5557	2.3547
Elongation factor Tu	EFTU_ACIBT	0.4	1.4		X				X					X								X	X			X				10.813	7.7448	15.855	9.8526
Outer membrane protein assembly factor BamA	A3M652_ACIBT	0.099	1.7		X		X	X									X													1.0298	1.2419	2.1468	1.6654
DNA topoisomerase 1	A3M1V0_ACIBT	0.012	0.3		X				X							X						X	X			X				2.3863	2.3529	0.86304	0.49604
Transport protein Uup	A3M1U4_ACIBT	0.00017	3.2						X													X	X			X				0.88355	0.91926	2.9396	2.9004
Elongation factor Ts	EFTS_ACIBT	0.04	0.06		X				X					X								X				X				67.313	99.605	3.9859	5.3842
Aminopeptidase N	A3M6H5_ACIBT	0.00013	0.3						X													X	X			X				2.6399	2.6074	0.86064	0.83744
Phenylalanine--tRNA ligase beta subunit	A3M2A9_ACIBT	0.015	0.05		X				X					X								X	X			X				5.1398	6.5015	0.35349	0.22368
Transcription termination/antitermination protein NusA	A3M1K3_ACIBT	0.48	1.1	X																		X				X				5.082	4.1526	4.9478	5.0933

Identified Proteins (1398)	Accession Number	T-Test (p-value): (p < 0.05)	Fold Change Untreated vs. Treated	Biological Regulation	Cellular Process	Developmental Process	Establishment of Localization	Localization	Metabolic Process	Multi-organisim Process	Reproduction	Reproductive Process	Response to Stimulus	Cytoplasm	Extracellular Region	Intracellular Organelle	Membrane	Organelle Part	Plasma Membrane	Ribosome	Antioxidant Activity	Binding	Catalytic Activity	Electron Carrier Activity	Enzyme Regulator Activity	Molecular Function	Molecular Transducer Activity	Structural Molecule Activity	Transporter Activity	Normalized emPAI Values			
																														Untreated 1	Untreated 2	Treated 1	Treated 2
Ribonuclease E	A3M1R6_ACIBT	0.0045	0.2	X	X				X					X			X		X			X	X			X				1.7989	1.7028	0.46617	0.31014
Uncharacterized protein	A3M8C2_ACIBT	0.41	1.3				X	X									X									X			X	6.5065	4.2888	8.7319	5.831
GTP-binding elongation factor family protein	A3M8J7_ACIBT	0.18	2		X				X													X	X			X				2.0056	2.2609	3.1864	5.2776
Pyruvate dehydrogenase E1 component	A3M9X3_ACIBT	0.022	0.3						X														X			X				1.8993	1.5691	0.43646	0.59941
60 kDa chaperonin	CH60_ACIBT	0.49	0.8		X				X					X								X				X				3.712	3.2762	3.5582	2.357
Glycosyltransferase	A3M0V9_ACIBT	0.33	2						X														X			X				7.9504	4.2757	16.597	7.8362
DNA polymerase I	A3M2B7_ACIBT	0.0093	0.5		X				X				X									X	X			X				1.465	1.3478	0.64448	0.72212
Uncharacterized protein	A3M8T9_ACIBT	0.061	1.6		X				X				X									X	X			X				0.86677	0.9736	1.5856	1.3298
IcmB protein	A3M2F1_ACIBT	0.016	2																											0.40235	0.37473	0.7193	0.81069
Putative ATP-dependent RNA helicase	A3M9A7_ACIBT	0.0052	2.5						X													X	X			X				0.91368	0.9506	2.3966	2.2023
Uncharacterized protein	A3M0R8_ACIBT	0.15	0.6		X				X													X	X			X				4.4897	3.6658	3.0306	1.8889
Aconitate hydratase 1	A3M0X5_ACIBT	0.11	0.6						X																					1.4691	1.246	0.98588	0.65595
NADH dehydrogenase I chain G	A3M2Q3_ACIBT	0.04	1.6		X				X								X					X	X	X		X				1.321	1.0351	1.9371	1.8595
Putative cell division protein (FstK)	A3M314_ACIBT	0.071	0.8		X												X					X				X				0.89354	0.80285	0.65973	0.69324
GTP pyrophosphokinase	A3M286_ACIBT	0.022	0.4		X				X													X	X			X				1.6449	1.491	0.68744	0.42384
AdeK	A3M8A6_ACIBT	0.021	2.3				X	X									X					X				X			X	3.9918	2.9017	7.5299	8.2256
Threonine--tRNA ligase	SYT_ACIBT	0.0074	0.2		X				X					X								X	X			X				4.861	4.2118	0.74709	0.80332
Fumarate hydratase	A3M1Z1_ACIBT	0.018	0.3		X				X														X			X				6.4375	7.7862	2.0703	1.6484
Chaperone protein DnaK	DNAK_ACIBT	0.049	0.4		X																	X				X				4.4996	3.3602	1.4906	1.4288
Rod shape-determining protein	A3M8E9_ACIBT	0.13	17		X	X																								1.7364	2.4306	22.051	47.825
Uncharacterized protein	A3M1H8_ACIBT	0.016	8.2																											0.10264	0.07009	0.62744	0.78243
CTP synthase	PYRG_ACIBT	0.27	0.8		X				X													X	X			X				2.8083	2.9218	2.6706	1.9323
Bifunctional uridylyltransferase/uridylyl-removing enzyme	GLND_ACIBT	0.043	1.7	X					X													X	X			X				0.55894	0.41221	0.8196	0.84335
Outer membrane protein CarO	A3M7R1_ACIBT	0.28	5														X													14.444	23.034	42.952	143.97
Ribonucleoside-diphosphate reductase beta subunit	A3M2P2_ACIBT	0.81	0.9		X				X													X	X			X				3.1983	3.0145	3.8385	1.8397
Putative serine protease	A3M7P8_ACIBT	0.2	1.2						X														X			X				5.4976	5.7198	6.1702	7.3607
Uncharacterized protein	A7FAS8_ACIBT	0.26	1.6																											1.0187	1.8498	2.6071	1.9186
Uncharacterized protein	A3M9H5_ACIBT	0.16	2.5		X				X																					0.76212	0.88057	2.6451	1.5061
Catalase-peroxidase	KATG_ACIBT	0.019	0.4		X				X				X								X	X	X			X				2.7644	2.3644	1.1379	1.0093
ATP-binding protease component	A3M5Z3_ACIBT	0.038	0.3						X													X	X			X				2.6101	1.9787	0.45458	0.72014
Phosphoenolpyruvate synthase	A3M6P7_ACIBT	0.054	0.5		X				X													X	X			X				1.5846	2.0146	0.80003	0.93774
Putative RNA binding protein	A3M9M3_ACIBT	0.017	0.7		X				X													X				X				3.2377	3.1148	1.9605	2.22
Transcription-repair-coupling factor	A3M556_ACIBT	0.014	0.7	X	X				X				X	X								X	X			X				0.29881	0.31089	0.22988	0.21389
Ribonuclease R	A3M948_ACIBT	0.017	0.2		X				X					X								X	X			X				1.1847	1.4248	0.26565	0.36591
NADH-quinone oxidoreductase subunit C/D	NUOCD_ACIBT	0.42	1.2				X	X	X								X		X			X	X			X				1.0345	1.0763	1.517	1.0558
Uncharacterized protein	A3M6L3_ACIBT	0.21	1.8																											0.23821	0.37052	0.64615	0.42846
Succinate dehydrogenase flavoprotein subunit	A3M882_ACIBT	0.01	0.3		X				X													X	X			X				1.7224	1.9517	0.47719	0.60728
Chaperone protein DnaJ	DNAJ_ACIBT	0.96	1		X				X				X	X								X				X				4.6032	3.0357	4.2786	3.255
30S ribosomal protein S8	RS8_ACIBT	0.14	2.8		X				X					X		X				X		X				X		X		50.131	14.732	73.51	108.68
ATP-dependent DNA helicase Rep	A3M323_ACIBT	0.0087	2		X				X					X								X	X			X				0.54584	0.64689	1.1505	1.1867
Aconitate hydratase 2	A3M6L1_ACIBT	0.56	0.8		X				X														X			X				22.436	11.351	13.783	12.363
DNA-directed RNA polymerase subunit beta'	RPOC_ACIBT-DECOY	1	INF																											0	0	0	0
Peptidyl-prolyl cis-trans isomerase	A3M6J2_ACIBT	0.73	0.8		X				X														X			X				36.727	46.358	20.056	50.291

Identified Proteins (1398)	Accession Number	T-Test (p-value): ($p < 0.05$)	Fold Change Untreated vs. Treated	Biological Regulation	Cellular Process	Developmental Process	Establishment of Localization	Localization	Metabolic Process	Multi-organisim Process	Reproduction	Reproductive Process	Response to Stimulus	Cytoplasm	Extracellular Region	Intracellular Organelle	Membrane	Organelle Part	Plasma Membrane	Ribosome	Antioxidant Activity	Binding	Catalytic Activity	Electron Carrier Activity	Enzyme Regulator Activity	Molecular Function	Molecular Transducer Activity	Structural Molecule Activity	Transporter Activity	Normalized emPAI Values			
																														Untreated 1	Untreated 2	Treated 1	Treated 2
UvrABC system protein B	A3M7M8_ACIBT	0.082	0.6		X				X				X	X								X	X			X				0.79187	1.0119	0.49981	0.55883
Sulfate adenylyltransferase subunit 1	A3M3D9_ACIBT	0.047	0.2		X				X													X	X			X				6.3158	4.562	0.81968	1.5876
Putative metalloprotease	A3M603_ACIBT	0.032	0.6						X													X	X			X				0.78881	0.69873	0.4648	0.39101
Carbamoyl-phosphate synthase (glutamine-hydrolyzing)	A3M856_ACIBT	0.69	0.8		X				X													X	X			X				0.13724	0.39256	0.20125	0.21219
Uncharacterized protein	A3M9B3_ACIBT	0.062	2.4																											0.9103	0.78432	1.7167	2.312
ATP-dependent helicase	A3M3Z2_ACIBT	0.22	0.6						X													X	X			X				0.15182	0.24527	0.10757	0.12259
Glutamate synthase large chain	A3M9I6_ACIBT	0.017	8.9		X				X														X			X				0	0.02471	0.10683	0.11294
ATP synthase subunit alpha	ATPA_ACIBT	0.31	0.8		X		X	X	X								X		X			X	X			X			X	2.8026	3.7776	2.5435	2.7174
D-amino acid dehydrogenase	DADA_ACIBT	0.00052	2.2		X				X														X			X				2.561	2.6646	5.6681	5.578
GTPase Obg	OBG_ACIBT	0.75	1.1						X					X								X	X			X				3.4448	1.7891	2.8629	2.9689
Lysine--tRNA ligase	SYK_ACIBT	0.035	0.2		X				X					X								X	X			X				2.1524	2.919	0.55976	0.49809
Uncharacterized protein	A3M4W1_ACIBT	0.03	0.3	X	X				X														X			X				0.72686	0.57173	0.12652	0.20069
DNA-directed DNA polymerase	A3M4P9_ACIBT	0.0056	0.5		X				X					X								X	X			X				0.48758	0.50729	0.2248	0.25785
30S ribosomal protein S3	RS3_ACIBT	0.081	2.4		X				X					X		X		X		X		X				X		X		7.0036	2.8135	11.871	11.786
Uncharacterized protein	A3M303_ACIBT	0.058	7																											1.1281	1.7275	12.165	7.8721
Putative protease	A3M7J4_ACIBT	0.021	0.3						X													X	X			X				0.84975	0.68787	0.16998	0.2147
Carboxy-terminal protease	A3M202_ACIBT	0.033	0.8						X														X			X				0.91739	0.95446	0.76599	0.69962
Uncharacterized protein	A3M2D4_ACIBT	0.54	0.8		X				X													X	X			X				0.11714	0.17433	0.10088	0.13979
Site-determining protein	A3M318_ACIBT	0.1	0.2		X																	X	X			X				15.001	25.726	4.8553	4.9707
24-dienoyl-CoA reductase	A3M3K7_ACIBT	0.09	0.6						X													X	X			X				0.35776	0.30239	0.15814	0.22716
Glycine--tRNA ligase beta subunit	SYGB_ACIBT	0.0018	0.04		X				X					X								X	X			X				4.3597	4.0191	0.1562	0.14211
Aconitate hydratase	A3M267_ACIBT	0.033	1.4						X														X			X				0.63248	0.65804	0.83963	0.92458
Putative outer membrane protein	A3M6L5_ACIBT	0.048	1.5														X													0.56117	0.46846	0.82288	0.74323
Protein RecA	RECA_ACIBT	0.18	0.5		X				X				X	X								X	X			X				5.8919	7.6293	4.8117	1.8359
Glyceraldehyde-3-phosphate dehydrogenase	A3M7M5_ACIBT	0.4	0.8						X													X	X			X				2.527	3.4714	2.7372	1.9355
50S ribosomal protein L5	RL5_ACIBT	0.0057	3		X				X					X		X				X		X				X		X		7.5859	6.4471	19.89	21.629
Ubiquinone/menaquinone biosynthesis C-methyltransferase UbiE	A3M1L6_ACIBT	0.4	0.8		X				X														X			X				7.2425	7.5352	4.364	7.2892
DNA primase	A3M2D7_ACIBT	0.47	0.4																											0.02701	0.11678	0	0.05277
Chaperone protein	A3M7N4_ACIBT	0.052	2.1																			X				X				1.4128	1.3285	2.5059	3.2099
Cell division protein ZipA	A3M2U2_ACIBT	0.28	1.5		X												X		X											4.6429	7.5266	7.629	10.47
Isocitrate lyase	A3M3E6_ACIBT	0.021	0.06		X				X														X			X				2.9399	3.8864	0.19902	0.18345
Aspartyl/glutamyl-tRNA(Asn/Gln) amidotransferase subunit B	A3M8F2_ACIBT	0.0013	0.1		X				X													X	X			X				1.4592	1.5182	0.2211	0.14863
Uncharacterized protein	A3M2D2_ACIBT	0.076	3																											1.1547	1.0519	4.0008	2.6922
Putative membrane protein	A3M3G0_ACIBT	0.1	5																											0.07675	0.07985	0.50173	0.2818
Glutamine--tRNA ligase	A3M6J1_ACIBT	0.074	0.2		X				X					X								X	X			X				1.791	2.8033	0.60646	0.37131
Inosine-5'-monophosphate dehydrogenase	A3M9W6_ACIBT	0.14	0.4		X				X													X	X			X				2.6501	4.3671	1.5615	1.3762
Acetyl-coenzyme A carboxylase carboxyl transferase subunit alpha	ACCA_ACIBT	0.38	0.7		X				X					X								X	X			X				6.1022	4.7637	2.9942	5.1037
Putative outer membrane protein	A3M322_ACIBT	0.082	0.4														X		X											32.191	24.178	8.6104	14.756
GMP synthase [glutamine-hydrolyzing]	A3M121_ACIBT	0.025	0.1		X				X													X	X			X				2.8245	3.7641	0.20422	0.43018
Uncharacterized protein	A3M2N2_ACIBT	0.96	1																											4.6429	3.8174	5.3803	2.9481
Outer membrane protein assembly factor BamD	A3M2Y0_ACIBT	0.095	2.4		X		X	X									X													2.0667	0.87084	3.8621	3.1948
30S ribosomal protein S7	RS7_ACIBT	0.33	0.8		X				X					X		X		X		X		X				X		X		8.1957	10.627	7.5931	8.0476
Response regulator protein	A3M661_ACIBT	0.9	1	X	X				X				X									X				X				1.6978	1.589	1.5786	1.7366

Identified Proteins (1398)	Accession Number	T-Test (p-value): (p < 0.05)	Fold Change Untreated vs. Treated	Biological Regulation	Cellular Process	Developmental Process	Establishment of Localization	Localization	Metabolic Process	Multi-organisim Process	Reproduction	Reproductive Process	Response to Stimulus	Cytoplasm	Extracellular Region	Intracellular Organelle	Membrane	Organelle Part	Plasma Membrane	Ribosome	Antioxidant Activity	Binding	Catalytic Activity	Electron Carrier Activity	Enzyme Regulator Activity	Molecular Function	Molecular Transducer Activity	Structural Molecule Activity	Transporter Activity	Normalized emPAI Values			
																														Untreated 1	Untreated 2	Treated 1	Treated 2
Polyribonucleotide nucleotidyltransferase	PNP_ACIBT	0.02	0.3	X	X				X					X								X	X			X				1.6982	2.0552	0.41919	0.5797
Uncharacterized protein	A7FAW6_ACIBT	0.63	1.2																			X				X				2.0254	2.8733	2.0985	3.9401
Elongation factor 4	LEPA_ACIBT	0.5	1.3	X	X				X								X		X			X	X			X				0.96282	1.0017	1.5761	0.92056
Putative glycosyltransferase	A3M0W0_ACIBT	0.17	1.5						X														X			X				3.9519	2.8598	4.5657	5.9468
Acyl coenzyme A reductase	A3M9M1_ACIBT	0.045	5						X														X			X				2.4277	2.153	9.3744	13.339
UvrABC system protein A	A3M9U0_ACIBT	0.15	1.3		X				X				X	X								X	X			X				0.28951	0.30121	0.42453	0.34529
Phosphoenolpyruvate carboxylase	CAPP_ACIBT	0.12	0.4		X				X													X	X			X				0.50276	0.76984	0.23551	0.31923
50S ribosomal protein L4	RL4_ACIBT	0.27	2.5		X				X					X		X				X		X				X		X		7.7323	5.5003	23.193	10.043
Malate synthase G	A3M534_ACIBT	0.015	0.06		X				X					X								X	X			X				1.5594	1.2762	0	0.17058
Putative VGR-related protein	A3M259_ACIBT	0.17	3.2																											0.29656	0.25965	1.1783	0.59679
Phosphoglycerate kinase	PGK_ACIBT	0.14	0.2		X				X					X								X	X			X				4.4063	9.3162	0.83734	1.2222
tRNA 2-thiocytidine biosynthesis protein TtcA	TTCA_ACIBT	0.0098	3.4		X				X					X								X				X				1.6058	1.9704	5.6829	6.4561
Pantothenate synthetase	PANC_ACIBT	0.012	0.8		X				X					X								X	X			X				6.3026	6.5573	5.284	5.2014
Aspartate--tRNA(Asp/Asn) ligase	SYDND_ACIBT	0.12	0.5		X				X					X								X	X			X				0.85666	1.2414	0.59243	0.41959
Putative hydrolase	A3M1Z9_ACIBT-DECOY	1	INF																											0	0	0	0
DNA mismatch repair enzyme	A3M2C8_ACIBT	0.17	2.7																											0.38145	0.60823	0.96562	1.7366
DNA-binding ATP-dependent protease La	A3M3G8_ACIBT	0.11	0.1						X													X	X			X				2.9853	5.9138	0.4508	0.44732
Uncharacterized protein	A3M8R9_ACIBT	0.016	9.7	X																		X				X				0.02623	0.02729	0.29015	0.22958
Elongation factor G	EFG_ACIBT-DECOY	1	INF																											0	0	0	0
Uncharacterized protein	A7FAR9_ACIBT	0.087	3.2																											1.4015	0.72495	2.7186	4.0113
Enolase	ENO_ACIBT	0.16	0.4		X				X					X	X							X	X			X				2.8869	4.4951	2.0828	1.0286
UPF0313 protein A1S_0183	A3M170_ACIBT	0.14	4.2						X													X	X			X				0.04344	0.09225	0.19906	0.37427
Putative transcriptional regulator (AraC family)	A3M1T3_ACIBT	0.42	1.5	X	X				X													X				X				3.9959	2.8884	6.5742	3.5583
DNA gyrase subunit A	A3M7Z9_ACIBT	0.0034	0.5		X				X					X		X						X	X			X				0.6975	0.66078	0.3074	0.32822
Uncharacterized protein	A7FAV5_ACIBT	0.93	1																											1.5097	1.3945	1.3143	1.622
Histidine--tRNA ligase	SYH_ACIBT	0.11	0.6		X				X					X								X	X			X				1.3269	1.571	1.0505	0.73462
2,3-bisphosphoglycerate-independent phosphoglycerate mutase	A3M1B1_ACIBT	0.0015	0.2		X				X					X								X	X			X				1.5228	1.5843	0.32637	0.25175
UDP-N-acetylglucosamine 1-carboxyvinyltransferase	A3M2I5_ACIBT	0.29	1.3	X	X				X					X									X			X				1.4937	2.2753	2.5023	2.4041
NADH dehydrogenase I chain F	A3M2Q2_ACIBT	0.32	1.7						X													X	X			X				1.1352	1.3538	1.4383	2.6718
Uncharacterized protein	A7FAV7_ACIBT	0.66	1.2																											0.47577	0.91221	0.69765	0.95181
Thermoresistant gluconokinase	A3M1Z7_ACIBT	0.057	3.9		X				X														X			X				3.5841	2.9132	10.308	14.922
Putative transcriptional regulator	A3M7F5_ACIBT	0.48	1.2	X	X				X													X				X				1.9034	2.6854	2.366	3.1967
RNA polymerase-associated protein RapA	A3M7I6_ACIBT	0.018	0.2	X	X				X													X	X			X				0.81104	0.65793	0.16377	0.17729
Valine--tRNA ligase	A3M8B1_ACIBT	0.013	0.2	X	X				X					X								X	X			X				1.144	1.0109	0.27424	0.11875
Protein translocase subunit SecD	A3M8S5_ACIBT	0.026	2				X	X									X		X							X			X	0.71926	0.56603	1.1935	1.326
Glutamate-ammonia-ligase adenylyltransferase	A3M8R7_ACIBT	0.13	0.5						X													X	X			X				0.24373	0.35052	0.16942	0.15476
Uncharacterized protein	A3M987_ACIBT	0.24	0.6																											1.6775	2.3414	1.5616	0.7644
UDP-N-acetylmuramoylalanine--D-glutamate ligase	MURD_ACIBT	0.041	0.5	X	X				X					X								X	X			X				3.6622	3.1275	1.9784	1.55
50S ribosomal protein L1	RL1_ACIBT	0.52	0.8	X	X				X					X		X		X		X		X				X		X		8.6087	6.3855	7.5517	4.6238
Putative very-long-chain acyl-CoA synthetase	A3M1R5_ACIBT	0.14	0.8		X				X														X			X				0.64081	0.66671	0.57577	0.46521
Pseudouridine synthase	A3M2V4_ACIBT	0.031	0.3		X				X													X	X			X				2.8457	3.3902	0.54482	1.1617
Catalase	A3M4H0_ACIBT	0.0035	1.8						X				X								X	X	X			X				0.45863	0.47717	0.88214	0.83934
Chromosome partitioning protein	A3M4Y5_ACIBT	0.11	2.2																			X				X				2.3607	3.7986	7.9606	5.642

Identified Proteins (1398)	Accession Number	T-Test (p-value): (p < 0.05)	Fold Change Untreated vs. Treated	Biological Regulation	Cellular Process	Developmental Process	Establishment of Localization	Localization	Metabolic Process	Multi-organisim Process	Reproduction	Reproductive Process	Response to Stimulus	Cytoplasm	Extracellular Region	Intracellular Organelle	Membrane	Organelle Part	Plasma Membrane	Ribosome	Antioxidant Activity	Binding	Catalytic Activity	Electron Carrier Activity	Enzyme Regulator Activity	Molecular Function	Molecular Transducer Activity	Structural Molecule Activity	Transporter Activity	Normalized emPAI Values			
																														Untreated 1	Untreated 2	Treated 1	Treated 2
UPF0176 protein A1S_2708	A3M877_ACIBT	0.24	1.5																											2.5256	1.9412	2.7359	3.9683
DNA topoisomerase 4 subunit B	A3MA04_ACIBT	0.92	1		X				X							X						X	X			X				0.81734	0.65742	0.80087	0.64969
Chromosomal replication initiator protein DnaA	DNAA_ACIBT	0.56	1.2	X	X				X					X								X				X				1.0403	1.5807	1.3217	1.7975
Secretion protein XcpR	A3M2C1_ACIBT	0.5	1.2		X		X	X														X				X			X	0.40758	0.75357	0.74247	0.70457
GTPase Era	A3M7P1_ACIBT	0.044	0.7						X					X			X		X			X	X			X				1.362	1.1958	0.90487	0.86485
Putative oxidoreductase molybdopterin	A3M813_ACIBT	0.0084	4.8						X													X	X			X				0.23647	0.14161	0.86696	0.96131
Putative transport protein	A3M739_ACIBT	0.29	6.2						X													X	X			X				0.11271	0	0.55068	0.15078
Succinyl-CoA ligase [ADP-forming] subunit alpha	A3M888_ACIBT	0.17	0.6						X													X	X			X				5.6798	7.7368	4.6656	2.7219
Oligopeptidase A	A3M950_ACIBT	0.036	0.3						X														X			X				3.2659	2.5006	0.86752	0.96207
Putative general secretion pathway protein	A3M1F2_ACIBT	0.16	1.5		X		X	X									X									X			X	0.52151	0.47099	0.87025	0.63856
Two-component regulatory activator (OmpR family)	A3M2P4_ACIBT	0.021	0.4	X	X				X				X									X				X				7.4732	6.6887	3.4646	2.5414
Putative oxidoreductase	A3M7I3_ACIBT	0.024	0.4						X													X	X	X		X				4.3092	4.4833	2.2709	1.4981
50S ribosomal protein L2	RL2_ACIBT	0.68	0.8		X				X					X		X		X		X		X	X			X		X		1.3394	2.882	1.9641	1.4905
Phosphoenolpyruvate-protein phosphotransferase	A3M1S4_ACIBT	0.029	0.4		X		X	X	X													X	X			X				0.69485	0.57568	0.2842	0.22999
Uncharacterized protein	A3M474_ACIBT	0.42	INF																											0	0	0	0.09187
Zn-dependent oligopeptidase	A3M543_ACIBT	0.11	3.6						X														X			X				0.05032	0.05235	0.23208	0.13724
Uncharacterized protein	A7FB81_ACIBT	0.12	2.9																											0.65045	0.55907	2.2278	1.3383
50S ribosomal protein L14	RL14_ACIBT	0.47	1.5		X				X					X		X		X		X		X				X		X		12.304	9.6849	23.705	10.265
WecC protein	A3M0V3_ACIBT	0.2	0.6						X													X	X			X				3.0959	2.0875	1.764	1.4277
Putative helicase	A3M6I0_ACIBT	0.46	1.3	X	X				X				X										X			X				0.505	0.52541	0.49862	0.87906
Succinate dehydrogenase iron-sulfur subunit	A3M883_ACIBT	0.0026	1.4		X				X													X	X	X		X				2.4116	2.5091	3.5363	3.5933
Phospho-2-dehydro-3-deoxyheptonate aldolase	A3M597_ACIBT	0.0015	0.04		X				X														X			X				4.1245	3.8352	0.14295	0.20339
Aspartate ammonia-lyase (Aspartase)	A3M5F9_ACIBT	0.16	0.5		X				X														X			X				1.5637	1.4428	0.49928	1.1133
Aconitate hydratase 2	A3M6L0_ACIBT	0.77	0.9						X													X				X				5.7095	6.9394	3.5608	7.6716
Acyl-CoA dehydrogenase A	A3M8P9_ACIBT	0.28	0.7						X													X	X			X				0.7845	0.52218	0.38525	0.49813
Malate dehydrogenase	MDH_ACIBT	0.015	0.2		X				X														X			X				5.4316	4.4177	0.76902	0.83162
Uncharacterized protein	A3M1L9_ACIBT	0.1	29																											1.607	0.81496	23.316	46.689
Hemolysin-type calcium-binding region	A3M3K9_ACIBT	0.018	INF						X	X					X		X					X	X			X				0	0	0.01593	0.02091
30S ribosomal protein S2	A3M751_ACIBT	0.3	2.1		X				X					X		X		X		X						X		X		15.087	29.696	30.504	62.516
Uncharacterized protein	A3M796_ACIBT	0.0078	0.4						X														X			X				3.8043	3.958	1.6421	1.2348
Lipoprotein	A3M7F6_ACIBT	0.38	0.8																											2.8793	4.1026	2.9942	2.5038
Uncharacterized protein	A3M7T2_ACIBT	0.64	0.9																											1.2927	1.3449	1.3865	1.0794
GTPase Der	DER_ACIBT	0.19	0.5																			X				X				1.3322	1.9486	1.1193	0.56596
tRNA uridine 5-carboxymethylaminomethyl modification enzyme MnmG	MNMG_ACIBT	0.054	0.1		X				X					X								X				X				1.6424	1.0645	0.08281	0.19955
30S ribosomal protein S4	RS4_ACIBT	0.19	1.7		X				X					X		X		X		X		X				X		X		2.477	4.684	5.459	6.8401
Anthranilate synthase component I TrpE	A3M1F5_ACIBT	0.12	1.7		X				X														X			X				0.07093	0.0738	0.10401	0.14441
Acetolactate synthase III large subunit	A3M252_ACIBT	0.092	0.5		X				X													X	X			X				0.61276	0.84802	0.28982	0.38908
Chromosome partitioning protein	A3M4Y4_ACIBT	0.03	5.4																											1.4951	2.3166	11.65	8.7943
Uncharacterized protein	A3M6P5_ACIBT	0.34	2.5				X	X																		X			X	0.04116	0.13357	0.12308	0.31242
Peptidyl-prolyl cis-trans isomerase	A3M0U9_ACIBT	0.15	1.9		X				X														X			X				4.3787	6.3318	12.214	8.3076
Copper resistance protein A	A3M8U7_ACIBT	0.11	1.5						X													X	X			X				0.22784	0.30392	0.42834	0.37022
Polyphosphate kinase	PPK_ACIBT	0.0092	3		X				X													X	X			X				0.15474	0.10486	0.39638	0.38728
50S ribosomal protein L3	RL3_ACIBT	0.63	1.3		X				X					X		X				X		X				X		X		5.872	3.4043	8.6104	3.7285

Identified Proteins (1398)	Accession Number	T-Test (p-value): (p < 0.05)	Fold Change Untreated vs. Treated	Biological Regulation	Cellular Process	Developmental Process	Establishment of Localization	Localization	Metabolic Process	Multi-organisim Process	Reproduction	Reproductive Process	Response to Stimulus	Cytoplasm	Extracellular Region	Intracellular Organelle	Membrane	Organelle Part	Plasma Membrane	Ribosome	Antioxidant Activity	Binding	Catalytic Activity	Electron Carrier Activity	Enzyme Regulator Activity	Molecular Function	Molecular Transducer Activity	Structural Molecule Activity	Transporter Activity	Normalized emPAI Values			
																														Untreated 1	Untreated 2	Treated 1	Treated 2
Beta-hydroxylase	A3M1I0_ACIBT	0.11	1.6		X				X																					1.06	1.5867	2.2363	2.0227
Putative membrane protein	A3M486_ACIBT	0.0086	3.1																											0.03272	0.03404	0.09745	0.11054
Putative ATPase	A3M512_ACIBT	0.0059	0.4																			X				X				2.2434	2.046	0.85116	0.80486
3-phosphoshikimate 1-carboxyvinyltransferase	A3M705_ACIBT	0.038	0.2		X				X														X			X				0.45416	0.61955	0.0708	0.13131
Putative monooxygenase flavin-binding family	A3M957_ACIBT	0.26	0.6						X														X			X				0.40195	0.62714	0.21321	0.38274
Putative RND type efflux pump	A3M0R2_ACIBT	0.084	0.5																											1.2589	1.0846	0.74399	0.4245
Putative polyketide biosynthetic dithiol-disulfide isomerase	A3M1H2_ACIBT	0.24	2.1						X														X			X				2.4624	5.1527	9.9658	5.8454
RNA polymerase sigma-54 factor	A3M2I2_ACIBT	0.41	1.4	X	X				X													X	X			X				0.84222	0.52553	0.74068	1.2317
Putative acyl-CoA carboxylase alpha chain protein	A3M4F7_ACIBT	0.85	0.9						X													X	X			X				0.3596	0.23705	0.24435	0.3206
Putative phage integrase	A3M6C3_ACIBT	0.69	2.1		X				X													X				X				0.08031	0	0	0.16492
Ferredoxin--NADP+ reductase	A3M722_ACIBT	0.038	0.2						X														X			X				4.03	3.0557	0.67677	1.0933
Putative protease	A3M725_ACIBT	0.43	0.9						X														X			X				1.6215	1.3154	1.4	1.1557
Putative trypsin-like serine protease	A3M7K2_ACIBT	0.0068	0.1						X														X			X				1.2397	1.426	0.1816	0.12135
Putative lytic murein transglycosylase soluble	A3M930_ACIBT	0.57	0.9						X														X			X				0.36398	0.30753	0.33797	0.27675
4-hydroxy-3-methylbut-2-enyl diphosphate reductase	ISPH_ACIBT	0.52	1.3		X				X													X	X			X				2.1789	1.9412	3.7035	1.8475
Isoleucine--tRNA ligase	A3M0S3_ACIBT	0.082	0.2	X	X				X					X								X	X			X				0.37762	0.24835	0	0.09757
Aspartokinase	A3M3S8_ACIBT	0.017	0.04		X				X													X	X			X				2.6252	3.3786	0.12562	0.11321
Uncharacterized protein	A3M3B3_ACIBT	0.091	0.5																											1.6936	1.9996	0.75842	1.2204
Aspartate aminotransferase A	A3M7N2_ACIBT	0.44	1.3						X													X	X			X				1.5216	1.8098	1.665	2.7275
Phosphate starvation-inducible protein (PhoH-like)	A3M933_ACIBT	0.072	0.7																			X				X				1.6987	2.0284	1.3016	1.238
Putative acetyl-CoA hydrolase/transferase	A3M9M7_ACIBT	0.017	0.2		X				X														X			X				1.4245	1.3179	0.21732	0.46346
Putative VGR-related protein	A3MA09_ACIBT	0.24	1.8																											0.20113	0.16443	0.42809	0.2472
30S ribosomal protein S5	RS5_ACIBT	0.4	2		X				X					X		X		X		X		X				X		X		13.57	11.332	37.798	13.247
Uncharacterized protein	A3M2E7_ACIBT	0.69	1.2																											2.0831	1.1725	2.29	1.5122
Aldehyde dehydrogenase	A3M3N1_ACIBT	0.46	1.5		X				X														X			X				0.14852	0.33081	0.46624	0.26124
Putative oxidoreductase protein	A3M4F3_ACIBT	0.33	1.3						X														X			X				1.8996	1.1415	2.1507	1.9058
Glutamate/aspartate transport protein	A3M4S4_ACIBT	0.049	1.4																											2.5277	2.6299	3.7066	3.2961
Phosphotransferase system fructose-specific EI/HPr/EIIA components	A3M628_ACIBT	0.25	0.6		X		X	X	X														X			X			X	0.54662	0.68879	0.49643	0.18471
Uncharacterized protein	A3M6X0_ACIBT	0.093	4.1						X																					0.21655	0.14548	0.5647	0.93126
Glutamyl-tRNA(Gln) amidotransferase subunit A	GATA_ACIBT	0.019	0.2		X				X													X	X			X				1.1682	1.3776	0.34871	0.151
Ketol-acid reductoisomerase	ILVC_ACIBT	0.66	1.2		X				X													X	X			X				1.2322	2.0833	1.4977	2.4792
Thiol:disulfide interchange protein	A3M0T8_ACIBT	0.8	1.1						X														X			X				5.6317	4.867	8.2581	3.5759
Uncharacterized protein	A3M0W7_ACIBT	0.011	0.5						X													X	X			X				1.0776	0.97036	0.50296	0.50643
Polyphosphate-AMP phosphotransferase	A3M1L8_ACIBT	0.64	1.1						X														X			X				0.49327	0.51321	0.4508	0.68356
Dihydrolipoamide S-acetyltransferase E2 component of the pyruvate dehydrogenase complex	A3M9X2_ACIBT	0.22	0.5						X														X			X				0.9295	1.589	0.6894	0.65833
2-isopropylmalate synthase	A3M1Y3_ACIBT	0.048	0.2		X				X														X			X				0.60879	0.44705	0.09064	0.17135
Sulfate adenylyltransferase subunit 2	A3M3D8_ACIBT	0.12	0.4		X				X													X	X			X				2.5256	1.6469	1.0269	0.44469
Fructose-16-bisphosphate aldolase, class II	A3M4X7_ACIBT	0.00087	0.2		X				X													X	X			X				4.8423	5.038	0.94801	1.0727
Uncharacterized protein	A3M766_ACIBT	0.69	1.3		X				X					X								X	X			X				0.25674	0.32788	0.55158	0.20011
Uncharacterized protein	A7FAV2_ACIBT	0.08	4.3																											0.41196	0.26978	1.7916	1.1312
tRNA-specific 2-thiouridylase MnmA	MNMA_ACIBT	0.055	1.7		X				X					X								X	X			X				1.2204	1.0759	2.087	1.742
TPR domain protein	A3M465_ACIBT	0.061	3.6																											0.31366	0.63334	1.4186	1.9578
Protein TolB	A3M7W7_ACIBT	0.51	0.8				X	X																						1.4056	0.93438	1.1047	0.7767

																														Normalized emPAI Values			
Identified Proteins (1398)	Accession Number	T-Test (p-value): (p < 0.05)	Fold Change Untreated vs. Treated	Biological Regulation	Cellular Process	Developmental Process	Establishment of Localization	Localization	Metabolic Process	Multi-organisim Process	Reproduction	Reproductive Process	Response to Stimulus	Cytoplasm	Extracellular Region	Intracellular Organelle	Membrane	Organelle Part	Plasma Membrane	Ribosome	Antioxidant Activity	Binding	Catalytic Activity	Electron Carrier Activity	Enzyme Regulator Activity	Molecular Function	Molecular Transducer Activity	Structural Molecule Activity	Transporter Activity	Untreated 1	Untreated 2	Treated 1	Treated 2
Putative pyridine nucleotide-disulfide oxidoreductase class I	A3M8W3_ACIBT	0.94	1	X	X				X				X									X	X			X				1.1962	0.94304	0.95884	1.2133
Phage tail tape meausure protein lambda family	A3M530_ACIBT-DECOY	1	INF																											0	0	0	0
Putative Zn-dependent protease with chaperone function	A3M3W6_ACIBT	0.22	0.9						X								X						X			X				3.2843	2.8723	2.7795	2.4635
Uncharacterized protein	A3M4X4_ACIBT	0.59	1.1																											0.81245	1.0104	0.97681	0.97346
AdeI	A3M8A4_ACIBT	0.59	1.2				X	X									X													2.008	1.6071	1.6885	2.5112
Acyl-CoA dehydrogenase	A3M8P8_ACIBT	0.35	0.8						X													X	X			X				0.87383	0.90915	0.85217	0.48948
Ribosomal protein S12 methylthiotransferase RimO	RIMO_ACIBT	0.48	1.2		X				X					X								X	X			X				1.1074	0.99631	1.4042	1.033
Putative ferric siderophore receptor protein	A3M1Y5_ACIBT	0.13	0.3				X	X									X					X				X	X			0.52761	0.34073	0.0713	0.20795
Peptide chain release factor 3	A3M263_ACIBT	0.03	0.2	X	X				X					X								X	X			X				1.103	0.89513	0.09506	0.28818
Putative fatty acid desaturase	A3M7I2_ACIBT	0.047	2.2						X																					1.0231	0.72734	2.063	1.7182
Dihydrolipoyl dehydrogenase	A3M886_ACIBT	0.12	0.9	X	X				X				X									X	X			X				1.6435	1.5142	1.4168	1.3099
Uncharacterized protein	A3M9U8_ACIBT	0.1	1.6																											3.4993	3.097	5.997	4.6497
DNA ligase	DNLJ_ACIBT	0.062	0.5		X				X				X									X	X			X				0.64685	0.75973	0.41781	0.26631
ATP-dependent dsDNA exonuclease	A3M337_ACIBT	0.11	0		X				X				X										X			X				0.05676	0.12129	0	0
Putative outer membrane protein	A3M0V2_ACIBT	0.11	1.4				X	X									X									X			X	1.975	1.5465	2.5221	2.3165
Uncharacterized protein	A3M1J2_ACIBT	0.85	1.1																											0.45301	0.84642	0.51178	0.90915
Histidine kinase	A3M2P5_ACIBT	0.14	1.3	X	X				X				X				X					X	X			X	X			0.45385	0.47219	0.6655	0.5463
Ribosome-binding ATPase YchF	A3M4R5_ACIBT	0.12	0.4						X													X	X			X				3.4583	2.8179	1.9568	0.71558
DNA polymerase III tau and gamma subunits (DNA elongation factor III)	A3M6D9_ACIBT	0.15	0.6		X				X													X	X			X				0.8605	0.89528	0.686	0.38998
Putative short-chain dehydrogenase	A3M6E4_ACIBT	0.003	0.4						X														X			X				2.3942	2.2348	0.8651	0.84255
Amidophosphoribosyltransferase	A3M6Y0_ACIBT	0.2	0.5		X				X													X	X			X				0.39115	0.60926	0.32224	0.19223
3-phosphoshikimate 1-carboxyvinyltransferase	A3M705_ACIBT-DECOY	1	INF																											0	0	0	0
Soluble pyridine nucleotide transhydrogenase	A3M756_ACIBT	0.012	0.3	X	X				X				X									X	X			X				1.0403	0.93775	0.22753	0.3412
Sensory box protein	A3M765_ACIBT	0.11	1.7	X	X				X				X										X			X	X			0.03508	0.03649	0.05143	0.06906
Dihydrolipoyllysine-residue succinyltransferase component of 2-oxoglutarate dehydrogenase complex	A3M885_ACIBT	0.87	1		X				X					X									X			X				1.8389	1.4471	1.2398	1.9025
Glutamate synthase small chain	A3M9I5_ACIBT	0.0097	9.1		X				X													X	X			X				0.07508	0.07812	0.63467	0.7573
Uncharacterized protein	A7FAR4_ACIBT	0.024	2.2																											0.52338	0.68234	1.4004	1.2397
Uncharacterized protein	A7FAX3_ACIBT	0.29	0.7																											0.30544	0.31779	0.16562	0.28643
ATP-dependent Clp protease ATP-binding subunit ClpX	CLPX_ACIBT	0.0032	0.5		X																	X				X				1.3402	1.3944	0.69862	0.64032
Putative RND family drug transporter	A3M2S0_ACIBT	0.016	0.2		X		X	X									X													1.2774	1.5527	0.14255	0.28319
DNA helicase	A3M1V3_ACIBT	0.029	6.8		X				X													X	X			X				0.10636	0.054	0.61818	0.46628
IcmO protein	A3M2F2_ACIBT	0.22	3																											0.06619	0.14205	0.2002	0.4201
Cyclic AMP receptor protein	A3M3W8_ACIBT	0.018	0.3	X	X				X													X				X				9.1727	9.5434	3.2692	1.4156
Uncharacterized protein	A3M634_ACIBT	0.1	0.5						X													X	X			X				1.1648	1.5464	0.62235	0.83824
RND family drug transporter	A3M8A5_ACIBT	0.2	0.4				X	X									X									X			X	0.14217	0.22919	0.04967	0.11468
Uncharacterized protein	A3MAB8_ACIBT	0.27	1.9						X														X			X				0.82048	1.0208	1.2031	2.2716
Probable malate:quinone oxidoreductase	MQO_ACIBT	0.14	0.7		X				X														X			X				0.54536	0.47117	0.30245	0.40861
Phosphoribosylformylglycinamidine cyclo-ligase	PUR5_ACIBT	0.035	4.4		X				X					X								X	X			X				0.80185	0.2276	2.4224	2.0836
Histidine kinase	A3M6M1_ACIBT	0.42	INF	X	X				X				X				X					X	X			X	X			0	0	0	0.07741
Lipoyl synthase	A3M757_ACIBT	0.0041	4.9		X				X					X								X	X			X				0.21671	0.35503	1.428	1.3855
Tn7-like transposition protein B	A3M7S4_ACIBT	0.026	2.2		X				X													X				X				0.1518	0.21548	0.38852	0.42682
50S ribosomal protein L22	A3M979_ACIBT	0.25	1.8		X				X					X		X		X		X		X				X		X		24.492	18.796	48.506	28.289
50S ribosomal protein L9	RL9_ACIBT	0.61	0.7		X				X					X		X				X		X				X		X		10.791	18.034	15.823	5.367

																														Normalized emPAI Values			
Identified Proteins (1398)	Accession Number	T-Test (p-value): (p < 0.05)	Fold Change Untreated vs. Treated	Biological Regulation	Cellular Process	Developmental Process	Establishment of Localization	Localization	Metabolic Process	Multi-organisim Process	Reproduction	Reproductive Process	Response to Stimulus	Cytoplasm	Extracellular Region	Intracellular Organelle	Membrane	Organelle Part	Plasma Membrane	Ribosome	Antioxidant Activity	Binding	Catalytic Activity	Electron Carrier Activity	Enzyme Regulator Activity	Molecular Function	Molecular Transducer Activity	Structural Molecule Activity	Transporter Activity	Untreated 1	Untreated 2	Treated 1	Treated 2
Putative transport protein	A3M0R0_ACIBT	0.13	0.5						X													X	X			X				0.44134	0.53889	0.16244	0.32888
Outer membrane protein assembly factor BamB	A3M214_ACIBT	0.17	2		X		X	X									X													1.4628	1.5219	3.6817	2.2695
Putative long chain fatty-acid CoA ligase	A3M4G2_ACIBT	0.61	0.8						X														X			X				0.51452	0.63067	0.6275	0.3267
Uncharacterized protein	A3M8M8_ACIBT	0.82	1.1																											1.1632	1.4349	2.0223	0.87569
Inner membrane protein (IMP) integration factor	A3M8Z0_ACIBT	0.094	0.6		X		X	X									X													1.7272	2.0638	1.3203	0.94762
Putative outer membrane protein	A3M9U2_ACIBT	0.36	3.2																											2.1388	1.8768	2.6452	10.016
Uncharacterized protein	A3MAB3_ACIBT	0.5	0.7																											1.3401	0.69745	0.78383	0.73117
Phosphoribosylamine--glycine ligase	A3M6S2_ACIBT	0.036	0.05		X				X													X	X			X				2.1095	3.0687	0.12625	0.1138
Iron-regulated protein	A3M2N8_ACIBT	0.019	8.7							X					X							X				X				0.03683	0	0.16774	0.15315
Uncharacterized protein	A3M7Y9_ACIBT	0.0016	1.9																											1.5463	1.6088	3.1146	3.0147
Esterase	A3M8W5_ACIBT	0.54	1.3						X														X			X				0.91022	0.94701	1.6026	0.8207
Catabolite repression control protein	A3M9Y6_ACIBT	0.45	0.8		X				X				X										X			X				0.7945	1.0553	0.87698	0.64404
Uncharacterized protein	A3M7L9_ACIBT	0.99	1																											0.08567	0.08913	0	0.17681
Uncharacterized protein	A3M0W1_ACIBT	0.091	11																											0.41746	0.27319	2.6594	4.866
Amidohydrolase	A3M2M7_ACIBT	0.16	0.3						X														X			X				0.25909	0.26956	0	0.16451
Tyrosine--tRNA ligase	A3M0R7_ACIBT	0.028	0.2		X				X					X								X	X			X				2.1869	2.8621	0.58493	0.41387
ABC transporter-like protein	A3M5I2_ACIBT	0.3	7.4						X													X	X			X				0.04591	0	0.06733	0.27204
D-amino acid dehydrogenase small subunit	A3M667_ACIBT	0.056	4.8						X														X			X				0.27407	0.18269	1.3169	0.89248
Penicillin-binding protein 1B	A3M713_ACIBT	0.37	0.6		X				X				X									X	X			X				0.409	0.19537	0.20223	0.15222
Putative hydrolase	A3M834_ACIBT	0.2	0.6						X														X			X				2.8457	1.9035	1.5809	1.3555
Shikimate kinase	A3M9I9_ACIBT	0.058	1.5		X				X					X								X	X			X				3.5897	4.6	6.4832	6.0503
Putative ATPase	A3MA72_ACIBT	0.47	0.9		X				X													X				X				0.61219	0.77441	0.55182	0.66091
DNA-directed RNA polymerase subunit alpha	RPOA_ACIBT	0.05	0.4		X				X													X	X			X				1.974	2.0538	0.51552	1.0776
D-lactate dehydrogenase	A3M0X1_ACIBT	0.27	0.5		X		X	X	X								X		X			X	X			X				0.42275	0.35587	0.08937	0.32265
Aconitate hydratase 1	A3M0X5_ACIBT-DECOY	1	INF																											0	0	0	0
Glutathione peroxidase	A3M148_ACIBT	0.31	0.6						X				X								X		X			X				13.361	11.502	10.983	3.8878
Uncharacterized protein	A3M2W2_ACIBT	0.00059	0.1		X				X													X	X			X				11.03	11.476	1.1124	1.3099
Putative universal stress protein	A3M430_ACIBT	0.1	0.5										X																	2.604	3.6753	1.5014	1.6534
Cytochrome o ubiquinol oxidase subunit II	A3M6P9_ACIBT	0.049	0.5		X		X	X	X								X					X	X	X		X			X	1.8428	2.2074	0.90171	1.1701
Aminopeptidase N	A3M781_ACIBT	0.35	0.7						X													X	X			X				0.12035	0.12522	0.11552	0.05002
Isocitrate dehydrogenase [NADP]	A3M7J9_ACIBT	0.66	0.8		X				X													X	X			X				0.7721	0.40462	0.57027	0.40271
Putative tRNA-i(6)A37 modification enzyme	A3M932_ACIBT	0.036	2.7						X													X	X			X				1.872	1.2619	4.7464	3.8698
Uncharacterized protein	A3MA32_ACIBT	0.24	0.6																											1.018	1.6038	0.87984	0.74896
Uncharacterized protein	A7FAS8_ACIBT-DECOY	1	INF																											0	0	0	0
Acetyl-coenzyme A carboxylase carboxyl transferase subunit beta	ACCD_ACIBT	0.03	0.3		X				X					X								X	X			X				3.2325	2.4975	0.83443	0.75531
Chaperone protein HscA homolog	HSCA_ACIBT	0.039	0.3		X				X													X	X			X				0.64734	0.49484	0.17354	0.20397
Putative lipoprotein	A3M376_ACIBT	0.00039	0						X														X			X				0.10539	0.10965	0	0
D-ala-D-ala-carboxypeptidase penicillin-binding protein 5 (Precursor)	A3M7F9_ACIBT	0.0059	0.05						X														X			X				1.4288	1.2744	0	0.12511
Uncharacterized protein	A3M4P5_ACIBT	0.065	INF		X		X	X	X														X			X				0	0	0.17034	0.29569
Serine hydroxymethyltransferase	GLYA_ACIBT	0.038	0.1		X				X					X								X	X			X				1.2836	1.5371	0	0.41008
5-methyltetrahydropteroyltriglutamate-homocysteine methyltransferase	A3M2N3_ACIBT	0.13	0.3		X				X													X	X			X				0.76998	1.3997	0.31004	0.29542
Putative FMN oxidoreductase	A3M3K0_ACIBT	0.051	0.3						X													X	X			X				1.1009	1.5315	0.41609	0.25043
Mismatch repair protein	A3M435_ACIBT	0.0007	0.2		X				X				X									X				X				0.90817	0.94488	0.1849	0.16971

Normalized emPAI Values

Identified Proteins (1398)	Accession Number	T-Test (p-value): (p < 0.05)	Fold Change Untreated vs. Treated	Biological Regulation	Cellular Process	Developmental Process	Establishment of Localization	Localization	Metabolic Process	Multi-organisim Process	Reproduction	Reproductive Process	Response to Stimulus	Cytoplasm	Extracellular Region	Intracellular Organelle	Membrane	Organelle Part	Plasma Membrane	Ribosome	Antioxidant Activity	Binding	Catalytic Activity	Electron Carrier Activity	Enzyme Regulator Activity	Molecular Function	Molecular Transducer Activity	Structural Molecule Activity	Transporter Activity	Untreated 1	Untreated 2	Treated 1	Treated 2
Glutathione peroxidase	A3M4Q4_ACIBT	0.12	0.5						X				X								X		X			X				7.96	10.302	5.8163	3.2053
Putative kinase	A3M6M8_ACIBT	0.33	1.3		X				X													X	X			X				0.57236	0.72234	1.0181	0.70966
Putative AAA ATPase superfamily	A3M6X1_ACIBT	0.12	2.5																											0.55337	0.57573	1.0737	1.704
Biotin synthase	BIOB_ACIBT	0.044	1.8		X				X													X	X			X				0.66103	0.52221	0.96931	1.1026
3-isopropylmalate dehydratase large subunit	LEUC_ACIBT	0.022	0.4		X				X													X	X			X				0.44494	0.46292	0.23381	0.15747
UDP-N-acetylmuramate--L-alanine ligase	MURC_ACIBT	0.82	1.1	X	X				X					X								X	X			X				0.32923	0.44372	0.48276	0.3369
Succinyl-CoA ligase [ADP-forming] subunit beta	SUCC_ACIBT	0.12	0.4		X				X													X	X			X				3.127	2.263	1.5329	0.78365
Heme-binding protein A	A3M541_ACIBT	0.56	1.4																											0.11855	0.06003	0.0846	0.15901
UDP-N-acetylmuramoyl-tripeptide--D-alanyl-D-alanine ligase	A3M9K1_ACIBT	0.024	0.1	X	X				X					X								X	X			X				0.94084	1.1313	0	0.21941
GacS-like sensor kinase protein	A3M283_ACIBT	0.18	1.1	X	X				X				X				X						X			X	X			0.19415	0.202	0.22385	0.20789
OmpA/MotB	A3M3X9_ACIBT	0.0071	0.1														X		X											2.3489	2.7129	0.37992	0.36944
Uncharacterized protein	A3M5Z5_ACIBT	0.1	0.4																											3.2369	3.9753	0.72112	2.0553
ATP-dependent RNA helicase RhlB	A3M6Y9_ACIBT	0.059	0.4		X				X					X								X	X			X				1.1556	0.85364	0.43255	0.34045
Putative integrase	A3M8S0_ACIBT	0.0029	INF		X				X													X				X				0	0	0.11431	0.10266
Septum formation penicillin binding protein 3, peptidoglycan synthetase	A3M9K3_ACIBT	0.71	0.7																			X				X				0.39712	0.19011	0.0846	0.35578
Putative two-component sensor	A3M9U7_ACIBT	0.069	2.2	X	X		X	X	X				X				X						X			X	X		X	0.09185	0.12923	0.2811	0.21493
Glucose-6-phosphate isomerase	G6PI_ACIBT	0.11	0.4		X				X					X									X			X				0.43323	0.54213	0.29037	0.12574
Phosphoribosylaminoimidazole-succinocarboxamide synthase	PUR7_ACIBT	0.078	0.1		X				X													X	X			X				4.0342	6.7499	0.4638	1.0075
Peptide chain release factor 1	RF1_ACIBT	0.0051	0.2		X				X					X								X				X				1.7272	1.5527	0.29823	0.20277
50S ribosomal protein L15	RL15_ACIBT	0.36	0.8		X				X					X		X		X		X		X				X		X		5.2946	4.1807	3.2024	4.3761
Nucleoside diphosphate kinase	NDK_ACIBT	0.16	0.07		X				X					X								X	X			X				29.489	11.853	0.87588	1.904
Ribonuclease D	A3M5B9_ACIBT	0.19	2		X				X													X	X			X				0.41184	0.55956	1.2071	0.73637
Replicative DNA helicasechromosome replication chain elongation	A3M6Q8_ACIBT	0.31	1.4		X				X													X	X			X				0.5236	0.78091	0.76778	1.0457
Putative phosphate transporter	A3M7H2_ACIBT	0.48	0.8																											0.49659	0.86011	0.51901	0.52492
Peptidoglycan-associated lipoprotein	A3M7W8_ACIBT	0.084	0.7														X		X											4.9744	5.1755	2.8553	3.8878
Putative long-chain fatty acid transport protein	A3M8E1_ACIBT	0.84	1.1																											1.1614	0.4405	0.93411	0.83573
Uncharacterized protein	A3M8S8_ACIBT	0.24	0.4		X				X													X	X			X				1.7	0.82377	0.58326	0.50274
Putative acyl-CoA dehydrogenase	A3M9B4_ACIBT	0.17	1.8						X													X	X			X				0.25362	0.12483	0.3719	0.30676
Putative RND family drug transporter	A3M1D6_ACIBT	0.77	1.1				X	X									X									X			X	0.16205	0.08129	0.11457	0.16013
Putative transport protein	A3M424_ACIBT	0.18	2.7						X													X	X			X				0.70221	1.583	2.231	3.8698
Adenylate kinase	KAD_ACIBT	0.045	0.01		X				X					X								X	X			X				16.173	10.434	0.2449	0.10605
Phosphate acetyltransferase	A3M1Z2_ACIBT	0.089	0.1		X				X					X									X			X				0.47902	0.82694	0	0.13788
Poly(A) polymerase I	A3M290_ACIBT	0.16	0.6		X				X													X	X			X				0.60131	0.41724	0.32989	0.25464
Putative ClpA/B-type chaperone	A3M491_ACIBT	0.13	2.1																			X				X				0.0795	0.12639	0.17813	0.25908
Glutamate dehydrogenase	A3M9D7_ACIBT	0.033	3.5		X				X														X			X				0.38356	0.28741	1.3294	1.0286
Putative RND family drug transporter	A3M9L6_ACIBT	0.12	3.6				X	X																		X			X	0.25909	0.3734	1.4427	0.82888
50S ribosomal protein L10	RL10_ACIBT	0.17	1.4		X				X					X		X				X		X				X		X		7.7357	5.1224	9.0833	9.2826
Paraquat-inducible protein	A3M6T7_ACIBT	0.063	1.9																											0.20384	0.13718	0.2989	0.34173
ATP-dependent Clp protease proteolytic subunit	A3M1Y7_ACIBT	0.19	0.4						X					X									X			X				3.2431	2.1751	0.26125	1.6649
Uncharacterized protein	A3M454_ACIBT	0.039	0						X													X	X			X				0.82836	0.54912	0	0
Exopolyphosphatase	A3M2B2_ACIBT	0.74	1.1		X				X													X	X			X				0.30473	0.31705	0.44685	0.25004
Adenylosuccinate synthetase	A3M3W5_ACIBT	0.0048	0.6		X				X					X								X	X			X				1.203	1.2516	0.71616	0.65879
Putative diguanylate cyclase/phosphodiesterase	A3M632_ACIBT	0.054	3.2	X	X								X													X				0.1536	0.05087	0.30737	0.33727

Identified Proteins (1398)	Accession Number	T-Test (p-value): (p < 0.05)	Fold Change Untreated vs. Treated	Biological Regulation	Cellular Process	Developmental Process	Establishment of Localization	Localization	Metabolic Process	Multi-organisim Process	Reproduction	Reproductive Process	Response to Stimulus	Cytoplasm	Extracellular Region	Intracellular Organelle	Membrane	Organelle Part	Plasma Membrane	Ribosome	Antioxidant Activity	Binding	Catalytic Activity	Electron Carrier Activity	Enzyme Regulator Activity	Molecular Function	Molecular Transducer Activity	Structural Molecule Activity	Transporter Activity	Normalized emPAI Values			
																														Untreated 1	Untreated 2	Treated 1	Treated 2
NADH-quinone oxidoreductase subunit I	NUOI_ACIBT	0.036	1.7						X								X		X			X	X			X				1.8983	1.9751	3.5943	3.0553
Putative penicillin binding protein (PonA)	A3M9J5_ACIBT	0.32	0.6														X					X				X				0.43175	0.34663	0.10986	0.3412
Putative membrane-bound lytic murein transglycosylase	A3M8X1_ACIBT	0.011	0.1																											1.3863	1.6436	0.12259	0.23895
Putative UDP-galactose phosphate transferase	A3M0W2_ACIBT	0.015	2.7						X														X			X				1.1449	1.5536	3.4667	3.8579
Dehydrogenase/reductase	A3M278_ACIBT	< 0.00010	3.7						X														X			X				0.63584	0.66154	2.427	2.4337
Dihydropteroate synthase	A3M2H9_ACIBT	0.68	0.9		X				X													X	X			X				2.6146	1.8777	1.721	2.32
Putative signal peptide	A3M3D7_ACIBT	0.78	0.9																											0.76124	1.219	1.1163	0.65113
Oxidoreductase	A3M4F4_ACIBT	0.41	0.8						X														X			X				0.54536	0.89298	0.53628	0.54498
Acyl-CoA dehydrogenase	A3M4G0_ACIBT	0.013	0.3						X													X	X			X				1.0194	0.8857	0.28488	0.19331
Putative outer membrane protein (OmpH)	A3M651_ACIBT	0.023	0.3																			X				X				5.8066	4.8221	1.1657	1.8144
Adenosylhomocysteinase	A3M762_ACIBT	0.0043	0.4		X				X					X									X			X				0.68091	0.70843	0.23652	0.28616
Putative protease (SohB)	A3M7G8_ACIBT	0.14	1.6						X								X		X				X			X				1.2923	0.90196	1.895	1.5535
DNA replication protein	A3MAB7_ACIBT	0.059	2.7		X				X														X			X				0.22736	0.23654	0.73865	0.53317
Fructose-1,6-bisphosphatase class 1	F16PA_ACIBT	0.12	0.4		X				X					X								X	X			X				2.1348	1.3566	0.76616	0.68457
50S ribosomal protein L11	RL11_ACIBT	0.0041	1.9		X				X					X		X				X		X				X		X		4.2474	4.419	8.2456	7.8021
UPF0246 protein A1S_2267	Y2267_ACIBT	0.17	0.5																											2.4356	2.1244	1.6245	0.54855
Phenylalanine--tRNA ligase alpha subunit	A3M2A8_ACIBT	0.00047	0.07		X				X					X								X	X			X				2.0218	2.1035	0.15807	0.14389
Two-component response regulator	A3M9M5_ACIBT	0.099	0.2	X	X				X				X									X				X				2.5634	4.3515	0.69198	0.91744
Amino acid adenylation	A3M108_ACIBT-DECOY	1	INF																											0	0	0	0
Uncharacterized protein	A3M9B9_ACIBT	0.16	3.5		X				X				X									X	X			X				0.08144	0	0.11942	0.16742
Arabinose 5-phosphate isomerase	A3M420_ACIBT	0.00078	0.02						X													X	X			X				1.8658	1.9412	0	0.0718
Beta-ketoacyl-ACP synthase I	A3M301_ACIBT	0.032	0.8						X														X			X				2.5634	2.667	2.1187	1.9291
Putative hydrolase haloacid dehalogenase-like family	A3M591_ACIBT	0.84	0.8						X													X	X			X				0.09698	0.26991	0.06954	0.24145
Phosphate import ATP-binding protein PstB	A3M7G9_ACIBT	0.042	2.4				X	X	X								X		X			X	X			X			X	0.94318	0.58262	1.7122	2.0035
30S ribosomal protein S9	A3M908_ACIBT	0.69	1.1		X				X					X		X				X						X		X		6.2923	4.8997	6.9057	5.2765
Uncharacterized protein	A3M9S1_ACIBT	0.18	1.6																											0.25159	0.16821	0.36891	0.2869
Uncharacterized protein	A3MA33_ACIBT	0.015	0.2		X				X													X	X			X				1.958	2.0372	0.64779	0.28051
tRNA dimethylallyltransferase	MIAA_ACIBT	0.058	1.3		X				X													X	X			X				1.281	1.1029	1.5544	1.5374
Glutamate 5-kinase	PROB_ACIBT	0.00039	0		X				X					X								X	X			X				1.2627	1.3137	0	0
Magnesium and cobalt efflux protein	A3M1P1_ACIBT	0.072	2.7						X													X	X			X				0.6084	0.44678	1.1862	1.6736
Putative DNA modification methylase	A3M1A3_ACIBT	0.54	1.3		X				X													X	X			X				0.25973	0.37436	0.52763	0.29686
Putative signal peptide	A3M3G5_ACIBT	0.33	0.6																											1.2483	2.0112	1.2924	0.7926
Uncharacterized protein	A3M4A3_ACIBT	0.058	1.6																											2.0906	2.1751	3.0656	3.6893
Putative outer membrane protein	A3M9W2_ACIBT	0.75	1.1																											2.2853	1.5923	1.7885	2.4447
Glucosamine--fructose-6-phosphate aminotransferase	A3MA40_ACIBT	0.023	0.4						X													X	X			X				3.6848	3.8338	1.9636	1.3278
50S ribosomal protein L7/L12	RL7_ACIBT	1	1		X				X					X		X				X						X		X		10.757	26.398	21.106	16.111
30S ribosomal protein S10	RS10_ACIBT	0.48	1.8		X				X					X		X				X		X				X		X		7.1076	7.3949	19.931	6.273
30S ribosomal protein S11	RS11_ACIBT	0.24	3.1		X				X					X		X				X		X				X		X		3.7836	7.2671	23.705	10.265
Uncharacterized protein	A3M4N5_ACIBT	0.42	INF						X														X			X				0	0	0	0.07376
Uncharacterized protein	A3M2D8_ACIBT	0.0079	INF																											0	0	0.32423	0.38776
MviM protein	A3M0V4_ACIBT	0.26	1.6						X														X			X				1.0645	1.1075	2.2466	1.3352
Putative chromatin partitioning ATPase (ParA family ATPase)	A3M115_ACIBT	0.067	4.3																											0.27076	0.84551	2.7782	1.9687
DNA primase	A3M2X8_ACIBT	0.33	0.6						X														X			X				0.33719	0.45482	0.11106	0.34561

Identified Proteins (1398)	Accession Number	T-Test (p-value): (p < 0.05)	Fold Change Untreated vs. Treated	Biological Regulation	Cellular Process	Developmental Process	Establishment of Localization	Localization	Metabolic Process	Multi-organisim Process	Reproduction	Reproductive Process	Response to Stimulus	Cytoplasm	Extracellular Region	Intracellular Organelle	Membrane	Organelle Part	Plasma Membrane	Ribosome	Antioxidant Activity	Binding	Catalytic Activity	Electron Carrier Activity	Enzyme Regulator Activity	Molecular Function	Molecular Transducer Activity	Structural Molecule Activity	Transporter Activity	Normalized emPAI Values			
																														Untreated 1	Untreated 2	Treated 1	Treated 2
Putative N-6 Adenine-specific DNA methylase	A3M3X5_ACIBT	0.23	0.6		X				X					X								X	X			X				0.38782	0.29048	0.26212	0.11351
Putative transcriptional regulator	A3M622_ACIBT	0.18	2.1	X	X				X													X				X				0.93092	0.96855	2.4485	1.4665
Uncharacterized protein	A3M6V3_ACIBT	0.00081	9.9																											0.24499	0.2549	2.4125	2.5386
Methylmalonate-semialdehyde dehydrogenase	A3M6W5_ACIBT	0.31	1.9						X														X			X				0.14154	0.3143	0.57228	0.30744
Uncharacterized protein	A3M8E4_ACIBT	0.073	0.5		X				X														X			X				0.90133	0.67674	0.35355	0.41301
Uncharacterized protein	A3M8G8_ACIBT	0.62	1.4						X														X			X				0.77759	1.6577	2.3363	1.0117
2-nonaprenyl-3-methyl-6-methoxy-1,4-benzoquinol hydroxylase	COQ7_ACIBT	0.17	2.2		X				X								X		X			X	X			X				0.86465	1.222	1.7224	2.8055
L-lactate dehydrogenase	LLDD_ACIBT	0.52	1.4		X				X								X		X			X	X			X				0.56552	0.20558	0.63377	0.45133
Glucose dehydrogenase	A3M8K9_ACIBT	0.0065	0.1						X																					1.5103	1.5714	0.27121	0.05635
Putative enoyl-CoA hydratase/isomerase family protein	A3M100_ACIBT	0.14	1.6						X														X			X				0.96267	0.64655	1.1499	1.3661
Phenylacetic acid degradation protein paaN	A3M4B9_ACIBT	0.47	0.7						X														X			X				0.27944	0.22699	0.07443	0.26094
Organic solvent tolerance protein	A3M4X9_ACIBT	0.88	1		X								X				X													0.32726	0.26484	0.27225	0.30798
Putative ferric siderophore receptor protein	A3M588_ACIBT	0.077	0.1				X	X									X									X	X			0.09307	0.14841	0	0.02892
Electron transfer flavoprotein beta-subunit	A3M801_ACIBT	0.034	0.3																					X		X				3.5145	3.0633	0.47239	1.2754
Putative toluene tolerance protein (Ttg2D)	A3M9A3_ACIBT	0.093	0.5																											4.467	3.771	2.6291	1.464
Cell division protein ftsA	A3M9X7_ACIBT	0.23	1.3	X	X																									0.92421	0.96156	1.3552	1.0515
Uncharacterized protein	A7FBU5_ACIBT	0.014	0.4																											4.5277	4.7107	2.3565	1.7779
50S ribosomal protein L13	RL13_ACIBT	0.95	1		X				X					X		X				X						X		X		8.1801	3.9137	7.2274	5.1941
Uncharacterized protein	A3M2D9_ACIBT	0.46	2.1																											0.05867	0.12548	0.08604	0.30859
Putative fatty acid desaturase	A3M8P3_ACIBT	0.044	6.5						X														X			X				0	0.09048	0.26555	0.32553
ATPase	A3M7S5_ACIBT	0.011	4.1																											0.12915	0.06525	0.40196	0.39336
Putative hemolysin-related protein	A3M3E3_ACIBT	0.0057	0.3						X													X	X			X				0.45655	0.475	0.11535	0.1613
2-octaprenylphenol hydroxylase of ubiquinone biosynthetic pathway	A3M1L4_ACIBT	0.57	0.6						X														X			X				0.27795	0.28919	0	0.33877
Adenylosuccinate lyase	A3M7G5_ACIBT	0.088	0.1		X				X														X			X				0.54854	0.95925	0.1118	0.10032
Uncharacterized protein	A3M1T0_ACIBT	0.87	1																											2.6913	2.8001	2.2776	3.4252
Uncharacterized protein	A3M2D5_ACIBT	0.065	2																											0.44457	0.33123	0.85398	0.68359
Quinolinate synthase A	A3M2L3_ACIBT	0.92	1		X				X					X								X	X			X				0.61925	0.35045	0.31386	0.60892
Aldose 1-epimerase	A3M3A3_ACIBT	0.62	0.9						X													X	X			X				0.56552	0.58838	0.63377	0.35909
Uncharacterized protein	A3M4P6_ACIBT	0.33	0.7																											1.583	1.9412	0.77774	1.6037
Pseudouridine synthase	A3M7K0_ACIBT	0.095	0.7		X				X													X	X			X				1.1022	0.8945	0.66508	0.69985
Uncharacterized protein	A3M8I6_ACIBT	0.18	0.4																											4.8276	2.5091	1.4487	1.2587
Putative antioxidant protein	A3M8M5_ACIBT	0.17	0.2						X												X		X			X				4.2212	8.76	2.1749	0.36914
ATPase E1-E2 type:Copper-translocating P-type ATPase:Heavy metal translocating P-type ATPase	A3M8V0_ACIBT	0.67	0.9				X	X	X								X					X	X			X			X	0.25081	0.26095	0.28781	0.15926
Dihydroxy-acid dehydratase	ILVD_ACIBT	0.12	1.7		X				X													X	X							0.05979	0.0622	0.08767	0.12047
Uncharacterized protein	A7FAX7_ACIBT	0.054	3.3																											0.1352	0.29935	0.8123	0.63041
Uncharacterized protein	A3M5C4_ACIBT	0.99	1																											0.27407	0.08783	0.12379	0.24155
Phenylacetate-CoA oxygenase/reductase PaaK subunit	A3M4C4_ACIBT	0.074	0.2		X				X				X									X	X	X		X				0.45617	0.4746	0	0.2072
Phosphoserine aminotransferase	SERC_ACIBT	0.0013	0.08		X				X					X								X	X			X				2.7084	2.8179	0.14709	0.29347
Putative phage primase	A3M2F4_ACIBT	0.42	INF																											0	0	0	0.10949
Choline dehydrogenase-related flavoprotein	A3M3Q5_ACIBT	0.61	2.8						X													X	X			X				0.06294	0	0	0.17474
33 kDa chaperonin	A3M1N2_ACIBT	0.16	3.5		X									X								X				X				0.40613	0.79121	1.4204	2.7444
Putative RND family drug transporter	A3M244_ACIBT	0.48	1.4				X	X									X					X				X			X	0.55141	0.96478	1.3598	0.7782
Citrate synthase	A3M879_ACIBT	0.15	1.4		X				X					X									X			X				0.48434	0.38735	0.54593	0.65252

Identified Proteins (1398)	Accession Number	T-Test (p-value): (p < 0.05)	Fold Change Untreated vs. Treated	Biological Regulation	Cellular Process	Developmental Process	Establishment of Localization	Localization	Metabolic Process	Multi-organisim Process	Reproduction	Reproductive Process	Response to Stimulus	Cytoplasm	Extracellular Region	Intracellular Organelle	Membrane	Organelle Part	Plasma Membrane	Ribosome	Antioxidant Activity	Binding	Catalytic Activity	Electron Carrier Activity	Enzyme Regulator Activity	Molecular Function	Molecular Transducer Activity	Structural Molecule Activity	Transporter Activity	Untreated 1	Untreated 2	Treated 1	Treated 2
																														Normalized emPAI Values			
Putative membrane protein	A3M8G5_ACIBT	0.47	0.8																											2.1579	2.8217	2.4713	1.3702
Putative esterase	A3M8L8_ACIBT	0.54	1.4						X																					0.77374	1.0263	1.7946	0.77712
30S ribosomal protein S6	RS6_ACIBT	0.091	2		X				X					X		X				X		X				X		X		3.155	2.3802	6.2282	4.6708
Ferric enterobactin receptor	A3M3B8_ACIBT	0.49	1.6				X	X									X									X	X			0.14011	0.07062	0.09953	0.24507
Phage integrase	A3M3W1_ACIBT	0.42	INF		X				X													X				X				0	0	0	0.11121
Putative oxidoreductase (Short chain dehydrogenase)	A3M5V3_ACIBT	0.98	1						X														X			X				0.72747	0.52897	0.4638	0.8013
Peptide chain release factor 1	RF1_ACIBT-DECOY	1	INF																											0	0	0	0
Dual-specificity RNA methyltransferase RlmN	RLMN_ACIBT	0.13	INF		X				X					X								X	X			X				0	0	1.8369	0.79542
Uncharacterized protein	A3M864_ACIBT	0.42	0						X														X			X				0	0.12771	0	0
Regulatory protein LysR:LysR, substrate-binding	A3M6W8_ACIBT	0.25	2.2	X	X				X													X				X				0.11457	0.1192	0.168	0.34194
Enoyl-[acyl-carrier-protein] reductase [NADH]	A3M243_ACIBT	0.12	0.6		X				X														X			X				1.0437	1.3511	0.63489	0.8246
Uncharacterized protein	A3M475_ACIBT	0.91	1																											0.55556	0.40983	0.57762	0.35276
Biotin carboxylase (A subunit of acetyl-CoA carboxylase)	A3M694_ACIBT	0.0012	0.3						X													X	X			X				0.68279	0.71038	0.23708	0.22106
Putative signal peptide	A3M844_ACIBT	0.51	1.3																											2.1839	3.1198	4.3971	2.5515
Coproporphyrinogen oxidase	A3M9B1_ACIBT	0.023	2.7		X				X														X			X				0.50737	0.52788	1.241	1.5061
Uncharacterized protein	A7FBY8_ACIBT	0.39	0.7																											1.9377	1.1019	1.2138	0.83735
Diaminopimelate epimerase	DAPF_ACIBT	0.083	0.6		X				X					X									X			X				1.2921	1.0807	0.63228	0.82043
Putative short-chain dehydrogenase	A3MA17_ACIBT	0.16	2.5						X														X			X				0.8395	0.87343	1.5761	2.7813
Phosphoglucosamine mutase	GLMM_ACIBT	0.3	0.7						X													X	X			X				0.26367	0.38025	0.24806	0.23206
S-adenosylmethionine synthase	METK_ACIBT	0.1	0.3		X				X					X								X	X			X				0.56012	0.89486	0.13753	0.27192
Uncharacterized protein	A3MAA6_ACIBT	0.045	0.08		X				X														X			X				0.35636	0.52035	0	0.06824
UDP-N-acetylmuramoyl-L-alanyl-D-glutamate--2,6-diaminopimelate ligase	A3M9K2_ACIBT	0.01	0.09	X	X				X					X								X	X			X				0.50633	0.5268	0	0.0935
RtcR	A3M6Z8_ACIBT	0.42	INF	X																		X				X				0	0	0	0.08388
N-succinylglutamate 5-semialdehyde dehydrogenase	A3M9D3_ACIBT	0.087	0.06		X				X														X			X				1.1856	2.1854	0.10962	0.0983
Putative FMN oxidoreductase	A3M8J0_ACIBT	0.0022	0.2						X													X	X			X				0.90039	0.93678	0.14255	0.20277
Uncharacterized protein	A3M706_ACIBT	0.0091	0.02		X				X					X								X	X			X				1.4513	1.7457	0	0.06054
Peptidylprolyl isomerase	A3M571_ACIBT	0.0017	0.09		X				X														X			X				3.023	3.1452	0.18447	0.38137
Phage integrase	A3M8T8_ACIBT	0.0012	INF		X				X													X				X				0	0	0.24101	0.22499
Putative oxoacyl-(Acyl carrier protein) reductase	A3M0T5_ACIBT	0.0031	0.1						X														X			X				2.86	2.9756	0.4638	0.20084
Ubiquinone biosynthesis O-methyltransferase	A3M0T7_ACIBT	0.00025	2.4		X				X														X			X				1.2913	1.3435	3.19	3.2185
Phosphomannomutase	A3M325_ACIBT	0.28	0.7						X													X	X			X				0.42649	0.34253	0.22501	0.3369
tRNA N6-adenosine threonylcarbamoyltransferase	A3M6X3_ACIBT	0.3	1.9		X				X					X								X	X			X				1.0512	0.70087	2.216	1.1287
Putative signal peptide	A3M7H9_ACIBT	0.12	0.9																											1.6573	1.7243	1.4897	1.5858
Uncharacterized protein	A3M7J1_ACIBT	0.1	4.2																											0.1448	0.15065	0.4538	0.77946
Putative partition-related protein	A3M9E4_ACIBT	0.99	1																											2.1071	3.4043	3.877	1.6788
Peptide chain release factor 2	A3M9W1_ACIBT	0.023	0.6		X				X					X								X				X				1.1261	1.1717	0.56487	0.71506
ATP synthase subunit b	ATPF_ACIBT	0.055	0.4		X		X	X	X								X		X				X			X			X	3.368	4.5499	1.3121	1.6185
Phosphatidylserine decarboxylase proenzyme	PSD_ACIBT	0.011	1.9		X				X														X			X				0.8053	0.83785	1.5086	1.6635
tRNA/tmRNA (uracil-C(5))-methyltransferase	TRMA_ACIBT	0.52	1.4		X				X														X			X				1.0304	0.44555	0.82133	1.1919
IncC protein	A3M2E4_ACIBT	0.036	0.2																											0.77038	1.0216	0.17926	0.26091
Putative transcriptional regulator (TetR family)	A3M7I4_ACIBT	0.89	0.9	X	X				X													X				X				1.0275	0.79351	1.5067	0.09759
ATP synthase gamma chain	ATPG_ACIBT	0.027	1.4		X		X	X	X								X		X			X	X			X			X	0.12356	0.12855	0.18118	0.1661
Chaperone SurA	A3M4X8_ACIBT	0.35	0.6	X	X				X	X												X	X			X				0.25909	0.26956	0.2439	0.05087

																														Normalized emPAI Values			
Identified Proteins (1398)	Accession Number	T-Test (p-value): (p < 0.05)	Fold Change Untreated vs. Treated	Biological Regulation	Cellular Process	Developmental Process	Establishment of Localization	Localization	Metabolic Process	Multi-organisim Process	Reproduction	Reproductive Process	Response to Stimulus	Cytoplasm	Extracellular Region	Intracellular Organelle	Membrane	Organelle Part	Plasma Membrane	Ribosome	Antioxidant Activity	Binding	Catalytic Activity	Electron Carrier Activity	Enzyme Regulator Activity	Molecular Function	Molecular Transducer Activity	Structural Molecule Activity	Transporter Activity	Untreated 1	Untreated 2	Treated 1	Treated 2
Exonuclease V gamma chain	A3M1M0_ACIBT	0.19	0.2		X				X														X			X				0.03664	0.07756	0	0.02326
2-dehydro-3-deoxyphosphooctonate aldolase	KDSA_ACIBT	0.0091	0.09		X				X					X									X			X				1.5433	1.6057	0	0.26936
Uncharacterized protein	A3M297_ACIBT	0.042	0.1																											7.0398	4.8989	1.0275	0.65124
Putative polysaccharide deacetylase	A3M8R3_ACIBT	0.11	0.5						X														X			X				0.29612	0.30808	0.20369	0.0882
Putative signal peptide	A3M9I3_ACIBT	0.5	0.6																											2.2112	6.5209	3.2424	1.7674
UDP-N-acetylglucosamine--N-acetylmuramyl-(pentapeptide) pyrophosphoryl-undecaprenol N-acetylglu	MURG_ACIBT	0.29	1.3	X	X				X								X		X			X	X			X				0.32941	0.47929	0.48303	0.59305
30S ribosomal protein S13	RS13_ACIBT	0.38	0.8		X				X					X		X				X		X				X		X		3.9619	5.6714	4.1258	3.4612
CsuC	A3M6U8_ACIBT	0.00057	0.1		X																									1.3536	1.4083	0.1936	0.17817
Acetyl-coenzyme A synthetase	A3M9V4_ACIBT	0.05	1.4		X				X													X	X			X				0.05401	0.05619	0.07919	0.07034
ATP-dependent DNA helicase	A3M998_ACIBT	0.42	0		X				X				X									X	X			X				0	0.10865	0	0
General secretion pathway protein F	A3M1N5_ACIBT	0.028	INF		X		X	X									X									X			X	0	0	0.1298	0.18316
Uncharacterized protein	A3M7E6_ACIBT	0.61	2.7	X	X								X													X				0.04815	0	0	0.13092
Uncharacterized protein	A3M432_ACIBT	0.68	1.4																											1.3811	0.57027	2.0253	0.67558
Putative propionyl-CoA carboxylase (Beta subunit)	A3M4F9_ACIBT	0.44	1.5						X														X			X				0.2963	0.14461	0.43448	0.24292
Methyl-directed mismatch repair enzyme	A3M6J7_ACIBT	0.26	2		X				X				X									X				X				0.30804	0.7346	1.2656	0.77488
Putative membrane protein	A3M808_ACIBT	0.27	0.6																											2.2484	2.3393	2.0011	0.8665
Uncharacterized protein	A3M897_ACIBT	0.6	0.7																											1.1135	3.0155	1.6328	1.3043
4-hydroxyphenylpyruvate dioxygenase	A3MA63_ACIBT	0.07	0.5		X				X													X	X			X				0.59218	0.47001	0.30177	0.20528
Ribosomal protein L11 methyltransferase	PRMA_ACIBT	0.39	0.8		X				X					X									X			X				1.4105	1.2093	0.81788	1.2658
Aspartate-semialdehyde dehydrogenase	A3M1T8_ACIBT	0.011	0.09		X				X					X								X	X			X				0.31934	0.33225	0	0.06173
Transcriptional regulator protein (OmpR family)	A3M8P5_ACIBT	0.0099	0.09	X	X				X				X									X				X				1.9954	1.6815	0.21952	0.09506
Putative nicotinamide phosphoribosyl transferase	A3M5L3_ACIBT	0.2	0		X				X														X			X				0.07093	0.23662	0	0
Lipoprotein-releasing system ATP-binding protein LolD	A3M7Y4_ACIBT	0.67	2.2				X	X	X								X		X			X	X			X			X	0.15934	0	0	0.35169
Putative sigma(54) modulation protein RpoX	A3M2I3_ACIBT	0.078	2.4						X																					4.2244	4.3952	8.5782	12.16
Methionine--tRNA ligase	A3M2S4_ACIBT	0.2	1.5		X				X					X								X	X			X				0.16022	0.10849	0.23494	0.17801
UDP-N-acetylenolpyruvoylglucosamine reductase	A3M6D3_ACIBT	0.14	0.5	X	X				X					X								X	X			X				1.1585	1.4289	0.91125	0.49791
Toluene tolerance efflux transporter	A3M9A6_ACIBT	0.26	1.9						X													X	X			X				0.63016	0.65563	1.5761	0.85049
Uncharacterized protein	A3M9Z5_ACIBT	0.29	0.8						X																					3.9008	2.8865	2.7919	2.4769
Ribonuclease G endoribonuclease G	A3M8E5_ACIBT	0.77	1.1		X				X													X	X			X				0.15144	0.24463	0.22206	0.20612
Putative PLP-dependent aminotransferase	A3M4Q2_ACIBT	0.14	0.06						X													X	X			X				0.23566	0.54516	0	0.04657
Uncharacterized protein	A3M6D5_ACIBT	0.23	0.5																											0.51268	0.29442	0.12752	0.24972
Ribosomal RNA large subunit methyltransferase E	RLME_ACIBT	0.071	0.2		X				X					X									X			X				1.9104	3.0476	0.24608	0.53921
3-ketoacyl-CoA thiolase	FADA_ACIBT	0.17	0.3		X				X					X									X			X				1.4628	0.7521	0.14057	0.45901
SsDNA exonuclease	A3M9W3_ACIBT	0.39	1.2		X				X				X									X	X			X				0.1984	0.13362	0.18833	0.22287
Putative nitroreductase	A3M8G2_ACIBT	0.0049	0.06						X														X			X				0.74663	0.7768	0	0.09589
Succinylglutamate desuccinylase	ASTE_ACIBT	0.045	0.06		X				X													X	X			X				1.0512	1.5723	0	0.14628
Rubredoxin reductase	A3M3D2_ACIBT	0.021	0.1						X													X	X			X				1.6246	1.2556	0.13679	0.19388
Methylisocitrate lyase	A3M0X4_ACIBT	0.0082	0.2		X				X														X			X				2.163	1.897	0.3865	0.50001
Putative hydrolase haloacid dehalogenase-like family	A3M1S6_ACIBT	0.48	0.5		X				X														X			X				1.4167	1.866	0	1.7325
Iron-sulfur-dependent L-serine dehydratase single chain form	A3M4B8_ACIBT	0.15	INF						X													X	X			X				0	0	0.11509	0.28914
Uncharacterized protein	A3M2F0_ACIBT	0.0037	13																											0.20161	0.10029	1.8516	2.0446
Putative acyl-CoA dehydrogenase	A3M759_ACIBT	0.003	INF		X				X														X			X				0	0	0.10708	0.09595
Formyltetrahydrofolate deformylase	A3M1W2_ACIBT	0.62	1.2		X				X													X	X			X				0.40771	0.79461	0.84468	0.61786

Identified Proteins (1398)	Accession Number	T-Test (p-value): (p < 0.05)	Fold Change Untreated vs. Treated	Biological Regulation	Cellular Process	Developmental Process	Establishment of Localization	Localization	Metabolic Process	Multi-organisim Process	Reproduction	Reproductive Process	Response to Stimulus	Cytoplasm	Extracellular Region	Intracellular Organelle	Membrane	Organelle Part	Plasma Membrane	Ribosome	Antioxidant Activity	Binding	Catalytic Activity	Electron Carrier Activity	Enzyme Regulator Activity	Molecular Function	Molecular Transducer Activity	Structural Molecule Activity	Transporter Activity	Normalized emPAI Values			
																														Untreated 1	Untreated 2	Treated 1	Treated 2
Carbonic anhydrase	A3M3C2_ACIBT	0.2	0.4						X													X	X			X				2.7121	5.2196	1.8802	1.0701
Aminoacyl-histidine dipeptidase	A3M703_ACIBT	0.18	2.4						X														X			X				0.52792	0.44157	1.4952	0.83824
Cell division protein FtsQ	A3M9X8_ACIBT	0.45	0.8		X						X	X					X		X											0.77712	1.031	0.85874	0.62923
Urocanate hydratase	A3MA51_ACIBT	0.78	0.9						X														X			X				6.7679	14.775	9.9241	9.0173
NADH-quinone oxidoreductase subunit B	NUOB_ACIBT	0.33	1.7				X	X	X								X		X			X	X			X				0.77943	1.094	2.0001	1.094
Threonine synthase	A3M1B9_ACIBT	0.00025	2.3		X				X													X	X			X				0.20398	0.21222	0.4697	0.46857
Putative lipopolysaccharide core biosynthesis glycosyl transferase LpsC	A3M8R2_ACIBT	0.51	0.6						X														X			X				0.28755	0.67992	0.19813	0.41496
Multifunctional CCA protein	CCA_ACIBT	0.053	0.3		X				X													X	X			X				0.3743	0.50672	0.122	0.17132
Single-stranded DNA-binding protein	A3M9T2_ACIBT	0.12	0.2		X				X													X				X				1.8219	3.0843	0.28149	0.87556
Elongation factor P--(R)-beta-lysine ligase	A3M809_ACIBT	0.16	0.3		X				X					X								X	X			X				0.36004	0.23732	0.15906	0
Uncharacterized protein	A3M4A1_ACIBT	0.19	4.3																											0.0874	0	0.12816	0.25114
Transcription elongation factor GreA	A3M857_ACIBT	0.21	0.3	X	X				X													X				X				2.3684	4.1758	1.8436	0.32182
AdeB	A3M5I3_ACIBT	0.0041	INF				X	X									X									X			X	0	0	0.10267	0.11675
Polyphosphate kinase	PPK_ACIBT-DECOY	1	INF																											0	0	0	0
Tryptophanyl-tRNA synthetase	A3M889_ACIBT	0.088	0.08		X				X					X								X	X			X				1.1967	0.66532	0	0.13979
Putative transcriptional regulator (LysR family)	A3M5G0_ACIBT	0.014	0.1	X	X				X													X				X				0.7322	0.76179	0	0.15723
DNA-binding ATP-dependent protease La	A3M3G9_ACIBT	0.058	0.6						X													X	X			X				3.1764	2.7338	2.0126	1.6684
Alkyl hydroperoxide reductase C22 subunit	A3M3Z1_ACIBT	0.23	0.4						X				X								X		X			X				3.0202	5.9408	2.0528	1.9177
Putative transcriptional regulator (TetR family)	A3M4X3_ACIBT	0.055	5.3	X	X				X													X				X				0.18286	0.67544	1.8935	2.6439
Uncharacterized protein	A3M503_ACIBT	0.038	4.7																											0.92572	0.96313	5.1811	3.7644
Histidine kinase	A3M8R8_ACIBT	0.21	1.6	X	X				X				X				X					X	X			X	X			0.36837	0.75625	0.87735	0.97538
Putative lipoprotein	A3M8Z6_ACIBT	0.39	0.8														X		X											2.3211	3.1665	1.8123	2.4893
Xenobiotic reductase	A3M9V9_ACIBT	0.056	0.3						X													X	X			X				1.1493	0.80989	0.3129	0.2132
Uncharacterized protein	A7FAX6_ACIBT	0.57	1.2																											0.51338	0.53413	0.46806	0.81066
Probable septum site-determining protein MinC	MINC_ACIBT	0.13	0.6	X	X	X																								1.3229	1.7353	0.77549	1.0591
Probable transcriptional regulatory protein A1S_1496	Y1496_ACIBT	0.21	0.8	X	X				X					X								X				X				1.5866	1.313	1.0667	1.2425
RNA polymerase-binding transcription factor DksA	A3M1C9_ACIBT	0.23	0.09	X										X								X				X				10.748	3.2645	0.63713	0.66546
Uncharacterized protein	A3MAB9_ACIBT	0.16	0.2						X														X			X				0.96668	2.0099	0.45963	0.19903
NADP+-dependent succinate semialdehyde dehydrogenase	A3M9S5_ACIBT	0.26	0.7		X				X														X			X				0.24336	0.16289	0.11082	0.15453
Putative GGDEF family protein	A3M7N0_ACIBT	0.043	1.4	X	X								X													X				0.0705	0.07335	0.10338	0.09252
Uncharacterized protein	A3MAA8_ACIBT	0.072	1.6	X	X				X													X				X				0.55998	0.41294	0.82114	0.74142
Glucose dehydrogenase	A3M8L0_ACIBT	0.0076	0.2						X																					3.7079	3.2728	0.70787	0.4372
Putative peptide signal	A3M9R8_ACIBT	0.32	3.3																											1.7289	6.5466	20.763	6.9094
Vitamin B12 receptor	A3M8N9_ACIBT	0.35	0.6				X	X									X									X	X			0.17896	0.32756	0.17034	0.15564
L-sorbosone dehydrogenase	A3M6T3_ACIBT	0.78	0.9						X													X	X			X				0.16475	0.0826	0.11641	0.10461
Uncharacterized protein	A3M936_ACIBT	0.11	6.9																											0.32516	0.3383	2.9846	1.5746
Putative outer membrane protein A	A3M7X4_ACIBT	0.42	INF																											0	0	0	0.05666
Surface adhesion protein putative	A3M7G7_ACIBT	0.42	INF																											0	0	0	0.06621
ABC-type Fe3+ transport system	A3M4E3_ACIBT-DECOY	1	INF																											0	0	0	0
Uncharacterized protein	A7FAX8_ACIBT	0.06	8.9																											0.12626	0.13136	0.8845	1.4051
L-aspartate oxidase	A3M7P9_ACIBT	0.17	0		X				X					X									X			X				0.13185	0.37578	0	0
Proline--tRNA ligase	SYP_ACIBT	0.26	0	X	X				X					X								X	X			X				0.06171	0.28023	0	0
Uncharacterized protein	A3M4C0_ACIBT	0.055	0																											0.23426	0.38519	0	0

Identified Proteins (1398)	Accession Number	T-Test (p-value): (p < 0.05)	Fold Change Untreated vs. Treated	Biological Regulation	Cellular Process	Developmental Process	Establishment of Localization	Localization	Metabolic Process	Multi-organisim Process	Reproduction	Reproductive Process	Response to Stimulus	Cytoplasm	Extracellular Region	Intracellular Organelle	Membrane	Organelle Part	Plasma Membrane	Ribosome	Antioxidant Activity	Binding	Catalytic Activity	Electron Carrier Activity	Enzyme Regulator Activity	Molecular Function	Molecular Transducer Activity	Structural Molecule Activity	Transporter Activity	Normalized emPAI Values			
																														Untreated 1	Untreated 2	Treated 1	Treated 2
Putative outer membrane protein W	A3M1G8_ACIBT	0.93	1.1														X													1.7929	2.3988	0.99464	3.4676
Lipopolysaccharide export system protein LptA	A3M423_ACIBT	0.61	1.6				X	X														X				X				1.5403	1.6026	3.9084	0.97804
50S ribosomal protein L3 glutamine methyltransferase	A3M5C6_ACIBT	0.028	0.5		X				X													X	X			X				1.1918	1.24	0.71056	0.51107
Uncharacterized protein	A3M5Y5_ACIBT	0.042	1.6																											0.56371	0.44829	0.8266	0.77778
Putative mechanosensitive ion channel	A3M6G2_ACIBT	0.3	0.6		X		X	X					X				X									X			X	0.61397	0.39259	0.25554	0.38985
CsuA/B	A3M6V1_ACIBT	0.13	0.6																											4.7466	4.9384	2.4408	3.7822
Putative ATP-dependent protease	A3M7F4_ACIBT	0.1	2.2						X													X	X			X				0.3824	0.39786	1.0435	0.70876
Putative membrane protein	A3M7R6_ACIBT	0.77	0.9																											4.435	3.4151	4.8132	2.0842
50S ribosomal protein L19	RL19_ACIBT	0.69	1.1		X				X					X		X				X						X		X		5.1821	5.3915	7.5988	4.4351
50S ribosomal protein L6	RL6_ACIBT	0.94	1		X				X					X		X				X		X				X		X		1.5797	2.8556	2.3164	2.2275
Uncharacterized protein	A7FBU6_ACIBT	0.031	5.9																											0.30655	0.51165	2.7451	2.0553
Urease accessory protein UreG	UREG_ACIBT	0.033	3						X					X								X	X			X				0.39518	0.41115	1.3761	1.0701
Cytochrome d terminal oxidase polypeptide subunit I	A3M607_ACIBT	0.00099	0.2														X													0.37842	0.39372	0.0978	0.08738
Nucleoid-associated protein A1S_1684	Y1684_ACIBT	0.17	0.5											X		X						X				X				1.4231	0.84188	0.50937	0.5138
Histidine kinase	A3M8U9_ACIBT	0.26	6	X	X				X				X				X					X	X			X	X			0.07558	0	0.11082	0.34472
Uncharacterized protein	A3M8D0_ACIBT	0.014	0.2																											0.42824	0.44555	0.13753	0.05955
Glutaryl-CoA dehydrogenase	A3M2L7_ACIBT	0.41	0.4						X													X	X			X				0.08852	0.19192	0	0.11713
Ribonuclease H	A3M3J2_ACIBT	0.5	0.6		X				X					X								X	X			X				0.30607	0.61659	0.10172	0.4451
Tryptophan synthase beta chain	A3M8N7_ACIBT	0.077	0.2		X				X														X			X				0.28609	0.41384	0	0.1162
Uncharacterized protein	A3M1Q3_ACIBT	0.65	0.7						X														X			X				3.0364	1.6116	0.88338	2.5865
Ribose-phosphate pyrophosphokinase	A3M2X3_ACIBT	0.44	0.6		X				X					X								X	X			X				0.90731	0.39932	0.35549	0.45367
Transcriptional regulator	A3M6K5_ACIBT	0.33	2.7	X	X				X													X				X				0.23746	0.54974	1.6014	0.56184
Type II secretion system protein L	A3M6Y4_ACIBT	0.5	1.4				X	X									X									X			X	0.30534	0.57906	0.81613	0.44389
Putative lipid A biosynthesis lauroyl acyltransferase	A3M7Y2_ACIBT	0.04	3.6		X				X								X						X			X				0.2281	0.37459	1.2361	0.94665
DNA repair protein radA	A3M807_ACIBT	0.56	0.8		X				X				X									X	X			X				0.35254	0.72032	0.37342	0.43961
Putative esterase	A3M8C5_ACIBT	0.94	1						X																					0.77038	0.42752	0.60254	0.62349
Putative signal peptide	A3M8M3_ACIBT	0.88	1.1																											1.6342	6.0357	4.7494	3.6836
Glutamyl-tRNA synthetase	A3M9K9_ACIBT	0.75	0.9		X				X					X								X	X			X				0.40119	0.30012	0.42299	0.18316
D-alanine--D-alanine ligase	DDL_ACIBT	0.67	0.8	X	X				X					X								X	X			X				1.4242	0.7746	1.0917	0.74484
Anthranilate phosphoribosyltransferase	TRPD_ACIBT	0.76	1.1		X				X													X	X			X				0.22155	0.36333	0.32488	0.31091
Uncharacterized protein	A7FAV6_ACIBT	0.95	1																											0.13983	0.14548	0.20504	0.08879
Histidine ammonia-lyase	A3MA50_ACIBT	0.44	2.6		X				X					X									X			X				0.1482	0.07456	0.10509	0.46346
Putative FAD/FMN-containing dehydrogenase	A3M9F4_ACIBT	0.048	0.2						X													X	X			X				0.65045	0.4492	0.10986	0.09852
24-dienoyl-CoA reductase	A3M5Y8_ACIBT	0.053	0.1						X													X	X			X				0.22784	0.30392	0.0775	0
Uncharacterized protein	A7FBU7_ACIBT	0.029	INF																											0	0	0.1379	0.19559
Phosphogluconate dehydratase	A3M1Z4_ACIBT	0.26	0.7		X				X														X			X				0.33289	0.26927	0.17936	0.26106
Putative hemolysin	A3M4A5_ACIBT	0.87	0.9																											0.9851	1.5468	1.4445	0.94402
Putative pyruvate decarboxylase	A3M7H4_ACIBT	0.069	0.4						X													X	X			X				0.19765	0.28241	0.09113	0.08125
Elongation factor P	EFP_ACIBT	0.48	0.9		X				X					X								X				X				1.8367	1.9109	1.4828	1.8999
Co-chaperone protein HscB homolog	HSCB_ACIBT	0.73	0.9		X																	X				X				1.9472	2.0259	2.151	1.5994
50S ribosomal protein L17	RL17_ACIBT	0.76	0.9		X				X					X		X				X						X		X		3.5674	3.7116	2.595	4.1333
YcgL domain-containing protein A1S_1688	Y1688_ACIBT	0.34	1.9																											7.4162	5.4471	7.6771	17.021
Putative phosphotransferase	A3M5V5_ACIBT	0.08	5						X														X			X				0.19002	0.1977	1.2151	0.74164

Identified Proteins (1398)	Accession Number	T-Test (p-value): (p < 0.05)	Fold Change Untreated vs. Treated	Biological Regulation	Cellular Process	Developmental Process	Establishment of Localization	Localization	Metabolic Process	Multi-organisim Process	Reproduction	Reproductive Process	Response to Stimulus	Cytoplasm	Extracellular Region	Intracellular Organelle	Membrane	Organelle Part	Plasma Membrane	Ribosome	Antioxidant Activity	Binding	Catalytic Activity	Electron Carrier Activity	Enzyme Regulator Activity	Molecular Function	Molecular Transducer Activity	Structural Molecule Activity	Transporter Activity	Normalized emPAI Values			
																														Untreated 1	Untreated 2	Treated 1	Treated 2
Uncharacterized protein	A7FBP1_ACIBT	0.34	2.2																											0.09916	0.21603	0.47851	0.2072
Uracil-DNA glycosylase	UNG_ACIBT	0.63	0.8		X				X				X	X									X			X				1.2693	1.3206	0.46592	1.5208
Methylenetetrahydrofolate reductase	A3M763_ACIBT	0.051	0.3		X				X					X									X			X				1.0539	1.0965	0.18928	0.52318
Transcription termination/antitermination protein NusG	A3M1F8_ACIBT	0.014	0.3	X	X				X																					4.8118	4.0438	1.0412	1.2054
Ribonuclease PH	RNPH_ACIBT	0.15	0.3		X				X													X	X			X				1.0102	1.7134	0.2224	0.64144
NAD-dependent malic enzyme	A3M129_ACIBT	0.29	0.4		X				X													X	X			X				0.35171	0.13387	0.09163	0.0817
Putative transport protein	A3M4W8_ACIBT	0.029	0.08				X	X	X								X					X	X			X			X	0.2473	0.18781	0	0.03621
Glutathione synthetase	A3M9Y2_ACIBT	0.43	0.4		X				X					X								X	X			X				0.23746	0.54974	0	0.33551
Putative phospholipid/glycerol acyltransferase	A3M321_ACIBT	0.4	7.6						X														X			X				0.11162	0.11613	0.16367	1.5699
Putative NADH pyrophosphatase	A3M3J0_ACIBT	0.023	0.1						X													X	X			X				1.18	1.5377	0.20617	0.08928
Uncharacterized protein	A3M5J3_ACIBT	0.3	0.4																											0.73501	0.76472	0	0.62733
VirP protein	A3MA44_ACIBT	0.66	0.7						X														X			X				0.13618	0.14168	0	0.18411
Uncharacterized protein	A7FBU1_ACIBT	0.046	INF																											0	0	1.0709	0.68105
Ribosome-recycling factor	RRF_ACIBT	0.027	0		X				X					X																1.0256	0.72903	0	0
Peptidyl-prolyl cis-trans isomerase	A3M0U8_ACIBT	0.63	1.5		X				X								X						X			X				0.34275	0.35661	0.81219	0.21764
Uncharacterized protein	A3M116_ACIBT	0.45	0.8																											5.1821	3.9365	2.7232	4.4351
Putative oxidoreductase/dehydrogenase	A3M8A9_ACIBT	0.074	0.4						X														X			X				2.0917	1.4703	0.67976	0.7181
Putative RNA binding protein	A3M9M4_ACIBT	0.77	0.9																											1.3893	1.0521	0.61793	1.5023
Putative glutathione S-transferase	A3MAA5_ACIBT	0.018	5.3						X														X			X				0.37806	0.64009	2.9724	2.4287
Monofunctional biosynthetic peptidoglycan transglycosylase	MTGA_ACIBT	0.78	0.9	X	X				X								X		X				X			X				0.14906	0.3321	0.21858	0.20268
Aldehyde dehydrogenase	A3M1W0_ACIBT	0.81	1.1		X				X														X			X				0.07272	0.15653	0.10663	0.14829
Uncharacterized protein	A3M1H1_ACIBT	0.00091	INF																											0	0	0.26906	0.25329
4-hydroxy-3-methylbut-2-en-1-yl diphosphate synthase (flavodoxin)	ISPG_ACIBT	0.022	4.2		X				X													X	X			X				0.09748	0.10142	0.4697	0.37225
Putative flavohemoprotein	A3M988_ACIBT	0.045	0.1				X	X	X													X	X			X			X	1.7624	1.1953	0.20207	0.18644
Uncharacterized protein	A3M922_ACIBT	0.57	0.3																											0.20045	0	0	0.06087
Cytidylate kinase	KCY_ACIBT	0.0081	0.05		X				X					X								X	X			X				3.8708	3.3573	0	0.34986
Putative tRNA hydroxylase	A3M7N8_ACIBT	0.54	4.1																											0	0.16732	0	0.6919
Putative homocysteine S-methyltransferase family protein	A3M360_ACIBT	0.95	1		X				X					X								X	X			X				0.78054	0.81208	1.1445	0.49561
Uncharacterized protein	A3M479_ACIBT	0.46	1.2																											0.14632	0.23611	0.21456	0.257
GCN5-related N-acetyltransferase	A3M4A2_ACIBT	0.074	1.8						X														X			X				0.80096	1.2334	1.7384	1.8331
50S ribosomal protein L24	RL24_ACIBT	0.76	0.8		X				X					X		X				X		X				X		X		3.7464	15.697	5.4935	9.5796
D-alanyl-D-alanine endopeptidase penicillin-binding protein 7 and penicillin-binding protein 8	A3M1B8_ACIBT	0.00035	2.3						X														X			X				0.22514	0.23424	0.52077	0.52696
D-3-phosphoglycerate dehydrogenase	A3M9F5_ACIBT	0.14	0.5		X				X													X	X			X				0.29007	0.19294	0.13046	0.11775
50S ribosomal protein L27	RL27_ACIBT	0.47	1.5		X				X					X		X				X						X		X		8.5186	8.8629	18.729	8.1099
Putative membrane protein	A3M8G4_ACIBT	0.62	1.4																											0.04938	0.10517	0.14822	0.06418
50S ribosomal protein L31	RL31_ACIBT	0.63	1.2		X				X					X		X				X		X				X		X		2.4352	1.3393	1.8876	2.6325
Cytochrome o ubiquinol oxidase subunit I	A3M6Q0_ACIBT	0.17	0.6		X		X	X	X								X					X	X	X		X			X	0.22525	0.17145	0.07668	0.14303
L-24-diaminobutyrate:2-ketoglutarate 4-aminotransferase	A3M7H8_ACIBT	0.6	0.8						X													X	X			X				0.36075	0.17389	0.24507	0.16533
Beta-hexosaminidase	A3M201_ACIBT	0.069	0.2	X	X				X					X									X			X				0.35515	0.3695	0.15709	0
Macrolide export ATP-binding/permease protein MacB	A3M245_ACIBT	0.048	5.5				X	X	X				X				X		X			X	X			X			X	0	0.05699	0.16481	0.15034
Signal recognition particle receptor FtsY	A3M613_ACIBT	0.19	0.2		X		X	X						X			X		X			X	X			X				1.5295	0.62029	0.14499	0.20656
Putative dyp-type peroxidase	A3M130_ACIBT	0.55	1.8						X												X	X	X			X				0.24159	0.25135	0.168	0.70876
Putative peptidoglycan-binding LysM	A3M2W4_ACIBT	0.0017	0.4																											3.408	3.5457	1.3232	1.2068

Identified Proteins (1398)	Accession Number	T-Test (p-value): (p < 0.05)	Fold Change Untreated vs. Treated	Biological Regulation	Cellular Process	Developmental Process	Establishment of Localization	Localization	Metabolic Process	Multi-organisim Process	Reproduction	Reproductive Process	Response to Stimulus	Cytoplasm	Extracellular Region	Intracellular Organelle	Membrane	Organelle Part	Plasma Membrane	Ribosome	Antioxidant Activity	Binding	Catalytic Activity	Electron Carrier Activity	Enzyme Regulator Activity	Molecular Function	Molecular Transducer Activity	Structural Molecule Activity	Transporter Activity	Normalized emPAI Values			
																														Untreated 1	Untreated 2	Treated 1	Treated 2
Uncharacterized protein	A7FAZ3_ACIBT	0.14	3																											0.24426	0.12045	0.68027	0.40042
Putative acyltransferase	A3M217_ACIBT	0.034	3.5						X														X			X				0.13565	0.14113	0.4234	0.55664
Putative ABC transporter ATP-binding protein	A3M546_ACIBT	0.0008	INF				X	X	X													X	X			X				0	0	0.28128	0.26575
Putative acetyl-CoA synthetase/AMP-(Fatty) acid ligase	A3M6N1_ACIBT	0.42	INF						X														X			X				0	0	0	0.08341
Uncharacterized protein	A3M2D1_ACIBT	0.013	INF																											0	0	0.54871	0.69047
Histidine kinase	A3M9M6_ACIBT	0.078	0.1	X	X				X				X				X					X	X			X	X			0.1439	0.23206	0	0.04422
Uncharacterized protein	A3M5A7_ACIBT	0.27	1.5																											0.40301	0.59205	0.83443	0.60959
Putative signal peptide	A3M8I8_ACIBT	0.24	0.6																											0.45751	0.67679	0.41992	0.2905
Putative penicillin binding protein (PonA)	A3M9J6_ACIBT	0.65	1.3																			X				X				0.33362	0.63756	0.89857	0.3891
Putative two-component response regulator	A3M9U9_ACIBT	0.068	0.8	X	X				X				X									X				X				1.9243	2.0021	1.7003	1.5243
Uncharacterized protein	A3M9Z4_ACIBT	0.94	1																											1.8983	2.5502	2.7836	1.5564
Uncharacterized protein	A7FBS8_ACIBT	0.099	1.6		X				X																					0.41746	0.61441	0.86595	0.78849
Putative transcriptional regulator (AraC family)	A3M8L3_ACIBT	0.7	0.8	X	X				X													X				X				0.40235	0.19243	0.13013	0.33332
Putative ferrous iron transport protein B	A3M1C4_ACIBT	0.073	1.7				X	X									X					X				X			X	0.18305	0.12355	0.26842	0.25264
DNA polymerase III delta prime subunit	A3M4Z1_ACIBT	0.53	0.9																											0.51672	0.5376	0.53908	0.43269
Putative acyltransferase	A3M8Z9_ACIBT	0.025	3.6						X														X			X				0.63584	0.29193	1.5919	1.7815
30S ribosomal protein S18	RS18_ACIBT	0.68	1.2		X				X					X		X				X		X				X		X		2.1627	2.2501	1.7064	3.6275
Uncharacterized protein	A3M0V1_ACIBT	0.37	0.8		X				X														X			X				2.0009	2.8355	2.0754	1.7305
Putative signal peptide	A3M9D0_ACIBT	0.032	0.5																											1.0515	1.094	0.4929	0.66769
Uncharacterized protein	A3M2E9_ACIBT	0.17	6.7																											0.27182	0.1333	1.9043	0.8246
Putative signal peptide	A3M1G9_ACIBT	0.18	2.3																											0.31876	1.326	1.8689	1.8385
Hca cluster transcriptional activator (LysR family)	A3M1S1_ACIBT	0.3	2	X	X				X													X				X				0.7322	0.12202	0.81144	0.88713
Uncharacterized protein	A3M351_ACIBT	0.0025	INF																											0	0	0.13497	0.12199
Putative acetyl-CoA acetyltransferase	A3M5G2_ACIBT	0.078	3.9						X														X			X				0.09667	0.10057	0.29649	0.46374
Uncharacterized protein	A7FAW9_ACIBT	0.033	0.4																											0.65612	0.68264	0.15709	0.31644
Uncharacterized protein	A7FAZ2_ACIBT	0.52	0.7																											0.19212	0.09576	0.13497	0.05845
Uncharacterized protein	A3M6R3_ACIBT	0.14	4.1																											0.13871	0.06993	0.55976	0.30052
Putative lysophospholipase	A3M6D0_ACIBT	0.98	1																											0.09831	0.10229	0	0.20528
Uncharacterized protein	A3M9Q2_ACIBT	0.65	0.7																											0.1055	0.10976	0	0.14068
Putative thiol:disulphide interchange protein (DsbC-like)	A3M8I7_ACIBT	0.085	0.2																											1.0102	1.36	0	0.47659
Lipid A export ATP-binding/permease protein MsbA	A3M4Y8_ACIBT	0.056	0				X	X	X								X		X			X	X			X			X	0.26574	0.43984	0	0
Uncharacterized protein	A3M5J5_ACIBT	0.023	10																			X				X				0	0.09919	0.45846	0.55766
Methionyl-tRNA formyltransferase	FMT_ACIBT	0.0091	0.04		X				X														X			X				1.1113	0.93606	0	0.07251
Argininosuccinate synthetase	A3M3K6_ACIBT	0.00039	0		X				X													X	X			X				2.9921	3.113	0	0
Response regulator	A3M1B7_ACIBT	0.74	1.2	X	X				X				X									X				X				1.5781	0.63638	1.2994	1.2774
Methylated-DNA--protein-cysteine methyltransferase	A3M2L1_ACIBT	0.041	2.3		X				X				X	X									X			X				1.2682	1.3195	2.603	3.288
Putative lipoprotein	A3M3E7_ACIBT	0.46	1.9																											2.0046	2.0857	6.0941	1.8655
Uncharacterized protein	A3M480_ACIBT	0.29	0.9																											1.1665	1.2136	1.1571	1.0293
ATP synthase epsilon chain	ATPE_ACIBT	0.25	1.5		X		X	X	X								X		X			X	X			X			X	3.327	2.4981	3.5209	5.0014
50S ribosomal protein L30	RL30_ACIBT	0.29	1.3		X				X					X		X		X		X						X		X		6.3673	6.6246	9.3367	7.0062
Putative siderophore biosynthesis protein putative acetyltransferase	A3M590_ACIBT	0.6	1.2						X														X			X				0.56882	0.59181	0.8341	0.52059
Membrane-bound lytic murein transglycosylase B	A3M744_ACIBT	0.25	0.5																											0.49836	0.3695	0.33014	0.06802
Tyrosine aminotransferase tyrosine repressible, PLP-dependent	A3M0X2_ACIBT	0.097	0.4		X				X													X	X			X				0.51569	0.4115	0.26694	0.11559

Identified Proteins (1398)	Accession Number	T-Test (p-value): (p < 0.05)	Fold Change Untreated vs. Treated	Biological Regulation	Cellular Process	Developmental Process	Establishment of Localization	Localization	Metabolic Process	Multi-organisim Process	Reproduction	Reproductive Process	Response to Stimulus	Cytoplasm	Extracellular Region	Intracellular Organelle	Membrane	Organelle Part	Plasma Membrane	Ribosome	Antioxidant Activity	Binding	Catalytic Activity	Electron Carrier Activity	Enzyme Regulator Activity	Molecular Function	Molecular Transducer Activity	Structural Molecule Activity	Transporter Activity	Normalized emPAI Values: Untreated 1	Untreated 2	Treated 1	Treated 2
Ribosomal RNA small subunit methyltransferase G	RSMG_ACIBT	0.59	0.7		X				X					X									X			X				1.1595	0.61502	0.24728	0.96109
3-dehydroquinate synthase	AROB_ACIBT	0.0097	0.3		X				X					X									X			X				0.759	0.78968	0.30631	0.20851
Putative VGR-related protein	A3M472_ACIBT	0.44	1.6																											0.16794	0.17473	0.38373	0.16616
Uncharacterized protein	A3M991_ACIBT	0.62	1.4																											0.10813	0.23654	0.33338	0.14436
Uncharacterized protein	A3M1L7_ACIBT	0.18	0.2																											0.31204	0.15182	0	0.09266
Uncharacterized protein	A7FBR8_ACIBT	0.78	0.9																											0.09587	0.20855	0.14057	0.12728
Uncharacterized protein	A3M937_ACIBT	0.15	0.3																											0.52886	0.78926	0	0.33581
Putative membrane protein	A3M320_ACIBT	0.14	1.7																											0.09694	0.10086	0.14215	0.20215
Uncharacterized protein	A7FAV4_ACIBT	0.03	INF																											0	0	0.50759	0.35543
S-adenosylmethionine:tRNA ribosyltransferase-isomerase	QUEA_ACIBT	0.17	0		X				X					X									X			X				0.61925	0.22269	0	0
Uncharacterized protein	A3M8F5_ACIBT	0.11	0																											0.10119	0.22065	0	0
Delta-aminolevulinic acid dehydratase	A3M344_ACIBT	0.0012	0.03		X				X													X	X			X				1.2067	1.2555	0	0.06699
Putative signal peptide	A3M7L5_ACIBT	0.22	2.8																											0.08943	0	0.13114	0.11839
Putative RND family drug transporter	A3M247_ACIBT	0.055	0.2				X	X									X									X			X	0.42594	0.44315	0	0.16994
N-succinylarginine dihydrolase	ASTB_ACIBT	0.2	0.2		X				X														X			X				0.07867	0.16979	0	0.04995
Taurine dioxygenase	A3M4M9_ACIBT	0.77	0.6						X														X			X				0	0.26229	0	0.16008
Glycine/D-amino acid oxidases (Deaminating)	A3M3M0_ACIBT	0.12	0.06						X														X			X				0.52398	0.24518	0	0.04657
Putative FAD-dependent monooxygenase	A3M3H9_ACIBT	0.1	2		X				X													X	X			X				0.39218	0.18792	0.57507	0.59065
Putative ATP-dependent protease	A3M7F3_ACIBT	0.17	1.9						X														X			X				0.24075	0.55815	0.78665	0.70564
Esterase	A3M3I9_ACIBT	0.015	4.2						X														X			X				0.2521	0.12412	0.82773	0.7483
Homoserine dehydrogenase	A3M1C0_ACIBT	0.19	1.8		X				X													X	X			X				0.27335	0.39472	0.72423	0.47691
Peptidyl-prolyl cis-trans isomerase	A3M375_ACIBT	0.074	0.4		X				X														X			X				3.0727	2.1713	1.1226	0.84874
Uncharacterized protein	A3M146_ACIBT	0.21	0.4																											1.2793	2.5152	0.7541	0.81233
Outer-membrane lipoprotein carrier protein	A3M898_ACIBT	0.74	1.1				X	X																		X			X	0.33941	0.57027	0.4977	0.5004
Uncharacterized protein	A7FBR6_ACIBT	0.67	1.1																											0.50355	0.32612	0.45963	0.45722
Gamma-glutamyl phosphate reductase	PROA_ACIBT	0.3	2.6		X				X					X								X	X			X				0.0861	0.08958	0.12625	0.32177
DNA replication and repair protein RecF	RECF_ACIBT	0.041	11		X				X				X	X								X				X				0	0.09974	0.64372	0.45901
Trehalose-6-phosphate synthase	A3M2U7_ACIBT	0.028	0.1		X				X														X			X				0.31579	0.32856	0	0.0937
Uncharacterized protein	A3M2E6_ACIBT	0.18	3.1																											0	0.15895	0.22402	0.26949
Transposition site target selection protein D	A3M7S6_ACIBT	0.013	9.9																											0.11344	0	0.55466	0.56652
Uncharacterized protein	A3M1A6_ACIBT	0.062	0.1																											1.8699	1.1763	0.24256	0.10503
Phosphate-specific transport system accessory protein PhoU	A3M1D7_ACIBT	0.55	3.4	X	X		X	X						X																0.15036	0.15643	0	1.0326
Cysteine--tRNA ligase	SYC_ACIBT	0.11	0		X				X					X								X	X			X				0.07226	0.15552	0	0
Uncharacterized protein	A3M2D6_ACIBT	0.24	5.1																											0	0.09974	0.14057	0.36496
Lipid A biosynthesis lauroyltransferase	A3M1U2_ACIBT	0.044	7		X				X								X						X			X				0.10881	0	0.33558	0.4245
Putative epimerase	A3M7J5_ACIBT	0.065	INF						X														X			X				0	0	0.91182	0.52562
Glutamine synthetase	A3M777_ACIBT	0.072	INF		X				X													X	X			X				0	0	1.3178	0.73337
Glutamyl-tRNA reductase	HEM1_ACIBT	0.11	8.7		X				X													X	X			X				0.08182	0	0.24928	0.46019
Oxygen-insensitive NADPH nitroreductase	A3M6J4_ACIBT	0.043	0.03						X														X			X				2.7284	4.1307	0	0.2198
Merops peptidase family S24	A3M3T0_ACIBT	0.14	0.09						X								X					X	X			X				0.6505	0.29794	0	0.08546
Transposase	A3M191_ACIBT	0.016	1.4																			X				X				0.69531	0.72341	1.0196	0.95485
Putative membrane protein	A3M2R7_ACIBT	0.022	0.4																											0.66007	0.68675	0.30919	0.18422
Cysteine desulfurase	A3M566_ACIBT	0.0088	0.6						X													X	X			X				0.28637	0.29794	0.19736	0.18183

Identified Proteins (1398)	Accession Number	T-Test (p-value): ($p < 0.05$)	Fold Change Untreated vs. Treated	Biological Regulation	Cellular Process	Developmental Process	Establishment of Localization	Localization	Metabolic Process	Multi-organisim Process	Reproduction	Reproductive Process	Response to Stimulus	Cytoplasm	Extracellular Region	Intracellular Organelle	Membrane	Organelle Part	Plasma Membrane	Ribosome	Antioxidant Activity	Binding	Catalytic Activity	Electron Carrier Activity	Enzyme Regulator Activity	Molecular Function	Molecular Transducer Activity	Structural Molecule Activity	Transporter Activity	Normalized emPAI Values Untreated 1	Untreated 2	Treated 1	Treated 2
Putative ATPase	A3M5C3_ACIBT	0.79	0.9																			X				X				0.77038	0.60427	0.85165	0.36878
Putative phosphotyrosine protein phosphatase	A3M6D4_ACIBT	0.44	1.9		X				X														X			X				1.5664	1.1757	3.9852	1.3269
Uncharacterized protein	A7FAW1_ACIBT	0.0022	2																											0.54248	0.5644	1.1429	1.094
Uncharacterized protein	A7FB56_ACIBT	0.91	1																											1.7103	1.275	1.7971	1.086
Putative transcriptional regulator (TetR family)	A3M257_ACIBT	0.044	0.9	X	X				X													X				X				0.64134	0.66726	0.57621	0.59201
DNA-binding protein HU-beta	A3M570_ACIBT	0.11	0.4		X																	X				X				8.067	8.393	1.6279	5.1223
Uncharacterized protein	A3M1Y0_ACIBT	0.35	3																											0.1858	0.42067	1.4131	0.41995
Putative phosphoenolpyruvate synthase regulatory protein	PSRP_ACIBT	0.057	0.5		X				X													X	X			X				1.0337	1.0755	0.39549	0.65638
Putative peroxidase	A3M5Z0_ACIBT	0.14	1.9						X												X		X			X				0.1109	0.2429	0.34235	0.32931
Acetoacetyl-CoA transferase alpha subunit	A3M5G5_ACIBT	1	1						X														X			X				0.79247	0.8245	0.50014	1.116
Thymidylate synthase	A3M1W9_ACIBT	0.043	0.2		X				X					X									X			X				0.59303	0.81929	0.18248	0.16736
Uncharacterized protein	A7FAW3_ACIBT	0.97	1																											0.47265	0	0.31454	0.1362
Putative membrane protein	A3M1E7_ACIBT	0.77	1.1																											0.17211	0.08615	0.12142	0.17044
CysI-like sulfite reductase protein	A3M8K8_ACIBT	0.19	0						X													X	X			X				0.20031	0.06548	0	0
Putative enoyl-CoA hydratase II	A3M4C6_ACIBT	0.2	0.2		X				X				X										X			X				0.1378	0.30548	0	0.0875
Uncharacterized protein	A3MAB1_ACIBT	0.22	2.7																											0	0.19862	0.27994	0.26439
NAD/NADP-dependent betaine aldehyde dehydrogenase	BETB_ACIBT	0.19	0		X				X													X	X			X				0.23727	0.07679	0	0
WecE protein	A3M0V6_ACIBT	0.37	0.5						X													X	X			X				0.32941	0.79251	0.14666	0.38376
Serine acetyltransferase	A3M4P7_ACIBT	0.16	3.6		X				X					X									X			X				0.13304	0	0.19509	0.28675
Ferredoxin--NADP+ reductase	A3M2R5_ACIBT	0.016	0.1						X													X	X			X				0.90785	0.94454	0.20451	0
Putative MutT/nudix family protein	A3M2J7_ACIBT	0.085	5.9						X														X			X				0.60466	0.17893	1.7449	2.8586
Phage major capsid protein HK97	A3M524_ACIBT-DECOY	1	INF																											0	0	0	0
Uncharacterized protein	A7FBL1_ACIBT	0.01	13																											0	0.12286	0.81788	0.73802
Putative signal peptide	A3M9Z3_ACIBT	0.78	1.1																											1.2265	0.98973	1.3949	0.97757
3-deoxy-D-manno-2-octulosonate transferase	A3M9V8_ACIBT	0.27	0.7						X														X			X				0.25909	0.17305	0.11749	0.16451
Putative arginyl-tRNA--protein transferase	ATE_ACIBT	0.32	0.6		X				X					X									X			X				0.26258	0.43433	0.18183	0.26507
Pyruvate ferredoxin/flavodoxin oxidoreductase	A3M4F2_ACIBT	0.15	0.5																											0.83218	0.5198	0.3309	0.31723
Putative lipoprotein-34 (NlpB)	A3MA69_ACIBT	0.42	1.8																											2.2295	1.0026	4.1177	1.783
Uncharacterized protein	A3M786_ACIBT	0.089	0.2																											1.364	0.81093	0.22943	0.21344
Putative membrane protein	A3M1A9_ACIBT	0.0018	2.9														X													0.09508	0.09892	0.2914	0.27614
Putative transcriptional regulator (PcaU-like)	A3M382_ACIBT	0.043	0.2	X	X				X													X				X				0.63872	0.88595	0.19434	0.17889
Chorismate synthase	AROC_ACIBT	0.25	2.2		X				X														X			X				0.10119	0.10527	0.14837	0.29641
Putative glycosyltransferase	A3M8R1_ACIBT	0.14	0.3						X														X			X				0.63259	1.0212	0.15238	0.40167
Uncharacterized protein	A3M714_ACIBT	0.54	1.6																											0.70071	0.20309	1.0275	0.44493
Putative UDP-glucose 6-dehydrogenase	A3M0W4_ACIBT	0.09	0.2						X													X	X			X				1.4167	0.83852	0.23581	0.2198
Acyl-CoA dehydrogenase	A3M5Q0_ACIBT	0.58	0.3						X													X	X			X				0.11916	0	0	0.03682
Uncharacterized protein	A3M8V6_ACIBT-DECOY	1	INF																											0	0	0	0
Uncharacterized protein	A3M4G9_ACIBT	0.22	2.8																											0.07152	0	0.10487	0.0939
Histidine kinase	A3M660_ACIBT	0.051	5.2	X	X				X				X				X					X	X			X	X			0.19759	0.09838	0.63377	0.91075
Uncharacterized protein	A3M352_ACIBT	0.09	5.3																											0.14003	0	0.43794	0.30373
Putative signal peptide	A3M3I8_ACIBT	0.098	0.03																											2.2441	1.1406	0	0.10259
Sulfurtransferase	A3MA24_ACIBT	0.16	0.3						X														X			X				0.41746	0.43433	0	0.26507
Riboflavin synthase alpha chain	A3M1A4_ACIBT	0.079	INF		X				X														X			X				0	0	2.7813	1.5012

Identified Proteins (1398)	Accession Number	T-Test (p-value): (p < 0.05)	Fold Change Untreated vs. Treated	Biological Regulation	Cellular Process	Developmental Process	Establishment of Localization	Localization	Metabolic Process	Multi-organism Process	Reproduction	Reproductive Process	Response to Stimulus	Cytoplasm	Extracellular Region	Intracellular Organelle	Membrane	Organelle Part	Plasma Membrane	Ribosome	Antioxidant Activity	Binding	Catalytic Activity	Electron Carrier Activity	Enzyme Regulator Activity	Molecular Function	Molecular Transducer Activity	Structural Molecule Activity	Transporter Activity	Normalized emPAI Values			
																														Untreated 1	Untreated 2	Treated 1	Treated 2
RNA-binding protein Hfq	A7FBM2_ACIBT	0.95	1	X																		X				X				2.6054	2.7107	3.8204	1.6543
Peptide methionine sulfoxide reductase MsrA	MSRA_ACIBT	0.39	0.7		X				X				X										X			X				1.0794	1.123	1.0772	0.46647
Transcriptional repressor NrdR	NRDR_ACIBT	0.32	0.7	X	X				X													X				X				0.84535	1.3081	0.7432	0.79832
30S ribosomal protein S14	RS14_ACIBT	0.5	1.3		X				X					X		X				X		X				X		X		5.2355	2.5114	5.3027	4.709
Transposase	A3M192_ACIBT	0.17	2.5		X				X													X				X				0.19537	0.20326	0.62603	0.35452
Uncharacterized protein	A3M874_ACIBT	0.38	3.5																											0.90124	2.6767	2.3593	10.119
23S ribosomal RNA G745 methyltransferase	A3M9J7_ACIBT	0.18	0.5						X													X	X			X				0.27182	0.45047	0.18788	0.17259
ATP-dependent dsDNA exonuclease	A3M338_ACIBT	0.2	4.1		X				X														X			X				0	0.08493	0.1197	0.23267
UbiH protein 2-octaprenyl-6-methoxyphynol hydroxylase, FAD/NAD(P)-binding	A3M3H8_ACIBT	0.39	1.4		X				X													X	X			X				0.18446	0.30012	0.27049	0.41643
Uncharacterized protein	A3M8V5_ACIBT	0.33	2.2																											0.19054	0.09501	0.4375	0.18945
Putative signal peptide	A3M647_ACIBT	0.037	0.3																											0.76705	0.60178	0.17863	0.25989
Putative signal peptide	A3M9I7_ACIBT	0.93	1																											0.25676	0.26714	0.17801	0.36576
Homoserine O-acetyltransferase	METX_ACIBT	0.036	1.4		X				X					X									X			X				0.09084	0.09451	0.1332	0.12033
Branched-chain amino acid transferase	A3M8R6_ACIBT	0.14	0.5		X				X														X			X				0.38381	0.25223	0.16856	0.15393
Methionine import ATP-binding protein MetN	A3M6S5_ACIBT	0.21	1.8				X	X	X								X		X			X	X			X			X	0.21404	0.22269	0.49393	0.2994
Putative membrane protease subunit	A3M8C7_ACIBT	0.037	0.2						X								X						X			X				0.81266	0.84551	0	0.27379
Putative phage related protein	A3M2N6_ACIBT	0.22	3.3		X				X				X									X	X			X				0.07796	0.08111	0.36891	0.15975
PaaX	A3M4D1_ACIBT	0.011	0.09		X				X																					0.35515	0.3695	0	0.06802
Heavy metal response regulator	A3M8U8_ACIBT	0.35	3.4	X	X								X									X				X				0.15369	0.1599	0.22536	0.84512
Uncharacterized protein	A3M200_ACIBT	0.03	3.5						X														X			X				0.12225	0.12719	0.37931	0.48916
1-(5-phosphoribosyl)-5-[(5-phosphoribosylamino)methylideneamino] imidazole-4-carboxamide isomera	HIS4_ACIBT	0.028	0.1		X				X					X									X			X				0.32822	0.34149	0	0.09716
Uncharacterized protein	A3M679_ACIBT	0.14	0.4		X				X																					0.39841	0.26135	0.17434	0.07549
Putative transcriptional regulator (AraC family)	A3M4W6_ACIBT	0.064	0.2	X	X				X													X				X				0.47634	0.35388	0	0.13718
Putative membrane protein	A3M891_ACIBT	0.85	1.2																											0.25302	0.12455	0.37102	0.07602
Putative phosphotransferase	A3M4Y1_ACIBT	0.13	0.09						X														X			X				0.46219	0.21865	0	0.06369
Aldehyde reductase	A3MA84_ACIBT	0.008	0.08						X														X			X				1.1022	1.1467	0	0.18035
Putative ferrous iron transport protein B	A3M2F8_ACIBT	0.68	0.6				X	X									X					X				X			X	0.11895	0.06023	0	0.11644
Uncharacterized protein	A3M8R0_ACIBT	0.16	3.6																											0.11271	0	0.16527	0.23846
Arginine biosynthesis bifunctional protein ArgJ	A3M2L2_ACIBT	0.022	0		X				X					X									X			X				0.41305	0.56127	0	0
Uncharacterized protein	A3M7X5_ACIBT-DECOY	1	INF																											0	0	0	0
Putative intracellular protease/amidase	A3M8D3_ACIBT	0.00039	0		X				X														X			X				1.2418	1.292	0	0
Uncharacterized protein	A3M457_ACIBT	0.061	5.6																											0.97617	0	3.0627	2.4196
Uncharacterized protein	A3M2C0_ACIBT	0.9	1																											0.41912	0.617	0.61459	0.37655
Cys-tRNA(Pro)/Cys-tRNA(Cys) deacylase	A3M579_ACIBT	0.34	0.6	X	X				X														X			X				1.9006	0.93883	0.78877	0.85734
Bifunctional purine biosynthesis protein PurH	A3M6S0_ACIBT	0.33	0.6		X				X														X			X				0.78808	1.437	0.80374	0.5004
Putative signal peptide	A3M9F3_ACIBT	0.63	1.3																											3.7274	1.9086	2.69	4.3562
30S ribosomal protein S16	RS16_ACIBT	0.36	1.5		X				X					X		X				X						X		X		3.4604	5.6598	7.977	5.2623
Glutamate racemase	A3MA43_ACIBT	0.79	0.9	X	X				X														X			X				0.46563	0.92113	0.68277	0.56217
Uncharacterized protein	A3M6T6_ACIBT	0.18	0.5																											0.33453	0.56151	0.2284	0.21241
Putative transcriptional regulator	A3M3E4_ACIBT	0.0086	0.4																											1.8093	1.8825	0.75966	0.54981
Putative exported protein	A3M9R4_ACIBT	0.2	2.1																											0.71009	0.44927	1.5257	0.9097
RNA polymerase sigma factor RpoH	A3M6Z1_ACIBT	0.074	0.3	X	X	X			X				X	X								X				X				0.40456	0.59445	0.17677	0.07654
Succinyl-diaminopimelate desuccinylase	DAPE_ACIBT	0.4	0.4		X				X													X	X			X				0.19987	0.09946	0	0.12691

																														Normalized emPAI Values			
Identified Proteins (1398)	Accession Number	T-Test (p-value): ($p < 0.05$)	Fold Change Untreated vs. Treated	Biological Regulation	Cellular Process	Developmental Process	Establishment of Localization	Localization	Metabolic Process	Multi-organisim Process	Reproduction	Reproductive Process	Response to Stimulus	Cytoplasm	Extracellular Region	Intracellular Organelle	Membrane	Organelle Part	Plasma Membrane	Ribosome	Antioxidant Activity	Binding	Catalytic Activity	Electron Carrier Activity	Enzyme Regulator Activity	Molecular Function	Molecular Transducer Activity	Structural Molecule Activity	Transporter Activity	Untreated 1	Untreated 2	Treated 1	Treated 2
Protein chain initiation factor IF-3	A3M2A0_ACIBT	0.062	0.5		X				X													X				X				1.2745	1.326	0.46742	0.80926
Uncharacterized protein	A3M8R4_ACIBT	0.16	0.3																											0.20764	0.21603	0	0.13184
Uncharacterized protein	A7FAY5_ACIBT	0.081	INF																											0	0	0.26906	0.50435
Amino-acid acetyltransferase	A3M0T2_ACIBT	0.054	0.3		X				X					X									X			X				0.34132	0.2568	0.11229	0.04862
Ferrochelatase	HEMH_ACIBT	0.033	1.4		X				X					X								X	X			X				0.10299	0.10715	0.15102	0.13718
Ribosomal RNA large subunit methyltransferase J	A3M924_ACIBT	0.52	0.7		X				X													X	X			X				0.25395	0.12499	0.17615	0.07628
Pseudouridine synthase	A3M2Y1_ACIBT	0.26	0.3		X				X													X	X			X				0.60856	0.21931	0.14751	0.06388
Putative chaperone	A3M7N3_ACIBT	0.76	1.4																											0.10412	0	0.07454	0.06611
Uncharacterized protein	A3M2D5_ACIBT-DECOY	1	INF																											0	0	0	0
Uncharacterized protein	A3M2E3_ACIBT	0.42	INF																											0	0	0	0.21388
Uncharacterized protein	A3M8Y9_ACIBT	0.45	0.5		X		X	X									X													0.46327	0.48199	0	0.48663
Uncharacterized protein	A7FAW0_ACIBT	0.59	3																											0	0.14628	0	0.43508
Uncharacterized protein	A3MA29_ACIBT	0.64	0.6																											0.85893	0.89365	0	1.1379
Putative dienelactone hydrolase	A3MA75_ACIBT	0.02	0.03						X														X			X				1.2918	1.692	0	0.09547
Aldehyde dehydrogenase 1	A3M6I5_ACIBT	0.42	INF						X														X			X				0	0	0	0.09291
Short-chain dehydrogenase/reductase SDR	A3M5Q2_ACIBT	0.16	0.05						X														X			X				0.41746	1.0406	0	0.07874
tRNA-dihydrouridine synthase	A3M2T5_ACIBT	0.42	3.6		X				X													X	X			X				0	0.12039	0.35799	0.07348
Hemerythrin-like metal-binding protein	A3M329_ACIBT	0.63	2.3																			X				X				0.81317	0.50892	0	3.0505
Putative oxidoreductase aldo/keto reductase family	A3M3W7_ACIBT	0.0077	0.05						X														X			X				1.3291	1.168	0	0.13304
Cell shape-determining protein MreC	A3M8E8_ACIBT	0.97	1	X																										0.12489	0.12994	0	0.2672
Protein tyrosine phosphatase	A3M2H4_ACIBT	0.13	0.5		X				X														X			X				3.6216	2.8355	2.0754	0.89868
Outer membrane protein assembly factor BamE	A3M332_ACIBT	0.048	1.6		X		X	X									X													1.7091	1.7782	2.5062	2.945
Adenine deaminase	A3M3S3_ACIBT	0.66	0.8		X				X													X	X			X				0.34348	0.65816	0.50367	0.30558
Universal stress protein	A3M6F5_ACIBT	0.53	0.8										X	X																2.8905	3.0073	3.0966	1.8353
Putative RND family drug transporter	A3M7Z2_ACIBT	0.11	2.1				X	X																		X			X	0.3052	0.50927	0.71776	0.96043
30S ribosomal protein S17	RS17_ACIBT	0.95	1		X				X					X		X				X		X				X		X		3.2541	3.3856	4.7717	2.0662
Putative protease putative signal peptide peptidase sppA	A3M7Y1_ACIBT	0.28	1.8						X														X			X				0.44885	0.46699	1.0859	0.57586
Ribonuclease 3	RNC_ACIBT	0.59	1.5		X				X					X								X	X			X				0.5236	0.78091	1.4813	0.47659
30S ribosomal protein S21	RS21_ACIBT	0.2	0.6		X				X					X		X				X						X		X		6.5119	4.2109	3.4979	2.5699
Glutamate racemase	A3M1P5_ACIBT	0.15	2.5	X	X				X														X			X				0.26559	0.27632	0.87698	0.50448
tRNA modification GTPase MnmE	MNME_ACIBT	0.017	1.4		X				X					X								X	X			X				0.16593	0.17264	0.24331	0.2273
Putative lipoprotein (VacJ) transmembrane	A3M2C7_ACIBT	0.24	2.8														X													0.25029	0.41294	0.582	1.2725
Electron transfer flavoprotein alpha-subunit	A3M800_ACIBT	0.93	1																			X		X		X				1.0046	0.7768	1.0948	0.63786
Uncharacterized protein	A3M865_ACIBT	0.35	1.6																											0.05456	0.03753	0.05289	0.09642
50S ribosomal protein L23	RL23_ACIBT	0.85	0.9		X				X					X		X				X		X				X		X		2.3769	1.561	2.2	1.5092
Probable GTP-binding protein EngB	ENGB_ACIBT	0.046	0.5		X																	X	X			X				0.58783	0.61159	0.24608	0.37325
Ribosomal protein L22	A3M350_ACIBT	0.62	0.8											X		X				X										0.79499	0.49848	0.70256	0.30422
Putative hydrolase	A3M2W6_ACIBT	0.19	0.6						X														X			X				0.79691	0.57627	0.5026	0.35169
Putative nucleoprotein/polynucleotide-associated enzyme	A3M4W9_ACIBT	0.088	0.4																											1.048	1.0904	0.29115	0.66546
Uncharacterized protein	A3M6S1_ACIBT	0.092	0.4		X				X														X			X				0.69773	0.72592	0.44696	0.19354
Formamidopyrimidine-DNA glycosylase	A3M8J3_ACIBT	0.28	0.6		X				X				X									X	X			X				0.43119	0.2817	0.18719	0.27379
Dihydrolipoamide acetyltransferase	A3M5D4_ACIBT	0.13	1.7						X														X			X				0.07412	0.07712	0.10869	0.15135
Putative UDP-glucose 4-epimerase	A3M0W6_ACIBT	0.22	0.2						X													X	X			X				1.0172	0.36825	0.15661	0.06781

																														Normalized emPAI Values			
Identified Proteins (1398)	Accession Number	T-Test (p-value): (p < 0.05)	Fold Change Untreated vs. Treated	Biological Regulation	Cellular Process	Developmental Process	Establishment of Localization	Localization	Metabolic Process	Multi-organisim Process	Reproduction	Reproductive Process	Response to Stimulus	Cytoplasm	Extracellular Region	Intracellular Organelle	Membrane	Organelle Part	Plasma Membrane	Ribosome	Antioxidant Activity	Binding	Catalytic Activity	Electron Carrier Activity	Enzyme Regulator Activity	Molecular Function	Molecular Transducer Activity	Structural Molecule Activity	Transporter Activity	Untreated 1	Untreated 2	Treated 1	Treated 2
Uncharacterized protein	A3M4F6_ACIBT	0.13	INF						X														X			X				0	0	0.37374	0.16184
Putative flavoprotein monooxygenase	A3MA01_ACIBT	0.42	0						X														X			X				0	0.19824	0	0
Uncharacterized protein	A3M8P0_ACIBT	0.34	1.9																											0.66973	0.1954	0.98207	0.62025
Uncharacterized protein	A3M8T6_ACIBT	0.0016	0.08																											4.295	4.4686	0.48062	0.20812
UDP-N-acetylmuramate:L-alanyl-gamma-D-glutamyl-meso-diaminopimelate ligase	A3M8X5_ACIBT	0.03	0.08		X				X													X	X			X				0.35344	0.2656	0	0.05018
GTPase HflX	A3M900_ACIBT	0.00039	0											X								X	X			X				0.35165	0.36587	0	0
DNA repair protein RecN	A3M1J1_ACIBT	0.042	0.08		X				X				X									X				X				0.1969	0.28132	0	0.03932
Transcriptional regulator LysR family	A3M732_ACIBT	0.78	0.9	X	X				X													X				X				0.10486	0.22905	0.15376	0.13979
Uncharacterized protein	A3M617_ACIBT	0.31	7.9																											0	0.24936	0.35145	1.6185
Protein GrpE	GRPE_ACIBT	0.063	0	X	X									X								X			X	X				0.4345	0.74376	0	0
Dihydrofolate reductase	A3M1W8_ACIBT	0.16	0		X				X													X	X			X				2.1485	0.81496	0	0
Putative DNA binding protein	A3M1E9_ACIBT	0.87	0.9	X																		X				X				1.3531	1.4078	1.9842	0.49141
Uncharacterized protein	A3M235_ACIBT	0.44	0.8																											2.209	2.2983	2.2714	1.4027
Putative nucleotidyl transferase	A3M4Y2_ACIBT	0.97	1						X														X			X				0.78808	0.57027	0.4977	0.87698
ATP-dependent dethiobiotin synthetase BioD	A3M2V3_ACIBT	0.72	0.8		X				X					X								X	X			X				0.81988	0.59181	0.8341	0.36118
Uncharacterized protein	A3MA13_ACIBT	0.21	1.3																											1.0041	1.0447	1.4724	1.1568
N utilization substance protein B homolog	NUSB_ACIBT	0.25	0.6	X	X				X													X				X				0.89483	0.93099	0.78278	0.33896
Uncharacterized protein	A3M210_ACIBT	0.034	0.4																			X				X				1.1428	0.92572	0.42874	0.29697
Glutamate/aspartate transport protein	A3M4S7_ACIBT	0.84	0.9				X	X	X								X		X			X	X			X				0.30927	0.51648	0.4535	0.31521
Acetolactate synthase isozyme III small subunit	A3M253_ACIBT	0.95	1		X				X													X	X			X				1.2465	1.2969	1.8278	0.79149
Peptide deformylase	A3M157_ACIBT	0.2	0.4		X				X													X	X			X				4.0098	2.0259	1.0626	1.2364
Acetoacetyl-CoA transferase beta subunit	A3M5G4_ACIBT	0.79	1.1						X														X			X				0.17635	0.39764	0.2586	0.39525
Integration host factor subunit beta	A3M506_ACIBT	0.49	0.5	X	X				X							X						X				X				3.8853	0.93766	0.55953	1.6336
Uncharacterized protein	A3M821_ACIBT	0.33	3																											0.45132	0.46956	0.66179	2.1146
Uncharacterized protein	A3M3E1_ACIBT	0.33	0.7																											0.81941	0.85253	0.32665	0.77166
Uncharacterized protein	A3M557_ACIBT	0.98	1						X														X			X				0.94196	2.0818	2.0754	0.89868
Peptidyl-prolyl cis-trans isomerase	A3M0S1_ACIBT	0.16	0.3		X				X														X			X				2.1485	1.2039	0.31454	0.73475
L-24-diaminobutyrate decarboxylase	A3M7H7_ACIBT	0.19	4.3		X				X													X	X			X				0.06965	0	0.10213	0.1952
Putative siderophore biosynthesis protein	A3M7U5_ACIBT	0.026	INF																											0	0	0.11204	0.15635
Putative acinetobactin biosynthesis protein	A3M7B5_ACIBT	0.13	INF																											0	0	0.31942	0.13832
Uncharacterized protein	A3M5Z7_ACIBT	0.42	INF																											0	0	0	0.32598
Universal stress protein	A3M196_ACIBT	0.2	0.2										X																	0.6084	0.28061	0.1865	0
Histidinol-phosphate aminotransferase	HIS8_ACIBT	0.047	0.1		X				X													X	X			X				0.44599	0.60793	0	0.12914
Putative oxidoreductase protein putative dehydrogenase (Flavoprotein)	A3M229_ACIBT	0.19	4																											0	0.17305	0.2439	0.44839
Methionine aminopeptidase	A3M752_ACIBT	0.14	0.1		X				X													X	X			X				0.44396	0.87343	0.19214	0
Choline dehydrogenase	A3M364_ACIBT	0.22	0						X													X	X			X				0.80702	0.22883	0	0
Putative homoserine kinase (ThrH)	A3M8E2_ACIBT	0.11	4.9		X				X														X			X				0.1737	0.39122	1.7681	1.0021
Uncharacterized protein	A3M3H5_ACIBT	0.09	0.04																											0.97022	1.8219	0	0.11861
Integrase	A3M513_ACIBT	0.42	INF		X				X													X				X				0	0	0	0.6034
Uncharacterized protein	A7FBI1_ACIBT	0.73	1.1																											1.2793	1.8645	1.876	1.535
Transcriptional factor	A3M6R4_ACIBT	0.69	1.1																											0.33684	0.22269	0.31386	0.2994
Uncharacterized protein	A7FAS7_ACIBT	0.48	2.4																											0.67123	1.1998	3.7516	0.73222
Uncharacterized protein	A7FB63_ACIBT	0.001	0.1																											7.7485	8.0617	1.289	0.99293

Identified Proteins (1398)	Accession Number	T-Test (p-value): (p < 0.05)	Fold Change Untreated vs. Treated	Biological Regulation	Cellular Process	Developmental Process	Establishment of Localization	Localization	Metabolic Process	Multi-organisim Process	Reproduction	Reproductive Process	Response to Stimulus	Cytoplasm	Extracellular Region	Intracellular Organelle	Membrane	Organelle Part	Plasma Membrane	Ribosome	Antioxidant Activity	Binding	Catalytic Activity	Electron Carrier Activity	Enzyme Regulator Activity	Molecular Function	Molecular Transducer Activity	Structural Molecule Activity	Transporter Activity	Normalized emPAI Values			
																														Untreated 1	Untreated 2	Treated 1	Treated 2
Iron-sulfur cluster insertion protein ErpA	ERPA_ACIBT	0.87	1.1		X				X													X				X		X		0.81848	1.5	2.114	0.51971
NADH-quinone oxidoreductase subunit H	NUOH_ACIBT	0.52	0.7						X								X		X			X	X			X				0.21945	0.10877	0.1533	0.06638
Putative phosphoglycolate phosphatase 2 (PGP 2)	A3M0T6_ACIBT	0.24	0.5						X														X			X				0.52101	0.7768	0.22144	0.47408
Uncharacterized protein	A3M3M4_ACIBT	0.59	1.6																											0.29486	0.30678	0.20288	0.73337
Uncharacterized protein	A3M239_ACIBT	0.71	1.1																											0.60243	1.0638	0.88338	0.98357
Uncharacterized protein	A7FAV9_ACIBT	0.018	5.4																											0.10985	0.11429	0.53531	0.67026
Uncharacterized protein	A3M5W2_ACIBT	0.047	0.5																											1.8816	1.9577	0.78278	1.1948
Peptidyl-prolyl cis-trans isomerase	A3M572_ACIBT	0.058	0.2						X														X			X				1.0875	0.76965	0.29973	0.12979
Iron-binding protein	A3M564_ACIBT	0.0033	0.1		X				X													X				X				2.6268	2.733	0.43927	0.19021
Uncharacterized protein	A3M262_ACIBT	0.13	INF																											0	0	0.28974	0.12546
Uncharacterized protein	A3M878_ACIBT	0.026	0.1																											0.5032	0.52353	0	0.14422
Putative high affinity choline transport protein (Bet-like)	A3M9U3_ACIBT	0.3	0.7				X	X									X		X							X			X	0.10818	0.17309	0.07738	0.10564
Transcriptional regulator LysR family	A3M4F5_ACIBT	0.2	0.2	X	X				X													X				X				0.11307	0.2479	0	0.0718
Uncharacterized protein	A3M9Q6_ACIBT	0.64	0.6																											0.0906	0.09426	0	0.12
Putative transcriptional regulator	A3M511_ACIBT	0.16	INF	X	X				X													X				X				0	0	0.14881	0.39046
Putative membrane protein	A3M1T9_ACIBT	0.64	0.4																											0.29582	0	0	0.12
Putative Holliday junction resolvase	A3M1I9_ACIBT	0.33	0.4		X				X				X	X								X	X			X				0.54628	0.9549	0	0.58277
Protease HtpX	HTPX_ACIBT	0.41	0.5						X								X		X			X	X			X				0.25582	0.26615	0	0.25788
Transcriptional regulator Cro/CI family	A3M515_ACIBT	0.049	0.07																			X				X				0.57191	0.85795	0	0.10404
GTP cyclohydrolase-2	RIBA_ACIBT	0.046	0		X				X													X	X			X				0.63748	0.40655	0	0
ADP-ribose pyrophosphatase	A3M6U4_ACIBT	0.42	INF						X														X			X				0	0	0	0.23372
Putative chromosomal replication initiator DnaA-type	A3M7X6_ACIBT	0.01	0.09	X																										0.53695	0.55865	0	0.09846
Imidazole glycerol phosphate synthase subunit HisF	HIS6_ACIBT	0.14	9.5		X				X					X									X			X				0	0.15247	0.45963	0.99529
Glutaminase	A3M3A0_ACIBT	0.16	3.5		X				X														X			X				0.08125	0	0.11914	0.167
Glucosamine--fructose-6-phosphate aminotransferase	A3MA41_ACIBT	0.049	0.1						X													X	X			X				0.77943	1.094	0	0.21344
CsuE	A3M6U6_ACIBT-DECOY	1	INF																											0	0	0	-1
UDP-N-acetylglucosamine 2-epimerase	A3M8Q9_ACIBT	0.053	0.6		X				X														X			X				0.42305	0.44015	0.28408	0.19275
High affinity Zn transport protein	A3M135_ACIBT	0.95	1				X	X														X				X				0.28403	0.29551	0.41649	0.18035
Inositol-1-monophosphatase	A3M9A8_ACIBT	0.7	1.4		X				X																					0.27835	0.46191	0.19214	0.85049
Cro-like protein	A3MAB0_ACIBT	0.27	4.1																											0.39076	0.40655	2.4346	0.80288
Putative signal peptide	A3M7F8_ACIBT	0.84	1.1																											1.0129	2.2722	2.2479	1.3867
Transketolase	A3M4V4_ACIBT	0.78	0.9						X														X			X				0.31487	0.15311	0.2158	0.19993
Putative glutathione S-transferase	A3M4S3_ACIBT	0.0019	0.4						X														X			X				0.45161	0.46986	0.19509	0.17961
SsrA-binding protein	SSRP_ACIBT	0.5	0.8											X								X				X				0.49607	0.51612	0.32876	0.52432
Acetoin:26-dichlorophenolindophenol oxidoreductase beta subunit	A3M5D3_ACIBT	0.29	1.7						X														X			X				0.22736	0.1125	0.33338	0.22783
Acyl-[acyl-carrier-protein]--UDP-N-acetylglucosamine O-acyltransferase	A3M648_ACIBT	0.49	2		X				X					X									X			X				0.29994	0.14628	0.70464	0.19045
RecBCD enzyme subunit RecD	A3M1M4_ACIBT	0.12	1.7		X				X														X			X				0.05928	0.06167	0.08692	0.11938
Glutamate--cysteine ligase	A3MA11_ACIBT	0.00039	0		X				X													X	X			X				0.18495	0.19243	0	0
Putative esterase	A3M6E2_ACIBT	0.03	0.1																											0.1734	0.18041	0	0.05296
NolF secretion protein	A3M8H9_ACIBT	0.31	0.6				X	X									X													0.20161	0.32923	0.14136	0.20093
Putative phospholipase D endonuclease domain	A3M8Q3_ACIBT	0.16	3.5		X				X														X			X				0.07108	0	0.10422	0.14473
Putative ABC1 protein	A3M1Q7_ACIBT	0.15	0.09						X														X			X				0.16713	0.37533	0	0.05111
Uncharacterized protein	A3M8D7_ACIBT	0.16	0.3																											0.4384	0.45612	0	0.27837

Identified Proteins (1398)	Accession Number	T-Test (p-value): (p < 0.05)	Fold Change Untreated vs. Treated	Biological Regulation	Cellular Process	Developmental Process	Establishment of Localization	Localization	Metabolic Process	Multi-organisim Process	Reproduction	Reproductive Process	Response to Stimulus	Cytoplasm	Extracellular Region	Intracellular Organelle	Membrane	Organelle Part	Plasma Membrane	Ribosome	Antioxidant Activity	Binding	Catalytic Activity	Electron Carrier Activity	Enzyme Regulator Activity	Molecular Function	Molecular Transducer Activity	Structural Molecule Activity	Transporter Activity	Normalized emPAI Values Untreated 1	Untreated 2	Treated 1	Treated 2
Histidine utilization repressor	A3MA54_ACIBT	0.042	0	X	X				X													X				X				0.47835	0.31073	0	0
Uroporphyrinogen decarboxylase	DCUP_ACIBT	0.097	0		X				X					X									X			X				0.20952	0.10406	0	0
Uncharacterized protein	A3M2E8_ACIBT	0.027	INF				X	X														X				X				0	0	0.11942	0.16742
Uncharacterized protein	A7FAW2_ACIBT	0.07	INF																											0	0	0.20179	0.35902
Putative membrane protein	A3M9V6_ACIBT	0.15	0.09				X	X									X													0.2281	0.52598	0	0.06888
Uncharacterized protein	A7FAZ1_ACIBT	0.16	0.3																											0.46563	0.48444	0	0.29566
Peptidyl-prolyl cis-trans isomerase	A3M7K5_ACIBT	0.1	0.2		X				X														X			X				4.3183	2.6235	0.34904	1.183
Thioredoxin	A3M2B1_ACIBT	0.23	0.5	X	X				X														X			X				3.4357	2.3994	2.142	0.92752
Uncharacterized protein	A3M189_ACIBT	0.065	2.6																											0.85893	0.89365	1.876	2.6102
IscRSUA operon repressor	A3M567_ACIBT	0.42	1.6																											0.52979	0.92328	0.77687	1.6011
50S ribosomal protein L21	RL21_ACIBT	0.26	2.2		X				X					X		X				X		X				X		X		1.5209	1.5824	2.2302	4.709
30S ribosomal protein S20	RS20_ACIBT	0.77	0.8		X				X					X		X				X		X				X		X		3.1903	1.1041	1.5561	2.0257
NADH-quinone oxidoreductase subunit A	NUOA_ACIBT	0.0016	0.4				X	X	X								X		X			X	X			X				1.4787	1.5384	0.64923	0.6803
NADH dehydrogenase FAD-containing subunit	A3M5Q9_ACIBT	0.31	0.6						X													X	X			X				0.1895	0.30865	0.1332	0.18837
30S ribosomal protein S12	RS12_ACIBT	0.71	1.1		X				X					X		X		X		X		X				X		X		0.68422	1.2258	1.0033	1.1508
50S ribosomal protein L25	RL25_ACIBT	0.021	3.7		X				X					X		X				X		X				X		X		0.91274	0.40139	2.3938	2.5133
Tryptophan synthase alpha chain	TRPA_ACIBT	0.033	0.08		X				X														X			X				0.6819	0.49768	0	0.08892
2,3,4,5-tetrahydropyridine-2,6-dicarboxylate N-succinyltransferase	DAPD_ACIBT	0.91	1		X				X					X									X			X				0.28637	0.29794	0.19736	0.41304
Phosphofructokinase	A3M629_ACIBT	0.00059	0.1		X				X													X	X			X				1.1212	1.1665	0.16856	0.15393
Orotate phosphoribosyltransferase	PYRE_ACIBT	0.081	4.1		X				X													X	X			X				0.17198	0.17893	0.88665	0.55576
Cat operon transcriptional regulator (LysR family)	A3M5S5_ACIBT	0.083	2.6	X	X				X													X				X				0.11533	0.11999	0.35673	0.2446
Esterase	A3M9Y9_ACIBT	0.00075	3						X														X			X				0.11611	0.1208	0.35925	0.34727
Putative transport protein	A3M377_ACIBT	0.88	1.3						X														X			X				0	0.08513	0	0.10794
Putative transcriptional regulator	A3M1I6_ACIBT	0.00039	0	X	X				X													X				X				0.65655	0.68308	0	0
DNA polymerase IV	A3M173_ACIBT	0.034	1.4		X				X				X	X								X	X			X				0.09831	0.10229	0.14416	0.13067
Putative acyl-CoA dehydrogenase	A3M0Z8_ACIBT	0.097	0						X													X	X			X				0.2022	0.10057	0	0
Nucleotide-binding protein A1S_0588	Y588_ACIBT	0.42	INF																			X				X				0	0	0	0.25989
Uncharacterized protein	A3M845_ACIBT	0.68	2.1		X				X					X									X			X				0	0.16279	0	0.34445
Histidine kinase	A3M8B9_ACIBT	0.42	INF	X	X				X				X				X					X	X			X	X			0	0	0	0.10195
Putative flavin-binding monooxygenase	A3M3R2_ACIBT	0.69	2						X														X			X				0.07007	0	0	0.14254
Peptidyl-prolyl cis-trans isomerase	A3M8W4_ACIBT	0.13	0		X				X														X			X				0.21719	0.49848	0	0
Tyrosine recombinase XerC	A3M0R9_ACIBT	0.13	INF		X				X					X								X	X			X				0	0	0.3412	0.14775
DNA polymerase V component	A3M4H3_ACIBT	0.37	4.3																											0.17284	0.17982	0.25344	1.2678
Argininosuccinate synthetase	A3M3K5_ACIBT	0.022	0						X													X	X			X				1.9006	1.4048	0	0
Uncharacterized protein	A3M7T4_ACIBT	0.42	INF				X	X	X								X					X	X			X			X	0	0	0	0.1331
AdeA membrane fusion protein	A3M5I5_ACIBT	0.35	21				X	X									X													0	0.22883	4.2775	0.51243
Putative RND type efflux pump involved in aminoglycoside resistance (AdeT)	A3M995_ACIBT	0.77	0.6				X	X																						0	0.22977	0	0.14023
Uncharacterized protein	A3M2Z5_ACIBT	0.077	INF																											0	0	0.85398	0.46534
Nicotinate-nucleotide pyrophosphorylase	A3M0U5_ACIBT	0.11	0.04		X				X														X			X				1.3325	0.65271	0	0.08289
Uncharacterized protein	A3M1F3_ACIBT	0.018	0.08																											2.4968	2.0774	0	0.38615
5'-nucleotidase SurE	SURE_ACIBT	0.42	INF		X				X					X								X	X			X				0	0	0	0.3167
3-hydroxyacyl-[acyl-carrier-protein] dehydratase FabZ	FABZ_ACIBT	0.2	0.9		X				X					X									X			X				1.4153	1.4725	1.3813	1.2705
HtrA-like serine protease	A3M768_ACIBT	0.3	0.5						X														X			X				0.57191	1.1617	0.51788	0.36314

Identified Proteins (1398)	Accession Number	T-Test (p-value): ($p < 0.05$)	Fold Change Untreated vs. Treated	Biological Regulation	Cellular Process	Developmental Process	Establishment of Localization	Localization	Metabolic Process	Multi-organisim Process	Reproduction	Reproductive Process	Response to Stimulus	Cytoplasm	Extracellular Region	Intracellular Organelle	Membrane	Organelle Part	Plasma Membrane	Ribosome	Antioxidant Activity	Binding	Catalytic Activity	Electron Carrier Activity	Enzyme Regulator Activity	Molecular Function	Molecular Transducer Activity	Structural Molecule Activity	Transporter Activity	Normalized emPAI Values: Untreated 1	Untreated 2	Treated 1	Treated 2
Uncharacterized protein	A3MA99_ACIBT	0.68	1.3																											0.25098	0.16782	0.36803	0.15936
50S ribosomal protein L20	RL20_ACIBT	0.87	1.1		X				X					X		X				X		X				X		X		0.70468	1.2669	1.7855	0.44744
50S ribosomal protein L16	RL16_ACIBT	0.14	2		X				X					X		X				X		X				X		X		0.59732	0.62146	1.4852	0.97339
Phosphocarrier protein (HPr-like)	A3M296_ACIBT	0.32	2.2																											1.9423	1.1041	4.6781	2.0257
EsvF1	A3M9L5_ACIBT	0.73	1.3				X	X									X													0.28767	0.19141	0.12947	0.50597
Uncharacterized protein	A3MA38_ACIBT	0.28	0.6						X														X			X				1.1571	1.2039	0.31454	1.0204
Methionine biosynthesis protein	A3M1Y1_ACIBT	0.73	1.1																											0.39518	0.67133	0.57948	0.59589
Putative hydrolase	A3M2K6_ACIBT	0.76	1.1						X														X			X				0.27396	0.1343	0.18928	0.27721
Positive pho regulon response regulator	A3MA20_ACIBT	0.32	0.6	X	X				X				X									X				X				0.87295	0.54301	0.34432	0.55429
Uncharacterized protein	A3M422_ACIBT	0.064	2.7																											0.1997	0.20777	0.64111	0.45699
UDP-3-O-acylglucosamine N-acyltransferase	LPXD_ACIBT	0.007	0.2		X				X														X			X				0.62364	0.64884	0.15057	0.0652
Putative signal peptide	A3M8P2_ACIBT	0.43	1.4																											0.85893	0.53505	0.7541	1.1379
Putative RND family drug transporter	A3MA92_ACIBT	0.14	0.2				X	X									X													0.34236	0.22618	0	0.13804
High frequency lysogenization protein HflD homolog	HFLD_ACIBT	0.26	2.2											X			X		X											0.14411	0.14993	0.21131	0.4481
4-hydroxy-tetrahydrodipicolinate synthase	A3MA68_ACIBT	0.16	0.09		X				X					X									X			X				0.26258	0.61441	0	0.07874
RNA-splicing ligase RtcB	A3M8K1_ACIBT	0.091	INF		X				X													X	X			X				0	0	0.12914	0.25329
Uncharacterized protein	A7FBW7_ACIBT	0.23	0.5																											0.44396	0.65563	0.19214	0.40013
Alkyl hydroperoxide reductase subunit F	A3M3Y7_ACIBT	0.047	0																											0.71009	0.44927	0	0
Putative allophanate hydrolase subunit 1 and 2	A3M453_ACIBT	0.19	0						X														X			X				0.21791	0.07089	0	0
Phage integrase family protein	A3M2G6_ACIBT	0.42	INF		X				X													X				X				0	0	0	0.1792
Uncharacterized protein	A7FB89_ACIBT	0.18	3.8																											0	0.31438	0.44308	0.75627
Beta-lactamase	A3M792_ACIBT	0.57	0.3		X				X				X										X			X				0	0.19824	0	0.05798
Acetyl-CoA carboxylase	A3M915_ACIBT	0.42	INF						X														X			X				0	0	0	0.08825
Uncharacterized protein	A3M7J0_ACIBT	0.27	INF																											0	0	0.41096	2.0842
Uncharacterized protein	A7FAY1_ACIBT	0.039	6																											0	0.14058	0.42165	0.41496
Uncharacterized protein	A3M8Q8_ACIBT	0.3	2.2																											0.14351	0.14931	0.44951	0.19465
Putative secretion pathway ATPase	A3M719_ACIBT	0.0034	INF				X	X														X				X				0	0	0.0856	0.07619
Putative transcriptional regulator (TetR family)	A3MA18_ACIBT	0.028	0.1																			X				X				0.34108	0.35486	0	0.10071
Protein translocase subunit SecY	A3M964_ACIBT	0.16	0.3				X	X									X		X											0.16713	0.17389	0	0.10612
Phenylacetic acid degradation B	A3M4C1_ACIBT	0.25	0																											0.36946	1.6044	0	0
Alkyl hydroperoxide reductase subunit F	A3M3Y6_ACIBT	0.42	0						X													X	X			X				0	0.29672	0	0
DNA polymerase III subunit beta	A3M0Q5_ACIBT	0.062	INF		X				X					X								X	X			X				0	0	0.44513	0.76069
Guanylate kinase	A3M9H3_ACIBT	0.00039	0		X				X					X								X	X			X				1.9384	2.0167	0	0
UPF0301 protein A1S_0320	A3M1J0_ACIBT	0.054	1.9																											0.73464	0.76433	1.5828	1.2579
Uncharacterized protein	A3M2E5_ACIBT	0.0054	1.4																											0.24761	0.25762	0.36309	0.35137
MFS permease	A3M843_ACIBT	0.95	1				X	X									X									X			X	0.16713	0.17389	0.24507	0.10612
Uncharacterized protein	A3M4R0_ACIBT	0.33	1.2		X				X														X			X				0.60243	0.62678	0.88338	0.64924
CsuE	A3M6U6_ACIBT	0.46	1.2																											0.22736	0.23654	0.33338	0.22783
tRNA (guanine-N(7)-)-methyltransferase	A3M3Y2_ACIBT	0.0082	0.6		X				X														X			X				0.30655	0.31894	0.21044	0.19465
Putative biopolymer transport protein (ExbB)	A3M1W4_ACIBT	0.46	0.8				X	X									X									X			X	0.39743	0.41349	0.26814	0.41222
Uncharacterized protein	A3M1P4_ACIBT	0.0065	0.6																											0.37004	0.38499	0.25094	0.23496
Geranyltranstransferase	A3M8C9_ACIBT	0.0047	1.4		X				X														X			X				0.25582	0.26615	0.37512	0.36427
Uncharacterized protein	A3M1G5_ACIBT	0.032	4.3																											0.21319	0.48845	1.6834	1.3516

Identified Proteins (1398)	Accession Number	T-Test (p-value): (p < 0.05)	Fold Change Untreated vs. Treated	Biological Regulation	Cellular Process	Developmental Process	Establishment of Localization	Localization	Metabolic Process	Multi-organisim Process	Reproduction	Reproductive Process	Response to Stimulus	Cytoplasm	Extracellular Region	Intracellular Organelle	Membrane	Organelle Part	Plasma Membrane	Ribosome	Antioxidant Activity	Binding	Catalytic Activity	Electron Carrier Activity	Enzyme Regulator Activity	Molecular Function	Molecular Transducer Activity	Structural Molecule Activity	Transporter Activity	Normalized emPAI Values			
																														Untreated 1	Untreated 2	Treated 1	Treated 2
NADH dehydrogenase II	A3M7K4_ACIBT	0.24	2.1						X													X	X			X				0.08221	0.08554	0.12055	0.23452
3-methylglutaconyl-CoA hydratase	A3M4F8_ACIBT	0.023	1.4						X														X			X				0.13726	0.14281	0.20127	0.18565
Glutamate-1-semialdehyde 2,1-aminomutase	GSA_ACIBT	0.22	2.8		X				X					X								X	X			X				0.08462	0	0.12409	0.11178
Uncharacterized protein	A3M265_ACIBT	0.00081	3																											0.11457	0.1192	0.35425	0.34194
Diaminopimelate decarboxylase	A3MAA9_ACIBT	0.042	11		X				X													X	X			X				0.0874	0	0.57996	0.41008
Segregation and condensation protein B	A3M2V5_ACIBT	0.3	2.5		X									X																0.38858	0	0.5698	0.40235
Putative acinetobactin biosynthesis protein	A3M797_ACIBT	0.092	INF						X														X			X				0	0	0.13148	0.25845
Putative hemin storage signal peptide protein	A3M6P4_ACIBT	0.22	2.8						X														X			X				0.05666	0	0.08308	0.07388
Uncharacterized protein	A3M258_ACIBT	0.88	1.3																											0	0.12455	0	0.16066
Putative transcriptional regulator	A3M3E5_ACIBT	0.25	0	X																						X				0.33405	1.4078	0	0
Uncharacterized protein	A3M2V7_ACIBT	0.023	0.1																			X				X				0.91447	0.95143	0	0.24537
tRNA (cytidine(34)-2'-O)-methyltransferase	A3M867_ACIBT	0.11	0		X				X					X								X	X			X				0.50683	0.23787	0	0
Putative transcriptional regulator (LysR family)	A3M2M5_ACIBT	0.68	2.1	X	X				X													X				X				0.11457	0	0	0.24281
Putative signal peptide	A3M6L8_ACIBT	0.12	0																											0.18883	0.42811	0	0
Uncharacterized protein	A7FB59_ACIBT	0.12	0																											0.10615	0.23199	0	0
Uncharacterized protein	A7FAX0_ACIBT	0.24	0.3																											0.78746	0.43606	0	0.37655
Putative flavoprotein oxidoreductase	A3M2N4_ACIBT	0.4	0.4						X													X	X			X				0.83872	1.8125	0	1.1062
30S ribosomal protein S15	RS15_ACIBT	0.8	0.9		X				X					X		X				X		X				X		X		1.0021	1.891	1.4694	1.1541
Putative O-methyltransferase protein	A3M431_ACIBT	0.34	0.6						X														X			X				0.39076	0.66324	0.26396	0.40477
NAD-dependent aldehyde dehydrogenase	A3M7H6_ACIBT	0.041	1.4						X														X			X				0.07692	0.08003	0.11279	0.10124
Putative ATP-binding protein	A3M2S6_ACIBT	0.91	0.9																			X				X				0.40119	0.09209	0.27049	0.18316
Uncharacterized protein	A3M549_ACIBT	0.03	3.2																											0.14779	0.32908	0.74552	0.8013
Ribosome maturation factor RimP	RIMP_ACIBT	0.62	0.7											X																0.75019	1.5893	0.3033	1.2916
Peptide methionine sulfoxide reductase MsrB	MSRB_ACIBT	0.099	0.4		X				X				X									X	X			X				2.0887	1.5314	0.37748	0.93461
Tyrosine recombinase XerD	A3M1C2_ACIBT	0.24	5.2		X				X					X								X	X			X				0	0.11726	0.16527	0.44293
Putative glutathione S-transferase protein	A3M4J5_ACIBT	0.0093	0.2						X														X			X				1.1301	1.1757	0.30882	0.13373
Putative acyltransferase	A3M8C4_ACIBT	0.17	3.7						X														X			X				0.17029	0	0.24971	0.37959
Shikimate dehydrogenase (NADP(+))	AROE_ACIBT	0.00039	0		X				X													X	X			X				0.29612	0.30808	0	0
Cysteine synthase	A3M164_ACIBT	0.026	0.1		X				X														X			X				0.52839	0.54974	0	0.15078
Uncharacterized protein	A3M221_ACIBT	0.078	0.1						X														X			X				0.12749	0.20486	0	0.03932
Histidinol dehydrogenase	A3M2I7_ACIBT	0.77	0.6		X				X													X	X			X				0	0.18223	0	0.11121
Dihydroorotate dehydrogenase (quinone)	PYRD_ACIBT	0.098	INF		X				X								X		X				X			X				0	0	0.16057	0.32451
Indole-3-glycerol phosphate synthase	TRPC_ACIBT	0.00039	0		X				X														X			X				0.45161	0.46986	0	0
Uncharacterized protein	A3M4C2_ACIBT	0.029	0.1																											0.29362	0.30548	0	0.0875
Uncharacterized protein	A7FAU8_ACIBT	0.2	4.5																											0.13005	0	0.1907	0.39659
3-isopropylmalate dehydratase small subunit	LEUD_ACIBT	0.085	0.1		X				X					X									X			X				0.35497	0.59826	0	0.10454
Solanesyl diphosphate synthase	A3M8A1_ACIBT	0.16	3.6		X				X														X			X				0.1109	0	0.16262	0.23425
1-deoxy-D-xylulose-5-phosphate synthase	A3M9A9_ACIBT	0.093	0		X				X													X	X			X				0.11539	0.05847	0	0
Uncharacterized protein	A3M634_ACIBT-DECOY	1	INF																											0	0	0	0
Hydrolase isochorismatase family	A3M9S3_ACIBT	0.026	INF						X														X			X				0	0	0.55437	0.77075
Putative ribosome biogenesis GTPase RsgA	A3M240_ACIBT	0.013	0						X													X	X			X				0.60231	0.47772	0	0
EstB	A3M3D1_ACIBT	0.0021	INF																											0	0	0.16635	0.15182
Uncharacterized protein	A7FAX2_ACIBT	0.15	4.9																											0.23324	0.24266	0.75966	1.5511

Identified Proteins (1398)	Accession Number	T-Test (p-value): (p < 0.05)	Fold Change Untreated vs. Treated	Biological Regulation	Cellular Process	Developmental Process	Establishment of Localization	Localization	Metabolic Process	Multi-organisim Process	Reproduction	Reproductive Process	Response to Stimulus	Cytoplasm	Extracellular Region	Intracellular Organelle	Membrane	Organelle Part	Plasma Membrane	Ribosome	Antioxidant Activity	Binding	Catalytic Activity	Electron Carrier Activity	Enzyme Regulator Activity	Molecular Function	Molecular Transducer Activity	Structural Molecule Activity	Transporter Activity	Normalized emPAI Values Untreated 1	Untreated 2	Treated 1	Treated 2
Putative RND family drug transporter	A3M7Z3_ACIBT	0.86	1.3																											0.09534	0	0	0.12654
50S ribosomal protein L18	RL18_ACIBT	0.27	0.8		X				X					X		X				X		X				X		X		2.1159	2.2014	1.9842	1.3435
Putative type III effector	A3M1Q5_ACIBT	0.37	1.4																											2.2072	1.4618	2.0602	2.9728
Uncharacterized protein	A3M2A7_ACIBT	0.67	1.4																											0.66973	0.6968	1.4324	0.42526
WbbJ protein	A3M0V5_ACIBT	0.23	0.6																											0.71009	1.0825	0.6332	0.45088
Putative dehydratase	A3M6E3_ACIBT	0.65	0.8		X				X					X									X			X				0.41912	0.27423	0.3865	0.16736
Anhydro-N-acetylmuramic acid kinase	A3M0R6_ACIBT	0.47	1.2		X				X													X	X			X				0.19703	0.20499	0.28892	0.19617
Uncharacterized protein	A3M9Y8_ACIBT	0.16	0.7																											0.87295	0.90823	0.76531	0.55429
Uracil phosphoribosyltransferase	UPP_ACIBT	0.19	0.6		X				X													X	X			X				0.89725	0.64384	0.55739	0.39293
Putative outer membrane lipoprotein	A3M7M7_ACIBT	0.13	2.3				X	X																		X			X	0.17816	0.40205	0.56665	0.79189
Cell division topological specificity factor	MINE_ACIBT	0.49	0.6	X	X																									0.97486	0.42486	0.5988	0.25929
Uncharacterized protein	A3M8J5_ACIBT	0.37	4.6																											0.3075	0.31993	0.45091	2.4392
Regulatory protein LysR:LysR, substrate-binding	A3M6N9_ACIBT	0.32	1.6	X	X				X													X				X				0.1165	0.2558	0.36052	0.24733
Putative transcriptional regulator (TetR family)	A3M4T2_ACIBT	0.067	2.7	X	X				X													X				X				0.1858	0.19331	0.5929	0.41995
7-cyano-7-deazaguanine synthase	A3M7R5_ACIBT	0.79	1.1		X				X													X	X			X				0.16621	0.37311	0.24373	0.36914
Uncharacterized protein	A3M1E5_ACIBT	0.24	3.3																											0.75019	0.2152	2.24	0.96997
Homocysteine synthase	A3M9L9_ACIBT	0.16	3.5		X				X													X	X			X				0.08462	0	0.12409	0.17448
Putative anthranilate phosphoribosyltransferase	A3M710_ACIBT	0.097	0						X														X			X				0.21671	0.10747	0	0
Bifunctional protein FolD	FOLD_ACIBT	0.057	0		X				X														X			X				0.28519	0.47394	0	0
Uncharacterized protein	A3M9J8_ACIBT	0.099	0.1																											0.80007	1.4618	0	0.21839
Putative tRNA-i(6)A37 modification enzyme	A3M931_ACIBT	0.21	3.8		X				X													X	X			X				1.0035	0.61623	4.343	1.8806
Putative DNA polymerase III delta subunit	A3M248_ACIBT	0.5	0.5		X				X													X	X			X				0.34461	0.2276	0	0.30663
Ribosomal RNA small subunit methyltransferase H	RSMH_ACIBT	0.18	0.2		X				X					X									X			X				0.24075	0.11881	0	0.07251
Secreted trypsin-like serine protease	A3M7R9_ACIBT	0.42	INF						X														X			X				0	0	0	0.44201
Transcriptional regulator MerR family	A3M443_ACIBT	0.42	INF	X																		X				X				0	0	0	0.13425
Putative membrane protein	A3M6P8_ACIBT	0.028	0.1																											0.29866	0.31073	0	0.08892
Major membrane protein I (MMP-I)	A3M4J0_ACIBT	0.00012	INF																											0	0	0.5587	0.57127
Ribonuclease T	A3M3K2_ACIBT	0.27	0		X				X													X	X			X				0.83429	0.1721	0	0
Uncharacterized protein	A3MA46_ACIBT	0.42	0																											0.35139	0	0	0
Transaldolase	A3M6E8_ACIBT	0.56	1.8		X				X					X									X			X				0.10881	0.11321	0.33558	0.06909
Putative transcriptional regulator	A3M5M2_ACIBT	0.21	2.8	X	X				X													X				X				0.11849	0	0.17374	0.15892
Uncharacterized protein	A7FBN3_ACIBT	0.0012	INF	X	X				X													X				X				0	0	0.23691	0.22089
Uncharacterized protein	A3M8K7_ACIBT	0.00039	0																											1.2906	1.3428	0	0
Putative LysR-type transcriptional regulator	A3M7R0_ACIBT	0.48	2.5	X	X				X													X				X				0.38381	0.25223	1.3304	0.2437
Large-conductance mechanosensitive channel	MSCL_ACIBT	0.22	0.5				X	X									X		X							X			X	0.9851	1.5468	0.85414	0.36986
ATP synthase subunit a	ATP6_ACIBT	0.95	1		X		X	X	X								X		X				X			X			X	0.41091	0.42752	0.60254	0.26091
LPS-assembly lipoprotein LptE	A3M249_ACIBT	0.27	2.6		X												X													0.47265	0.81496	2.3565	1.0204
Ferric uptake regulator	A3M333_ACIBT	0.0011	0.4	X																		X				X				0.91002	0.9468	0.35636	0.34419
UTP--glucose-1-phosphate uridylyltransferase	A3M0W3_ACIBT	0.53	0.7		X				X														X			X				0.12489	0.27527	0.18314	0.0793
Uncharacterized protein	A3M1V5_ACIBT	0.11	0.5																											0.42916	0.44651	0.28786	0.12465
Pseudouridine synthase	A3M6K3_ACIBT	0.45	0.5		X				X													X	X			X				0.5159	0.15576	0.21952	0.09506
Uncharacterized protein	A3M428_ACIBT	0.12	3.7																											0.38643	0.18536	1.3409	0.79189
Uncharacterized protein	A3M7M9_ACIBT	0.28	1.7																											0.28287	0.13842	0.41479	0.28675

Identified Proteins (1398)	Accession Number	T-Test (p-value): (p < 0.05)	Fold Change Untreated vs. Treated	Biological Regulation	Cellular Process	Developmental Process	Establishment of Localization	Localization	Metabolic Process	Multi-organisim Process	Reproduction	Reproductive Process	Response to Stimulus	Cytoplasm	Extracellular Region	Intracellular Organelle	Membrane	Organelle Part	Plasma Membrane	Ribosome	Antioxidant Activity	Binding	Catalytic Activity	Electron Carrier Activity	Enzyme Regulator Activity	Molecular Function	Molecular Transducer Activity	Structural Molecule Activity	Transporter Activity	Normalized emPAI Values			
																														Untreated 1	Untreated 2	Treated 1	Treated 2
Uncharacterized protein	A3M213_ACIBT	0.16	0.4																											0.33134	0.55581	0.22637	0.09802
Putative acetyltransferase	A3M6K2_ACIBT	0.32	0.6						X														X			X				0.87295	0.54301	0.34432	0.55429
Pyridine nucleotide transhydrogenase (Proton pump) alpha subunit (Part1)	A3M275_ACIBT	0.097	0						X													X	X			X				0.20702	0.10287	0	0
Acetate kinase	A3M1Z3_ACIBT	0.42	INF		X				X					X								X	X			X				0	0	0	0.18891
Putative (RR)-butanediol dehydrogenase	A3M5D8_ACIBT	0.42	INF						X													X	X			X				0	0	0	0.13979
Ribosome maturation factor RimM	A3M9G6_ACIBT	0.16	0.3		X				X					X		X				X		X				X				0.42396	0.44109	0	0.2692
Ribonuclease HII	A3M3S6_ACIBT	0.031	6.6		X				X					X								X	X			X				0.19856	0	0.63713	0.66546
EsvE2	A3M742_ACIBT	0.00039	0																											0.58783	0.61159	0	0
Xanthine phosphoribosyltransferase	XPT_ACIBT	0.014	0.3		X				X					X									X			X				1.0184	1.0595	0.28464	0.44201
Histidine kinase	A3M1B6_ACIBT	0.42	INF	X	X				X				X				X					X	X			X	X			0	0	0	0.08915
Transcriptional regulator for ferulate or vanillate catabolism (GntR family)	A3M3Q0_ACIBT	0.67	0.7	X	X				X													X				X				0.15789	0.16427	0	0.21552
Imidazolonepropionase	HUTI_ACIBT	0.13	INF		X				X					X								X	X			X				0	0	0.42653	0.18469
Transcriptional regulator LysR family	A3M7J7_ACIBT	0.42	INF	X	X				X													X				X				0	0	0	0.16243
CatA3	A3M5S8_ACIBT	0.065	0.1		X				X													X	X			X				0.3954	0.25947	0	0.07498
Non-canonical purine NTP pyrophosphatase	NTPA_ACIBT	0.12	0.06		X				X													X	X			X				0.62615	1.2887	0	0.11255
Beta-ketoacyl-ACP synthase I	A3M302_ACIBT	0.26	0.2																											2.3375	6.5932	0.7363	1.4843
Putative glutamine-dependent NAD(+) synthetase (NAD(+) synthase)	A3M2Z7_ACIBT	0.7	2						X														X			X				0	0.10057	0	0.20154
Putative D-amino acid oxidase	A3M343_ACIBT	0.24	2.7						X														X			X				0	0.09784	0.1379	0.12475
Streptomycin 3''-adenylyltransferase	A3M122_ACIBT	0.0017	INF						X				X										X			X				0	0	0.19214	0.17674
Protein-export protein SecB	SECB_ACIBT	0.69	0.8		X		X	X						X								X				X				1.3379	0.93099	1.3121	0.56818
Short-chain dehydrogenase/reductase SDR	A3M0Y2_ACIBT	0.67	1.1						X														X			X				0.54528	0.35142	0.49529	0.49764
Putative hemagglutinin/hemolysin-related protein	A3M893_ACIBT	0.76	1.1																											0.20764	0.33951	0.30447	0.28965
Putative tRNA/rRNA methyltransferase	A3M7X0_ACIBT	0.0084	0.6		X				X													X	X			X				0.29866	0.31073	0.20534	0.18964
Uncharacterized protein	A3M876_ACIBT	0.13	2.8																											0.44275	0.46064	1.571	0.93891
Putative transcriptional regulator	A3M4Z4_ACIBT	0.3	2.2	X	X				X													X				X				0.16946	0.17631	0.53691	0.23249
Tn7 transposase A	A3M7S3_ACIBT	0.8	1.1		X				X													X	X			X				0.12626	0.27845	0.18514	0.27045
23S rRNA (guanosine-2'-O-)-methyltransferase RlmB	A3M1J4_ACIBT	0.0018	0.4		X				X					X								X	X			X				0.50842	0.52897	0.21672	0.20084
Universal stress protein	A3M861_ACIBT	0.27	0.4										X	X																1.4574	0.59616	0.37471	0.36383
Translation initiation factor IF-1	IF1_ACIBT	0.22	0.4		X				X					X								X				X				2.3855	2.4819	1.8549	0.32347
Uncharacterized protein	A3M681_ACIBT	0.17	1.3																											1.1637	1.2107	1.7064	1.3732
Probable cytosol aminopeptidase	AMPA_ACIBT	0.12	3.9						X					X								X	X			X				0	0.1618	0.35437	0.27483
Putative transcriptional regulator (LysR family)	A3M5V7_ACIBT	0.42	INF	X	X				X													X				X				0	0	0	0.14874
Fe/S biogenesis protein NfuA	NFUA_ACIBT	0.3	0.4		X				X													X				X				0.61166	0.39122	0	0.38838
ATP phosphoribosyltransferase	A3M2I6_ACIBT	0.13	5.4		X				X					X								X	X			X				0.34275	0	1.1686	0.68364
Uncharacterized protein	A3M7N7_ACIBT	0.01	0.09						X														X			X				0.49409	0.51406	0	0.0915
Aspartate carbamoyltransferase non-catalytic chain	A3M3X7_ACIBT	0.73	0.7						X														X			X				0.33453	0.16205	0	0.34269
Curved DNA-binding protein	A3M1N0_ACIBT	0.00039	0		X																	X				X				0.23505	0.24455	0	0
Uncharacterized protein	A3M3W9_ACIBT	0.24	0										X																	0.2694	1.074	0	0
Putative lipid II flippase MurJ	A3M0U7_ACIBT	0.42	INF	X	X		X	X	X								X		X											0	0	0	0.09024
Sul1delta fusion protein	A3M197_ACIBT	0.22	2.8				X	X									X									X			X	0.07365	0	0.10799	0.09679
Uncharacterized protein	A3M3N2_ACIBT	0.42	INF	X	X				X													X				X				0	0	0	0.20215
ATP synthase subunit delta	ATPD_ACIBT	0.32	0.7		X		X	X	X								X		X				X			X			X	0.75019	0.78051	0.3033	0.70111
Uncharacterized protein	A7FBT0_ACIBT	0.31	2.7																											0.11495	0.1196	0.16856	0.45367

Identified Proteins (1398)	Accession Number	T-Test (p-value): (p < 0.05)	Fold Change Untreated vs. Treated	Biological Regulation	Cellular Process	Developmental Process	Establishment of Localization	Localization	Metabolic Process	Multi-organisim Process	Reproduction	Reproductive Process	Response to Stimulus	Cytoplasm	Extracellular Region	Intracellular Organelle	Membrane	Organelle Part	Plasma Membrane	Ribosome	Antioxidant Activity	Binding	Catalytic Activity	Electron Carrier Activity	Enzyme Regulator Activity	Molecular Function	Molecular Transducer Activity	Structural Molecule Activity	Transporter Activity	Normalized emPAI Values: Untreated 1	Untreated 2	Treated 1	Treated 2
Putative transcriptional repressor	A3M0S6_ACIBT	0.085	4.4	X	X				X													X				X				0.36615	0	0.87168	0.74101
Thiol:disulfide interchange protein	A3M1C1_ACIBT	0.16	0.3																											0.28059	0.29193	0	0.17817
Bacterioferritin	A3M9H8_ACIBT	0.049	INF	X	X		X	X	X													X	X			X				0	0	0.32665	0.5203
Putative signal peptide	A3M4V6_ACIBT	0.95	1																											0.6871	0.71487	1.0075	0.43628
Putative preprotein translocase IISP family membrane subunit	A3M8S4_ACIBT	0.31	0.7																											2.3062	2.3994	2.142	0.92752
UPF0345 protein A1S_0323	Y323_ACIBT	0.81	0.9																											1.405	0.83241	1.1732	0.89211
Uncharacterized protein	A3M9I1_ACIBT	0.31	0.7																											0.66973	1.0163	0.59984	0.62025
Uncharacterized protein	A3M1E6_ACIBT	0.95	1																											1.359	1.414	1.9928	0.86294
Putative acyltransferase	A3M4T1_ACIBT	0.17	1.8						X														X			X				0.19856	0.20658	0.29115	0.45391
Methyl-directed mismatch repair enzyme	A3M6J8_ACIBT	0.22	3.5		X				X				X																	0.23481	0.54301	1.9094	0.82682
Uncharacterized protein	A7FBG2_ACIBT	0.019	1.4																											0.15789	0.16427	0.23152	0.21552
UPF0145 protein A1S_2738	A3M8A7_ACIBT	0.002	1.4																											0.33088	0.34425	0.48519	0.48611
Putative ATP-dependent protease	A3M7F2_ACIBT	0.79	1.3						X														X			X				0.48915	0	0.32457	0.31059
Putative cold shock protein	A3M6Z0_ACIBT	0.19	0.4	X	X				X					X								X				X				2.6553	1.4407	0.80545	0.87923
Argininosuccinate lyase	A3M1E0_ACIBT	0.77	0.6		X				X					X									X			X				0	0.22338	0	0.13633
Uncharacterized protein	A3M9L1_ACIBT	0.42	INF		X		X	X	X														X			X				0	0	0	0.15779
Uncharacterized protein	A3M2V9_ACIBT	0.27	0.1																											0.6871	0.19972	0	0.12189
Resolvase	A3MAB2_ACIBT	0.27	1.7		X				X													X	X			X				0.37602	0.18072	0.55138	0.38838
Pyridoxine/pyridoxamine 5'-phosphate oxidase	PDXH_ACIBT	0.18	0.2		X				X													X	X			X				0.33777	0.16352	0	0.0998
Uncharacterized protein	A3M827_ACIBT	0.072	0																											0.69091	1.2392	0	0
Histidine kinase	A3M8P6_ACIBT	0.11	0	X	X				X				X				X					X	X			X	X			0.07122	0.15321	0	0
Oxidoreductase short chain dehydrogenase/reductase family	A3M816_ACIBT	0.66	0.7						X														X			X				0.13835	0.14395	0	0.18723
Formimidoylglutamase	A3MA47_ACIBT	0.094	5.2		X				X													X	X			X				0.11808	0	0.3657	0.25107
Putative amino-acid transport protein	A3M8G1_ACIBT	0.28	0.4				X	X									X									X			X	0.23566	0.1579	0	0.14963
Malonyl CoA-acyl carrier protein transacylase	A3M2W1_ACIBT	0.0054	0.07						X														X			X				0.54059	0.56244	0	0.07299
Uncharacterized protein	A3M9M9_ACIBT	0.043	0.06						X														X			X				1.7289	1.1748	0	0.18388
Putative glycosyl transferase	A3M222_ACIBT	0.062	INF						X														X			X				0	0	0.44173	0.75337
Uncharacterized protein	A3M6M0_ACIBT	0.18	6.9	X	X				X				X									X				X				0	0.15247	0.73839	0.31974
UPF0178 protein A1S_2615	Y2615_ACIBT	0.05	0																											0.88012	0.54708	0	0
Dephospho-CoA kinase	A3M1J6_ACIBT	0.086	0.1		X				X					X								X	X			X				0.38643	0.65534	0	0.11313
Putative Rhodanese-related sulfurtransferase	A3M8T5_ACIBT	0.3	0.4						X														X			X				2.5312	1.6501	0	1.6072
CsuB	A3M6U9_ACIBT	0.11	0																											0.573	0.26587	0	0
Uncharacterized protein	A3M7R7_ACIBT	0.038	INF																											0	0	0.2284	0.34269
Thioredoxin reductase	A3M313_ACIBT	0.014	0		X				X				X	X							X	X	X			X				0.7322	0.57573	0	0
Uncharacterized protein	A7FBA2_ACIBT	0.18	0																											3.7502	1.2447	0	0
Putative ferredoxin-dependent glutamate synthase	A3M6C9_ACIBT	0.42	INF		X				X														X			X				0	0	0	0.23372
Uncharacterized protein	A3M8I2_ACIBT	0.00039	0																											4.5199	4.7026	0	0
Preprotein translocase IISP family, membrane subunit	A3M8S6_ACIBT	0.95	1																											0.75741	0.78802	1.1106	0.48093
Glutaredoxin	A3M238_ACIBT	0.95	1	X	X				X														X	X		X				1.0612	1.1041	1.5561	0.67381
Uncharacterized protein	A3M128_ACIBT	0.58	1.4																											0.1737	0.63638	0.55138	0.56266
Putative membrane protein	A3M890_ACIBT	0.027	1.4																											0.12139	0.1263	0.17801	0.16303
Replicative DNA helicasechromosome replication chain elongation	A3M6Q7_ACIBT	0.3	2.5		X				X													X	X			X				0.31587	0.75572	1.8473	0.79994
Putative membrane protein	A3M944_ACIBT	0.77	0.9																											0.2106	0.48199	0.30882	0.29416

Identified Proteins (1398)	Accession Number	T-Test (p-value): (p < 0.05)	Fold Change Untreated vs. Treated	Biological Regulation	Cellular Process	Developmental Process	Establishment of Localization	Localization	Metabolic Process	Multi-organisim Process	Reproduction	Reproductive Process	Response to Stimulus	Cytoplasm	Extracellular Region	Intracellular Organelle	Membrane	Organelle Part	Plasma Membrane	Ribosome	Antioxidant Activity	Binding	Catalytic Activity	Electron Carrier Activity	Enzyme Regulator Activity	Molecular Function	Molecular Transducer Activity	Structural Molecule Activity	Transporter Activity	Normalized emPAI Values			
																														Untreated 1	Untreated 2	Treated 1	Treated 2
A/G specific adenine glycosylase	A3MA77_ACIBT	0.22	2.8		X				X				X									X	X			X				0.09587	0	0.14057	0.12728
Pirin-related protein	A3M125_ACIBT	0.2	0.2																											0.11198	0.24538	0	0.0711
Phycobiliprotein putative	A3M5F6_ACIBT	0.42	INF																											0	0	0	0.14158
2-amino-4-hydroxy-6-hydroxymethyldihydropteridine pyrophosphokinase	A3M291_ACIBT	0.31	8		X				X														X			X				0.22566	0	0.3309	1.4738
Methyl-directed mismatch repair	A3M436_ACIBT	0.12	0		X				X				X									X				X				0.1737	0.39122	0	0
Type III pantothenate kinase	COAX_ACIBT	0.33	2.2		X				X					X								X	X			X				0.14531	0.32321	0.73138	0.3167
Uncharacterized protein	A7FBB2_ACIBT	0.39	0.3																											0.93377	0.25827	0	0.35235
Glutamate:aspartate symport protein (DAACS family)	A3M1U0_ACIBT	0.42	INF				X	X									X									X			X	0	0	0	0.17634
Putative glycoprotein endopeptidase metalloprotease	A3M2J9_ACIBT	0.14	0.1		X				X														X			X				0.58136	1.1837	0	0.22771
Porphobilinogen deaminase	A3M1E3_ACIBT	0.42	INF		X				X														X			X				0	0	0	0.15779
Ribonuclease P protein component	A3M8Z2_ACIBT	0.17	8.1		X				X													X	X			X				0.27364	0	1.5432	0.66822
Integration host factor subunit alpha	IHFA_ACIBT	0.041	0.2	X	X				X													X				X				1.5638	1.627	0.54756	0
Uncharacterized protein	A7FBR3_ACIBT	0.42	INF																											0	0	0	0.36682
Uncharacterized protein	A3MA22_ACIBT	0.42	INF						X														X			X				0	0	0	0.18945
Ribosomal RNA small subunit methyltransferase B	A3MAA2_ACIBT	0.21	0	X	X				X					X								X	X			X				0.08087	0.27226	0	0
Uncharacterized protein	A3M264_ACIBT	0.42	0																											0	0.49175	0	0
Uncharacterized protein	A3M618_ACIBT	0.00039	0																											0.58735	0.61109	0	0
Putative transport protein	A3M3C4_ACIBT	0.42	INF						X													X	X			X				0	0	0	0.34269
Putative transcriptional regulator (LysR family)	A3M0Z5_ACIBT	0.42	INF	X	X				X													X				X				0	0	0	0.15779
Uncharacterized protein	A3M7D1_ACIBT	0.17	3.7						X														X			X				0.16542	0	0.24256	0.36712
Putative aminomutase	A3M7E4_ACIBT	0.096	INF						X													X	X			X				0	0	0.15013	0.30042
Putative deoxyribonuclease	A3M4Z3_ACIBT	0.0086	0.6		X				X														X			X				0.29362	0.30548	0.20207	0.18644
Ferric enterobactin receptor	A3M3B9_ACIBT	0.0098	1.4				X	X									X									X	X			0.20808	0.21649	0.30512	0.29032
Putative glutathionine S-transferase	A3M3Y5_ACIBT	0.015	1.4						X														X			X				0.17458	0.18163	0.25599	0.24005
Putative antigen	A3M3H1_ACIBT	0.2	0.2														X													0.13672	0.30293	0	0.08681
Putative organic radical activating enzyme	A3M7R4_ACIBT	0.2	2.9																											0	0.48749	0.68706	0.72722
Uncharacterized protein	A3M852_ACIBT	0.25	1.3																			X				X				0.83774	0.8716	1.2284	0.93993
Uncharacterized protein	A3M5P4_ACIBT	0.0002	3																											0.13726	0.14281	0.42874	0.42278
Uncharacterized protein	A3M8M9_ACIBT	0.071	0.5						X													X	X			X				1.8473	1.9219	0.62056	1.173
PKHD-type hydroxylase A1S_0473	Y473_ACIBT	0.23	2.7						X													X	X			X				0	0.1599	0.22536	0.2094
NADH dehydrogenase I chain E	A3M2Q1_ACIBT	0.16	0.2						X													X	X			X				0.47265	0.81496	0	0.30012
UPF0271 protein A1S_1267	Y1267_ACIBT	0.058	0						X														X			X				0.3163	0.52897	0	0
Superoxide dismutase [Cu-Zn]	A3M9E6_ACIBT	0.19	0.2		X				X				X								X	X	X			X				0.46022	0.21779	0	0.13292
Putative transcriptional regulator (TetR/AcrR family)	A3M9T9_ACIBT	0.16	1.8	X	X				X													X				X				0.17635	0.18348	0.2586	0.39525
Putative MutT/nudix family protein	A3M2J6_ACIBT	0.12	0						X														X			X				0.19091	0.43321	0	0
Uncharacterized protein	A3M261_ACIBT	0.00039	0																											0.39841	0.41451	0	0
Succinylornithine transaminase	A3M9D5_ACIBT	0.023	0						X													X	X			X				0.65351	0.47808	0	0
Putative signal peptide	A3M8W6_ACIBT	0.041	0																											0.4225	0.27632	0	0
Uncharacterized protein	A3M5R6_ACIBT	0.42	0																											0.27182	0	0	0
Phosphoserine phosphatase	A3MA31_ACIBT	0.86	1.3		X				X														X			X				0.09482	0	0	0.12582
Ribosomal silencing factor RsfS	A3M279_ACIBT	0.025	0.1	X	X									X																0.64664	0.67277	0	0.18087
Putative transcription regulator protein	A3M162_ACIBT	0.42	INF	X	X				X													X				X				0	0	0	0.23876
L-carnitine dehydrogenase	A3M2L9_ACIBT	0.42	INF						X														X			X				0	0	0	0.18067

Identified Proteins (1398)	Accession Number	T-Test (p-value): (p < 0.05)	Fold Change Untreated vs. Treated	Biological Regulation	Cellular Process	Developmental Process	Establishment of Localization	Localization	Metabolic Process	Multi-organisim Process	Reproduction	Reproductive Process	Response to Stimulus	Cytoplasm	Extracellular Region	Intracellular Organelle	Membrane	Organelle Part	Plasma Membrane	Ribosome	Antioxidant Activity	Binding	Catalytic Activity	Electron Carrier Activity	Enzyme Regulator Activity	Molecular Function	Molecular Transducer Activity	Structural Molecule Activity	Transporter Activity	Normalized emPAI Values			
																														Untreated 1	Untreated 2	Treated 1	Treated 2
Putative sulfide dehydrogenase	A3M8W2_ACIBT	0.17	0						X														X			X				6.3029	2.2322	0	0
tRNA pseudouridine synthase B	A3M9G1_ACIBT	0.099	0		X				X													X	X			X				0.24761	0.12202	0	0
DNA-binding protein	A3M6R9_ACIBT	0.95	1	X																		X				X				1.0021	1.0426	1.4694	0.63628
Biotin carboxyl carrier protein of acetyl-CoA carboxylase (BCCP)	A3M693_ACIBT	0.00069	0.2		X				X					X									X			X				1.6524	1.7192	0.41096	0.4032
Multifunctional protein	A3M3C3_ACIBT	0.83	0.9																											0.42916	0.44651	0.62931	0.12465
Uncharacterized protein	A3M165_ACIBT	0.78	0.9																											0.40669	0.19435	0.27391	0.25823
Putative esterase	A3M2K3_ACIBT	0.12	0		X				X														X			X				0.12534	0.27632	0	0
Uncharacterized protein	A3M7J3_ACIBT	0.21	0.2																											0.27364	0.64328	0	0.17375
Uncharacterized protein	A3M2T8_ACIBT	0.76	1.1		X				X														X			X				0.30387	0.14808	0.20871	0.30937
50S ribosomal protein L32	RL32_ACIBT	0.47	0.5		X				X					X		X		X		X						X		X		1.594	0.64161	0	1.0122
Putative phosphoglycerate mutase related protein	A3M5V4_ACIBT	0.42	INF																											0	0	0	0.20361
Putative acyltransferase (PhnO)	A3M3X0_ACIBT	0.56	0.3						X														X			X				0	0.5866	0	0.15991
Uncharacterized protein	A3M2R6_ACIBT	0.42	INF																											0	0	0	0.57783
DNA-directed RNA polymerase subunit omega	RPOZ_ACIBT	0.42	0		X				X													X	X			X				0	1.0007	0	0
Uncharacterized protein	A7FAT6_ACIBT	0.45	3.8																											3.439	0	1.6457	11.489
Putative transcriptional regulator LysR family	A3M5N7_ACIBT	0.42	INF	X	X				X													X				X				0	0	0	0.14874
Esterase	A3M5T3_ACIBT	0.23	4.5		X				X														X			X				0.14003	0	0.43794	0.18964
Putative membrane protein	A3M300_ACIBT	0.42	INF																											0	0	0	0.32182
Cysteine desulfurase	A3M565_ACIBT	0.25	0.2						X													X	X			X				1.0021	3.0759	0	0.63628
Uncharacterized protein	A3M6X8_ACIBT	0.42	0																											0.34963	0	0	0
Nitrogen assimilation regulatory protein P-II 2	A3M199_ACIBT	0.16	0.3	X	X				X																X	X				0.79116	0.82314	0	0.50236
Putative signal peptide	A3M8F6_ACIBT	0.42	0																											0.3052	0	0	0
Acetyltransferase	A3M6U2_ACIBT	0.13	INF						X														X			X				0	0	0.30512	0.70651
Uncharacterized protein	A7FBM5_ACIBT	0.04	INF																											0	0	0.77105	1.1714
Uncharacterized protein	A3M1P8_ACIBT	0.42	INF																											0	0	0	0.23249
Putative toluene tolerance protein Ttg2F	A3M2I4_ACIBT	0.07	0																											2.0842	1.173	0	0
Putative poly(R)-hydroxyalkanoic acid synthase	A3M5B5_ACIBT	0.15	INF																											0	0	0.14057	0.36496
NADH dehydrogenase I chain L	A3M2Q8_ACIBT	0.97	1		X				X								X						X			X				0.18209	0.05983	0.17324	0.07502
Putative ferrous iron transport protein A	A3M1C3_ACIBT	0.58	1.5																			X				X				0.92454	0.40588	1.3557	0.58705
Putative esterase	A3MA05_ACIBT	0.16	0.3																											0.39743	0.41349	0	0.25235
Uncharacterized protein	A7FAW8_ACIBT	0.0021	1.4																											0.33729	0.35092	0.49458	0.49683
Uncharacterized protein	A3M2V8_ACIBT	0.23	4.5																											0.15789	0	0.4977	0.21552
Uncharacterized protein	A3M4L9_ACIBT	0.65	0.7																											0.11808	0.12286	0	0.15835
Uncharacterized protein	A3M8T7_ACIBT	0.11	0.1																											3.0635	5.6498	0.88873	0.38484
Preprotein translocase IISP family auxillary membrane component	A3M1K1_ACIBT	0.53	1.5		X		X	X									X									X			X	1.586	0.39271	1.3051	1.6072
Putative lipoprotein	A3M6S4_ACIBT	0.5	0.6																											0.3858	0.94963	0.56572	0.24497
UDP-2,3-diacylglucosamine hydrolase	A3M6J3_ACIBT	0.09	5.3		X				X					X									X			X				0.14117	0	0.44173	0.30652
Uncharacterized protein	A3M3K8_ACIBT	0.0017	INF																											0	0	0.1966	0.18109
Peptidylprolyl isomerase	A3M8J4_ACIBT	0.22	0.2		X				X														X			X				0.39452	0.97449	0	0.25051
Glutathione-dependent formaldehyde-activating GFA	A3M9R9_ACIBT	0.025	0.1						X														X			X				0.58249	0.60603	0	0.16468
Inorganic pyrophosphatase	A3M183_ACIBT	0.19	0.2		X				X					X								X	X			X				0.46327	0.21911	0	0.13373
Putative membrane protein	A3M9I2_ACIBT	0.37	0.2																											0.48626	0.1091	0	0.13979
D-methionine transport protein	A3M4R9_ACIBT-DECOY	1	INF																											0	0	0	0

																														Normalized emPAI Values			
Identified Proteins (1398)	Accession Number	T-Test (p-value): (p < 0.05)	Fold Change Untreated vs. Treated	Biological Regulation	Cellular Process	Developmental Process	Establishment of Localization	Localization	Metabolic Process	Multi-organisim Process	Reproduction	Reproductive Process	Response to Stimulus	Cytoplasm	Extracellular Region	Intracellular Organelle	Membrane	Organelle Part	Plasma Membrane	Ribosome	Antioxidant Activity	Binding	Catalytic Activity	Electron Carrier Activity	Enzyme Regulator Activity	Molecular Function	Molecular Transducer Activity	Structural Molecule Activity	Transporter Activity	Untreated 1	Untreated 2	Treated 1	Treated 2
Putative transcriptional regulator	A3M6I4_ACIBT	0.0026	INF	X	X								X									X				X				0	0	0.1308	0.11807
Anti-sigm factor ChrR	A3M4K3_ACIBT	0.42	INF																											0	0	0	0.21447
Putative DNA transformation protein (ComF)	A3M999_ACIBT	0.42	INF		X				X														X			X				0	0	0	0.22312
Uncharacterized protein	A7FBH2_ACIBT	0.42	INF																											0	0	0	0.27589
2-keto-D-gluconate reductase	A3M6X7_ACIBT	0.00039	0						X													X	X			X				0.36763	0.38249	0	0
Uncharacterized protein	A7FBT5_ACIBT	0.86	1.3																											0.89483	0	0	1.1948
1-deoxy-D-xylulose 5-phosphate reductoisomerase	DXR_ACIBT	0.42	0		X				X													X	X			X				0.19106	0	0	0
Ditrans,polycis-undecaprenyl-diphosphate synthase ((2E,6E)-farnesyl-diphosphate specific)	A3M656_ACIBT	0.42	0	X	X				X													X	X			X				0.29612	0	0	0
Uncharacterized protein	A3M126_ACIBT	0.42	0										X																	0.60243	0	0	0
AdeA membrane fusion protein	A3M5I4_ACIBT	0.0014	INF																											0	0	1.4445	1.3409
Uncharacterized protein	A3M621_ACIBT	0.14	0.3																											1.3873	0.82314	0.49942	0.21626
Urease accessory protein UreE	UREE_ACIBT	0.0042	0.6		X				X					X								X				X				0.5032	0.52353	0.33306	0.31951
Putative transcriptional repressor of for multidrug resistance pump (MarR family)	A3M5U9_ACIBT	0.78	1.1	X	X				X													X				X				0.20684	0.47261	0.3033	0.47634
Uncharacterized protein	A3M0Z2_ACIBT	0.044	2.9																											0.31587	0.32864	1.0651	0.79994
Uncharacterized protein	A3M1U8_ACIBT	0.0028	1.4																											0.28959	0.30129	0.42464	0.41825
Putative thioesterase	A3M7U0_ACIBT	0.21	2.8						X														X			X				0.13726	0	0.20127	0.18565
NAD kinase	A3M712_ACIBT	0.6	2.9		X				X					X								X	X			X				0	0.12328	0	0.35557
Organic solvent tolerance protein	A3M4Y0_ACIBT	0.6	1.7																											0	0.50539	0.32251	0.51243
Universal stress protein	A3M633_ACIBT	0.83	1.2										X	X																0	0.59616	0.37471	0.36383
Putative phosphoglycolate phosphatase protein	A3M1R7_ACIBT	0.42	3						X														X			X				0.34963	0	0.82962	0.222
Putative thioesterase	A3M7W3_ACIBT	0.22	2.8						X														X			X				0	0.26587	0.37471	0.36383
Uncharacterized protein	A3M4E7_ACIBT-DECOY	1	INF																											0	0	-1	0
Uncharacterized protein	A7FBM4_ACIBT	0.13	0																											0.25935	0.60603	0	0
Uncharacterized protein	A3M2W5_ACIBT	0.65	0.4		X				X													X	X			X				0.51338	0	0.21858	0
Uroporphyrinogen-III synthase	A3M1E4_ACIBT	0.11	INF		X				X														X			X				0	0	0.20048	0.4208
Putative transcriptional regulator	A3M269_ACIBT	0.27	INF	X	X				X													X				X				0	0	0.36439	0.07472
Capsular polysaccharide synthesis enzyme	A3M0V8_ACIBT	0.95	1																											0.41888	0.43581	0.61422	0.26597
Superoxide dismutase	A3M771_ACIBT	0.95	1		X				X				X								X	X	X			X				0.38012	0.39548	0.55739	0.24136
3',5'-cyclic adenosine monophosphate phosphodiesterase CpdA	A3M1D0_ACIBT	0.56	1.8						X													X	X			X				0.12908	0.1343	0.40172	0.08196
Uncharacterized protein	A3M3H6_ACIBT	0.0065	0.6																											0.37004	0.38499	0.25094	0.23496
Putative transcriptional repressor of for multidrug resistance pump (MarR family)	A3M631_ACIBT	0.22	2.8	X																						X				0	0.23632	0.33306	0.31951
Uncharacterized protein	A7FBC9_ACIBT	0.56	0.3																											0	0.56397	0	0.15431
Recombination protein RecR	A3M5B8_ACIBT	0.11	0		X				X				X									X				X				0.402	0.19228	0	0
Uncharacterized protein	A7FAW4_ACIBT	0.00039	0																											0.64921	0.67544	0	0
Uncharacterized protein	A3M8N0_ACIBT	0.42	0		X				X													X	X			X				0.28059	0	0	0
Transcriptional repressor of Zn transport system (Fur family)	A3M134_ACIBT	0.13	0	X																		X				X				0.22863	0.52732	0	0
Putative lipoprotein	A3M6S3_ACIBT	0.55	1.9																											0.21994	0.22883	0.7123	0.13966
Uncharacterized protein	A7FAV1_ACIBT	0.082	0.4																											0.93664	0.97449	0.57851	0.25051
Release factor glutamine methyltransferase	A3M6N6_ACIBT	0.52	0.7		X				X													X	X			X				0.27613	0.1353	0.1907	0.08258
Outer-membrane lipoprotein carrier protein	A3M227_ACIBT	0.21	2.8				X	X																						0.1737	0	0.25471	0.23876
Uncharacterized protein	A3M2P6_ACIBT	0.078	0.1																											1.2042	0.72592	0	0.19354
Uncharacterized protein	A7FAT0_ACIBT	0.48	0.5																											2.1627	1.2107	1.7064	0
50S ribosomal protein L28	RL28_ACIBT	0.2	2.9		X				X					X		X				X						X		X		0	0.48749	0.68706	0.72722

Identified Proteins (1398)	Accession Number	T-Test (p-value): (p < 0.05)	Fold Change Untreated vs. Treated	Biological Regulation	Cellular Process	Developmental Process	Establishment of Localization	Localization	Metabolic Process	Multi-organisim Process	Reproduction	Reproductive Process	Response to Stimulus	Cytoplasm	Extracellular Region	Intracellular Organelle	Membrane	Organelle Part	Plasma Membrane	Ribosome	Antioxidant Activity	Binding	Catalytic Activity	Electron Carrier Activity	Enzyme Regulator Activity	Molecular Function	Molecular Transducer Activity	Structural Molecule Activity	Transporter Activity	Normalized emPAI Values			
																														Untreated 1	Untreated 2	Treated 1	Treated 2
Uncharacterized protein	A3M6K1_ACIBT	0.023	0.1																											0.83774	0.8716	0	0.22737
Surface antigen	A3M4G7_ACIBT-DECOY	1	INF																											0	0	0	0
Uncharacterized protein	A3M4V9_ACIBT	0.2	2.9																											0	0.51505	0.7259	0.77623
Putative adenylate or guanylate cyclase	A3M559_ACIBT	0.27	INF	X	X				X				X										X			X				0	0	0.65755	0.12979
Deoxyuridine 5'-triphosphate nucleotidohydrolase	DUT_ACIBT	0.17	0.2		X				X													X	X			X				0.55932	0.98003	0.36662	0
Tetraacyldisaccharide 4'-kinase	A3M4Y9_ACIBT	0.16	INF		X				X													X	X			X				0	0	0.1547	0.40901
Putative poly(Hydroxyalcanoate) granule associated protein	A3M569_ACIBT	0.11	0																											0.53791	0.25109	0	0
Uncharacterized protein	A3M8U2_ACIBT	0.14	0																											0.32171	0.77154	0	0
Uncharacterized protein	A3M3D5_ACIBT	0.00052	INF																											0	0	0.31848	0.30422
Putative acetyltransferase	A3M610_ACIBT	0.42	INF						X														X			X				0	0	0	0.6803
Uncharacterized protein	A3M668_ACIBT	0.13	0																											0.21584	0.49509	0	0
Uncharacterized protein	A3M8Y7_ACIBT	0.82	1.5																											0	0.45123	0	0.66403
Putative fumarylacetoacetate hydrolase family protein	A3M943_ACIBT	0.12	0						X														X			X				0.16864	0.37896	0	0
Uncharacterized protein	A3M4F1_ACIBT	0.1	0																											0.28403	0.13895	0	0
Putative signal peptide	A3M6Y8_ACIBT	0.13	0																											0.31303	0.74805	0	0
Haemin storage system HmsR protein	A3M6P3_ACIBT	0.42	INF						X														X			X				0	0	0	0.10689
Uncharacterized protein	A3M8X9_ACIBT	0.42	INF																											0	0	0	0.11559
Uncharacterized protein	A7FAY0_ACIBT	0.5	0.6																											0.65262	0.29881	0.42114	0.18236
Uncharacterized protein	A3M583_ACIBT	0.85	1.4		X		X	X	X																	X			X	0.15369	0	0	0.2094
Uncharacterized protein	A7FAW7_ACIBT	0.48	0.4																											0.60608	1.6236	0	0.99086
Putative transcriptional regulator (TetR family)	A3M6C5_ACIBT	0.12	INF	X	X				X													X				X				0	0	0.26673	0.59589
Pyrroline-5-carboxylate reductase	A3M2B5_ACIBT	0.00039	0		X				X														X			X				0.29238	0.3042	0	0
Stringent starvation protein B	A3M910_ACIBT	0.85	1.4																											0	0.26587	0	0.36383
Uncharacterized protein	A3M6S8_ACIBT	0.42	INF																											0	0	0	5.2539
Oligopeptidase A	A3M949_ACIBT	0.72	0.7																											0.2872	0.29881	0	0.41439
Uncharacterized protein	A3M216_ACIBT	0.68	0.7																											0.17908	0.18632	0	0.24674
Uncharacterized protein	A7FBU3_ACIBT	0.42	INF																											0	0	0	0.78949
Uncharacterized protein	A7FBW1_ACIBT	0.42	INF																											0	0	0	0.78949
RNA pyrophosphohydrolase	RPPH_ACIBT	0.42	INF						X													X	X			X				0	0	0	0.50105
Preprotein translocase IISP family, membrane subunit	A3M8S7_ACIBT	0.42	INF																											0	0	0	0.34687
