# Supplementary material for: Exploring the Antimicrobial Action of Quaternary Amines against Acinetobacter baumannii
Source: mBio. 2018 Feb 6;9(1):e02394-17. doi: 10.1128/mBio.02394-17 (PMC5801471; doi:10.1128/mBio.02394-17)
Supplement: TABLE S5 [file mbo001183722st5.pdf]

Table S5. Bacterial Strains and Plasmids

Bacterial Strains and Plasmids	SOURCE	IDENTIFIER
Acinetobacter baumannii strain 17978	ATCC	ATCC 17978
Acinetobacter baumannii strain 19606	ATCC	ATCC 19606
Acinetobacter baumannii strain 5075	(65) Jacobs, et al.	5075
Acinetobacter baumannii strain AYE	ATCC	ATCC BAA-1710
Acinetobacter baumannii	Centre Hospitalier Universitaire de Caen	Clinical 1
Acinetobacter baumannii	Centre Hospitalier Universitaire de Caen	Clinical 2
Acinetobacter baumannii	Centre Hospitalier Universitaire de Caen	Clinical 3
Acinetobacter baumannii	Centre Hospitalier Universitaire de Caen	Clinical 4
Acinetobacter baumannii	Centre Hospitalier Universitaire de Caen	Clinical 5
Acinetobacter baumannii 17978 Δ*lon*::Kan^r	(23) Tucker, et al.	AT07
Acinetobacter baumannii 17978 Δ*oxyR*::Kan^r	(23) Tucker, et al.	AT09
Acinetobacter baumannii 17978 Δ*adeB*::Kan^r	(23) Tucker, et al.	AT16
Acinetobacter baumannii 17978 carrying pABBR_MCS	(23) Tucker, et al.	AT11
Acinetobacter baumannii 17978 Δ*lon*::Kan^r carrying pABBR_MCS	This study	AC1
Acinetobacter baumannii 17978 Δ*oxyR*::Kan^r carrying pABBR_MCS	(23) Tucker, et al.	AT12
Acinetobacter baumannii 17978 Δ*adeB*::Kan^r carrying pABBR_MCS	(23) Tucker, et al.	AT20
Acinetobacter baumannii 17978 Δ*lon*::Kan^r carrying pABBR_NPlon	This study	AC2
Acinetobacter baumannii 17978 Δ*oxyR*::Kan^r carrying pABBR_NPoxyR	(23) Tucker, et al.	AT10
Acinetobacter baumannii 17978 Δ*adeB*::Kan^r carrying pABBR_NPadeB	(23) Tucker, et al.	AT18

Acinetobacter baumannii 17978 R2 parental wild type	(26) Arroyo, et al.	17978
Acinetobacter baumannii 17978 R2	(26) Arroyo, et al.	R2
Acinetobacter baumannii 17978 BZK Mutant	This study	Mutant 1
Acinetobacter baumannii 17978 BZK Mutant	This study	Mutant 2
Acinetobacter baumannii 17978 BZK Mutant	This study	Mutant 3
Acinetobacter baumannii 17978 BZK Mutant	This study	Mutant 4
Acinetobacter baumannii 17978 BZK Mutant	This study	Mutant 5
Acinetobacter baumannii 17978 BZK Mutant	This study	Mutant 8
Acinetobacter baumannii 17978 BZK Mutant	This study	Mutant 9
Acinetobacter baumannii 17978 BZK Mutant	This study	Mutant 10
Acinetobacter baumannii 17978 BZK Mutant	This study	Mutant 11
Staphylococcus aureus Newman	Lab stock	Newman
Staphylococcus aureus MU50	Lab stock	MU50
Clostridium difficile 43255	ATCC	43255
Clostridium difficile 630	ATCC	630
pABBR_NPlon	This study	pABBR_NPlon
pABBR_NPoxyR	(23) Tucker, et al.	pABBR_NPoxyR
pABBR_NPadeB	(23) Tucker, et al.	pABBR_NPadeB
pABBR_MCS	(23) Tucker, et al.	pABBR_MCS
